# Mechanism of the Oxidative Ring-Closure Reaction during Gliotoxin Biosynthesis by Cytochrome P450 GliF

**DOI:** 10.3390/ijms25168567

**Published:** 2024-08-06

**Authors:** Muizz Qureshi, Thirakorn Mokkawes, Yuanxin Cao, Sam P. de Visser

**Affiliations:** Manchester Institute of Biotechnology, Department of Chemical Engineering, The University of Manchester, 131 Princess Street, Manchester M1 7DN, UKyuanxin.cao@manchester.ac.uk (Y.C.)

**Keywords:** density functional theory, enzyme catalysis, inorganic reaction mechanisms, cytochrome P450 enzymes, mono-oxygenases

## Abstract

During gliotoxin biosynthesis in fungi, the cytochrome P450 GliF enzyme catalyzes an unusual C–N ring-closure step while also an aromatic ring is hydroxylated in the same reaction cycle, which may have relevance to drug synthesis reactions in biotechnology. However, as the details of the reaction mechanism are still controversial, no applications have been developed yet. To resolve the mechanism of gliotoxin biosynthesis and gain insight into the steps leading to ring-closure, we ran a combination of molecular dynamics and density functional theory calculations on the structure and reactivity of P450 GliF and tested a range of possible reaction mechanisms, pathways and models. The calculations show that, rather than hydrogen atom transfer from the substrate to Compound I, an initial proton transfer transition state is followed by a fast electron transfer en route to the radical intermediate, and hence a non-synchronous hydrogen atom abstraction takes place. The radical intermediate then reacts by OH rebound to the aromatic ring to form a biradical in the substrate that, through ring-closure between the radical centers, gives gliotoxin products. Interestingly, the structure and energetics of the reaction mechanisms appear little affected by the addition of polar groups to the model and hence we predict that the reaction can be catalyzed by other P450 isozymes that also bind the same substrate. Alternative pathways, such as a pathway starting with an electrophilic attack on the arene to form an epoxide, are high in energy and are ruled out.

## 1. Introduction

Gliotoxin is a mycotoxin synthesized by several fungi types, including *Aspergillus fumigatus*, *Eurotium chevalieri*, *Gliocladium fimbriatum* and *Penicillium* species [1,2,3]. Myco-toxins are secondary metabolites of fungi that can cause disease and death in humans after intake due to their inhibition of cytokines released by leukocytes. Furthermore, gliotoxin has been reported to inhibit picornavirus RNA synthesis and can disrupt protein synthesis [3]. However, there are also beneficial health effects of gliotoxin intake in humans as it has been reported to have antiviral, antibacterial and immunosuppressive properties [4,5]. In particular, there are reports that it has antiviral activity against Nipah, Hendra and influenza A viruses [5]. Furthermore, gliotoxin may have benefits as a result of anti-cancer activity by inducing the apoptosis of cancer cells [6,7,8,9]. To be specific, it targets the neurogenic locus notch homolog 2 protein, as well as the Wnt/β-catenin pathway, and the transferases farnesyl transferase and geranyl transferase I. Besides these examples, gliotoxin can inhibit the growth of Adriamycin-resistant non-small-cell lung cancer cell lines by inducing mitochondria-dependent apoptosis. As such, gliotoxin is an important natural product; however, it is challenging to synthesize using organic chemistry approaches.

**Scheme 1:**
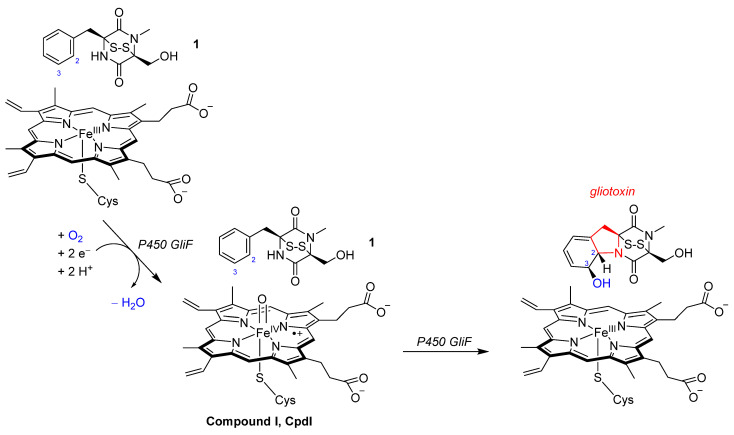
Oxidative ring-closure reaction in 1 catalyzed by P450 GliF as part of the gliotoxin biosynthesis through proton transfer from solvent or proton transfer from substrate channels.

Gliotoxin is an epipolythiodioxopiperazine compound that contains a transannular disulfide bridge across a piperazine ring; Scheme 1. It is synthesized in a cascade of reactions, whereby the oxidative ring-closure reaction of its precursor, namely (1*S*,4*S*)-1-benzyl-4-(hydroxymethyl)-5-methyl-2,3-dithia-5,7-diazabicyclo[2.2.2]octane-6,8-dione (**1**) as shown in Scheme 1, is catalyzed by a cytochrome P450 isozyme called P450 GliF [10,11,12,13]. This enzymatic step uses one molecule of dioxygen, two electrons from redox partners and two protons from the solvent to embed a five-ring into the substrate scaffold between the phenyl substituent and one of the nitrogen atoms of the piperazine group, as highlighted in red in Scheme 1. Experimental work showed that, in the same enzymatic cycle, the C_3_-position of the phenyl ring is hydroxylated [10,11,12,13]. Currently, it is unknown what the order of these reaction steps is and whether initially ring-closure takes place or oxygen atom transfer. Early biochemical studies suggested that first an epoxide intermediate is formed across the C_2_–C_3_ bond of the phenyl ring [10,12], which, thereafter, would react with the assistance of a water molecule to form gliotoxin products. This mechanism would implicate a reaction starting with oxygen atom transfer to the C_3_-position. However, substrate **1** has a weak N–H bond that should be susceptible to hydrogen atom abstraction by the P450 oxidant similarly to a phenolic O–H bond in the vancomycin biosynthesis [14,15,16]. Because the oxidative cyclization by P450 enzymes to form gliotoxin by P450 GliF is controversial, we decided to pursue a computational study and establish the details of its reaction cycle.

The P450s are widespread mono-oxygenases found in most forms of life that utilize a single dioxygen molecule and two electrons from a redox partner and two protons from the solvent [17,18,19,20,21,22,23,24,25,26,27,28,29]. In the catalytic cycle, the iron(III)-heme is converted into an active species called Compound I (CpdI), which is an iron(IV)–oxo heme cation radical species that reacts with the substrate (Scheme 1). Generally, P450s act as mono-oxygenases, whereby a single oxygen atom is transferred to the substrate, resulting in aliphatic or aromatic hydroxylation or double-bond epoxidation [20,24,27]. However, there are also reports of substrate desaturation and cyclization reactions by P450s [30,31,32,33,34,35,36,37]. The overall reaction performed by P450 GliF is interesting from a chemical synthesis point of view, particularly if it can be applied in biotechnology that will enable the biosynthesis of drug molecules or their precursors. Unfortunately, little is known about the mechanism and the general features of the protein that drive the reaction process in P450 GliF.

P450s are heme enzymes that bind the heme to the protein through a cysteinate ligand in the axial position (shown in Scheme 1) and accept dioxygen on the distal site [38,39]. In a number of P450 isozymes, an oxidative ring-closure reaction of the substrate has been reported [30,31,32,33,34,35,36,37,40,41,42,43]. This, for instance, can happen during lignin biodegradation through the formation of an acetal group from a phenol with an *ortho*-methoxy substituent by two consecutive hydrogen atom abstraction steps [40]. In addition, glycopeptide biosynthesis involves the linkage of two phenol groups by covalent bonds, whereby an initial phenolic hydrogen atom abstraction results in the coupling of two aromatic rings, which has been shown to take place during vancomycin biosynthesis [42,44]. In these examples, the substrate contains a weak phenolic O–H bond and its abstraction by CpdI triggers the reaction. However, the gliotoxin precursor does not contain a phenol group; hence, the gliotoxin biosynthesis must proceed via a different mechanism. In this work, we focus on understanding the oxidative ring-closure reaction of **1** by P450 GliF and tested a variety of possible pathways for substrate activation. This work shows that CpdI is the active oxidant but the rate-determining step is the ring-closure reaction.

## 2. Results

### 2.1. Model Set-Up and MD Simulations

**Figure 1:**
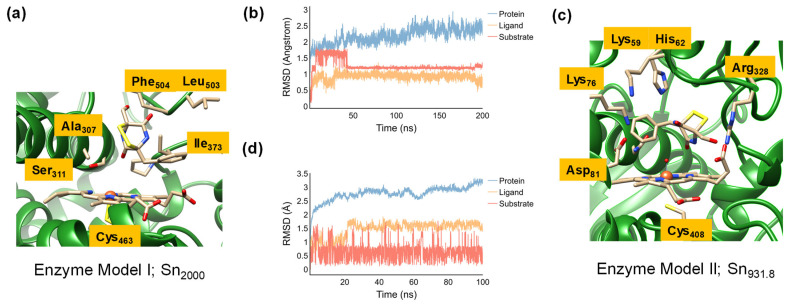
MD simulation results on enzyme model **I** (**a**,**b**) and enzyme model **II** (**c**,**d**) for binding of **1** into the structures. Key snapshots (Sn) shown of the MD simulations performed on enzyme model **I** and **II** (**a**,**c**) and RMSD profiles of the protein (in blue), heme ligand (in amber) and substrate (in red) are given as a function of time (**b**,**d**).

In the absence of crystal structure coordinates of P450 GliF, we created full enzyme structures based on a homology model (enzyme model **I**) and a recently proposed structure from AlphaFold version 2.3.2 (enzyme model **II**). These structures were fitted with heme and a substrate and solvated in water during the set-up and, thereafter, a molecular dynamics simulation was performed for both protein structures as described in the Methodology section. The enzyme model **I** set-up started from the 4UYL protein databank (pdb) file [45,46], which is a set of crystal structure coordinates from a P450 sterol-14a-demethylase (CYP51B) from *Aspergillus fumigatus* that is used for the biosynthesis of antifungal drugs. The CYP51B enzyme was reported to be a close homologue of P450 GliF based on biosynthetic gene clusters [10]. Moreover, its substrate is of similar size and molecular weight and the catalytic cycle operates under similar biochemical conditions. Hence, we reasoned that it may be a good model for studies on the gliotoxin biosynthesis reaction mechanism by P450 GliF, which may have a similar active site structure and shape. Subsequently, we inserted a substrate (**1** in Scheme 1) into the binding pocket. A 200 ns MD simulation was run at room temperature conditions without constraints on the atoms, and the last structure of the MD simulation (snapshot 2000, Sn_2000_) is shown in Figure 1a. The MD simulation on enzyme model **I** gives a very rigid structure, with the root-mean-square-deviation (RMSD) of the substrate, heme and protein that stabilizes within 50 ns; Figure 1b. The substrate is bound alongside the I-helix in Sn_2000_ in the vicinity of the side chains of the residues Ala_307_ and Ser_311_, although no clear hydrogen bonding interactions are seen. The phenyl group of the substrate is positioned in between the apolar side chains of Pro_372_, Ile_373_ and Leu_503_. However, the lack of polar and charged residues in the substrate-binding pocket of this enzyme may not position the substrate in the ideal orientation for catalysis, and hence we also analyzed an alternative structure based on P450 GliF; see below. We then used the *k*-clustering technique (Supporting Information Figure S2) and distributed all MD snapshots for enzyme model **I** into three groups. However, two of the orientations have the substrate relatively far from the heme and therefore cannot count as catalytically active structures, and hence were discarded. The average structure of the third group of MD conformations was selected to create a QM cluster model for mechanistic studies.

We also generated a P450 GliF structure (enzyme model **II**) based on the recently proposed structure from AlphaFold [47]. This protein structure has a similar fold as compared to the 4UYL pdb structure but has a number of charged and polar residues in the substrate-binding pocket, namely Lys_59_, Lys_60_, Lys_76_ and Asp_81_, that may incur electrostatic effects on the reaction mechanism. We therefore decided to compare the two structures for gliotoxin biosynthesis and find out whether the structural differences lead to substrate-binding and mechanistic differences during the catalysis. The full enzyme structure (enzyme model **II**) was set up in the same way as enzyme model **I**, where the substrate was inserted into the substrate-binding pocket and hydrogen atoms and solvent were added. Enzyme model **II** was subjected to a 100 ns MD simulation. Snapshot Sn_931.8_ is shown in Figure 1c. It has the substrate near the heme-binding pocket, whereby its alcohol group forms hydrogen bonding interactions with one of the propionate groups of the heme. Above the substrate in the binding pocket are two Lys side chains and one His residue (Lys_59_, His_62_ and Lys_76_) that appear to provide electrostatic and hydrogen bonding interactions to the substrate. The MD simulation, similar to the one reported above on enzyme model **I**, converged rapidly, with a constant RMSD value for the substrate, protein and heme (Figure 1d).

Although, structurally, the P450 enzyme models **I** and **II** show similarities in three-dimensional fold and heme binding, not surprisingly, they do show differences in substrate positioning. Both have a heme active site that is located next to the I-helix, which is the commonly seen substrate-binding position in P450 enzymes [48,49]. The I-helix contains several polar residues (typically Thr and Asp residues) that deliver protons into the active site during the catalytic cycle [50]. Both isozymes have a similarly sized substrate-binding pocket that will be able to accommodate the piperazine (**1**) substrate, although the two protein structures bind the substrate in a different orientation due to differences in polarity and charge distributions within the substrate-binding pocket. Therefore, we decided to create QM cluster models of both enzymatic structures that contain the heme, oxidant and their second-coordination sphere of residues only and study the oxidative ring-closure reaction to form gliotoxin.

### 2.2. CpdI Structure and Electronic Configuration

**Figure 2:**
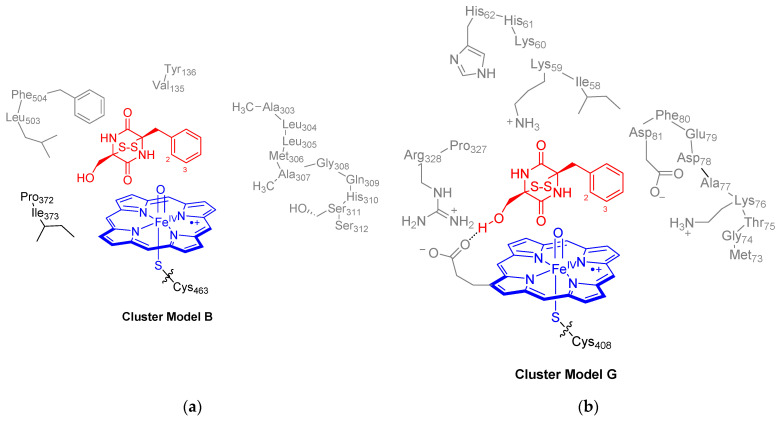
QM cluster models **B** (**a**) and **G** (**b**) investigated in this work. Cluster model **B** labeling based on the 4UYL pdb, while the labeling for model **G** is for P450 GliF. Model **A** contains the atoms highlighted in blue and red only.

Next, we created QM cluster models for the reactant complexes **Re** based on the MD simulations on the full enzyme structures **I** and **II** as described in the previous section (Section 2.1). The cluster models are designated as QM cluster models **A**, **B** and **G**, see Figure 2, and contain the key parts of the protein and substrate that are essential for catalysis. These QM cluster models are typically 200–400 atoms in size and describe the oxidant and substrate and their second-coordination sphere that influences the reaction center through direct electrostatic, steric and hydrogen bonding interactions [51,52]. Previous modeling using QM cluster models showed that these systems can reproduce experimentally determined regioselectivities and give free energies of activation on par with experimental values and hence should be appropriate models for studying inorganic reaction mechanisms and bifurcation pathways [53,54,55]. The QM cluster type, i.e., **A**, **B** or **G**, is given in subscript after the label of each structure, whereas, in superscript, we give the spin state, which is 2 for doublet and 4 for quartet spin. Thus, ^4^**Re**_B_ is the quartet spin state reactant cluster for model **B**. In particular, we selected the snapshot Sn_2000_ of the MD simulation for enzyme model **I** to create the QM cluster models **A** and **B**. Thus, cluster model **A** is a minimal cluster model containing the heme without side chains and substrate only, while, in model **B**, the second coordination sphere was also included as shown in Figure 2. The QM cluster model **B** structure has 281 atoms and has a neutral charge. It includes part of the protein environment, with the protein chains of Val_135_-Tyr_136_, Ala_303_-Leu_304_-Leu_305_-Met_306_-Ala_307_-Gly_308_-Gln_309_-His_310_-Ser_311_-Ser_312_, Pro_372_-Ile_373_ and Leu_503_-Phe_504_. The Leu_304_, Leu_305_ and Gln_309_ amino acids truncated to a Gly residue as these residues point away from the active site and do not appear to be involved in the catalytic mechanism.

The average snapshot (Sn_931.8_) from the MD simulation on enzyme model **II** was selected as a representative structure from the third group of structures obtained from the *k*-clustering approach on P450 GliF (Supporting Information Figure S2) and was used to create cluster model **G**. The QM cluster model **G** included iron–protoporphyrin IX with all side chains (apart from one propionate group) truncated to hydrogen atoms (Figure 1b). The model included the peptide chains Ile_58_-Lys_59_-Lys_60_-His_61_-His_62_, Met_73_-Gly_74_-Thr_75_-Lys_76_-Ala_77_-Asp_78_-Glu_79_-Phe_80_-Asp_81_ and Pro_327_-Arg_328_. In model **G**, the residues Lys_60_, Thr_75_, Ala_77_, Asp_78_ and Glu_79_ were truncated to a Gly residue. Model **G** counted 330 atoms and had a +1 charge.

**Figure 3:**
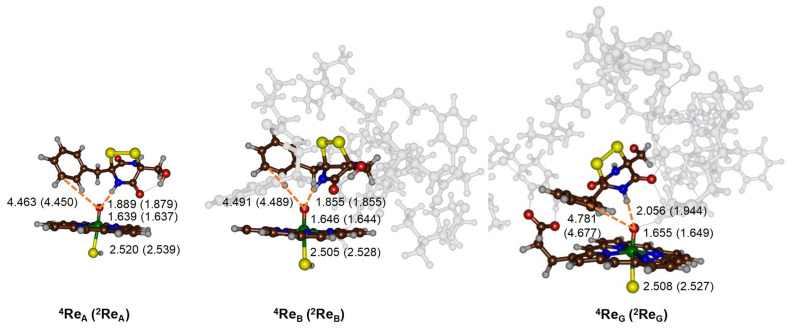
UB3LYP/BS1-optimized geometries of the reactant complexes (Re) for models **A**, **B** and **G** in the quartet and doublet spin states. All distances are in Å.

Full geometry optimization (without constraints) was performed for the reactant clusters (**Re**) for models **A**, **B** and **G** in the doublet and quartet spin states and the obtained local minima are shown in Figure 3. We considered both doublet and quartet spin states for the iron(IV)–oxo heme cation radical species, i.e., Compound I (CpdI), as these states were previously shown to be close in energy [56,57,58,59,60,61,62,63,64,65,66,67,68,69,70,71]. Thus, the doublet and quartet spin states both have an electronic configuration of δ_x2−y2_^2^ π*_xz_^1^ π*_yz_^1^ *a*_1u_^2^ *a*_2u_^1^. The molecular valence orbitals represent metal π and π* molecular orbitals and the heme orbitals *a*_1u_ and *a*_2u_ based on *D*_4h_ symmetry assignments. The *a*_1u_ is pure heme-based and doubly occupied in all structures reported here. The *a*_2u_ for an isolated porphyrin without axial and distal ligands is degenerate with the *a*_1u_ orbital, but a thiolate axial ligand mixes strongly with it and raises it in energy [39,56,67,72]. The 3d-metal orbitals mix with orbitals of first-coordination sphere atoms, apart from the δ_x2−y2_, which is a nonbonding orbital in the plane of the heme and low in energy and doubly occupied. The π*_xz_ and π*_yz_ orbitals represent the antibonding interactions of the atomic iron 3d_xz_/3d_yz_ with a 2p orbital on the oxo group. Higher lying and virtual are the σ*_z2_ and σ*_xy_ molecular orbitals for the antibonding interactions, with the metal 3d orbitals along the S–Fe–O axis (the z-axis) and in the xy-plane with nitrogen atoms of the heme. Nevertheless, all CpdI structures converge to the same electronic state, with unpaired electrons in π*_xz_, π*_yz_ and *a*_2u_ molecular orbitals. Interestingly, despite the major differences in the structural models, geometrically, all reactant structures are very similar. Thus, the iron–oxo distance ranges from 1.637 Å in ^2^**Re**_A_ to 1.655 Å in ^4^**Re**_G_. In all models, the doublet and quartet Fe–O distances are within 0.06 Å. These distances reflect a double bond for the Fe=O interaction as is expected from the orbital occupations. The calculated distances match previous calculations on P450 cluster models and QM/MM-optimized geometries [56,57,58,59,60,61,62,63,64,65,66,67,68,69,70,71,72]. The Fe–S bond is long in all structures due to the interaction of two second-row elements. The ^4^**Re**_B_ and ^4^**Re**_G_ Fe–S distances are almost identical at 2.505/2.508 Å and so are the ^2^**Re**_B_ and ^2^**Re**_G_ Fe–S distances: 2.528 and 2.527 Å. Due to second-coordination sphere effects and particularly protein interactions with the substrate, the positioning of the substrate is different in all structures. In both model **A** and **B**, the N–H group of the substrate forms a hydrogen bond with CpdI at a distance of 1.855–1.889 Å. The hydrogen bond is elongated to 2.056 Å in ^4^**Re**_G_ and 1.944 Å in ^2^**Re**_G_. Nevertheless, in all reactant structures, a hydrogen bond between the N–H group of the substrate and CpdI is seen and the system seems to be set up for hydrogen atom abstraction from that position. By contrast, the position of the aromatic ring is very different in all models, with the C_3_-position at a distance of 4.463/4.450 Å in ^4^**Re**_A_/^2^**Re**_A_. The distance is similar in ^4^**Re**_B_ and ^2^**Re**_B_ but the aromatic ring is perpendicular to the heme, while it is parallel to the heme in ^4^**Re**_G_ and ^2^**Re**_G_ but at a further distance of >4.6 Å. The large distance between the phenyl ring and the oxo group of CpdI may imply that an attack on the aromatic ring is difficult and high in energy; however, we calculated its reaction mechanism nevertheless. The optimized geometries were compared to the MD snapshot structures as well as to crystal structure coordinates of analogous P450 isozymes and, in general, the structures match well and show little deviation in protein fold and amino acid positions with respect to the starting structures.

### 2.3. Oxidative Ring-Closure Mechanism of 1 by CpdI

**Scheme 2:**
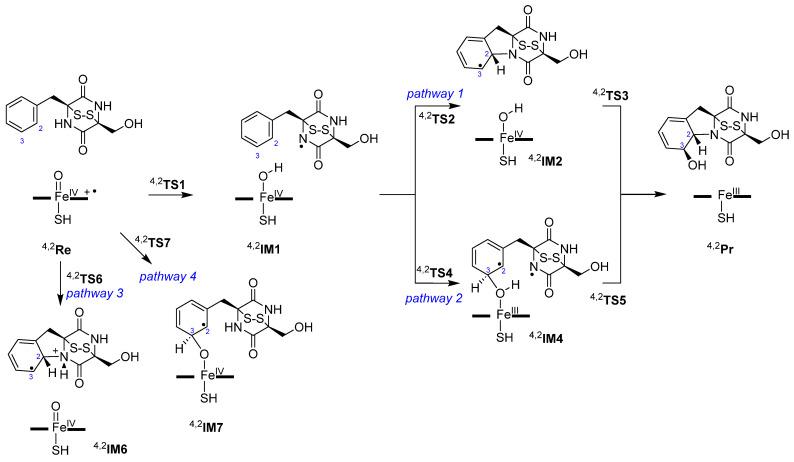
Reaction mechanism for oxidative ring-closure to form gliotoxin products in a reaction of **1** with P450GliF CpdI. The scheme gives the nomenclature of the transition states and local minima and the various reaction mechanisms and reaction channels that were considered.

Next, we investigated the oxidative ring-closure reaction mechanism of **1** by CpdI to form gliotoxin as shown in Scheme 2. We started from the reactant complexes (**Re**) of CpdI complexed with substrate **1** and explored four pathways, designated as pathways 1, 2, 3 and 4. These pathways initiate the reaction with either a hydrogen atom abstraction from the N–H bond (pathways 1 and 2), a ring-closure with simultaneous electron transfer (pathway 3) or an electrophilic addition to the phenyl group (pathway 4). In pathway 1 and 2, the reaction starts with a hydrogen atom abstraction transition state (**TS1**) to form an iron(IV)–hydroxo heme with a nearby substrate radical (intermediate **IM1**). From the radical intermediate, a bifurcation pathway was explored, namely either continuing with a ring-closure transition state **TS2** to form intermediate **IM2** (pathway 1) or, alternatively, with an initial OH rebound to the C_3_-position of the phenyl ring via transition state **TS4** to form structure **IM4** (pathway 2). Finally, from **IM2**, the OH rebound via transition state **TS3** gives gliotoxin products (**Pr**), and these products are also formed from **IM4** after ring-closure via transition state **TS5**.

**Figure 4:**
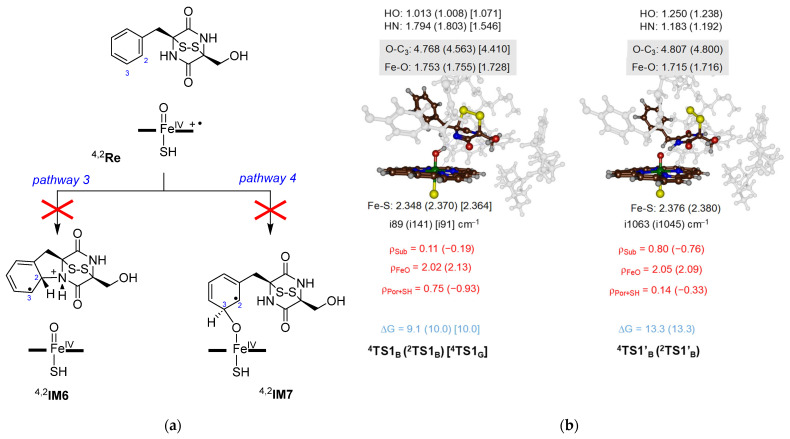
Activation of **1** by P450 CpdI via pathways 1, 2, 3 or 4. (**a**) Cluster model **B** results for pathway 3 and 4. (**b**) Cluster model **B**- and **G**-calculated transition state structures for proton transfer (**TS1**) and hydrogen atom abstraction (**TS1′**) from **1** by P450 CpdI. Data presented are UB3LYP/BS2//UB3LYP/BS1-calculated free energies (in kcal mol^−1^) at 298 K. Optimized **TS1** and **TS1′** transition state structures are shown in part b with bond lengths in Å and the imaginary frequency in cm^−1^.

In pathway 3, we attempted to start the reaction with a ring-closure of the substrate via transition state **TS6**. This step will trigger an electron transfer from the substrate to CpdI. However, a geometry scan from ^4^**Re**_B_ for the direct ring-closure mechanism for the shortening of the C_2_–N distance mechanism did not lead to a stable product structure. Instead, the energy continuously increased to values well over 40 kcal mol^−1^ and a stable local minimum was never formed. Therefore, a reaction starting with substrate ring-closure is unfeasible and unlikely to happen and pathway 3 can be discounted. Similarly to pathway 3, pathway 4 for the initial electrophilic reaction mechanism through an attack of CpdI on the C_3_-position of the aromatic ring of **1** did not lead to a low-energy reaction barrier (Figure 4a). This is surprising as P450 CpdI is well known to activate aromatic compounds through hydroxylation [73,74,75]. The geometry scan for C–O bond shortening gave a high-energy pathway for model **B**. Therefore, the electrophilic attack of CpdI on the phenyl ring can be ruled out as a starting point of the reaction. This means that neither aromatic hydroxylation nor aromatic ring epoxidation are viable mechanisms for P450 GliF and pathway 4 is ruled out as a possible mechanism for gliotoxin biosynthesis.

As pathway 3 and 4 for the gliotoxin biosynthesis by CpdI were ruled out as high-energy pathways (Figure 4a), we proceeded with N–H group activation by CpdI via a transition state **TS1** to form a radical intermediate **IM1** as the starting point for pathways 1 and 2. Thus, we characterized two types of **TS1** transition states for model **B**, namely one set representing a proton transfer (**TS1**) and another set corresponding to a hydrogen atom transfer (**TS1′**) based on the charge and spin distributions in the transition states. The optimized geometries of **TS1** and **TS1′** are shown in Figure 4b. The lowest barriers for activation of the N–H group by CpdI have a free energy of activation of ΔG^‡^ = 9.1 kcal mol^−1^ for ^4^**TS1**_B_ and 10.0 kcal mol^−1^ for ^2^**TS1**_B_, whereas the corresponding hydrogen atom transfer barriers were both located at ΔG^‡^ = 13.3 kcal mol^−1^. Previous calculations on aliphatic C–H hydrogen atom abstraction reactions by P450 CpdI models calculated the free energy of activations ranging from 10–20 kcal mol^−1^ depending on the strength of the C–H bond that was broken [49,50,65,69,70,71,76,77,78,79,80,81,82,83,84,85,86,87]. Nevertheless, for the P450 GliF model **B**, the proton transfer has a lower free energy than hydrogen atom transfer from **1** to CpdI and proton transfer will be the dominant mechanism. The group spin densities of the transition states (highlighted in red in Figure 4) show that the three unpaired electrons reside on iron–oxo and heme–thiolate orbitals. These spin densities are not dramatically different from the reactant complexes ^4,2^**Re**_B_ and hence the electronic configuration has not changed after the proton transfer took place. By contrast, in ^4,2^**TS1′**_B_, the spin on the substrate has increased to 0.80 in the quartet spin state and −0.76 in the doublet spin state, while the sum of the spin on the heme and thiolate groups has decreased to 0.14 and −0.33 in the quartet and doublet spin states, respectively. Consequently, the ^4,2^**TS1′**_B_ transition states give an electron transfer from the substrate into the *a*_2u_ orbital and the substrate radical is accumulating at the same time. This is the traditional hydrogen atom abstraction electronic configuration seen for P450 reactions [50].

The free energies of activation via **TS1** and **TS1′** are in line with computational studies on alternative substrates for N–H hydrogen atom abstraction reactions by P450 CpdI models [88,89,90,91,92,93,94,95]. Moreover, the observation that the two spin states are close in energy for the hydrogen atom transfer or proton transfer steps shows that the electron transfer processes are the same for the two spin states as highlighted before [50]. For model **G**, a proton transfer barrier of ΔG^‡^ = 10.0 kcal mol^−1^ is obtained; hence, the different models give qualitatively similar results. On all surfaces, the system relaxes to a radical intermediate (**IM1**), which is ΔG = −8.6 kcal mol^−1^ below reactants on the doublet spin state and ΔG = −9.1 kcal mol^−1^ for the quartet spin state for model **B**.

Optimized geometries of the **TS1** and **TS1′** transition states are shown in Figure 4b. The three **TS1** transition states have a small imaginary frequency of i89–i141 cm^−1^, while the **TS1′** transition states have an imaginary frequency of i1045/i1063 cm^−1^. Previous calculations on aliphatic hydrogen atom abstraction reactions by P450 CpdI model reactions gave transition states with an imaginary frequency of well over i1000 cm^−1^ [50,51,76,77,78,79,80,81,82,83,84,85,86,87]. As such, our **TS1′** transition state structures and frequencies match previous work in the field and characterize them as hydrogen atom transfer barriers. The animations of the imaginary frequencies of **TS1** and **TS1′** identify them as N–H–O stretch vibrations. Most probably the reason for the small imaginary frequencies in **TS1′** is because the structures are late on the potential energy surface, with short O–H and long N–H distances that position them close to the radical intermediates. Previously, hydride transfer was also shown to give small imaginary frequencies in the rate-determining transition state [95].

Geometrically, the Fe–O, Fe–S and O–C_3_ distances are very similar in the five transition state structures in Figure 4. However, the O–H and N–H distances are very different. The O–H distances are short in the proton transfer transition states, ranging from 1.008 to 1.071 Å, while they are much longer in the hydrogen transfer transition states, namely 1.250 Å for ^4^**TS1′**_B_ and 1.238 Å for ^2^**TS1′**_B_. Similar differences are seen for the N–H distance that is long for the **TS1** transition states, ranging from 1.546 Å for ^4^**TS1**_G_ and 1.794/1.803 Å for ^4^**TS1**_B_/^2^**TS1**_B_. In the **TS1′**_B_ structures, the N–H bond is much shorter, at 1.183 and 1.192 Å for ^4^**TS1′**_B_ and ^2^**TS1′**_B_, respectively. Interestingly, upon formation of the radical intermediates ^4,2^**IM1** for models **A** and **B**, one of the C–S bonds breaks and radical character is accumulated on the terminal sulfur atom rather than elsewhere on the substrate. The C–S bond, however, is reformed in the next step of the mechanism.

**Figure 5:**
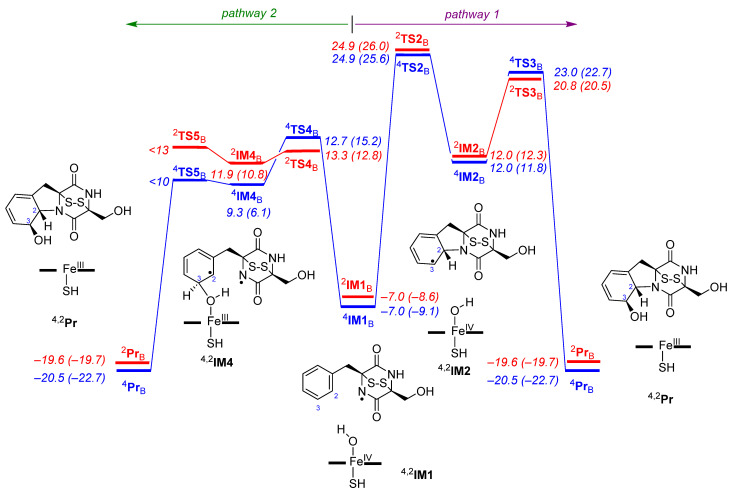
UB3LYP/BS2//UB3LYP/BS1-calculated potential energy profile for gliotoxin biosynthesis via pathway 1 (from middle to the **right**) and pathway 2 (from middle to the **left**). Energies (in kcal mol^−1^) are ΔE + ZPE values, with free energies at 298 K in parenthesis. Doublet spin landscape in red and quartet spin landscape in blue.

The potential energy landscapes for the reactions via pathways 1 and 2 starting from the radical intermediates ^4,2^**IM1**_B_ are shown in Figure 5. The ring-closure step via ^4,2^**TS2**_B_ incurs a significant barrier of ΔG = 25.6/26.0 kcal mol^−1^ on the quartet/doublet spin state surface. The ring-closed intermediate **IM2** that is formed is above the reactants in free energy and picks up the OH group from the iron(III)–hydroxo(heme) via a barrier of ΔG = 22.7/20.5 kcal mol^−1^ on the quartet and doublet spin state to form gliotoxin products with large exothermicity. Overall, the barriers of this mechanism appear relatively high and consequently the reaction will be slow, with a rate-determining ring-closure step. Nevertheless, the energetics via pathway 1 are close to those recently reported by Wang et al. using QM/MM approaches on P450 NascB, where CpdI reacts with a cyclo-(L-tryptophan-L-proline) substrate to undergo a N–H hydrogen atom abstraction followed by C–N bond formation and ring-closure of a piperidine ring that found an endothermic step prior to the ring-closure in the substrate [96]. We also calculated the mechanism for model **G** and found similar energetics and barrier heights. Therefore, the changes in the substrate-binding pocket and the polarity of the substrate environment do not appear to influence the energetics of the reaction much. Note as well that, in the ring-closure transition state, the disulfide bridge is fully back in position.

The alternative mechanism from ^4,2^**IM1**_B_ via pathway 2 is shown in Figure 5 from the middle to the left. This pathway continues with OH rebound to the C_3_-position of the phenyl group, with a free energy of activation of ΔG = 12.8 (doublet) and 15.2 (quartet) kcal mol^−1^. The subsequent ring-closure, however, is negligible, and we were unable to characterize these transition states. In particular, during the geometry optimizations, the structures collapsed to the product complexes. Moreover, a constraint geometry scan showed these pathways to have small barriers of less than 1 kcal mol^−1^. Overall, therefore, pathway 2 will be the dominant mechanism that proceeds with an initial proton transfer from the N–H group of the substrate to CpdI. After the transition state, a fast electron transfer takes place to form the radical intermediates **IM1**, and then the transfer of the OH group to the C_3_-position of the substrate. Finally, the ring-closure step then forms the gliotoxin products.

**Figure 6:**
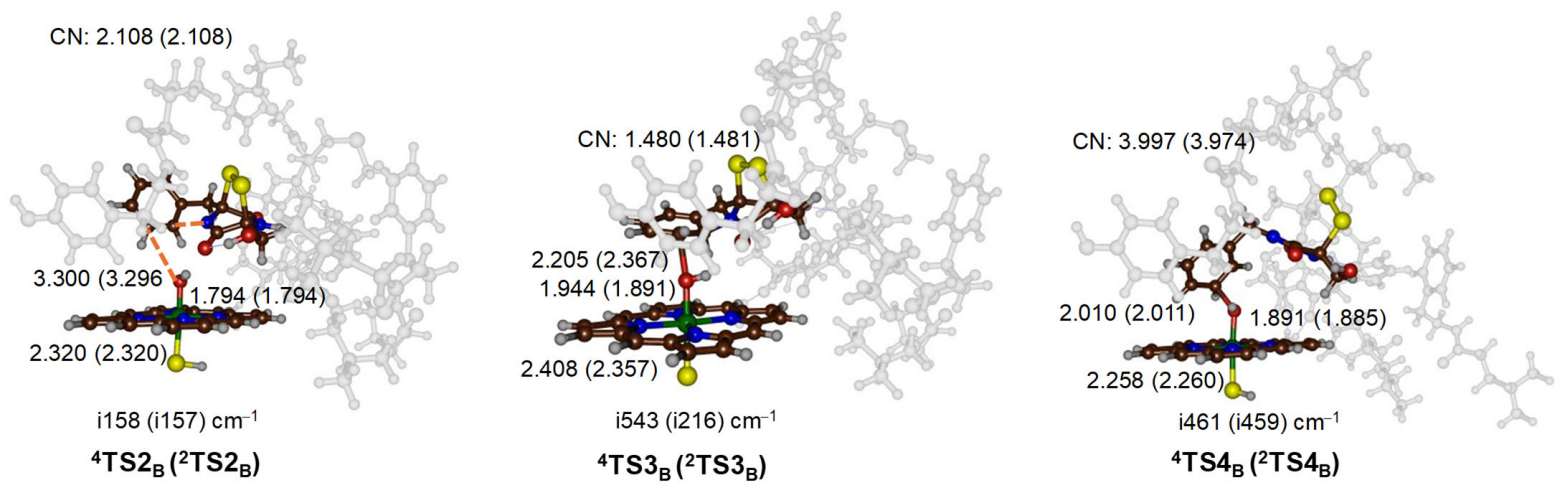
UB3LYP/BS1-optimized transition state structures for geometries alongside pathways 1 and 2. Bond lengths are in Å and the imaginary frequency is in cm^−1^.

Optimized transition state structures for the pathway 1 and 2 mechanisms for CpdI with **1** are shown in Figure 6. The ring-closure transition states ^4^**TS2**_B_/^2^**TS2**_B_ have an imaginary frequency of i158/i157 cm^−1^ for the C–N stretch vibration in the substrate. Their C–N distances are still long, namely 2.108 Å for both transition states. As a matter of fact, both transition states have a similar structure and energetics due to the same electronic configuration on both surfaces. The **TS3**-optimized geometries are very similar in structure for model **A** and **B**; therefore, the second coordination sphere appears to have little influence on this barrier. These transition states are located at relatively long C–O distances of 2.205 Å in ^4^**TS3**_B_ and 2.367 Å in ^2^**TS3**_B_, while the Fe–O distance has elongated significantly to values of 1.944 and 1.891 Å. In previous calculations of aromatic hydroxylation by P450 CpdI, the C–O distances were generally below 2.1 Å [97,98,99,100,101,102,103], and hence the OH transfer barriers happen earlier and the interaction between iron–hydroxo and the substrate is large. The **TS3** transition states give an imaginary mode for the C–O stretch vibration, with a magnitude of i216–i543 cm^−1^. These values are of similar magnitude to aromatic hydroxylation transition states reported for alternative systems before [97,98,99,100,101,102,103].

The transition states for pathway 2 are also shown in Figure 6. The ^4,2^**TS4**_B_ structures represent an OH transfer transition state and give a C–OH stretch vibration with an imaginary mode of i461/i459 cm^−1^. The C–O distance formed is 2.010 Å in ^4^**TS4**_B_ and 2.011 Å in ^2^**TS4**_B_, which are typical distances for OH rebound transition states [104,105]. In both structures, the C–S bond is broken and a radical is present on the terminal disulfide group. We tested whether C–S reformation from ^4^**IM4**_B_ would lead to products as well. This scan had a maximum of about 10 kcal mol^−1^ above ^4^**IM4**_B_ (Supporting Information, Figure S18) and closed the C–N ring to form gliotoxin products as well.

### 2.4. Oxidative Ring-Closure Mechanism 1^−^ by CpdI

It has been suggested that, in some cases, the substrate can donate a proton in the catalytic cycle and reacts with CpdI in its deprotonated form [19]. We therefore tested the reaction of CpdI with the deprotonated substrate **1^−^** for model **B**, designated model **B**_m_, which has the same residues included in the cluster model but misses the proton on the substrate; the model therefore is singly negatively charged. In the quartet spin state, the ^4^**Re**_Bm_ structure has a Fe–O distance of 1.630 Å and a Fe–S distance of 2.566 Å. These distances are in good agreement with those found for ^4^**Re**_B_ and ^4^**Re**_G_ discussed above and show that the structure changes little after substrate deprotonation.

Subsequently, we investigated potential reaction pathways of the activation of the deprotonated substrate by CpdI. For cluster model **B**_m_, the same pathways as discussed in Scheme 1 above were explored. However, pathways 1 and 2 are not possible for model **B**_m_ due to the missing proton, but, for pathways 3 and 4, constraint geometry scans were performed. The direct ring-closure of the deprotonated substrate is highly endothermic and does not lead to a stable intermediate. Therefore, a direct ring-closure of the deprotonated substrate is not possible. We then explored the aromatic hydroxylation channel for model **B**_m_ by initially running a C–O bond shortening scan. A transition state was located and geometry-optimized and found to be high in energy, namely ΔG^‡^ = 33.8 kcal mol^−1^ above the free energy of ^4^**Re**_Bm_. The transition state gives an imaginary frequency of i558 cm^−1^ for C–O bond formation at a C–O distance of 1.797 Å. Consequently, the deprotonated substrate will be inactive with P450 CpdI and no low-energy transition state between **1^−^** and CpdI is found that would lead to gliotoxin products.

## 3. Discussion

In this work, a computational study is presented on the oxidative ring-closure and hydroxylation of **1** by P450 enzymes to form gliotoxin products. We tested the reaction of CpdI with **1** and deprotonated **1**, i.e., **1^−^**, and found that the deprotonated substrate cannot react with CpdI either through direct ring-closure or through electrophilic addition reactions. Therefore, if the substrate donates a proton in the catalytic cycle of P450 that leads to the formation of CpdI, it will need to be reprotonated again with a proton from the solvent before substrate activation can take place. Nevertheless, the reaction between CpdI and **1** gives a low-energy pathway and will proceed fast.

For the reaction of CpdI with **1**, many pathways were investigated as summarized in Scheme 2 above, whereby an initial ring-closure and epoxidation and electrophilic addition steps were ruled out and do not give a stable local minimum. However, the lowest-energy pathway starts with proton transfer from **1** to CpdI via a transition state **TS1**. The calculated mechanism bears some similarity with the oxidative ring-closure observed and computationally investigated for the dipeptide *N*-methylvalyl-tryptophanol activation by P450 TleB and the chuangxinmycin biosynthesis by P450 CxnD that both start with an initial hydrogen atom abstraction from an indole N–H group [106,107]. However, in contrast to P450 GliF in P450 TleB and P450 CmnD, no aromatic ring activation happens, but a second hydrogen transfer is followed by a rate-determining ring-closure step.

**Figure 7:**
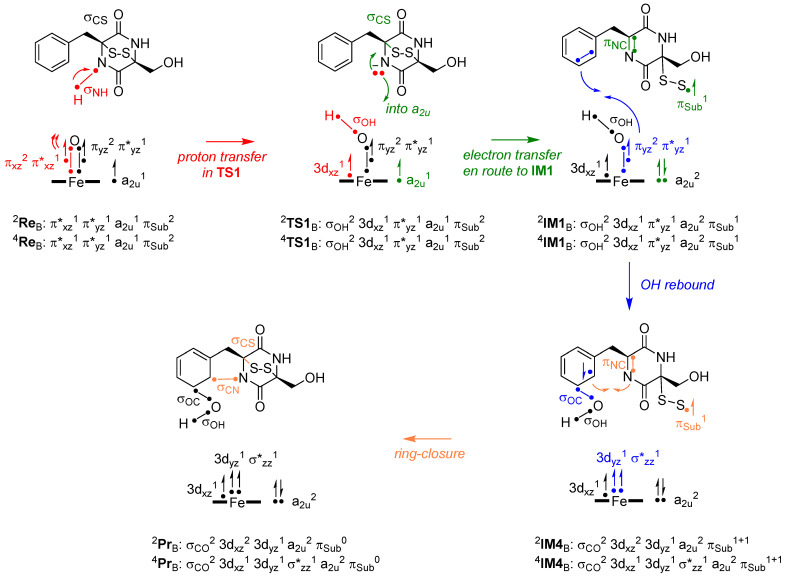
DFT-predicted reaction mechanism for the gliotoxin biosynthesis from **1** by CpdI in P450 enzymes. Valence orbitals are highlighted. A dot represents an electron and a line separating two dots represents a bond occupied by two electrons. Electron transfer pathways are highlighted with an arrow.

To understand and explain the electron transfer mechanisms for gliotoxin biosynthesis and gain insight into the electronic changes during the reaction mechanism, we devised a valence bond (VB) scheme for each step in the reaction mechanism as shown in Figure 7. These VB diagrams have been used previously to explain bifurcation patterns and unusual reaction pathways of metal-containing enzymes [108,109]. Thus, the reaction starts from CpdI and **1** with a transition state for proton transfer, whereby we highlight the relevant electrons and molecular valence orbitals that play a role in this step in red in Figure 7. Chemically, the proton transfer leads to cleavage of the N–H bond (σ_NH_ orbital) in the substrate, whereby the two electrons remain with the nitrogen atom and the proton forms a bond with the oxo group, i.e., the σ_OH_ orbital is formed. However, the σ_OH_ bond formation is coupled with the weakening of the Fe–O as the σ_OH_ orbital is built up from two electrons that were originally part of the π_xz_/π*_xz_ set of orbitals, which are the orbitals for the two-center three-electron bond along the Fe–O axis. The third electron from the π_xz_/π*_yz_ orbital remains as an atomic 3d_xz_ orbital on iron and is singly occupied. Consequently, the proton transfer from **1** to CpdI leads to the breaking of the σ_NH_ orbital in the substrate, the breaking of the π_xz_/π*_xz_ orbitals along the Fe–O bond and the formation of the σ_OH_ bond in the iron(IV)–hydroxo complex. The group spin densities in the proton transfer transition states (**TS1**) confirm this mechanism and show that very little radical characters on the substrate are seen (less than 0.2 units), while the FeO group has a spin of around 2 and the heme scaffold has a spin close to ±1. The spin on the FeO group polarizes mostly on iron and leads to elongation of the Fe–O interaction.

En route from the transition state to the next local minimum, i.e., **IM1**, an electron transfer takes place from the substrate into the *a*_2u_ orbital. This leads to structural and molecular orbital changes in the substrate, whereby the nitrogen atom donates its lone pair into a bond with the neighboring carbon atom and forms a N=C double bond through forming the π_NC_ molecular orbital. At the same time, the adjacent C–S bond breaks and a perthiyl group is formed that bears a radical. These changes in the electronic configurations of the substrate–oxidant complex are shown in green in Figure 7. As such, from **TS1** to the radical intermediate **IM1** leads to a reduction of the iron(IV)–hydroxo heme cation radical to a Compound II type configuration, namely an iron(IV)–hydroxo with a closed-shell heme. Within the substrate, the C–S bond is cleaved homolytically and one of its electrons pairs up with an electron on nitrogen to form the π_NC_ orbital. As the calculations in Figure 4b show, the two steps in the VB diagram in red (in **TS1**) and green (after **TS1**) can also happen in a single transition state via **TS1′** as a concerted hydrogen atom transfer transition state. However, the concerted transition state **TS1′** has a slightly higher free energy of activation of 4.2 kcal mol^−1^ over the proton transfer barrier **TS1** on the quartet spin state and hence the proton transfer will be dominant. This is unusual as typically the P450s react through hydrogen atom transfer with substrate. However, as the N–H bond is weak, the proton and electron transfer are non-synchronous and are identified as separate mechanistic steps. Nevertheless, both transition states relax to form the same radical intermediate **IM1** with an unpaired electron on the perthiyl group. The electronic configuration of ^4,2^**IM1** matches the group spin densities of these species, with one unpaired electron on the sulfur atom of the substrate and two unpaired electrons on the FeO group, while the heme has little unpaired spin density left.

In the next stage of the reaction, an OH rebound from the iron(IV)–hydroxo intermediate to the aromatic ring of the substrate takes place as highlighted in blue in Figure 7. Thus, the aromatic π-system of the phenyl ring breaks open and the π_C2–C3_ orbital returns to atomic orbitals. Thereafter, one electron of the C_2_–C_3_ bond forms a bond with an electron on the oxygen atom to give the σ_OC_ orbital for the C–O bond. This bond formation leaves a radical on the C_2_ position of the substrate. The OH transfer also leads to the breaking of the π_yz_/π*_yz_ two-center three-electron bond that reverts it back into atomic orbitals. Two of its electrons together with the 3d_xz_ electron give the metal the iron(III) oxidation state and either 3d_xz_^1^ 3d_yz_^1^ σ*_z2_^1^ in the quartet spin state or 3d_xz_^2^ 3d_yz_^1^ in the doublet spin state. The final stage of the reaction corresponds to the ring-closure step to form gliotoxin products. At this stage, the metal orbitals do not change anymore. The perthiyl radical attacks the C=N bond and reforms the σ_CS_ orbital and C–S bond. This creates a biradical with a down-spin electron on N adjacent to an up-spin radical on the C_2_ carbon, which then form the σ_CN_ orbital.

The calculated reaction mechanism for the final steps in the gliotoxin biosynthesis by P450 CpdI starts with proton transfer from substrate **1** to CpdI followed by fast electron transfer to give a radical intermediate with a perthiyl group with a free energy of activation of ΔG^‡^ = 9.1 (10.0) kcal mol^−1^ in the quartet (doublet) spin state. From the radical intermediate **IM1**, however, a significant barrier of ΔG^‡^ = 24.3 (quartet) and 21.4 (doublet) kcal mol^−1^ leads to the aromatic addition of the OH group to the C_3_ carbon. The final stage for ring-closure has a small barrier, which is not surprising as the substrate is in a biradical state in ^4,2^**IM4**_B_ with an up-spin electron on C_2_ and a down-spin electron on the perthiyl sulfur atom. The pairing up of two electrons on C_2_ and nitrogen simultaneous to the C–S bond reformation will cost little energy and lead to a highly stable product complex with large exothermicity. Overall, the rate-determining step in the reaction mechanism is the OH rebound step via ^4,2^**TS4**_B_. This is not surprising as the OH rebound requires the breaking of the phenyl π-system but also reduces the π_yz_/π*_yz_ set of orbitals to atomic orbitals. This OH rebound barrier is similar to the one calculated for the aromatic defluorination barrier by a non-heme iron(IV)–oxo system that happens with phenol hydrogen atom abstraction followed by OH rebound to the phenoxyl radical on the *ortho*- or *para*-position [110,111].

**Figure 8:**
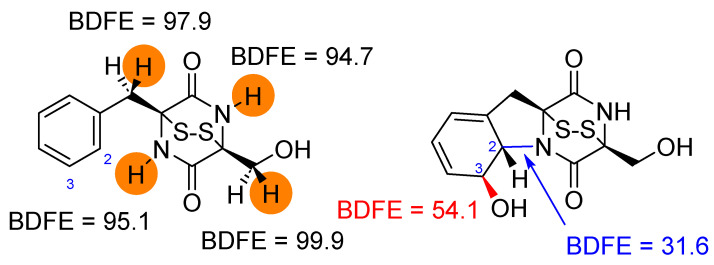
UB3LYP/BS2-calculated bond dissociation free energies at 298 K. Values in kcal mol^−1^ for diabatic hydrogen transfer contain zero-point, thermal and entropic corrections.

Thermochemical analysis on bond breaking and bond forming steps was carried out. To understand the bond formation and bond cleavage steps in the mechanism better, we calculated diabatic C–H/N–H bond dissociation free energies (BDFEs) of key bonds in the substrate as the difference in free energy between the optimized geometry of the substrate, an isolated hydrogen atom and the substrate with one hydrogen atom removed. In our model substrate, both nitrogen atoms of the substrate were taken in the N–H form while, in the enzyme, the second nitrogen atom is methylated. The data are shown in Figure 8, with four hydrogen atoms of the substrate highlighted. Surprisingly, the four N–H/C–H bonds investigated have similar BDFEs that range from ΔG = 94.7 kcal mol^−1^ for the N–H bond on the other side of the substrate to ΔG = 99.9 kcal mol^−1^ for the CH_2_ group of the terminal CH_2_OH group. The N–H group that is being activated in the reaction mechanism has a N–H BDFE of ΔG = 95.1 kcal mol^−1^. Consequently, the two N–H bonds have similar bond strengths and both could be activated by CpdI. Indeed, the enzyme utilizes the *N*-methylated form of **1** instead and thereby prevents activation of the other nitrogen atom of the substrate. This *N*-methylation is an important step prior to the P450 activation as it will guide the reaction to the correct hydrogen atom abstraction site and avoid side-products.

The data in Figure 8 represent diabatic BDFE values whereby the radical structure was not reoptimized. If, instead, we calculate adiabatic BDFEs after hydrogen abstraction from the N–H bond, this leads to immediate C–S cleavage with a radical located on the perthiyl group with an adiabatic BDFE of 69.5 kcal mol^−1^. Our observation, therefore, that the C–S bond is cleaved upon hydrogen atom abstraction corresponds to the creation of a stable perthiyl radical that is energetically favorable over a nitrogen radical. As a consequence, the hydrogen transfer from the N–H group leads to a relatively stable substrate radical with a perthiyl group. Indeed, the structures ^4,2^**IM1** are more stable than the reactant complexes by more than ΔG = 8 kcal mol^−1^. Finally, we calculated the C–OH BDFE and the strength of the C–N bond in the product complex. The weakest of the two bonds is the C–N bond, at ΔG = 31.6 kcal mol^−1^, compared to the C–OH BDFE of ΔG = 54.1 kcal mol^−1^. The low value of the C–OH bond is the result of the breaking of the π-system in the phenyl group, which will cost a considerable amount of energy. The ring-closure will contain steric strain and hence is the weakest bond to form and consequently will be the last step in the mechanism.

## 4. Materials and Methods

### 4.1. Structure Preparation

As there are no crystal structure coordinates available of P450 GliF, we used two starting structures, namely a P450 isozyme that catalyzes an analogous reaction pathway and a homology model generated with AlphaFold. Thus, the 4UYL protein databank file [45,46] represents an analogous protein to P450 GliF and was used as the enzyme model I structure. This is a heme and inhibitor ((*R*)-*N*-(1-(2,4-dichlorophenyl)-2-(1H-imidazol-1-yl)-ethyl)-4-(5-phenyl-1,3,4-oxadiazol-2-yl)benzamide) bound P450 isozyme. We removed the inhibitor and water molecules and used the structure as a P450 GliF model, i.e., enzyme model **I**. The other enzyme structure, i.e., enzyme model **II**, was taken from the recently predicted AlphaFold structure for P450 GliF [47]. We manually inserted the heme in enzyme model **II** and linked it to the sulfur atom of Cys_463_ of the protein and made sure that the propionate groups were in a salt bridge orientation with nearby Arg amino acid residues as seen in similar crystal structure coordinates. The gliotoxin structure was obtained from the T3DB database [112] and manually converted into **1** and geometry optimized at the UB3LYP/6-311 + G* level of theory in Gaussian-09 [113,114,115,116]. The optimized structure was then converted to PDB format and prepared for use in ligand docking into enzyme models **I** and **II**. Thereafter, hydrogen atoms were added to both protein structures under pH 7 conditions in Chimera [117], whereby all carboxylates were chosen as deprotonated and all Lys/Arg amino acid side chains were in their protonated state.

### 4.2. Substrate Docking

Molecular docking was performed using the AutoDock Vina software package version 1.2.5 for enzyme model **I** and **II** [118]. The ligand was docked into the substrate-binding pocket on the distal site of the heme in P450 GliF defined by the coordinates 1.18227, 0.118161 and 5.72849. A box size of 26.8839 × 37.3572 × 24.344 Å^3^ was set. The best binding pose was subsequently selected and a full molecular dynamics (MD) simulation was performed for both enzyme structures.

### 4.3. Molecular Dynamics Simulations

Forcefield parameters for CpdI were determined using the MCPB.py routine as implemented in the Amber software package version 20 [119]. For gliotoxin, the atomic charges were determined using the Antechamber module integrated within AmberTools version 24, employing the AM1 method for molecular geometry optimization [120]. Subsequently, the RESP charges were fitted utilizing the BCC method. A QM geometry optimization in Gaussian at the UB3LYP level of theory and a basis set containing LANL2DZ with effective core potential on iron and 6-31G* on the C, H, O, N and S atoms were performed [113,114,115,116,121]. The ff19SB forcefield was applied for protein residues and the general Amber forcefield (GAFF) for the non-protein systems [122,123,124]. Subsequently, the system was solvated using an OPC water box with a buffer distance of 20.0 Å from the protein edge. Sodium (Na^+^) and chloride (Cl^−^) ions were added to the surface of the structure to obtain a salt concentration of 1 mM and to neutralize the system. The complete enzyme model was minimized by applying a steepest descent algorithm for 20,000 steps followed by 10,000 steps of the conjugate gradient algorithm. Next, the system was gradually heated from 0 to 310 K over 500,000 steps with a time step of 0.002 ps. The Berendsen thermostat was used to control the system temperature during the heating stage. Finally, a production run was performed on the system at a constant temperature of 310 K for 5,000,000 steps. The Langevin thermostat, with a collision frequency of 2 picoseconds, was used to control the temperature. The trajectory data from the production run, containing the coordinates of all atoms as a function of time, were saved at regular intervals for further analysis. This production phase was repeated 200 times, resulting in a total simulation time of 2000 ns. The particle mesh Ewald (PME) method was employed for long-range electrostatics with a direct space sum cut-off of 10 Å. After performing molecular dynamics simulations, the results were analyzed and visualized.

### 4.4. QM Cluster Model Set-Up

Based on the *k*-clustering approach, the MD snapshots were ranked into three separate groups and the most representative snapshot of these clusters were analyzed. This approach was used previously to find an appropriate MD snapshot conformation for the quantum chemical calculations [125,126]. For enzyme model **I**, however, two of these structures had the substrate far away from the heme; hence, a structure from the third set of protein structures was selected for further QM studies. QM cluster models were created as explained in detail previously [50,51,52,127,128,129,130]. We created two QM cluster models based on snapshot 2000 (Sn_2000_) from the MD simulation on enzyme model **I**, namely QM cluster models **A** and **B** (Figure 2a). QM cluster model **A** is a minimal cluster model of 71 atoms of the oxidant and substrate **1** only, while model **B** includes also the second coordination sphere around the substrate. We will focus in the main text on the model **B** results only, while the model **A** data are relegated to the Supporting Information. In general, the two QM cluster models predict the same trends and structural features. Model **B** is a large QM cluster model of 281 atoms that is charge-neutral and contains the heme, substrate, part of the protein and one water molecule, whereby the protoporphyrin IX core had all substituents truncated to hydrogen atoms. The axial Cys_463_ residue was truncated to thiolate and the protein environment included the following active site protein chains: Val_135_-Tyr_136_, Ala_303_-Leu_304_-Leu_305_-Met_306_-Ala_307_-Gly_308_-Gln_309_-His_310_-Ser_311_-Ser_312_, Pro_372_-Ile_373_ and Leu_503_-Phe_504_, with Leu_304_, Leu_305_ and Gln_309_ truncated to a Gly residue by capping the side chain to C–H.

Model **G** was based on the last snapshot from the MD simulation on P450 GliF (enzyme model **II**) and included iron–protoporphyrin IX with all its side chains (apart from one propionate group) truncated to a hydrogen atom (Figure 2b). The model had thiolate as the axial Cys residue and included eight water molecules. In addition, the model contains the peptide chains Ile_58_-Lys_59_-Lys_60_-His_61_-His_62_, Met_73_-Gly_74_-Thr_75_-Lys_76_-Ala_77_-Asp_78_-Glu_79_-Phe_80_-Asp_81_ and Pro_327_-Arg_328_. In model **G**, the residues Lys_60_, Thr_75_, Ala_77_, Asp_78_ and Glu_79_ were truncated to a Gly residue. Model **G** counted 330 atoms and had a +1 charge.

### 4.5. DFT Calculations

The Gaussian-09 software package was employed for the DFT calculations presented in this work [116]. To validate the computational approach, for model **A**, the reactants and first transition state were calculated with several DFT methods, namely B3LYP [107,108], B3LYP-D3 [113,114,131], PBE0 [132] and M06L [133]. These different approaches gave little changes to the structure, spin-state energetics and unpaired spin populations, and hence the work was continued with UB3LYP only; see Supporting Information for details. Basis set BS1 was employed for geometry optimizations, constraint geometry scans, frequency calculations and intrinsic reaction coordinate scans and represents LANL2DZ with core potential on iron and 6-31G* on the rest of the atoms [115,121]. All structures were subjected to full geometry optimization without constraints and were characterized as minima with real frequencies only, while transition states had one imaginary frequency for the correct transition. Single-point calculations were performed with a larger basis set to correct the energies, namely BS2, which contained LACV3P+ (plus core potential) on iron and 6-311 + G* on the rest of the atoms. To verify that transition states connect to the two local minima as specified in the schemes, we ran intrinsic reaction coordinate (IRC) scans for a selection of structures. These IRCs confirmed the reaction pathways described in this work. All calculations were performed for the doublet and quartet spin states. In previous studies of our group, we validated the approaches described here and found an excellent agreement with respect to experimental rate constants and product distributions [134,135,136].

## 5. Conclusions

Cytochrome P450 GliF is a unique P450 isozyme that catalyzes the oxidative ring-closure and ring-hydroxylation steps in the final stages of the gliotoxin biosynthesis reaction. The chemical scaffold of gliotoxin is a common feature in drug molecules, and hence finding a biocatalytic reaction pathway for the biosynthesis of drug molecules is an important development. To understand the details of the reaction mechanism of gliotoxin biosynthesis, we performed a series of computational studies using molecular dynamics and quantum mechanics studies on various P450 GliF structures. Two enzyme structures were created, namely one with AlphaFold and one based on an analogous enzyme system. Both bind a substrate in the active site, although there appear to be differences in substrate orientation and its interactions with the protein. Nevertheless, the two MD simulations implicate that gliotoxin may be synthesized by both enzymes as their active sites are large enough to hold the substrate **1**. To test this, several cluster models of the enzyme active sites that contain the heme, its ligands, substrate and their direct environment were created. The QM cluster models all give the same electronic configuration of a CpdI-type species with three unpaired electrons in π*_xz_ π*_yz_ and *a*_2u_ in either a doublet or quartet overall spin state. Thereafter, pathways for gliotoxin biosynthesis were explored. Firstly, the reaction of deprotonated substrate **1^−^** with CpdI gives high-energy pathways and was ruled out. For the reaction of **1** with CpdI, four pathways were tested, starting with proton transfer, hydrogen atom transfer, electrophilic addition to the aromatic ring and direct ring-closure. The most viable reaction channel was found starting with a proton transfer that, en route to the local minimum, gives a fast electron transfer to give a radical intermediate. Subsequently, the OH group is transferred to the aromatic ring to give a biradical in the substrate that rapidly leads to ring-closure to form gliotoxin products. The mechanistic features are explained by thermodynamic and valence bond approaches and rationalize the rate-determining step in the reaction mechanism.

## Data Availability

All data associated with this work are provided in the Supporting Information associated with this work available upon request from the authors.

## Supplementary Materials

The following supporting information can be downloaded at: https://www.mdpi.com/article/10.3390/ijms25168567/s1.
