# Supplementary material for: Mechanism of the Oxidative Ring-Closure Reaction during Gliotoxin Biosynthesis by Cytochrome P450 GliF
Source: Int J Mol Sci. 2024 Aug 6;25(16):8567. doi: 10.3390/ijms25168567 (PMC11354885; doi:10.3390/ijms25168567)
Supplement: Supplementary file 1 [file ijms-25-08567-s001.zip › ijms-3101509-supplementary.pdf]

Supporting Information

Article

Mechanism of the Oxidative Ring-Closure Reaction During the Gliotoxin Biosynthesis by Cytochrome P450 GliF

Muizz Qureshi [1], Thirakorn Mokkawes [1], Yuanxin Cao [1] and Sam P. de Visser [1,*]

[1] Manchester Institute of Biotechnology and Department of Chemical Engineering, The University of Manchester, 131 Princess Street, Manchester M1 7DN, United Kingdom; sam.devisser@manchester.ac.uk

* Correspondence: sam.devisser@manchester.ac.uk

Part I: MD and *k*-clustering results:

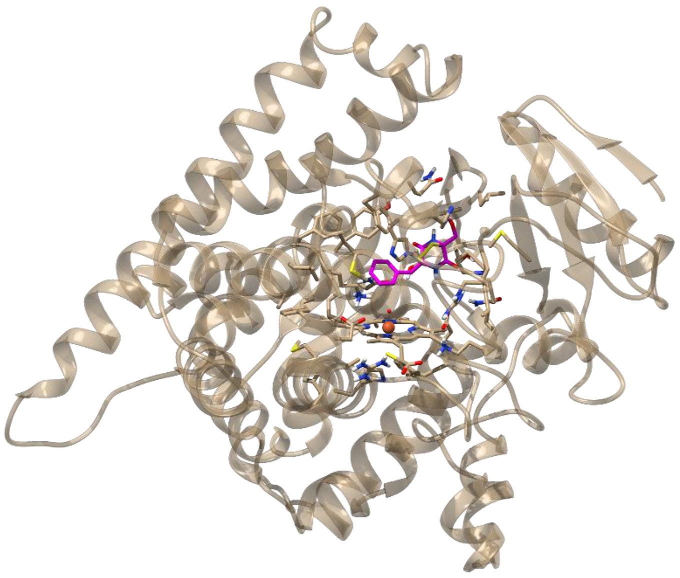

Figure S1. MD simulation snapshot Sn_{2000} of GliF for the MD simulation on protein model **I**. The gliotoxin substrate is shown in magenta.

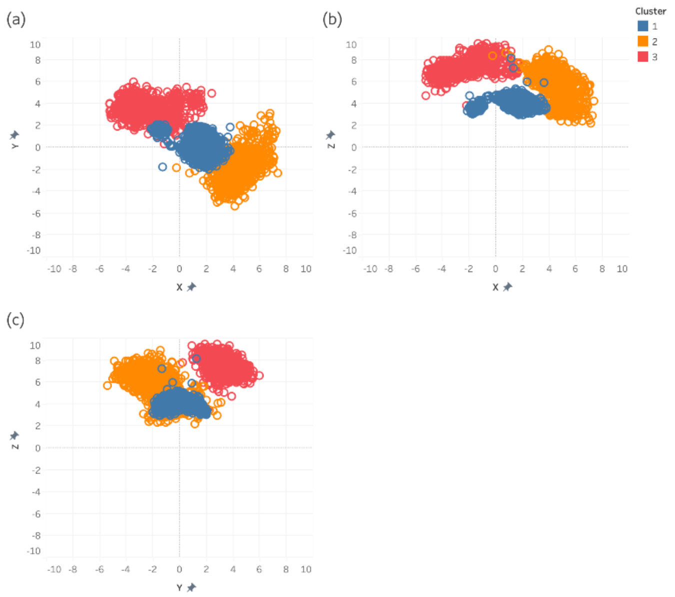

Figure S2. Visualisation through the *k*-means clustering approach with results with respect to the atoms R1, R2, and R3 for the MD data on P450 GliF on protein model **I** for the snapshots taken from the MF simulation on protein model **I**.

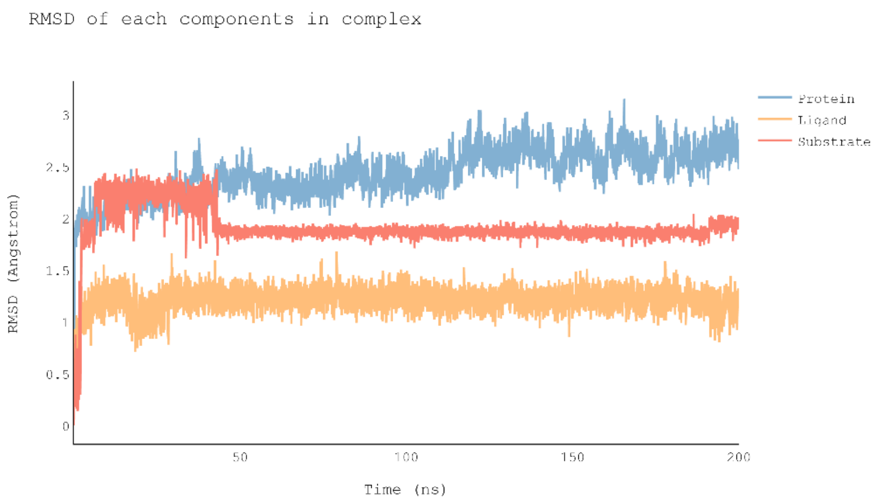

Figure S3. RMSD plot for the protein, ligand and substrate along the MD simulation for the P450 GliF structure **II**.

(a) (b)

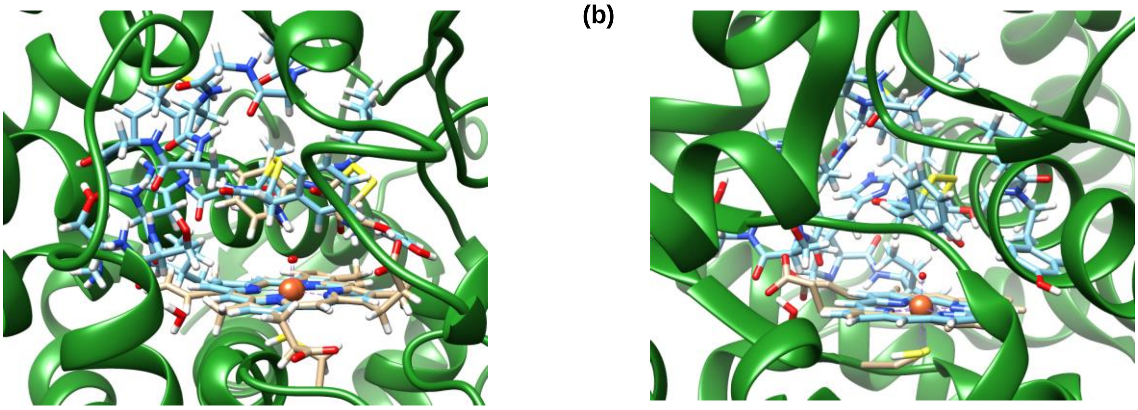

Overlay of MD snapshot and ^{4}Re$_B$

Overlay of 4UYL pdb and ^{4}Re$_B$

Figure S4. (a) Overlay of ^{4}**Re**$_B$ and 4UYL pdb file. (b) Overlay of ^{4}**Re**$_B$ and the last snapshot of the MD simulation for P450 GliF for protein model **II**. The ^{4}**Re**$_B$ structure compares well with the protein chains in both the MD snapshot and the analogous pdb structure.

Part II: DFT Cluster model results:

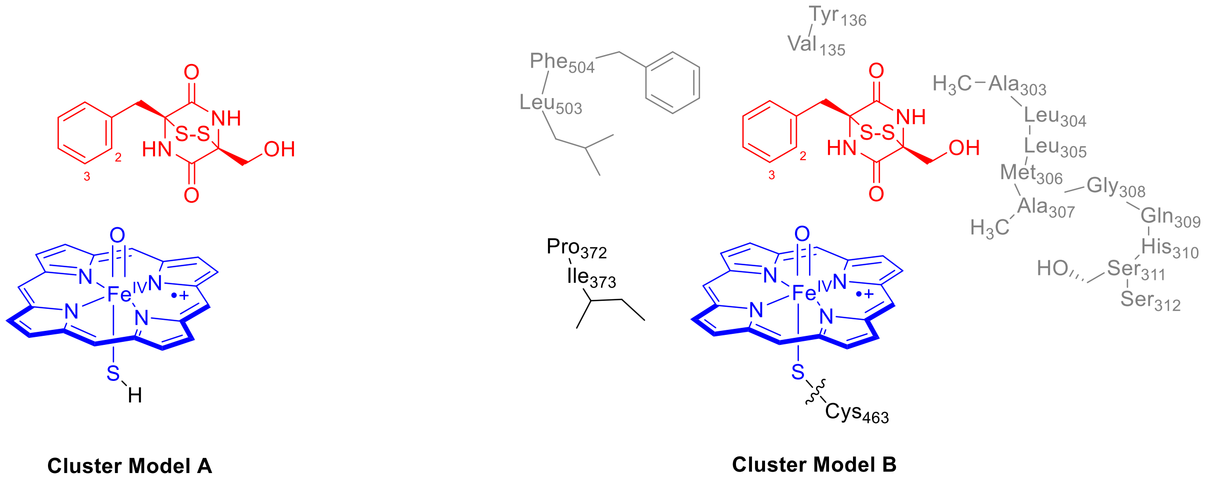

Scheme S1: Cluster models **A** and **B** investigated in this work. Labelling based on 4UYL pdb file.

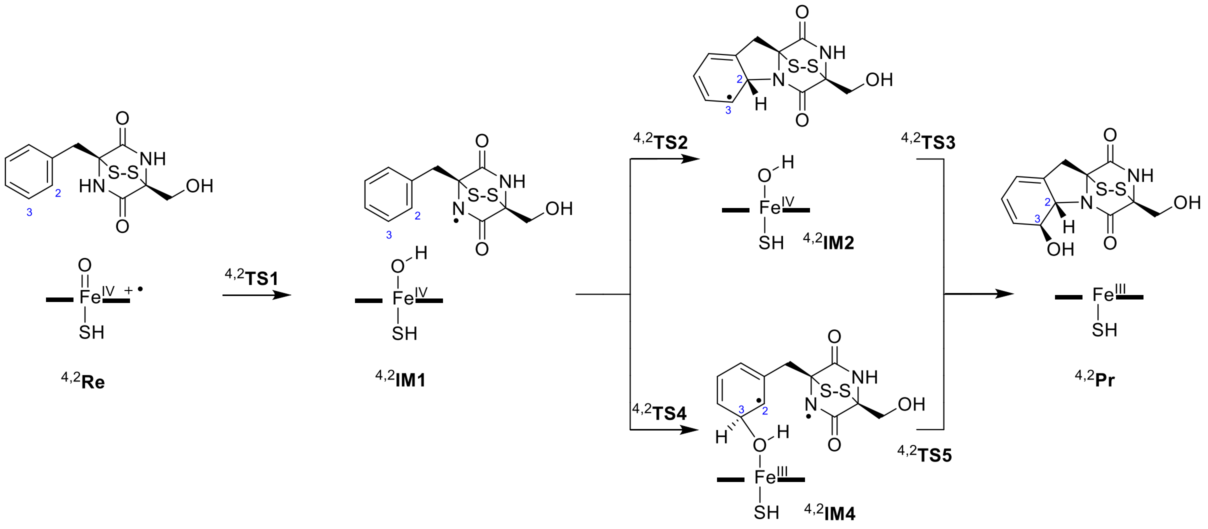

Scheme S2: Reaction scheme explored for gliotoxin biosynthesis for a radical pathway starting with hydrogen atom abstraction. Definitions of the labels of local minima and transition states are given.

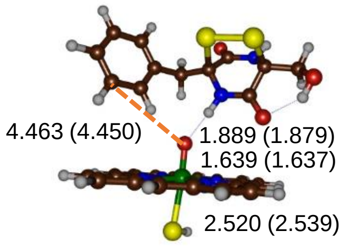

^{4}Re$_A$ (^{2}Re$_A$)

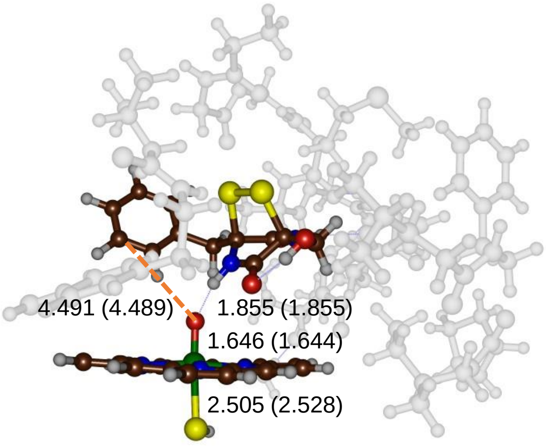

^{4}Re$_B$ (^{2}Re$_B$)

Figure S5: UB3LYP/BS1 optimized geometries of ^{4,2}**Re**$_A$ and ^{4,2}**Re**$_B$ as obtained in Gaussian-09. Bond lengths are in angstroms.

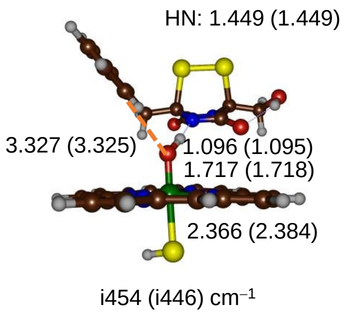

^{4}TS1$_A$ (^{2}TS1$_A$)

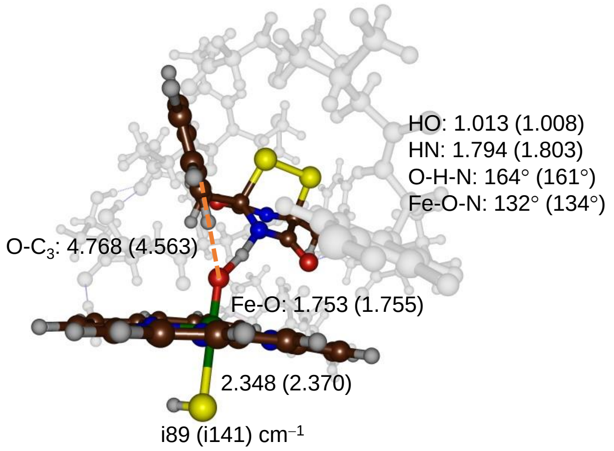

^{4}TS1$_B$ (^{2}TS1$_B$)

Figure S6: UB3LYP/BS1 optimized geometries of ^{4,2}**TS1**$_A$ and ^{4,2}**TS1**$_B$ as obtained in Gaussian-09. Bond lengths are in angstroms and the imaginary frequency in cm^{-1}.

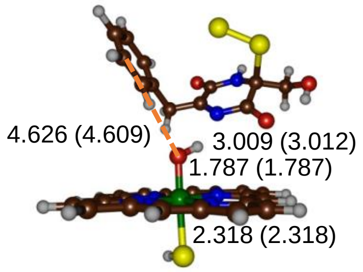

^{4}**IM1$_A$** (^{2}**IM1$_A$**)

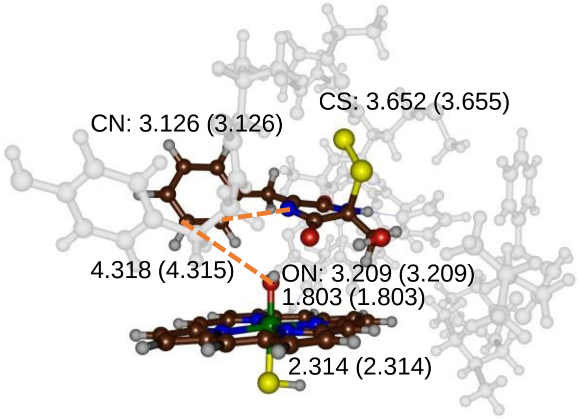

^{4}**IM1$_B$** (^{2}**IM1$_B$**)

Figure S7: UB3LYP/BS1 optimized geometries of ^{4,2}**IM1**$_A$ and ^{4,2}**IM1**$_B$ as obtained in Gaussian-09. Bond lengths are in angstroms.

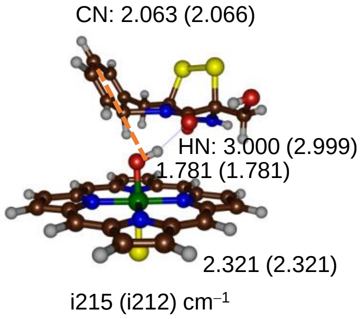

^{4}**TS2$_A$** (^{2}**TS2$_A$**)

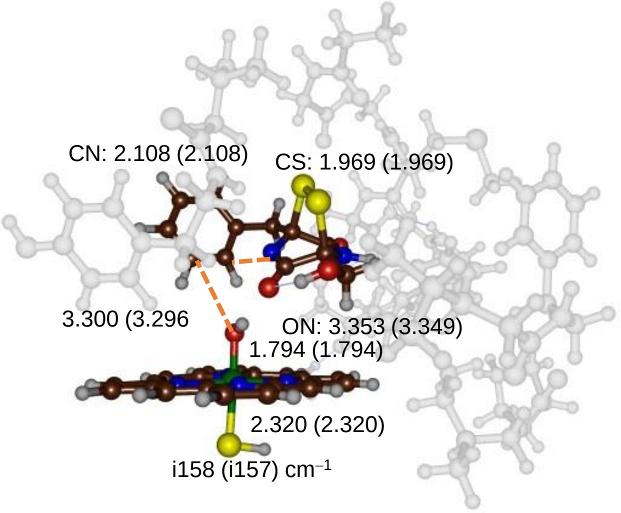

^{4}**TS2$_B$** (^{2}**TS2$_B$**)

Figure S8: UB3LYP/BS1 optimized geometries of ^{4,2}**TS2**$_A$ and ^{4,2}**TS2**$_B$ as obtained in Gaussian-09. Bond lengths are in angstroms and the imaginary frequency in cm^{-1}.

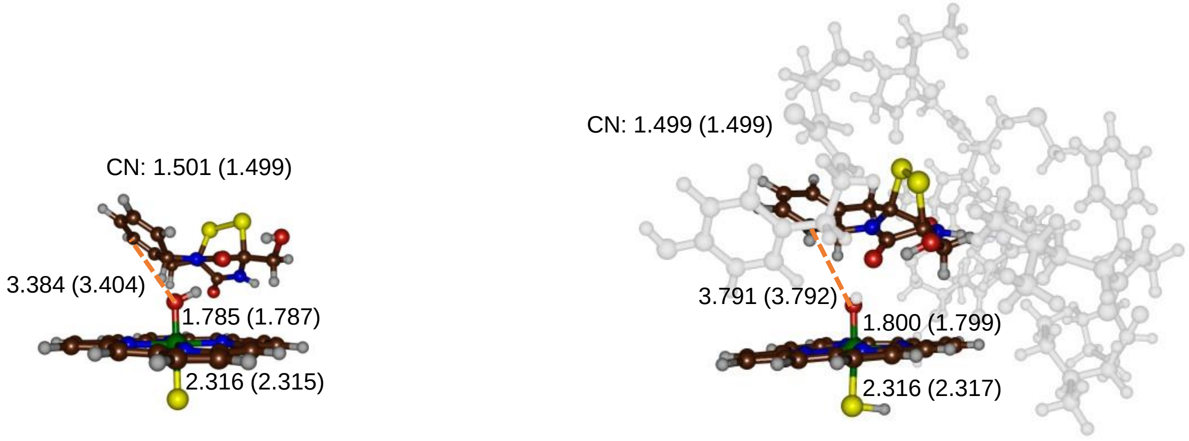

^{4}IM2$_A$ (^{2}IM2$_A$) **^{4}IM2$_B$ (^{2}IM2$_B$)**

Figure S9: UB3LYP/BS1 optimized geometries of ^{4,2}**IM2**$_A$ and ^{4,2}**IM2**$_B$ as obtained in Gaussian-09. Bond lengths are in angstroms.

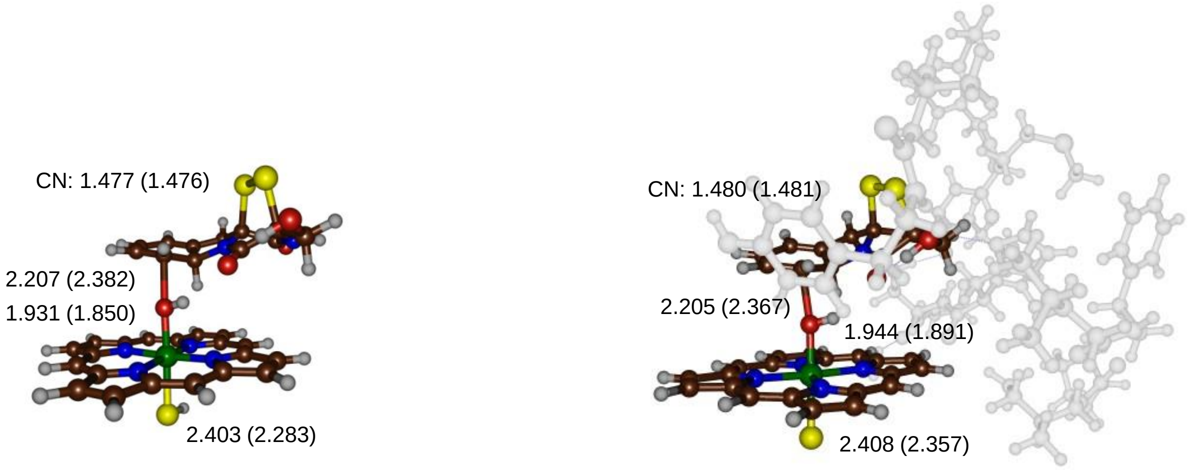

i537 (i405) cm^{-1} i543 (i216) cm^{-1}

^{4}TS3$_A$ (^{2}TS3$_A$) **^{4}TS3$_B$ (^{2}TS3$_B$)**

Figure S10: UB3LYP/BS1 optimized geometries of ^{4,2}**TS3**$_A$ and ^{4,2}**TS3**$_B$ as obtained in Gaussian-09. Bond lengths are in angstroms and the imaginary frequency in cm^{-1}.

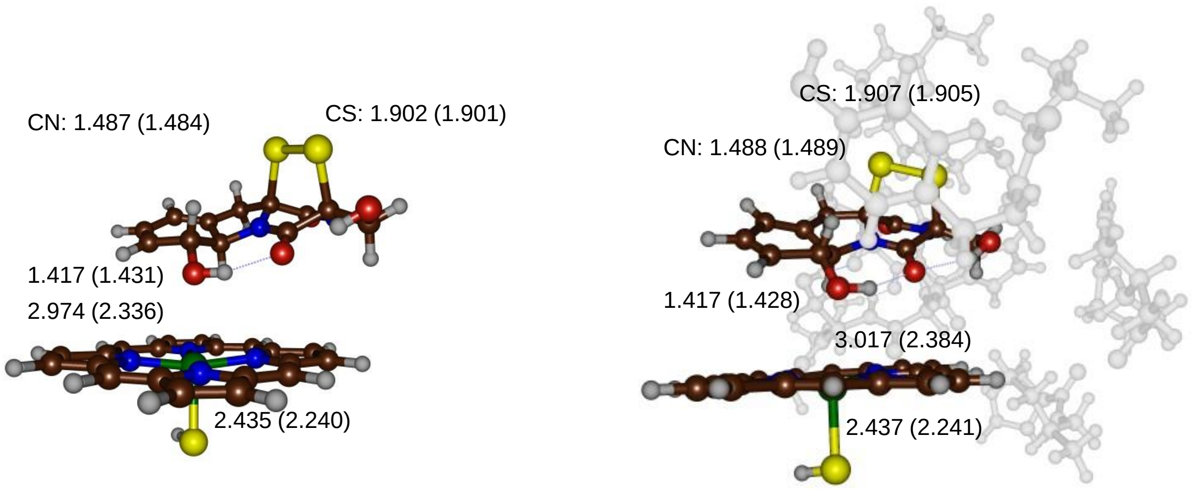

^{4}**Pr**$_A$ (^{2}**Pr**$_A$) ^{4}**Pr**$_B$ (^{2}**Pr**$_B$)

Figure S11: UB3LYP/BS1 optimized geometries of ^{4,2}**Pr**$_A$ and ^{4,2}**Pr**$_B$ as obtained in Gaussian-09. Bond lengths are in angstroms.

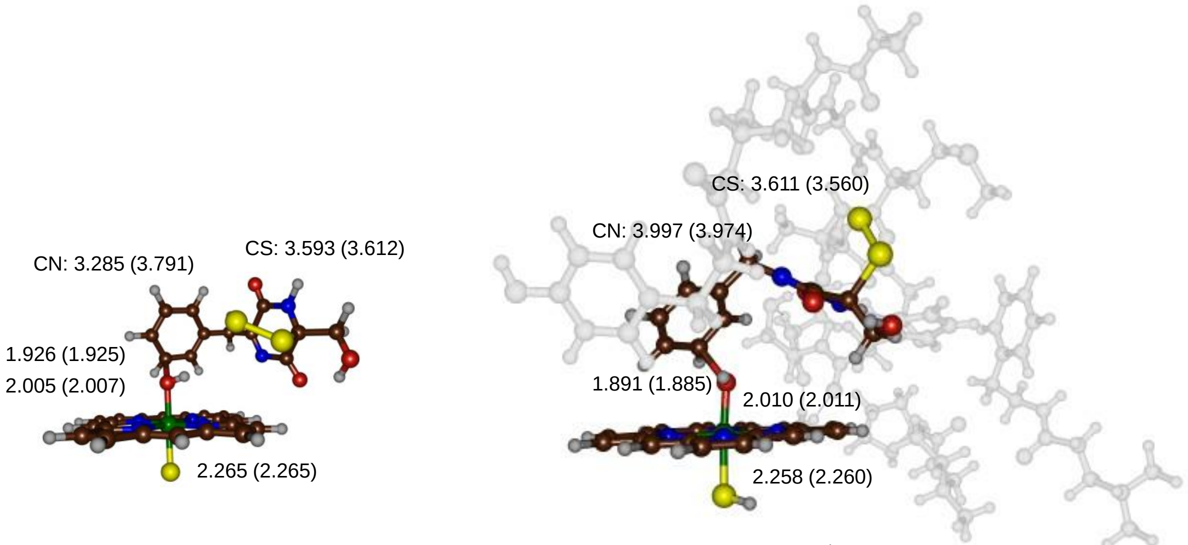

i425 (i426) cm^{-1} i461 (i459) cm^{-1}

^{4}**TS4**$_A$ (^{2}**TS4**$_A$) ^{4}**TS4**$_B$ (^{2}**TS4**$_B$)

Figure S12: UB3LYP/BS1 optimized geometries of ^{4,2}**TS4**$_A$ and ^{4,2}**TS4**$_B$ as obtained in Gaussian-09. Bond lengths are in angstroms and the imaginary frequency in cm^{-1}.

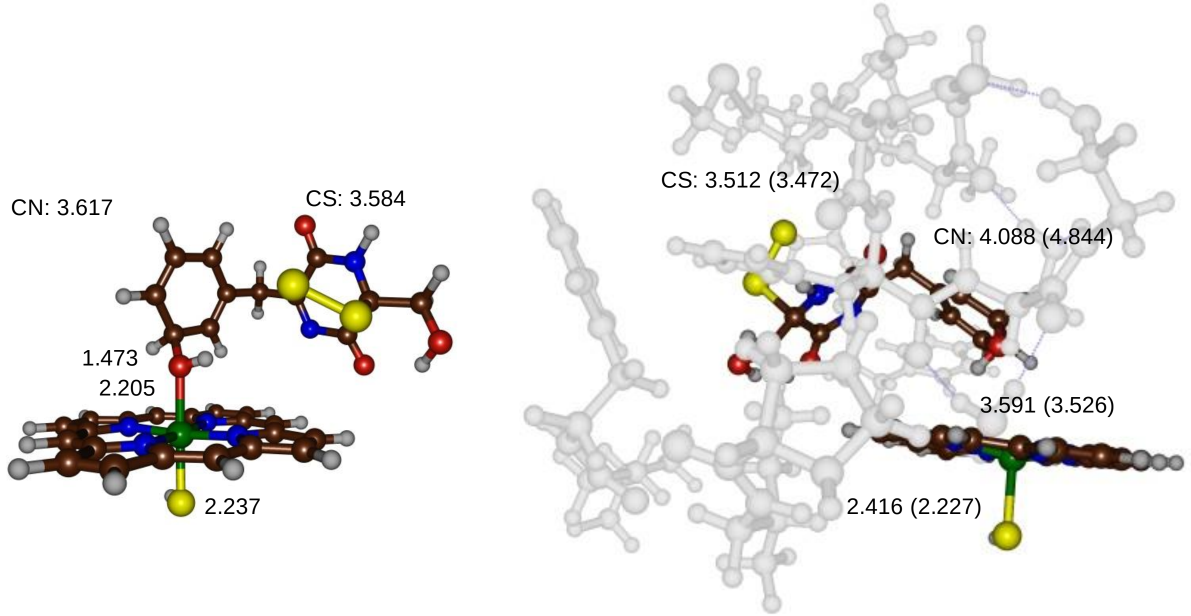

^{4}IM4$_{A}$ (^{2}IM4$_{A}$) **^{4}IM4$_{B}$ (^{2}IM4$_{B}$)**

Figure S13: UB3LYP/BS1 optimized geometries of ^{4,2}**IM4**$_{A}$ and ^{4,2}**IM4**$_{B}$ as obtained in Gaussian-09. Bond lengths are in angstroms.

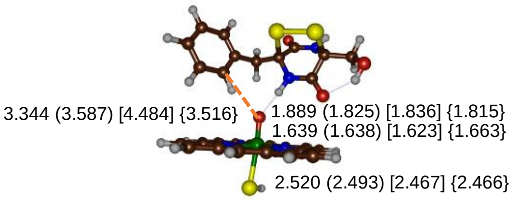

$^{4}\mathbf{Re}_{\mathbf{A,B3LYP}}$ ($^{4}\mathbf{Re}_{\mathbf{A,B3LYP\text{-}D3}}$) [$^{4}\mathbf{Re}_{\mathbf{A,PBE0}}$] {$^{4}\mathbf{Re}_{\mathbf{A,M06L}}$}

Figure S14: Optimized geometries of $^{4}\mathbf{Re}_{A}$ as obtained in Gaussian-09 using different density functional methods, namely B3LYP, B3LYP-D3, PBE0 and M06L. Bond lengths are in angstroms.

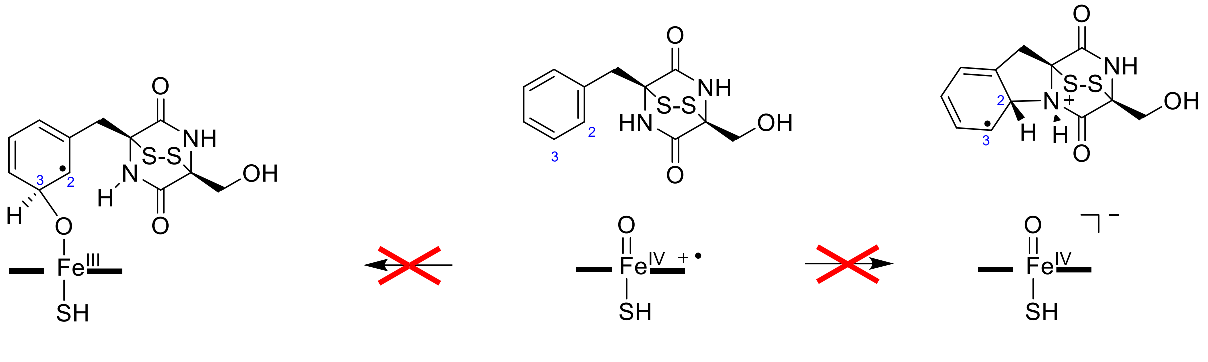

Scheme S3: Alternative mechanisms tested in this work. For details see Figures S17 and S18.

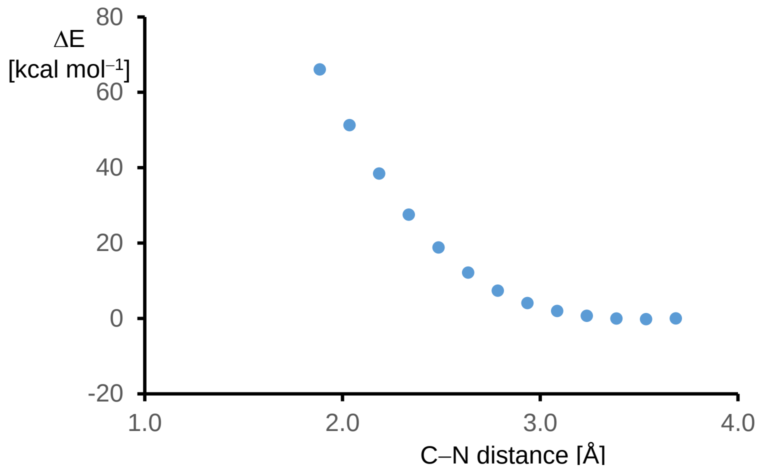

Figure S15: UB3LYP/BS1 calculated geometry scan starting from ^{4}**Re**$_{B}$ for direct ring-closure. As can be seen the scan does not lead to a stable intermediate, hence a pathway starting with ring-closure is not feasible.

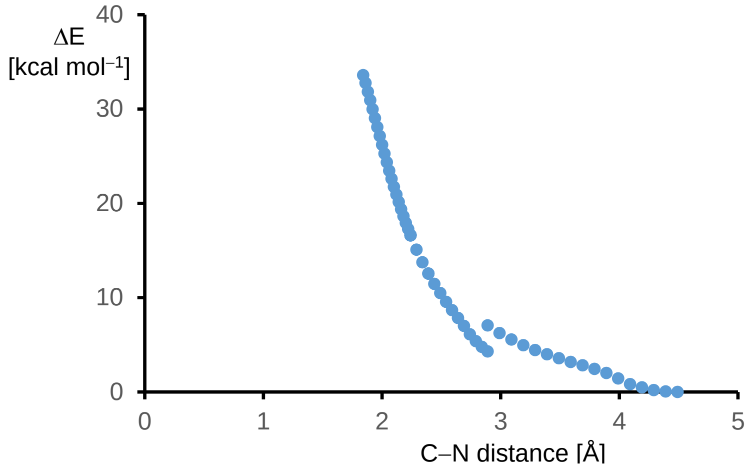

Figure S16: UB3LYP/BS1 calculated geometry scan starting from ^{4}**Re**$_{B}$ for electrophilic attack on C_2. As can be seen the scan does not lead to a stable intermediate, hence a pathway starting with aromatic hydroxylation is not feasible.

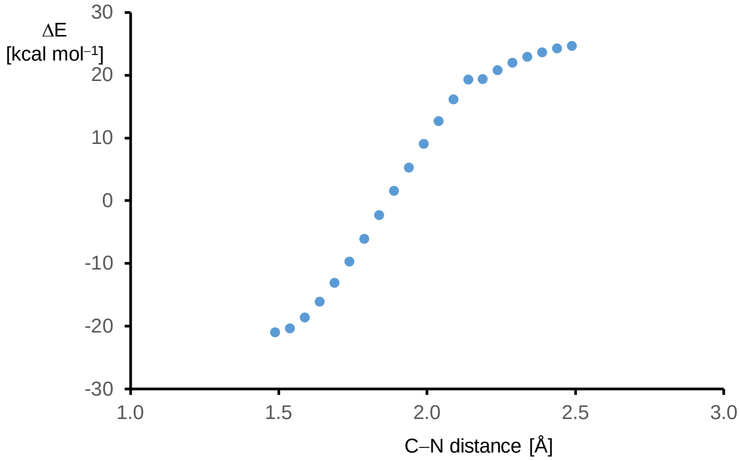

Figure S17: UB3LYP/BS1 calculated geometry scan starting from ^{4}**Prod**$_{B}$ for the ring-opening reaction. As can be seen the scan does not pass a barrier, hence the reverse reaction from ^{4}**IM4**$_{B}$ to ^{4}**Prod**$_{B}$ does not pass a barrier and is highly exothermic leading to products on the left.

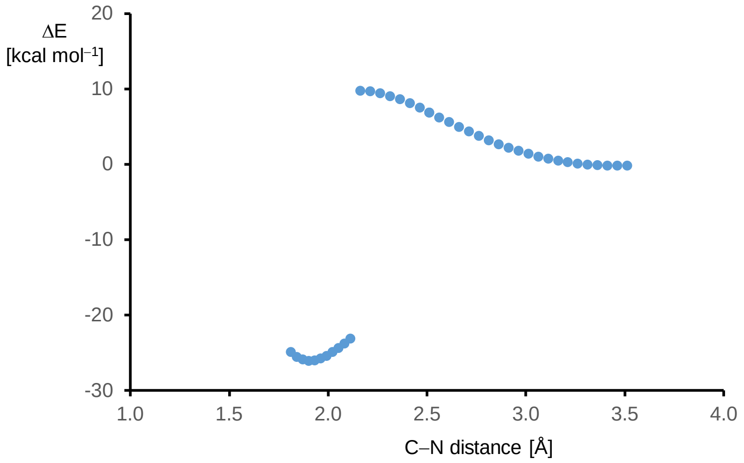

Figure S18: UB3LYP/BS1 calculated geometry scan starting from ^{4}**IM4**$_B$ for the C−S reformation. As can be seen the scan has a barrier of about 10 kcal mol^{-1} to form products on the left.

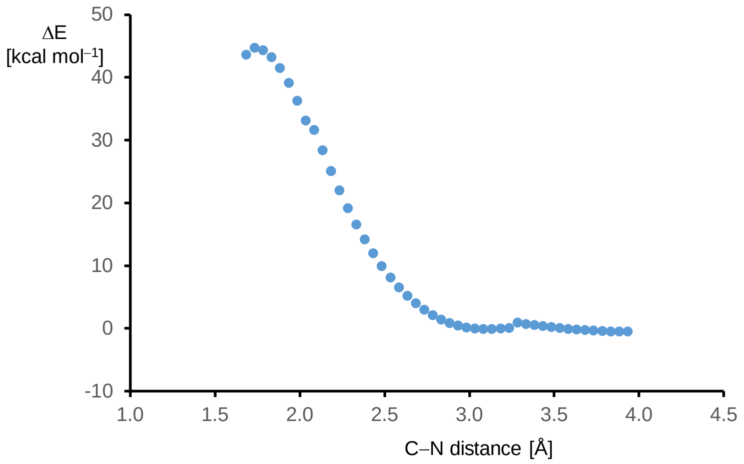

Figure S19: UB3LYP/BS1 calculated geometry scan for the ring-closure reaction for the complex with the deprotonated substrate (**1**$^-$) and CpdI, i.e. ^{4}**Re**$_{Bm}$. As can be seen the reaction is highly endothermic and does not lead to a stable product complex.

Table S1: Absolute (in au) and relative (in kcal mol^{-1}) energies and free energies of UB3LYP/BS1 optimized geometries in Gaussian for gliotoxin activation by a P450 Cpd I model A.

	E [BS1, au]	ZPE [au]	G [au]	ΔE [BS1]	ΔE+ZPE	ΔG
^{4}**Re**$_{A}$	-3181.847850	0.522439	-3181.402233	0.00	0.00	0.00
^{4}**TS1**$_{A}$	-3181.821927	0.515859	-3181.380894	16.27	12.14	13.39
^{4}**IM1**$_{A}$	-3181.853461	0.520559	-3181.413615	-3.52	-4.70	-7.14
^{4}**TS2**$_{A}$	-3181.802906	0.519737	-3181.355574	28.20	26.51	29.28
^{4}**IM2**$_{A}$	-3181.825076	0.520734	-3181.377379	14.29	13.22	15.60
^{4}**TS3**$_{A}$	-3181.805612	0.519958	-3181.359285	26.50	24.95	26.95
^{4}**Prod**$_{A}$	-3181.884631	0.524049	-3181.436498	-23.08	-22.07	-21.50
^{4}**TS4**$_{A}$	-3181.818590	0.519433	-3181.374305	18.36	16.47	17.53
^{4}**IM4**$_{A}$	-3181.833115	0.521120	-3181.387206	9.25	8.42	9.43
^{2}**Re**$_{A}$	-3181.847952	0.522331	-3181.402095	-0.06	-0.13	0.09
^{2}**TS1**$_{A}$	-3181.822199	0.515792	-3181.381027	16.10	11.93	13.31
^{2}**IM1**$_{A}$	-3181.853501	0.520669	-3181.412215	-3.55	-4.66	-6.26
^{2}**TS2**$_{A}$	-3181.802940	0.519768	-3181.354723	28.18	26.51	29.81
^{2}**IM2**$_{A}$	-3181.825383	0.520741	-3181.377004	14.10	13.03	15.83
^{2}**TS3**$_{A}$	-3181.814836	0.520861	-3181.365282	20.72	19.73	23.19
^{2}**Prod**$_{A}$	-3181.892335	0.525736	-3181.436830	-27.91	-25.85	-21.71
^{2}**TS4**$_{A}$	-3181.818430	0.519380	-3181.373625	18.46	16.54	17.95

Table S2: Group spin densities and charges (in au) of UB3LYP/BS1 optimized geometries in Gaussian for gliotoxin activation by a P450 Cpd I model A.

(a) Spin densities.

	Fe	O	Por	SH	Sub	Sum
^{4}**Re**$_{A}$	1.19	0.82	0.51	0.46	0.01	3.00
^{4}**TS1**$_{A}$	1.51	0.49	0.45	0.15	0.39	3.00
^{4}**IM1**$_{A}$	1.76	0.25	-0.12	0.10	1.00	3.00
^{4}**TS2**$_{A}$	1.76	0.27	-0.12	0.09	1.00	3.00
^{4}**IM2**$_{A}$	1.75	0.26	-0.12	0.11	1.00	3.00
^{4}**TS3**$_{A}$	2.28	-0.07	-0.10	0.23	0.67	3.00
^{4}**Prod**$_{A}$	2.50	0.00	0.02	0.47	0.00	3.00
^{2}**Re**$_{A}$	1.33	0.78	-0.59	-0.52	0.00	1.00
^{2}**TS1**$_{A}$	1.66	0.43	-0.66	-0.08	-0.36	1.00
^{2}**IM1**$_{A}$	1.76	0.25	-0.12	0.10	-1.00	1.00
^{2}**TS2**$_{A}$	1.76	0.27	-0.12	0.09	-0.99	1.00
^{2}**IM2**$_{A}$	1.74	0.25	-0.12	0.11	-0.98	1.00
^{2}**TS3**$_{A}$	1.28	0.33	-0.05	0.13	-0.69	1.00
^{2}**Prod**$_{A}$	1.12	0.00	-0.10	-0.03	0.00	1.00

(b) Charges.

	Fe	O	Por	SH	Sub	Sum
^{4}**Re**$_{A}$	0.48	-0.49	0.08	-0.03	-0.04	0.00
^{4}**TS1**$_{A}$	0.43	-0.67	0.14	-0.01	0.11	0.00
^{4}**IM1**$_{A}$	0.40	-0.71	-0.23	0.04	0.50	0.00
^{4}**TS2**$_{A}$	0.41	-0.72	-0.22	0.04	0.50	0.00
^{4}**IM2**$_{A}$	0.40	-0.70	-0.22	0.05	0.47	0.00
^{4}**TS3**$_{A}$	0.41	-0.70	-0.31	-0.07	0.66	0.00
^{4}**Prod**$_{A}$	0.45	-0.64	-0.40	-0.15	0.74	0.00
^{2}**Re**$_{A}$	0.50	-0.49	0.07	-0.03	-0.04	0.00
^{2}**TS1**$_{A}$	0.44	-0.68	0.15	-0.02	0.11	0.00
^{2}**IM1**$_{A}$	0.40	-0.71	-0.23	0.04	0.50	0.00
^{2}**TS2**$_{A}$	0.40	-0.72	-0.22	0.04	0.49	0.00
^{2}**IM2**$_{A}$	0.40	-0.70	-0.22	0.05	0.47	0.00
^{2}**TS3**$_{A}$	0.30	-0.67	-0.34	0.02	0.69	0.00
^{2}**Prod**$_{A}$	0.20	-0.65	-0.40	0.05	0.81	0.00

Table S3: Absolute energies and free energies (in au) of UB3LYP/BS1 optimized geometries in Gaussian for gliotoxin activation by a P450 Cpd I model B.

	E [BS1, au]	ZPE [au]	G [au]	E [BS2, au]
^{4}**Re**$_{B}$	-8225.094696	2.332048	-8222.990602	-8227.003702
^{4}**TS1**$_{B}$	-8225.073214	2.329253	-8222.972965	-8226.985401
^{4}**TS1'**$_{B}$	-8225.063979	2.326174	-8222.968620	-8226.973775
^{4}**IM1**$_{B}$	-8225.097739	2.330807	-8222.998232	-8227.013633
^{4}**TS2**$_{B}$	-8225.045105	2.330543	-8222.941527	-8226.962439
^{4}**IM2**$_{B}$	-8225.068026	2.331149	-8222.965123	-8226.983669
^{4}**TS3**$_{B}$	-8225.049160	2.330617	-8222.947018	-8226.965595
^{4}**Pr**$_{B}$	-8225.128108	2.334860	-8223.024733	-8227.039191
^{4}**TS4**$_{B}$	-8225.072149	2.330435	-8222.965703	-8226.981884
^{4}**IM4**$_{B}$	-8225.080226	2.331037	-8222.982157	-8226.987918
^{2}**Re**$_{B}$	-8225.095120	2.331897	-8222.990649	-8227.004148
^{2}**TS1**$_{B}$	-8225.075051	2.328343	-8222.970391	-8226.988297
^{2}**TS1'**$_{B}$	-8225.064648	2.326441	-8222.968462	-8226.974589
^{2}**IM1**$_{B}$	-8225.097740	2.330803	-8222.997449	-8227.013602
^{2}**TS2**$_{B}$	-8225.045129	2.330538	-8222.940849	-8226.962472
^{2}**IM2**$_{B}$	-8225.068046	2.331164	-8222.964349	-8226.983721
^{2}**TS3**$_{B}$	-8225.058903	2.330349	-8222.956953	-8226.968854
^{2}**Pr**$_{B}$	-8225.131662	2.336287	-8223.023485	-8227.039240
^{2}**TS4**$_{B}$	-8225.073269	2.331630	-8222.970530	-8226.982023
^{2}**IM4**$_{B}$	-8225.081376	2.332303	-8222.978815	-8226.984972

Table S4: Relative energies and free energies (in kcal mol^{-1}) of UB3LYP/BS1 optimized geometries in Gaussian for gliotoxin activation by a P450 Cpd I model B.

	ΔE [BS1]	ΔE+ZPE	ΔG [BS1]	ΔE [BS2]	ΔE+ZPE	ΔG [BS2]
^{4}**Re**$_{B}$	0.00	0.00	0.00	0.00	0.00	0.00
^{4}**TS1**$_{B}$	13.48	11.73	11.07	11.48	9.73	9.07
^{4}**TS1'**$_{B}$	19.27	15.59	13.79	18.78	15.09	13.30
^{4}**IM1**$_{B}$	-1.91	-2.69	-4.79	-6.23	-7.01	-9.11
^{4}**TS2**$_{B}$	31.12	30.17	30.80	25.89	24.95	25.57
^{4}**IM2**$_{B}$	16.74	16.17	15.99	12.57	12.01	11.82
^{4}**TS3**$_{B}$	28.57	27.68	27.35	23.91	23.01	22.69
^{4}**Pr**$_{B}$	-20.97	-19.20	-21.42	-22.27	-20.51	-22.72
^{4}**TS4**$_{B}$	14.15	13.14	15.62	13.69	12.68	15.17
^{4}**IM4**$_{B}$	9.08	8.45	5.30	9.90	9.27	6.12
^{2}**Re**$_{B}$	-0.27	-0.36	-0.03	-0.28	-0.37	-0.04
^{2}**TS1**$_{B}$	12.33	10.00	12.68	9.67	7.34	10.02
^{2}**TS1'**$_{B}$	18.86	15.34	13.89	18.27	14.75	13.31
^{2}**IM1**$_{B}$	-1.91	-2.69	-4.30	-6.21	-6.99	-8.60
^{2}**TS2**$_{B}$	31.10	30.16	31.22	25.87	24.92	25.99
^{2}**IM2**$_{B}$	16.72	16.17	16.47	12.54	11.98	12.29
^{2}**TS3**$_{B}$	22.46	21.39	21.12	21.87	20.80	20.52
^{2}**Pr**$_{B}$	-23.20	-20.54	-20.63	-22.30	-19.64	-19.74
^{2}**TS4**$_{B}$	13.45	13.18	12.60	13.60	13.34	12.75
^{2}**IM4**$_{B}$	8.36	8.52	7.40	11.75	11.91	10.79

Table S5: Group spin densities and charges (in au) of UB3LYP/BS1 optimized geometries in Gaussian for gliotoxin activation by a P450 Cpd I model B.

(a) Spin densities.

	Fe	O	Por	SH	Sub	Prot	Sum
^{4}**Re**$_B$	1.24	0.78	0.59	0.38	0.01	0.00	3.00
^{4}**TS1**$_B$	1.65	0.37	0.64	0.11	0.22	0.00	3.00
^{4}**TS1'**$_B$	1.56	0.49	0.08	0.06	0.80	0.01	3.00
^{4}**IM1**$_B$	1.79	0.22	-0.11	0.10	0.99	0.01	3.00
^{4}**TS2**$_B$	1.80	0.23	-0.12	0.08	1.00	0.00	3.00
^{4}**IM2**$_B$	1.79	0.22	-0.11	0.10	1.00	0.00	3.00
^{4}**TS3**$_B$	2.29	-0.08	-0.10	0.21	0.68	0.00	3.00
^{4}**Pr**$_B$	2.51	0.00	0.03	0.46	0.00	0.00	3.00
^{4}**TS4**$_B$	0.98	0.32	0.10	0.13	1.45	0.00	3.00
^{4}**IM4**$_B$	2.47	0.04	0.06	0.48	-0.04	-0.01	3.00
^{2}**Re**$_B$	1.39	0.73	-0.66	-0.46	0.01	0.00	1.00
^{2}**TS1**$_B$	1.81	0.32	-0.85	-0.08	-0.19	-0.01	1.00
^{2}**TS1'**$_B$	1.62	0.47	-0.31	-0.02	-0.76	0.00	1.00
^{2}**IM1**$_B$	1.79	0.22	-0.11	0.10	-0.99	-0.01	1.00
^{2}**TS2**$_B$	1.80	0.23	-0.12	0.08	-0.99	0.00	1.00
^{2}**IM2'**$_B$	1.01	0.05	-0.69	-0.35	0.98	0.00	1.00
^{2}**IM2**$_B$	1.78	0.23	-0.11	0.09	-0.99	0.00	1.00
^{2}**TS3**$_B$	1.11	-0.08	-0.54	-0.15	0.65	0.00	1.00
^{2}**Pr**$_B$	1.14	0.00	-0.10	-0.04	0.00	0.00	1.00
^{2}**TS4**$_B$	0.99	0.32	0.10	0.13	-0.53	0.00	1.00
^{2}**IM4**$_B$	1.20	0.04	-0.13	-0.07	-0.04	-0.01	1.00

(b) Charges.

	Fe	O	Por	SH	Sub	Prot	Sum
^{4}**Re**$_B$	0.47	-0.52	0.07	-0.06	-0.05	0.10	0.00
^{4}**TS1**$_B$	0.41	-0.70	0.31	0.05	-0.14	0.07	0.00
^{4}**TS1'**$_B$	0.43	-0.70	-0.18	-0.04	0.40	0.09	0.00
^{4}**IM1**$_B$	0.39	-0.70	-0.23	0.07	0.40	0.09	0.00
^{4}**TS2**$_B$	0.40	-0.73	-0.26	0.05	0.46	0.09	0.00
^{4}**IM2**$_B$	0.38	-0.71	-0.25	0.05	0.43	0.09	0.00
^{4}**TS3**$_B$	0.43	-0.69	-0.36	-0.06	0.60	0.09	0.00
^{4}**Pr**$_B$	0.46	-0.64	-0.46	-0.16	0.73	0.08	0.00
^{4}**TS4**$_B$	0.25	-0.60	-0.37	0.01	0.60	0.10	0.00
^{4}**IM4**$_B$	0.45	-0.62	-0.38	-0.15	0.62	0.08	0.00
^{2}**Re**$_B$	0.48	-0.53	0.05	-0.05	-0.06	0.10	0.00
^{2}**TS1**$_B$	0.42	-0.71	0.31	0.03	-0.14	0.08	0.00
^{2}**TS1'**$_B$	0.43	-0.70	-0.18	-0.04	0.41	0.09	0.00
^{2}**IM1**$_B$	0.39	-0.70	-0.23	0.07	0.40	0.09	0.00
^{2}**TS2**$_B$	0.40	-0.73	-0.26	0.05	0.46	0.09	0.00
^{2}**IM2'**$_B$	0.33	-0.73	-0.05	-0.04	0.40	0.09	0.00
^{2}**IM2**$_B$	0.38	-0.71	-0.25	0.05	0.43	0.09	0.00
^{2}**TS3**$_B$	0.30	-0.71	-0.23	-0.06	0.63	0.09	0.00
^{2}**Pr**$_B$	0.23	-0.64	-0.49	0.04	0.78	0.08	0.00
^{2}**TS4**$_B$	0.25	-0.60	-0.37	0.01	0.61	0.10	0.00
^{2}**IM4**$_B$	0.27	-0.63	-0.38	0.01	0.63	0.09	0.00

Table S6: Absolute (in au) and relative (in kcal mol^{-1}) energies and free energies of UB3LYP/BS1 optimized geometries in Gaussian for gliotoxin activation by a P450 Cpd I model G.

	E [BS1, au]	ZPE [au]	G [au]	E [BS2, au]
^{4}**Re**$_G$	-9659.023178	2.748285	-9656.525310	-9661.406558
^{4}**TS1**$_G$	-9659.001007	2.743864	-9656.505741	-9661.388051
^{4}**IM1**$_G$	-9659.011753	2.745122	-9656.522346	-9661.397701

Table S7: Group spin densities and charges (in au) of UB3LYP/BS1 optimized geometries in Gaussian for gliotoxin activation by a P450 Cpd I model G.

	ΔE [BS1]	ΔE+ZPE	ΔG	ΔE [BS2]	ΔE+ZPE	ΔG
^{4}**Re**$_G$	0.00	0.00	0.00	0.00	0.00	0.00
^{4}**TS1**$_G$	13.91	11.14	12.28	11.61	8.84	9.98
^{4}**IM1**$_G$	7.17	5.18	1.86	5.56	3.57	0.25

Cartesian coordinates:

Model B structures:

$^{4}Re_{B}$:

6	136.938461000	2.745774000	13.300385000
6	138.331481000	2.843834000	12.671116000
8	139.206372000	2.007417000	12.878943000
6	136.901364000	1.890405000	14.575923000
6	137.712447000	2.526321000	15.713744000
6	135.450862000	1.629694000	15.006808000
1	136.542008000	3.751498000	13.499878000
1	137.371862000	0.931070000	14.328977000
1	137.682739000	1.903184000	16.616096000
1	137.311953000	3.516661000	15.978149000
1	138.759360000	2.648747000	15.422652000
1	135.411998000	0.967531000	15.880960000
1	134.949158000	2.571487000	15.275884000
1	134.865912000	1.167355000	14.202993000
7	138.503512000	3.917942000	11.844034000
6	139.711610000	4.138752000	11.066692000
6	139.497678000	4.007629000	9.540903000
6	139.140687000	2.612242000	9.077936000
6	140.125611000	1.729145000	8.619396000
6	137.814524000	2.152285000	9.101859000
6	139.808473000	0.438387000	8.191301000
6	137.481632000	0.864601000	8.689831000
6	138.480781000	0.004013000	8.225210000
8	138.096324000	-1.242501000	7.793410000
1	137.747423000	4.586107000	11.744305000
1	140.441389000	3.405236000	11.417062000
1	140.427098000	4.330198000	9.052990000
1	138.720218000	4.714996000	9.224443000
1	141.163759000	2.053223000	8.588786000
1	137.023624000	2.817069000	9.438296000
1	140.592564000	-0.226160000	7.831138000
1	136.452946000	0.519640000	8.704719000
1	138.888927000	-1.745135000	7.548148000
6	134.862124000	5.619060000	17.604475000
6	133.678672000	5.657320000	16.647615000
8	133.382862000	6.647986000	15.980810000
6	135.315628000	7.007659000	18.056014000
1	134.615837000	4.984474000	18.465906000
1	134.532134000	7.516813000	18.627070000
1	136.202477000	6.927355000	18.691778000
1	135.561635000	7.631989000	17.193551000
7	132.935952000	4.502062000	16.572074000
6	132.039452000	4.265500000	15.442952000
6	130.820818000	5.186774000	15.369078000
8	130.220035000	5.324552000	14.303245000
1	133.312420000	3.682894000	17.032422000
1	132.562590000	4.383539000	14.488412000
7	130.454382000	5.823526000	16.509005000
6	129.395588000	6.819123000	16.508725000
6	129.636342000	8.034552000	15.595508000
8	128.675498000	8.727241000	15.252876000
1	131.035028000	5.702239000	17.326792000
1	128.455078000	6.375548000	16.171035000
7	130.907956000	8.262882000	15.190499000
6	131.200423000	9.313310000	14.222210000
6	130.482692000	9.107719000	12.880684000
8	130.356551000	10.080827000	12.118014000
6	132.711597000	9.478334000	13.998719000
6	133.488953000	9.997468000	15.218421000
16	132.862735000	11.541573000	15.989712000
6	133.096368000	12.743166000	14.631858000
1	131.661192000	7.640311000	15.475795000
1	130.799268000	10.256752000	14.607449000
1	133.150662000	8.516494000	13.700743000
1	132.839928000	10.154340000	13.147768000
1	134.538404000	10.140301000	14.937011000
1	133.476177000	9.258547000	16.024810000
1	132.758542000	13.710646000	15.013354000
1	132.498159000	12.485395000	13.753481000
1	134.150877000	12.829148000	14.352499000
7	129.966790000	7.895182000	12.613529000
6	129.178048000	7.647119000	11.414071000
6	128.015069000	8.635505000	11.279919000
8	127.583079000	8.926528000	10.148605000
6	128.656152000	6.202334000	11.407498000
1	130.129857000	7.115638000	13.246436000
1	129.811131000	7.806099000	10.537555000
1	128.140480000	6.000790000	10.465270000
1	127.974650000	6.020847000	12.245087000
1	129.495172000	5.506193000	11.495033000
7	127.453509000	9.155036000	12.387896000
6	126.370512000	10.118597000	12.247330000
6	126.770025000	11.456014000	11.597592000
8	125.916798000	12.136989000	11.021747000
1	127.841246000	8.952186000	13.308745000
1	125.961739000	10.322083000	13.241505000
1	125.577149000	9.701872000	11.622226000
7	128.057465000	11.849802000	11.745689000
6	128.566889000	13.044412000	11.098316000
6	129.358948000	12.851848000	9.792724000
8	129.582096000	13.834694000	9.089207000
1	128.727698000	11.218178000	12.176900000
1	127.719420000	13.687910000	10.858237000
7	129.790374000	11.590025000	9.504512000
6	130.639680000	11.334898000	8.346562000
6	130.010910000	10.424279000	7.270103000
8	130.532843000	10.372291000	6.156523000
6	131.996164000	10.725825000	8.752186000
6	132.839360000	11.588730000	9.645622000
6	132.810815000	12.945074000	9.871419000
7	133.874908000	11.024838000	10.375866000
6	134.458911000	12.020127000	11.018125000
7	133.847290000	13.200829000	10.749704000
1	129.805573000	10.908370000	10.259321000
1	130.792787000	12.298574000	7.856633000
1	132.538153000	10.504377000	7.824693000
1	131.829993000	9.763802000	9.250535000
1	132.145957000	13.718833000	9.518001000
1	135.312555000	11.945912000	11.676160000
1	134.142099000	14.103266000	11.099884000
7	128.931642000	9.687060000	7.640875000
6	128.335887000	8.703132000	6.744162000
6	126.863705000	8.970244000	6.388745000
8	126.423825000	8.596680000	5.290894000
6	128.494507000	7.248858000	7.252521000
8	127.546860000	6.872129000	8.234828000
1	128.601984000	9.749975000	8.597583000
1	128.891400000	8.786282000	5.808340000
1	129.531457000	7.130220000	7.606123000
1	128.345303000	6.572429000	6.408245000
1	127.552429000	7.547627000	8.940338000
7	126.094555000	9.595469000	7.302342000
6	124.667300000	9.816399000	7.109350000
6	124.185641000	10.895337000	8.073751000
8	124.400686000	10.448435000	9.403556000
1	126.413988000	9.694267000	8.259763000
1	124.109227000	8.886895000	7.286920000
1	124.727783000	11.831023000	7.875954000
1	123.115458000	11.081986000	7.891817000
1	124.750910000	11.181967000	9.947835000
7	133.369243000	15.790193000	4.644266000
6	133.965604000	16.314648000	5.895059000
6	135.365928000	15.731698000	6.185132000
8	135.992027000	16.099617000	7.185000000
6	132.954878000	15.956218000	7.014336000
6	132.127023000	14.815118000	6.402368000
6	132.027405000	15.238482000	4.934118000
1	134.115355000	17.401415000	5.854633000
1	132.308087000	16.817328000	7.219779000
1	133.462600000	15.692530000	7.945415000
1	132.670306000	13.865796000	6.478279000
1	131.150878000	14.688109000	6.879473000
1	131.800988000	14.403481000	4.262106000
1	131.233140000	15.993059000	4.812755000
7	135.805658000	14.834945000	5.280071000

6	137.128497000	14.224975000	5.293543000
6	137.129254000	12.724758000	4.927463000
6	136.739877000	11.797748000	6.098375000
6	138.526479000	12.336480000	4.414231000
6	135.356164000	12.021249000	6.718851000
1	135.156989000	14.666392000	4.514966000
1	137.557029000	14.370562000	6.288321000
1	136.407909000	12.575118000	4.106760000
1	137.506788000	11.897015000	6.876527000
1	136.801900000	10.760964000	5.732908000
1	138.809013000	12.922852000	3.531541000
1	138.563490000	11.276254000	4.137475000
1	139.278078000	12.507793000	5.194215000
1	135.143376000	11.252761000	7.471975000
1	134.558395000	11.977884000	5.965817000
1	135.290187000	12.992814000	7.216946000
16	134.585910000	4.337423000	2.740912000
1	133.360505000	4.780670000	2.380436000
6	140.953733000	12.671563000	9.583525000
6	139.831152000	13.547910000	9.037717000
8	139.248564000	13.279689000	7.987524000
6	140.398376000	11.519385000	10.446689000
6	141.463839000	10.533120000	10.968949000
6	140.853758000	9.617384000	12.042279000
6	142.082065000	9.697629000	9.837346000
1	141.487689000	12.274476000	8.716067000
1	139.871664000	11.953194000	11.309333000
1	139.647399000	10.963432000	9.869908000
1	142.267905000	11.121783000	11.438555000
1	141.598925000	8.910853000	12.427632000
1	140.477336000	10.199731000	12.892898000
1	140.016876000	9.040203000	11.631269000
1	142.843852000	9.013393000	10.229436000
1	142.562440000	10.320003000	9.074175000
1	141.311670000	9.092705000	9.342357000
7	139.491583000	14.625281000	9.806381000
6	138.354174000	15.481785000	9.487961000
6	136.991599000	14.933245000	9.967795000
6	136.795933000	14.963579000	11.466655000
6	137.317117000	13.956974000	12.298606000
6	136.094598000	16.018606000	12.074780000
6	137.154444000	14.008861000	13.685329000
6	135.924791000	16.072079000	13.461104000
6	136.456634000	15.067903000	14.272728000
1	139.955935000	14.749135000	10.694583000
1	138.302257000	15.592246000	8.403913000
1	136.224387000	15.528972000	9.463889000
1	136.895408000	13.910542000	9.585788000
1	137.852009000	13.122943000	11.850560000
1	135.686681000	16.811523000	11.450932000
1	137.572738000	13.221643000	14.307817000
1	135.378803000	16.900080000	13.905993000
1	136.328818000	15.108433000	15.350946000
7	136.177485000	4.546327000	5.261505000
7	133.879446000	6.197478000	4.990947000
7	134.527041000	2.227049000	5.192909000
6	136.957973000	2.231331000	5.472179000
6	136.186619000	6.994816000	5.208504000
6	131.442296000	6.211402000	4.711238000
6	132.228254000	1.441778000	4.885238000
6	137.166135000	3.599034000	5.390828000
6	134.821513000	7.196312000	5.084535000
6	131.255625000	4.831008000	4.667468000
6	133.584515000	1.237443000	5.117825000
8	134.010847000	4.237530000	6.848078000
6	138.459174000	4.232777000	5.458914000
6	134.184416000	8.490285000	5.038292000
6	129.976501000	4.201115000	4.444439000
6	134.199341000	-0.054900000	5.294324000
6	138.241867000	5.573422000	5.382137000
6	132.847008000	8.273502000	4.916091000
6	130.198281000	2.858262000	4.494977000
6	135.532182000	0.166992000	5.473220000
6	136.817020000	5.757825000	5.268916000
6	132.665830000	6.841497000	4.877625000
6	131.607782000	2.675492000	4.743235000
6	135.721544000	1.594597000	5.394935000
7	132.238066000	3.891860000	4.833050000
26	134.191230000	4.219061000	5.211782000
1	137.831231000	1.604424000	5.618174000
1	136.817369000	7.876788000	5.261582000
1	130.556130000	6.826977000	4.578025000
1	131.598606000	0.559691000	4.815257000
6	133.270754000	7.281946000	10.105346000
7	134.330196000	8.139537000	10.145051000
8	132.096630000	7.633453000	10.108444000
16	136.015958000	6.760572000	11.898913000
6	133.712897000	5.803204000	10.085517000
7	134.817415000	5.624616000	9.180658000
8	136.860274000	6.418132000	8.489783000
16	134.272991000	5.541163000	11.917247000
6	132.531287000	4.859244000	9.792585000
8	138.019880000	8.274816000	10.163992000
6	132.652182000	3.436426000	10.303618000
6	133.333882000	2.453782000	9.570374000
6	133.433412000	1.149042000	10.057293000
6	132.847783000	0.802924000	11.277744000
6	132.148218000	1.769105000	12.002867000
6	132.048061000	3.075082000	11.517462000
6	135.853772000	6.498568000	9.197081000
6	135.660186000	7.629901000	10.215275000
6	136.700206000	8.745142000	10.026623000
1	133.760830000	2.708631000	8.603876000
1	133.954063000	0.395988000	9.471320000
1	131.648061000	5.338709000	10.219408000
1	132.403287000	4.863637000	8.703626000
1	132.918234000	-0.216313000	11.648635000
1	131.662951000	1.501737000	12.938668000
1	131.484359000	3.815355000	12.082578000
1	136.519200000	9.190224000	9.036959000
1	136.543028000	9.519511000	10.781370000
1	134.155674000	9.154543000	10.292613000
1	134.734138000	4.960855000	8.395026000
1	138.111927000	7.593535000	9.467131000
8	128.298571000	7.100232000	3.867408000
1	127.567875000	7.587783000	4.318474000
1	128.418713000	7.570639000	3.029214000
1	136.272188000	2.307359000	12.543863000
1	140.101037000	5.142191000	11.285063000
1	135.681190000	5.107571000	17.077977000
1	129.248247000	7.170730000	17.533831000
1	129.215400000	13.590593000	11.793178000
1	124.496668000	10.113296000	6.070773000
1	133.336730000	16.499138000	3.918455000
1	137.772592000	14.764101000	4.582560000
1	141.665793000	13.266385000	10.172010000
1	139.396033000	3.702022000	5.566048000
1	134.710396000	9.434384000	5.097798000
1	129.061275000	4.750550000	4.261663000
1	133.667465000	-0.997701000	5.280266000
1	138.964285000	6.378684000	5.409413000
1	132.041634000	8.997218000	4.892069000
1	129.486272000	2.051557000	4.375232000
1	136.321192000	-0.549775000	5.661534000
1	138.542498000	16.469307000	9.922246000
1	131.684004000	3.232396000	15.502277000

$^{4}TS1_{B}$:

6	136.228796000	1.979671000	13.974052000
6	137.444629000	2.140154000	13.053459000
8	138.511228000	1.560025000	13.267547000
6	136.558382000	1.321845000	15.322712000
6	137.457247000	2.208761000	16.194666000
6	135.270782000	0.942278000	16.067662000
1	135.754960000	2.960250000	14.127313000
1	137.116226000	0.403474000	15.102139000
1	137.708083000	1.707466000	17.137713000
1	136.953572000	3.153824000	16.446541000
1	138.389830000	2.441952000	15.675022000
1	135.492089000	0.446301000	17.020310000

1	134.673453000	1.839479000	16.294457000
1	134.639521000	0.270470000	15.474026000
7	137.235038000	2.950473000	11.982473000
6	138.248460000	3.229523000	10.974219000
6	137.900852000	2.690501000	9.577872000
6	137.706506000	1.189969000	9.446016000
6	138.394855000	0.253341000	10.228383000
6	136.830568000	0.695722000	8.465763000
6	138.226769000	-1.118756000	10.028568000
6	136.655795000	-0.670643000	8.251139000
6	137.363272000	-1.587247000	9.034944000
8	137.164561000	-2.922775000	8.791986000
1	136.328762000	3.393230000	11.875202000
1	139.183054000	2.809624000	11.351866000
1	138.704155000	3.020576000	8.901162000
1	137.000535000	3.205344000	9.222772000
1	139.042196000	0.582689000	11.034911000
1	136.265350000	1.399622000	7.859809000
1	138.766931000	-1.825743000	10.657150000
1	135.971211000	-1.037589000	7.491977000
1	137.705014000	-3.432891000	9.415152000
6	134.971852000	5.685098000	17.597992000
6	133.779563000	5.731971000	16.653284000
8	133.446746000	6.749192000	16.045445000
6	135.333167000	7.051869000	18.181036000
1	134.784156000	4.952720000	18.393828000
1	134.520434000	7.449686000	18.798139000
1	136.227363000	6.971702000	18.806531000
1	135.531374000	7.770932000	17.382436000
7	133.077983000	4.557937000	16.515578000
6	132.203680000	4.340190000	15.364936000
6	130.924436000	5.176299000	15.334430000
8	130.285363000	5.278399000	14.286213000
1	133.478473000	3.731730000	16.942719000
1	132.726220000	4.564580000	14.428457000
7	130.554880000	5.800857000	16.480429000
6	129.479694000	6.779012000	16.481228000
6	129.698156000	7.983179000	15.546104000
8	128.725503000	8.648856000	15.181886000
1	131.167701000	5.723467000	17.279673000
1	128.542938000	6.317195000	16.158920000
7	130.968028000	8.228821000	15.147480000
6	131.255481000	9.266659000	14.165685000
6	130.565498000	9.025749000	12.816477000
8	130.447353000	9.985335000	12.033478000
6	132.767155000	9.460099000	13.968110000
6	133.504519000	10.041687000	15.184434000
16	132.819993000	11.592084000	15.891953000
6	133.010663000	12.745400000	14.486447000
1	131.734166000	7.636695000	15.463806000
1	130.827350000	10.208501000	14.525948000
1	133.235022000	8.498928000	13.716375000
1	132.900851000	10.107103000	13.096040000
1	134.554588000	10.205145000	14.917268000
1	133.496614000	9.330356000	16.015327000
1	132.638894000	13.715096000	14.828910000
1	132.422145000	12.429926000	13.620355000
1	134.061275000	12.856612000	14.202113000
7	130.054629000	7.809338000	12.563956000
6	129.275446000	7.542159000	11.362398000
6	128.096245000	8.511715000	11.219710000
8	127.643619000	8.767869000	10.087757000
6	128.776349000	6.089579000	11.360999000
1	130.216599000	7.038223000	13.207960000
1	129.910234000	7.704940000	10.487303000
1	128.244961000	5.881125000	10.428990000
1	128.113345000	5.895805000	12.210467000
1	129.628281000	5.407926000	11.429874000
7	127.550468000	9.057396000	12.323137000
6	126.468327000	10.020015000	12.177711000
6	126.863021000	11.336728000	11.484041000
8	126.002987000	12.007721000	10.905747000
1	127.945718000	8.870100000	13.244784000
1	126.078449000	10.251074000	13.173390000
1	125.661565000	9.591133000	11.578368000

7	128.156263000	11.722554000	11.591170000
6	128.669585000	12.878132000	10.879352000
6	129.463342000	12.606955000	9.588042000
8	129.669912000	13.539455000	8.815048000
1	128.827197000	11.104453000	12.041208000
1	127.825468000	13.511872000	10.604691000
7	129.920695000	11.335581000	9.393158000
6	130.816526000	11.015074000	8.286624000
6	130.228925000	10.016672000	7.263921000
8	130.870990000	9.748116000	6.242945000
6	132.161323000	10.463285000	8.792002000
6	132.969019000	11.399077000	9.641800000
6	132.907320000	12.763930000	9.799148000
7	134.012359000	10.894389000	10.403494000
6	134.568017000	11.932682000	10.999450000
7	133.932377000	13.085852000	10.669614000
1	129.945031000	10.710897000	10.196979000
1	130.971305000	11.944641000	7.731616000
1	132.746163000	10.171401000	7.913550000
1	131.986980000	9.540030000	9.356423000
1	132.234350000	13.507014000	9.398375000
1	135.419097000	11.910162000	11.664758000
1	134.197583000	14.010097000	10.983757000
7	129.021503000	9.479911000	7.560388000
6	128.372456000	8.517060000	6.676034000
6	126.917417000	8.871724000	6.326166000
8	126.490414000	8.627647000	5.187467000
6	128.464132000	7.054396000	7.176343000
8	127.533389000	6.720955000	8.185908000
1	128.613568000	9.696914000	8.462071000
1	128.924242000	8.561946000	5.736308000
1	129.504740000	6.875560000	7.492292000
1	128.249543000	6.395629000	6.330448000
1	127.605966000	7.385185000	8.899323000
7	126.146803000	9.432943000	7.280369000
6	124.739327000	9.744211000	7.058936000
6	124.289347000	10.824044000	8.036001000
8	124.423869000	10.332149000	9.360017000
1	126.432245000	9.417207000	8.254341000
1	124.124507000	8.844240000	7.196540000
1	124.895509000	11.728653000	7.885649000
1	123.240486000	11.082020000	7.818809000
1	124.799247000	11.037460000	9.925368000
7	132.888407000	16.277647000	4.708574000
6	133.425239000	16.663846000	6.034686000
6	134.889682000	16.222641000	6.243956000
8	135.472886000	16.506424000	7.295696000
6	132.482195000	15.997652000	7.069019000
6	131.806126000	14.873065000	6.269318000
6	131.632153000	15.518482000	4.891729000
1	133.433490000	17.752645000	6.173747000
1	131.728935000	16.719211000	7.406133000
1	133.030792000	15.654144000	7.949476000
1	132.475176000	14.007537000	6.190853000
1	130.866080000	14.538347000	6.715270000
1	131.504842000	14.786544000	4.086099000
1	130.746070000	16.174239000	4.892207000
7	135.427138000	15.532938000	5.218171000
6	136.810682000	15.082372000	5.152255000
6	136.974869000	13.611390000	4.708817000
6	136.743136000	12.587812000	5.840581000
6	138.387899000	13.418617000	4.131825000
6	135.364985000	12.606553000	6.512212000
1	134.796255000	15.405005000	4.430729000
1	137.260083000	15.232998000	6.136747000
1	136.244541000	13.415921000	3.906052000
1	137.519174000	12.745582000	6.600190000
1	136.918994000	11.586589000	5.418252000
1	138.564737000	14.076874000	3.272523000
1	138.539553000	12.385111000	3.798679000
1	139.142591000	13.642497000	4.895217000
1	135.270449000	11.776592000	7.223878000
1	134.555009000	12.508619000	5.778059000
1	135.201523000	13.532499000	7.071025000
16	135.266071000	4.847152000	2.987514000

1	134.074534000	5.375636000	2.631598000
6	140.922205000	13.712993000	9.133979000
6	139.699989000	14.526612000	8.722293000
8	139.151207000	14.360203000	7.633447000
6	140.504113000	12.343537000	9.709686000
6	141.673658000	11.394797000	10.042869000
6	141.176984000	10.222804000	10.904672000
6	142.380171000	10.880807000	8.778966000
1	141.525735000	13.578363000	8.231942000
1	139.919446000	12.513453000	10.625037000
1	139.829102000	11.851584000	8.996596000
1	142.408949000	11.962591000	10.634943000
1	141.994073000	9.527763000	11.132615000
1	140.769718000	10.579051000	11.859098000
1	140.383891000	9.654654000	10.402698000
1	143.219828000	10.226581000	9.041287000
1	142.778009000	11.695605000	8.163677000
1	141.687123000	10.299622000	8.156945000
7	139.236887000	15.416504000	9.649793000
6	137.985142000	16.144798000	9.476824000
6	136.734835000	15.370375000	9.950903000
6	136.653860000	15.173607000	11.448253000
6	137.315088000	14.108125000	12.084484000
6	135.928117000	16.069016000	12.251690000
6	137.262153000	13.949389000	13.471544000
6	135.869259000	15.912297000	13.639020000
6	136.538345000	14.852376000	14.255169000
1	139.682485000	15.438696000	10.556103000
1	137.861025000	16.356735000	8.414252000
1	135.866738000	15.924867000	9.581229000
1	136.730787000	14.404357000	9.433562000
1	137.869599000	13.392819000	11.481761000
1	135.410006000	16.902391000	11.781261000
1	137.785574000	13.119776000	13.940374000
1	135.300849000	16.619108000	14.238066000
1	136.495281000	14.729599000	15.333867000
7	136.752762000	4.403146000	5.479145000
7	134.686064000	6.362197000	5.555895000
7	134.843092000	2.400552000	4.802531000
6	137.233354000	2.016230000	5.167272000
6	137.068116000	6.792531000	5.929342000
6	132.294044000	6.764703000	5.161002000
6	132.494732000	2.022198000	4.222113000
6	137.605679000	3.324563000	5.437114000
6	135.745035000	7.194823000	5.822273000
6	131.944043000	5.473040000	4.782849000
6	133.798213000	1.593270000	4.436423000
8	134.445051000	3.986703000	6.911248000
6	138.954670000	3.750017000	5.702467000
6	135.288668000	8.554523000	5.960831000
6	130.620868000	5.082917000	4.364232000
6	134.248992000	0.234467000	4.275402000
6	138.908337000	5.097082000	5.902275000
6	133.939447000	8.540619000	5.768653000
6	130.684200000	3.745938000	4.105410000
6	135.585043000	0.231895000	4.543893000
6	137.531820000	5.488949000	5.765665000
6	133.579019000	7.170987000	5.505300000
6	132.039564000	3.328887000	4.356350000
6	135.942284000	1.589953000	4.865802000
7	132.801574000	4.398630000	4.752248000
26	134.758534000	4.377706000	5.231195000
1	138.014526000	1.263308000	5.184487000
1	137.804155000	7.554677000	6.159430000
1	131.519575000	7.526421000	5.168408000
1	131.768530000	1.274551000	3.918446000
6	133.430825000	7.079624000	10.129511000
7	134.479292000	7.948879000	10.170195000
8	132.250139000	7.426206000	10.122029000
16	136.147804000	6.667465000	12.020241000
6	133.884015000	5.615526000	10.066510000
7	134.993107000	5.354297000	9.269972000
8	137.088292000	6.112817000	8.730029000
16	134.434249000	5.475168000	12.104158000
6	132.706468000	4.648341000	9.896765000
8	138.161328000	8.088888000	10.256306000
6	132.827676000	3.278243000	10.532732000
6	133.667215000	2.290919000	9.996594000
6	133.748797000	1.027663000	10.584977000
6	132.979294000	0.725045000	11.712163000
6	132.131882000	1.696576000	12.247930000
6	132.058565000	2.963539000	11.663531000
6	136.002950000	6.239235000	9.340532000
6	135.805054000	7.446059000	10.272985000
6	136.848309000	8.549288000	10.051654000
1	134.246167000	2.512862000	9.106117000
1	134.409736000	0.280276000	10.155534000
1	131.817362000	5.155046000	10.276366000
1	132.575736000	4.542877000	8.811976000
1	133.029804000	-0.265259000	12.158067000
1	131.512255000	1.464312000	13.111507000
1	131.395980000	3.712347000	12.092804000
1	136.692057000	8.930969000	9.029433000
1	136.667680000	9.373005000	10.748154000
1	134.297679000	8.953570000	10.336650000
1	134.783739000	4.555619000	7.677583000
1	138.226726000	7.307866000	9.663523000
8	128.394675000	7.314121000	3.637233000
1	127.634407000	7.723216000	4.114661000
1	128.741284000	8.034981000	3.090893000
1	135.487503000	1.374280000	13.433313000
1	138.376834000	4.315427000	10.894750000
1	135.816030000	5.289590000	17.015071000
1	129.338792000	7.141964000	17.503344000
1	129.322699000	13.458284000	11.541992000
1	124.618965000	10.074861000	6.024113000
1	132.758102000	17.085134000	4.107321000
1	137.354008000	15.726979000	4.445472000
1	141.533099000	14.255547000	9.868118000
1	139.812763000	3.091244000	5.729091000
1	135.931858000	9.400160000	6.167564000
1	129.782841000	5.765203000	4.252353000
1	133.615437000	-0.595328000	3.990406000
1	139.718772000	5.774032000	6.137260000
1	133.223447000	9.352206000	5.787377000
1	129.892132000	3.089019000	3.769679000
1	136.277319000	-0.599616000	4.525883000
1	138.068577000	17.097660000	10.010797000
1	131.925542000	3.282813000	15.340655000

^{4}TS1'$_B$:

6	136.128732000	2.027379000	13.412607000
6	137.298401000	2.069309000	12.424840000
8	138.423956000	1.674630000	12.728827000
6	136.576076000	1.956292000	14.881190000
6	137.285847000	3.244388000	15.322658000
6	135.382344000	1.640713000	15.793987000
1	135.470901000	2.892640000	13.251656000
1	137.298769000	1.134893000	14.955587000
1	137.600109000	3.178931000	16.372403000
1	136.624632000	4.115975000	15.215749000
1	138.178789000	3.421083000	14.716722000
1	135.700806000	1.546815000	16.840012000
1	134.629116000	2.440664000	15.741115000
1	134.889909000	0.703095000	15.508587000
7	136.986102000	2.551627000	11.188092000
6	137.927326000	2.542237000	10.073054000
6	137.473933000	1.681710000	8.881332000
6	137.110767000	0.239919000	9.189785000
6	137.759598000	-0.512482000	10.178209000
6	136.107602000	-0.397734000	8.441601000
6	137.421623000	-1.847334000	10.409988000
6	135.761905000	-1.730317000	8.659899000
6	136.423427000	-2.462886000	9.650188000
8	136.049065000	-3.769141000	9.835025000
1	136.017532000	2.771797000	10.991982000
1	138.874369000	2.192443000	10.488197000
1	138.289305000	1.714623000	8.143171000
1	136.628611000	2.170768000	8.381014000
1	138.522630000	-0.056645000	10.802177000

1	135.593268000	0.157640000	7.660587000
1	137.936227000	-2.408063000	11.189394000
1	134.988075000	-2.214881000	8.072238000
1	136.603280000	-4.153788000	10.531760000
6	134.690810000	5.595979000	17.910283000
6	133.497232000	5.590671000	16.967372000
8	133.219800000	6.531175000	16.224084000
6	135.152621000	7.003624000	18.289365000
1	134.456308000	5.002570000	18.803666000
1	134.378715000	7.541445000	18.847360000
1	136.048444000	6.951596000	18.915362000
1	135.388299000	7.584511000	17.394299000
7	132.721850000	4.454117000	16.990390000
6	131.821768000	4.149862000	15.881242000
6	130.646671000	5.114018000	15.706516000
8	130.087242000	5.208698000	14.613988000
1	133.084275000	3.663693000	17.508678000
1	132.353839000	4.150539000	14.924185000
7	130.266631000	5.828137000	16.795081000
6	129.235680000	6.848595000	16.703213000
6	129.539616000	8.005095000	15.735227000
8	128.610364000	8.704860000	15.325572000
1	130.818579000	5.739495000	17.636835000
1	128.295821000	6.409767000	16.357656000
7	130.829103000	8.176821000	15.360554000
6	131.183967000	9.177517000	14.362028000
6	130.529730000	8.920874000	12.997268000
8	130.476851000	9.853131000	12.177543000
6	132.705835000	9.323545000	14.212981000
6	133.419161000	9.881499000	15.454267000
16	132.771361000	11.459966000	16.132205000
6	133.145272000	12.618202000	14.768101000
1	131.554179000	7.544867000	15.694533000
1	130.773518000	10.140717000	14.684244000
1	133.152675000	8.348205000	13.976322000
1	132.886462000	9.965587000	13.345616000
1	134.484806000	10.003050000	15.229189000
1	133.351394000	9.173566000	16.285267000
1	132.801677000	13.603291000	15.095406000
1	132.615947000	12.350719000	13.849309000
1	134.221060000	12.672344000	14.574617000
7	129.979008000	7.714672000	12.766120000
6	129.203418000	7.451514000	11.559738000
6	128.098284000	8.496085000	11.351838000
8	127.717323000	8.767090000	10.199075000
6	128.591167000	6.043078000	11.611057000
1	130.069325000	6.967209000	13.450440000
1	129.859561000	7.533795000	10.687991000
1	128.100310000	5.822111000	10.659824000
1	127.865028000	5.956886000	12.425996000
1	129.374511000	5.297770000	11.772925000
7	127.529717000	9.077241000	12.425837000
6	126.488196000	10.074402000	12.223304000
6	126.952478000	11.370793000	11.536185000
8	126.136918000	12.064532000	10.922923000
1	127.877110000	8.887897000	13.365715000
1	126.063279000	10.328914000	13.198717000
1	125.694829000	9.664871000	11.593260000
7	128.253766000	11.716105000	11.688439000
6	128.821741000	12.861489000	11.002234000
6	129.658934000	12.579597000	9.741972000
8	129.954314000	13.519652000	9.008350000
1	128.887758000	11.078079000	12.162168000
1	128.002102000	13.513899000	10.697914000
7	130.047718000	11.289098000	9.527538000
6	130.973104000	10.942598000	8.454061000
6	130.411077000	9.934838000	7.424688000
8	131.095196000	9.632721000	6.444437000
6	132.286525000	10.361939000	9.006820000
6	133.093902000	11.269121000	9.886027000
6	133.093200000	12.636668000	10.024429000
7	134.071692000	10.724148000	10.706573000
6	134.650155000	11.744242000	11.316042000
7	134.087978000	12.920199000	10.940987000
1	129.989730000	10.641605000	10.309851000
1	131.165654000	11.864589000	7.898834000
1	132.887129000	10.045245000	8.146612000
1	132.055125000	9.452341000	9.571605000
1	132.475388000	13.404022000	9.582515000
1	135.465904000	11.689091000	12.022420000
1	134.390673000	13.836637000	11.247017000
7	129.184725000	9.419059000	7.684281000
6	128.563126000	8.436029000	6.800575000
6	127.118589000	8.784527000	6.403404000
8	126.718173000	8.511773000	5.263520000
6	128.644750000	6.985537000	7.333857000
8	127.707037000	6.678758000	8.347103000
1	128.735689000	9.678103000	8.553664000
1	129.143783000	8.457361000	5.877438000
1	129.683757000	6.814201000	7.661847000
1	128.438521000	6.309352000	6.500928000
1	127.752808000	7.377321000	9.028274000
7	126.329034000	9.377469000	7.325712000
6	124.929564000	9.692541000	7.066126000
6	124.474054000	10.815200000	7.991301000
8	124.587619000	10.376802000	9.337124000
1	126.589786000	9.383102000	8.306319000
1	124.301829000	8.805300000	7.227115000
1	125.089543000	11.708229000	7.812491000
1	123.430166000	11.071873000	7.750698000
1	124.948539000	11.103396000	9.883417000
7	132.652052000	16.451324000	4.821920000
6	133.187395000	16.761669000	6.167972000
6	134.662767000	16.342608000	6.340233000
8	135.251811000	16.587361000	7.399033000
6	132.267093000	16.003051000	7.159503000
6	131.599031000	14.930080000	6.285934000
6	131.404752000	15.669568000	4.959792000
1	133.169878000	17.838312000	6.381416000
1	131.506690000	16.683888000	7.559128000
1	132.830205000	15.603608000	8.006528000
1	132.277452000	14.080861000	6.140201000
1	130.669336000	14.552146000	6.717132000
1	131.277384000	14.996374000	4.104507000
1	130.512246000	16.314722000	5.015203000
7	135.200839000	15.710144000	5.279248000
6	136.588973000	15.279789000	5.180996000
6	136.765335000	13.831285000	4.673105000
6	136.558978000	12.755925000	5.761049000
6	138.173096000	13.681264000	4.071261000
6	135.190302000	12.730979000	6.451883000
1	134.564411000	15.609435000	4.492197000
1	137.046256000	15.392960000	6.166878000
1	136.027871000	13.660938000	3.871366000
1	137.344694000	12.885878000	6.516392000
1	136.734144000	11.777160000	5.290289000
1	138.329743000	14.375936000	3.237040000
1	138.334079000	12.664350000	3.694842000
1	138.935144000	13.885215000	4.833130000
1	135.112819000	11.864105000	7.120375000
1	134.371664000	12.660497000	5.724678000
1	135.027568000	13.627163000	7.058056000
16	135.600929000	4.739900000	2.968151000
1	134.301472000	4.907958000	2.636826000
6	140.818854000	13.973056000	9.057510000
6	139.549719000	14.732704000	8.686210000
8	138.963693000	14.530218000	7.623752000
6	140.471749000	12.586507000	9.637876000
6	141.684847000	11.688193000	9.953780000
6	141.242429000	10.487965000	10.805978000
6	142.404730000	11.216987000	8.680968000
1	141.402149000	13.865579000	8.138618000
1	139.893265000	12.731167000	10.561659000
1	139.808391000	12.066675000	8.933806000
1	142.398388000	12.281055000	10.547710000
1	142.086855000	9.821953000	11.019854000
1	140.827058000	10.816883000	11.766697000
1	140.468230000	9.899321000	10.298266000
1	143.273089000	10.596395000	8.931370000
1	142.764277000	12.054061000	8.072157000

1	141.733571000	10.613791000	8.055963000
7	139.087992000	15.613254000	9.623702000
6	137.802820000	16.290219000	9.493377000
6	136.599448000	15.463025000	10.000097000
6	136.582804000	15.242852000	11.496261000
6	137.286560000	14.178749000	12.088021000
6	135.877889000	16.114629000	12.343792000
6	137.297768000	14.000793000	13.473885000
6	135.882870000	15.938382000	13.729960000
6	136.595735000	14.881761000	14.301215000
1	139.570026000	15.665620000	10.509798000
1	137.634377000	16.502972000	8.437125000
1	135.698822000	15.989981000	9.670114000
1	136.612736000	14.504988000	9.468328000
1	137.824569000	13.481202000	11.450852000
1	135.324797000	16.944313000	11.908344000
1	137.857052000	13.175340000	13.907648000
1	135.330378000	16.627816000	14.363142000
1	136.604640000	14.745958000	15.379256000
7	137.327474000	4.373790000	5.325539000
7	135.194881000	6.260224000	5.604907000
7	135.437628000	2.288593000	4.854189000
6	137.866429000	2.002355000	4.992837000
6	137.587974000	6.786040000	5.707808000
6	132.759841000	6.559327000	5.427049000
6	133.061741000	1.818778000	4.475379000
6	138.208909000	3.327943000	5.213861000
6	136.246047000	7.134492000	5.737921000
6	132.429112000	5.254865000	5.083028000
6	134.393108000	1.445321000	4.580037000
8	135.193032000	3.892414000	6.948065000
6	139.563049000	3.810335000	5.348037000
6	135.748460000	8.480736000	5.903306000
6	131.089400000	4.809660000	4.774577000
6	134.884103000	0.100478000	4.381596000
6	139.483695000	5.155981000	5.534694000
6	134.386819000	8.410405000	5.854333000
6	131.180010000	3.476087000	4.511761000
6	136.235375000	0.150699000	4.532979000
6	138.080908000	5.497008000	5.524001000
6	134.055699000	7.020639000	5.646735000
6	132.568651000	3.108041000	4.651348000
6	136.570310000	1.527433000	4.819333000
7	133.315623000	4.206284000	4.985196000
26	135.318278000	4.257225000	5.277062000
1	138.675419000	1.281631000	4.925677000
1	138.314890000	7.583097000	5.829533000
1	131.959394000	7.291518000	5.484002000
1	132.343734000	1.042965000	4.227847000
6	133.523988000	7.029004000	9.868550000
7	134.562276000	7.891345000	10.077991000
8	132.349619000	7.282404000	10.126193000
16	136.579263000	6.879169000	11.800874000
6	133.884099000	5.759950000	9.137255000
7	135.108510000	5.517731000	8.744599000
8	137.317715000	6.073924000	8.707234000
16	134.994062000	6.025980000	12.631852000
6	132.794252000	4.789619000	8.803952000
8	138.203025000	8.407980000	9.930811000
6	132.715165000	3.687109000	9.860762000
6	133.340895000	2.453777000	9.637450000
6	133.240621000	1.433363000	10.587860000
6	132.515944000	1.640048000	11.765323000
6	131.902532000	2.874303000	11.996808000
6	132.012160000	3.896828000	11.052948000
6	136.193076000	6.340582000	9.102836000
6	135.921144000	7.519526000	10.013831000
6	136.851446000	8.703990000	9.672515000
1	133.889012000	2.293361000	8.712510000
1	133.720158000	0.477456000	10.395739000
1	131.849610000	5.334125000	8.757710000
1	133.017141000	4.346549000	7.829079000
1	132.424675000	0.838721000	12.494002000
1	131.323747000	3.048829000	12.900210000
1	131.548944000	4.861865000	11.235869000
1	136.671526000	8.968736000	8.619652000
1	136.574960000	9.561320000	10.289896000
1	134.339645000	8.832877000	10.460823000
1	135.252327000	4.724634000	7.878929000
1	138.416070000	7.618736000	9.396595000
8	128.635500000	6.946827000	3.921777000
1	127.878114000	7.457514000	4.287446000
1	129.066546000	7.563798000	3.312051000
1	135.532042000	1.137585000	13.164786000
1	138.083813000	3.568037000	9.716320000
1	135.500844000	5.061750000	17.392895000
1	129.063114000	7.260504000	17.701572000
1	129.460000000	13.426692000	11.691458000
1	124.831681000	9.983457000	6.017161000
1	132.515188000	17.291017000	4.268087000
1	137.117291000	15.960951000	4.497537000
1	141.426857000	14.538513000	9.776566000
1	140.446173000	3.186380000	5.295696000
1	136.373063000	9.360076000	6.004322000
1	130.218580000	5.454840000	4.722053000
1	134.261325000	-0.754273000	4.150210000
1	140.288301000	5.867211000	5.671550000
1	133.650437000	9.202544000	5.913346000
1	130.387870000	2.791644000	4.235594000
1	136.954565000	-0.654477000	4.454552000
1	137.864231000	17.242570000	10.031097000
1	131.417475000	3.145035000	16.038671000

^{4}IM1$_B$:

6	135.974542000	2.202466000	13.366719000
6	137.190377000	1.910252000	12.480148000
8	138.201099000	1.356626000	12.919315000
6	136.259754000	2.057442000	14.869395000
6	137.209695000	3.150372000	15.379491000
6	134.947721000	2.049077000	15.667750000
1	135.589098000	3.208018000	13.144385000
1	136.758408000	1.090653000	15.008743000
1	137.418127000	3.022197000	16.449621000
1	136.775423000	4.150180000	15.235424000
1	138.162406000	3.110462000	14.844843000
1	135.141247000	1.906955000	16.739164000
1	134.410418000	3.001013000	15.545599000
1	134.278465000	1.244596000	15.338524000
7	137.044806000	2.277215000	11.179435000
6	138.072759000	2.073768000	10.166897000
6	137.652682000	1.132572000	9.027633000
6	137.397627000	-0.327075000	9.368561000
6	137.846834000	-0.938719000	10.544538000
6	136.709129000	-1.127501000	8.440876000
6	137.618364000	-2.297052000	10.783324000
6	136.478461000	-2.481912000	8.663820000
6	136.937120000	-3.074367000	9.845445000
8	136.685186000	-4.410367000	10.027737000
1	136.193470000	2.747965000	10.888829000
1	138.958765000	1.705851000	10.687914000
1	138.441906000	1.193372000	8.262548000
1	136.756089000	1.547206000	8.547365000
1	138.361609000	-0.361635000	11.306504000
1	136.349304000	-0.678123000	7.517135000
1	137.971977000	-2.747802000	11.709928000
1	135.948814000	-3.092325000	7.938396000
1	137.070120000	-4.683154000	10.875064000
6	134.544783000	5.803186000	18.002961000
6	133.321621000	5.819953000	17.099490000
8	133.075964000	6.734437000	16.312504000
6	135.074233000	7.201721000	18.324211000
1	134.318294000	5.244966000	18.920663000
1	134.341377000	7.785289000	18.891849000
1	135.988114000	7.131348000	18.921610000
1	135.301969000	7.745605000	17.404305000
7	132.482093000	4.735774000	17.207074000
6	131.523851000	4.434846000	16.147331000
6	130.417059000	5.473033000	15.955517000
8	129.853546000	5.575651000	14.866714000
1	132.813325000	3.952257000	17.755613000

1	132.018860000	4.344086000	15.174771000
7	130.093648000	6.241432000	17.025070000
6	129.115122000	7.310435000	16.904886000
6	129.452521000	8.385525000	15.857454000
8	128.546137000	9.076591000	15.387674000
1	130.638331000	6.141094000	17.870197000
1	128.140044000	6.907670000	16.618139000
7	130.752049000	8.510306000	15.496098000
6	131.139057000	9.445355000	14.447728000
6	130.524341000	9.113583000	13.082013000
8	130.512657000	9.997665000	12.207899000
6	132.664596000	9.574594000	14.331700000
6	133.342193000	10.233730000	15.541616000
16	132.707215000	11.888544000	16.021503000
6	133.103361000	12.865687000	14.527790000
1	131.453098000	7.864672000	15.853695000
1	130.725801000	10.427635000	14.701179000
1	133.112335000	8.582760000	14.188293000
1	132.874450000	10.142410000	13.420301000
1	134.419264000	10.306494000	15.353537000
1	133.217665000	9.616845000	16.437071000
1	132.792668000	13.892465000	14.739053000
1	132.557027000	12.508176000	13.650441000
1	134.178472000	12.863206000	14.324016000
7	129.964941000	7.904515000	12.901978000
6	129.251698000	7.582352000	11.672904000
6	128.116074000	8.577217000	11.397036000
8	127.740293000	8.768511000	10.225813000
6	128.720029000	6.142913000	11.717444000
1	130.007954000	7.197068000	13.632510000
1	129.942442000	7.679397000	10.831147000
1	128.260029000	5.889982000	10.758539000
1	127.986648000	6.011640000	12.519588000
1	129.549959000	5.453058000	11.895116000
7	127.534514000	9.219716000	12.427803000
6	126.504390000	10.213895000	12.161755000
6	126.999000000	11.492334000	11.460268000
8	126.200728000	12.192510000	10.831041000
1	127.873916000	9.087496000	13.379964000
1	126.043230000	10.493921000	13.113308000
1	125.734573000	9.789953000	11.512452000
7	128.306242000	11.811169000	11.611281000
6	128.910732000	12.919090000	10.895490000
6	129.740569000	12.571408000	9.646104000
8	130.046787000	13.473581000	8.870852000
1	128.923177000	11.172725000	12.106170000
1	128.113020000	13.586710000	10.567179000
7	130.109896000	11.267256000	9.485914000
6	130.984027000	10.856380000	8.393186000
6	130.319160000	9.934735000	7.346529000
8	130.891983000	9.744072000	6.271912000
6	132.256302000	10.158436000	8.915359000
6	133.149105000	11.023190000	9.757116000
6	133.223085000	12.391906000	9.866224000
7	134.119287000	10.446274000	10.562853000
6	134.764751000	11.446951000	11.136957000
7	134.254797000	12.642614000	10.750424000
1	130.060041000	10.658009000	10.298472000
1	131.251061000	11.764740000	7.847530000
1	132.810473000	9.790798000	8.043711000
1	131.977929000	9.271491000	9.497821000
1	132.635770000	13.182385000	9.423403000
1	135.593571000	11.362940000	11.825175000
1	134.607819000	13.548217000	11.034163000
7	129.146676000	9.350122000	7.697490000
6	128.485533000	8.383326000	6.825324000
6	127.033081000	8.737074000	6.464446000
8	126.598110000	8.436867000	5.343305000
6	128.571357000	6.930968000	7.351739000
8	127.667999000	6.631148000	8.399430000
1	128.776900000	9.525256000	8.624563000
1	129.038219000	8.407410000	5.885360000
1	129.618886000	6.743762000	7.638599000
1	128.324046000	6.257809000	6.526899000
1	127.741732000	7.326965000	9.080066000
7	126.271557000	9.355785000	7.391684000
6	124.865992000	9.664820000	7.159084000
6	124.435708000	10.841217000	8.026674000
8	124.559531000	10.480636000	9.394571000
1	126.572822000	9.407773000	8.358826000
1	124.240149000	8.791535000	7.389374000
1	125.056916000	11.716653000	7.790436000
1	123.391241000	11.094700000	7.785235000
1	124.954535000	11.227124000	9.888678000
7	132.621483000	16.899860000	4.964040000
6	133.237905000	17.115097000	6.294264000
6	134.696291000	16.613132000	6.369254000
8	135.339780000	16.743007000	7.416760000
6	132.330491000	16.355568000	7.294816000
6	131.598471000	15.334676000	6.410790000
6	131.366813000	16.131475000	5.124114000
1	133.288224000	18.179892000	6.556040000
1	131.607581000	17.048569000	7.741490000
1	132.910544000	15.912370000	8.107793000
1	132.248206000	14.476151000	6.203761000
1	130.679914000	14.956177000	6.864380000
1	131.192240000	15.495156000	4.249128000
1	130.489823000	16.789323000	5.239986000
7	135.159342000	16.046965000	5.237986000
6	136.513851000	15.547162000	5.045809000
6	136.578919000	14.128628000	4.436559000
6	136.367486000	12.997986000	5.466030000
6	137.940834000	13.940057000	3.746797000
6	135.045253000	13.017181000	6.241877000
1	134.483244000	16.032812000	4.478057000
1	137.019112000	15.565390000	6.014511000
1	135.790789000	14.053791000	3.669241000
1	137.204206000	13.029172000	6.175370000
1	136.447476000	12.043445000	4.925584000
1	138.095695000	14.679922000	2.951975000
1	138.019070000	12.943532000	3.296739000
1	138.753801000	14.045909000	4.475322000
1	134.950207000	12.120745000	6.867433000
1	134.180608000	13.039533000	5.566724000
1	134.980543000	13.885628000	6.904193000
16	135.617820000	4.947673000	3.008546000
1	135.611739000	6.296331000	2.919974000
6	140.836231000	13.666784000	8.675703000
6	139.595964000	14.509281000	8.395971000
8	138.960217000	14.394213000	7.349140000
6	140.440744000	12.265137000	9.184974000
6	141.618034000	11.287073000	9.375555000
6	141.166569000	10.064318000	10.190114000
6	142.231202000	10.852775000	8.035479000
1	141.382045000	13.587342000	7.731212000
1	139.918204000	12.378868000	10.145804000
1	139.714992000	11.828751000	8.485546000
1	142.398292000	11.807389000	9.953379000
1	141.990896000	9.353724000	10.323955000
1	140.817958000	10.359553000	11.187512000
1	140.342252000	9.531563000	9.699668000
1	143.078713000	10.176026000	8.195591000
1	142.596263000	11.703555000	7.449266000
1	141.490457000	10.320627000	7.424480000
7	139.217161000	15.361283000	9.394816000
6	137.964129000	16.106222000	9.350046000
6	136.738398000	15.306891000	9.848204000
6	136.751736000	15.015508000	11.332180000
6	137.428758000	13.899220000	11.855317000
6	136.107201000	15.875761000	12.237539000
6	137.473391000	13.660275000	13.231522000
6	136.146523000	15.639294000	13.613699000
6	136.832995000	14.531597000	14.117028000
1	139.734250000	15.343357000	10.262314000
1	137.773063000	16.393569000	8.315518000
1	135.854401000	15.888728000	9.570077000
1	136.697642000	14.376558000	9.270244000
1	137.920252000	13.210480000	11.172484000
1	135.574628000	16.743845000	11.854884000
1	138.010999000	12.795141000	13.612103000

1	135.641588000	16.321371000	14.292839000
1	136.868589000	14.349080000	15.187607000
7	137.187486000	4.982406000	5.646119000
7	134.965953000	6.763064000	5.397737000
7	135.471951000	2.798786000	4.971654000
6	137.902914000	2.655159000	5.326671000
6	137.283293000	7.420409000	5.909899000
6	132.529972000	6.910793000	5.077911000
6	133.136939000	2.133657000	4.598249000
6	138.140489000	3.993437000	5.604013000
6	135.939398000	7.690094000	5.689677000
6	132.287200000	5.558174000	4.899423000
6	134.494377000	1.869040000	4.722624000
8	134.922751000	4.547900000	7.045296000
6	139.442279000	4.556081000	5.859731000
6	135.353562000	9.005628000	5.766018000
6	130.987157000	5.005385000	4.594124000
6	135.081988000	0.558355000	4.611762000
6	139.267864000	5.896537000	6.029907000
6	134.016820000	8.862636000	5.537916000
6	131.160747000	3.660879000	4.476364000
6	136.423824000	0.708237000	4.803265000
6	137.858601000	6.157251000	5.876238000
6	133.782857000	7.457413000	5.318500000
6	132.561379000	3.389668000	4.696567000
6	136.659252000	2.110080000	5.039078000
7	133.227893000	4.559437000	4.966360000
26	135.193359000	4.772981000	5.276733000
1	138.754637000	1.982637000	5.331368000
1	137.936604000	8.264096000	6.108955000
1	131.687655000	7.593513000	5.040078000
1	132.480200000	1.296159000	4.386210000
6	133.463502000	6.692190000	10.535385000
7	134.525648000	7.546118000	10.492420000
8	132.296250000	7.062746000	10.605685000
16	136.718998000	7.110924000	12.258973000
6	133.811411000	5.209059000	10.473725000
7	135.000235000	4.768620000	10.245388000
8	137.021137000	5.330615000	9.368927000
16	135.271131000	6.617447000	13.511116000
6	132.638970000	4.281112000	10.651221000
8	138.129502000	7.836312000	9.807328000
6	132.896114000	2.806940000	10.441899000
6	133.349808000	2.326525000	9.203254000
6	133.543956000	0.958856000	9.010491000
6	133.289092000	0.052230000	10.043499000
6	132.827492000	0.521962000	11.272959000
6	132.633253000	1.892621000	11.467949000
6	136.017406000	5.693315000	9.966631000
6	135.870711000	7.129331000	10.439518000
6	136.757080000	8.104100000	9.638551000
1	133.571102000	3.021300000	8.396229000
1	133.899376000	0.601604000	8.048103000
1	132.214812000	4.471801000	11.647173000
1	131.867654000	4.650160000	9.959463000
1	133.457853000	-1.009656000	9.889840000
1	132.617069000	-0.174841000	12.080383000
1	132.267076000	2.254032000	12.427303000
1	136.445296000	8.042044000	8.586350000
1	136.571368000	9.122108000	9.989843000
1	134.330839000	8.567817000	10.576711000
1	135.793644000	4.442694000	7.472185000
1	138.270345000	6.941566000	9.443229000
8	128.536576000	7.094880000	3.832251000
1	127.766082000	7.513323000	4.280910000
1	128.890290000	7.797236000	3.266769000
1	135.181714000	1.504116000	13.064714000
1	138.321693000	3.046158000	9.726823000
1	135.315308000	5.223775000	17.473941000
1	129.004706000	7.790856000	17.881364000
1	129.562737000	13.484331000	11.571710000
1	124.739327000	9.893483000	6.097950000
1	132.468873000	17.775794000	4.474451000
1	137.051831000	16.242726000	4.384985000
1	141.496517000	14.156806000	9.403925000

1	140.362247000	3.986121000	5.888935000
1	135.908617000	9.912268000	5.971037000
1	130.087854000	5.597193000	4.454528000
1	134.526480000	-0.348393000	4.408682000
1	140.015503000	6.655057000	6.224747000
1	133.231551000	9.607580000	5.536732000
1	130.421611000	2.905395000	4.241394000
1	137.195868000	-0.050615000	4.793846000
1	138.089693000	17.019185000	9.941943000
1	131.055483000	3.472518000	16.376279000

$^4TS2_B$:

6	136.506142000	2.410850000	13.816827000
6	137.823232000	2.385152000	13.038860000
8	138.755287000	1.658035000	13.386903000
6	136.725298000	2.369459000	15.339558000
6	137.416307000	3.645348000	15.842476000
6	135.400714000	2.129845000	16.077516000
1	135.894098000	3.278683000	13.538435000
1	137.394398000	1.523398000	15.537222000
1	137.584552000	3.601818000	16.925968000
1	136.806650000	4.535165000	15.628722000
1	138.390494000	3.782695000	15.362322000
1	135.563031000	2.054601000	17.160403000
1	134.695467000	2.954534000	15.897827000
1	134.917566000	1.202357000	15.747176000
7	137.868450000	3.184004000	11.938678000
6	139.031114000	3.289464000	11.065498000
6	138.764974000	2.861334000	9.614535000
6	138.413986000	1.402697000	9.367098000
6	138.659760000	0.377250000	10.287674000
6	137.847840000	1.046335000	8.130980000
6	138.354340000	-0.953269000	9.983842000
6	137.542343000	-0.274443000	7.812934000
6	137.800889000	-1.286155000	8.745506000
8	137.488298000	-2.573651000	8.392045000
1	137.088729000	3.802320000	11.751293000
1	139.820948000	2.692823000	11.526292000
1	139.669993000	3.115814000	9.041270000
1	137.970809000	3.495693000	9.198145000
1	139.073844000	0.598570000	11.266756000
1	137.646695000	1.820880000	7.393762000
1	138.552570000	-1.732559000	10.718808000
1	137.106855000	-0.532921000	6.852328000
1	137.747759000	-3.165043000	9.115683000
6	134.575064000	6.129480000	18.201355000
6	133.390045000	6.110347000	17.246561000
8	133.127626000	7.036621000	16.480837000
6	135.020030000	7.544153000	18.575920000
1	134.337619000	5.540200000	19.096632000
1	134.236772000	8.075849000	19.126595000
1	135.912774000	7.505591000	19.207244000
1	135.253092000	8.123755000	17.679308000
7	132.607345000	4.980105000	17.287456000
6	131.696920000	4.664749000	16.190590000
6	130.508091000	5.614419000	16.022337000
8	129.911681000	5.658380000	14.946989000
1	132.949405000	4.201838000	17.836389000
1	132.219640000	4.674707000	15.227945000
7	130.166155000	6.378913000	17.088590000
6	129.146567000	7.409798000	16.974297000
6	129.432425000	8.497305000	15.922797000
8	128.497856000	9.171502000	15.485367000
1	130.744454000	6.328261000	17.915401000
1	128.187030000	6.967937000	16.693024000
7	130.715286000	8.636351000	15.512114000
6	131.055187000	9.554620000	14.432025000
6	130.351723000	9.219044000	13.110535000
8	130.279405000	10.101363000	12.238369000
6	132.573963000	9.641187000	14.216488000
6	133.343774000	10.329791000	15.353628000
16	132.748108000	11.998215000	15.838164000
6	132.956385000	12.913457000	14.268733000
1	131.445164000	8.030546000	15.880799000
1	130.682474000	10.549955000	14.696973000

1	132.987836000	8.633038000	14.081852000
1	132.738274000	10.170737000	13.273226000
1	134.401850000	10.396849000	15.076517000
1	133.291102000	9.735883000	16.271178000
1	132.670516000	13.948650000	14.474020000
1	132.309412000	12.519529000	13.479674000
1	133.999532000	12.898888000	13.939949000
7	129.793411000	8.004129000	12.971425000
6	129.026357000	7.652752000	11.783328000
6	127.899630000	8.655732000	11.515550000
8	127.509558000	8.842887000	10.348021000
6	128.463191000	6.229566000	11.908822000
1	129.902824000	7.300454000	13.698083000
1	129.686386000	7.700622000	10.914476000
1	127.988736000	5.939557000	10.967841000
1	127.736403000	6.155602000	12.724415000
1	129.278099000	5.529474000	12.114629000
7	127.327143000	9.299872000	12.550411000
6	126.280903000	10.279378000	12.290522000
6	126.747746000	11.562707000	11.579333000
8	125.935574000	12.239741000	10.942881000
1	127.689703000	9.180678000	13.495495000
1	125.826737000	10.558263000	13.245921000
1	125.510401000	9.840430000	11.652424000
7	128.045306000	11.915923000	11.739871000
6	128.618080000	13.055631000	11.048223000
6	129.425301000	12.765859000	9.769994000
8	129.707277000	13.703508000	9.027232000
1	128.679953000	11.282540000	12.218623000
1	127.803923000	13.720973000	10.757390000
7	129.797577000	11.473096000	9.548672000
6	130.610369000	11.106052000	8.395108000
6	129.895748000	10.210026000	7.361359000
8	130.331516000	10.165058000	6.211530000
6	131.911561000	10.390594000	8.809511000
6	132.869775000	11.219746000	9.613150000
6	132.928843000	12.577416000	9.821546000
7	133.931748000	10.608396000	10.262846000
6	134.615031000	11.577680000	10.844657000
7	134.045226000	12.786401000	10.609750000
1	129.767926000	10.828112000	10.333747000
1	130.840948000	12.032298000	7.865656000
1	132.403738000	10.050163000	7.890095000
1	131.668620000	9.485057000	9.378852000
1	132.284786000	13.383159000	9.502092000
1	135.511270000	11.463587000	11.437320000
1	134.405612000	13.675038000	10.932587000
7	128.860067000	9.452139000	7.808189000
6	128.261199000	8.421500000	6.966330000
6	126.779763000	8.644816000	6.622138000
8	126.329884000	8.196685000	5.557278000
6	128.465456000	6.996584000	7.533475000
8	127.570480000	6.649646000	8.576024000
1	128.576394000	9.523324000	8.779132000
1	128.796333000	8.471105000	6.016530000
1	129.519156000	6.912774000	7.846234000
1	128.294998000	6.280815000	6.726124000
1	127.559438000	7.376315000	9.227878000
7	126.017111000	9.317102000	7.508228000
6	124.588706000	9.528402000	7.316977000
6	124.129015000	10.725858000	8.141718000
8	124.342394000	10.447551000	9.517879000
1	126.360143000	9.500579000	8.444520000
1	124.025155000	8.635579000	7.621305000
1	124.683235000	11.621294000	7.826961000
1	123.060482000	10.904312000	7.943171000
1	124.720095000	11.232865000	9.962323000
7	133.214012000	16.095384000	4.797533000
6	133.815047000	16.534892000	6.078722000
6	135.233431000	15.964836000	6.304290000
8	135.840796000	16.216623000	7.351126000
6	132.833063000	16.066162000	7.179029000
6	132.067256000	14.916541000	6.505628000
6	131.921665000	15.423344000	5.067099000
1	133.939076000	17.625008000	6.116131000

1	132.140501000	16.878365000	7.429890000
1	133.360488000	15.782712000	8.093075000
1	132.669625000	14.000113000	6.518527000
1	131.108918000	14.697185000	6.984217000
1	131.748620000	14.617468000	4.345328000
1	131.072547000	16.122165000	4.998647000
7	135.710895000	15.218181000	5.289451000
6	137.046783000	14.640507000	5.231878000
6	137.069743000	13.146168000	4.839676000
6	136.760563000	12.188950000	6.010295000
6	138.449244000	12.801971000	4.252622000
6	135.409223000	12.373729000	6.710016000
1	135.061045000	15.111381000	4.514110000
1	137.517605000	14.783544000	6.207486000
1	136.312363000	12.991692000	4.053411000
1	137.567423000	12.287554000	6.747256000
1	136.820811000	11.162014000	5.618639000
1	138.673033000	13.409340000	3.367179000
1	138.497810000	11.747715000	3.955807000
1	139.234020000	12.980384000	4.997511000
1	135.244293000	11.578734000	7.447980000
1	134.575126000	12.342740000	5.997200000
1	135.357932000	13.328555000	7.241348000
16	135.023185000	4.947634000	2.453853000
1	134.578489000	6.222791000	2.404447000
6	141.054134000	12.928967000	9.235445000
6	139.915305000	13.851747000	8.815340000
8	139.368882000	13.738587000	7.718724000
6	140.515287000	11.528957000	9.594762000
6	141.597335000	10.476929000	9.911585000
6	140.960402000	9.242202000	10.569649000
6	142.397589000	10.075390000	8.662971000
1	141.736740000	12.863610000	8.382558000
1	139.851627000	11.624492000	10.466656000
1	139.890240000	11.172133000	8.765777000
1	142.298732000	10.920995000	10.635835000
1	141.715643000	8.476131000	10.782822000
1	140.476506000	9.503864000	11.518877000
1	140.195618000	8.788822000	9.926863000
1	143.177645000	9.348449000	8.917743000
1	142.889055000	10.933070000	8.190124000
1	141.742002000	9.611724000	7.914611000
7	139.522387000	14.778606000	9.738698000
6	138.344784000	15.617474000	9.546306000
6	137.020303000	14.946681000	9.974410000
6	136.886069000	14.731108000	11.465023000
6	137.437993000	13.601442000	12.095199000
6	136.222886000	15.672901000	12.269687000
6	137.338959000	13.424093000	13.477519000
6	136.119856000	15.498819000	13.652178000
6	136.679245000	14.373729000	14.262439000
1	139.953534000	14.758701000	10.651830000
1	138.268712000	15.856598000	8.484806000
1	136.212629000	15.579453000	9.594168000
1	136.947749000	13.993952000	9.437935000
1	137.947345000	12.854099000	11.491082000
1	135.789485000	16.555469000	11.803674000
1	137.777438000	12.544155000	13.941829000
1	135.602382000	16.242730000	14.252431000
1	136.598961000	14.236387000	15.337215000
7	136.663631000	5.100975000	5.058922000
7	134.187957000	6.515807000	4.956017000
7	135.296776000	2.705193000	4.310088000
6	137.721431000	2.920315000	4.660810000
6	136.391438000	7.513848000	5.408921000
6	131.749399000	6.286902000	4.718187000
6	133.086642000	1.705063000	3.913013000
6	137.753305000	4.269108000	4.983631000
6	135.014202000	7.574875000	5.246499000
6	131.717364000	4.921900000	4.475293000
6	134.470582000	1.650931000	4.009669000
8	134.529561000	4.239287000	6.475453000
6	138.958893000	5.015546000	5.244158000
6	134.233254000	8.777778000	5.394290000
6	130.508561000	4.187138000	4.183194000

6	135.251612000	0.453729000	3.831297000
6	138.586339000	6.309384000	5.449794000
6	132.927081000	8.434919000	5.210858000
6	130.885443000	2.898453000	3.959167000
6	136.554892000	0.795259000	4.039448000
6	137.151573000	6.356666000	5.318054000
6	132.907046000	7.017692000	4.944676000
6	132.320818000	2.843350000	4.109851000
6	136.574196000	2.203093000	4.347717000
7	132.803455000	4.084137000	4.440078000
26	134.725227000	4.592617000	4.727009000
1	138.666459000	2.387067000	4.639028000
1	136.912613000	8.443506000	5.616146000
1	130.799253000	6.812406000	4.714827000
1	132.566505000	0.787462000	3.657260000
6	133.027918000	6.944981000	10.424105000
7	134.148773000	7.680807000	10.166683000
8	131.886056000	7.384866000	10.470257000
16	136.000214000	6.461118000	11.899376000
6	133.369561000	5.470325000	10.677060000
7	134.291816000	4.974118000	9.738415000
8	136.236750000	5.375997000	8.563340000
16	134.150923000	5.624797000	12.478079000
6	132.168757000	4.506168000	10.742772000
8	137.789284000	7.378456000	9.664810000
6	132.744849000	3.156672000	10.399674000
6	133.482046000	3.115003000	9.164007000
6	134.369553000	2.029878000	8.915826000
6	134.475262000	0.997126000	9.832965000
6	133.692998000	1.013326000	11.001050000
6	132.849399000	2.103221000	11.288725000
6	135.355284000	5.692525000	9.379113000
6	135.420549000	7.046664000	10.139971000
6	136.513095000	7.962472000	9.567234000
1	133.158080000	3.680569000	8.294458000
1	134.912939000	2.014698000	7.978219000
1	131.680199000	4.529117000	11.720604000
1	131.435108000	4.846076000	10.001460000
1	135.145452000	0.166027000	9.636714000
1	133.766004000	0.192701000	11.709583000
1	132.315745000	2.138098000	12.235153000
1	136.232366000	8.181448000	8.526765000
1	136.530612000	8.901375000	10.127056000
1	134.087344000	8.719688000	10.205676000
1	135.301915000	4.573133000	6.980710000
1	137.735560000	6.576951000	9.105399000
8	128.299395000	6.791986000	4.128311000
1	127.531017000	7.223094000	4.571331000
1	128.468702000	7.347143000	3.352779000
1	135.943154000	1.519361000	13.505920000
1	139.370774000	4.333322000	11.057726000
1	135.395611000	5.599134000	17.696476000
1	129.021322000	7.884548000	17.951592000
1	129.273682000	13.608094000	11.731213000
1	124.403613000	9.690921000	6.251738000
1	133.104700000	16.870493000	4.151569000
1	137.639898000	15.207443000	4.499186000
1	141.617928000	13.344177000	10.081532000
1	139.954639000	4.590508000	5.249399000
1	134.647538000	9.752277000	5.618744000
1	129.525749000	4.641769000	4.143446000
1	134.840096000	-0.516235000	3.582537000
1	139.211875000	7.167605000	5.660326000
1	132.046169000	9.062445000	5.274310000
1	130.265227000	2.049268000	3.700734000
1	137.433925000	0.164811000	3.993530000
1	138.497336000	16.550598000	10.099336000
1	131.307057000	3.654989000	16.350833000

[4]IM2$_B$:

6	136.776985000	2.657392000	13.790138000
6	138.178867000	2.771069000	13.186973000
8	139.110264000	2.063621000	13.572560000
6	136.791308000	2.207761000	15.260160000
6	137.458032000	3.254291000	16.164486000
6	135.370864000	1.880634000	15.741732000
1	136.229965000	3.604136000	13.682921000
1	137.396187000	1.294307000	15.307120000
1	137.460332000	2.927926000	17.212010000
1	136.927379000	4.216376000	16.110423000
1	138.496448000	3.423725000	15.864837000
1	135.381915000	1.506159000	16.773321000
1	134.731383000	2.775105000	15.713507000
1	134.897056000	1.117893000	15.112400000
7	138.299157000	3.683081000	12.182673000
6	139.527118000	3.885789000	11.427936000
6	139.422825000	3.482997000	9.947253000
6	139.095029000	2.029365000	9.647793000
6	139.485540000	0.969892000	10.476253000
6	138.401249000	1.712177000	8.468388000
6	139.199902000	-0.354426000	10.136562000
6	138.109461000	0.396086000	8.116532000
6	138.516013000	-0.647967000	8.953875000
8	138.210268000	-1.928085000	8.571111000
1	137.498706000	4.257267000	11.944933000
1	140.304628000	3.319783000	11.944716000
1	140.380612000	3.753031000	9.475630000
1	138.666307000	4.114697000	9.464096000
1	139.985392000	1.163537000	11.419884000
1	138.079860000	2.513387000	7.806222000
1	139.507668000	-1.160002000	10.801967000
1	137.562140000	0.167191000	7.207227000
1	138.563932000	-2.542496000	9.233016000
6	134.547535000	5.923944000	18.067131000
6	133.424067000	5.879859000	17.041063000
8	133.169621000	6.811656000	16.279256000
6	134.962243000	7.347312000	18.441856000
1	134.253773000	5.351072000	18.956499000
1	134.141971000	7.887232000	18.926429000
1	135.809872000	7.324476000	19.133299000
1	135.254587000	7.910056000	17.551854000
7	132.683802000	4.720301000	17.016313000
6	131.839678000	4.402000000	15.868015000
6	130.642868000	5.334844000	15.664084000
8	130.114682000	5.424095000	14.555569000
1	133.029695000	3.939936000	17.560151000
1	132.411396000	4.433493000	14.934714000
7	130.216318000	6.026676000	16.749709000
6	129.162484000	7.023282000	16.657387000
6	129.449187000	8.210765000	15.721432000
8	128.507793000	8.912148000	15.344520000
1	130.747816000	5.934274000	17.604011000
1	128.241553000	6.568415000	16.281909000
7	130.730150000	8.395031000	15.325123000
6	131.056585000	9.382591000	14.303025000
6	130.350282000	9.118538000	12.965710000
8	130.279515000	10.046628000	12.142066000
6	132.573558000	9.507246000	14.094942000
6	133.324423000	10.175267000	15.256132000
16	132.704510000	11.825581000	15.772968000
6	132.886233000	12.770881000	14.217857000
1	131.467796000	7.775511000	15.653448000
1	130.668705000	10.354579000	14.627485000
1	133.009728000	8.513861000	13.926766000
1	132.729175000	10.072070000	13.170960000
1	134.384359000	10.260386000	14.991634000
1	133.268291000	9.559173000	16.158923000
1	132.592102000	13.799116000	14.445375000
1	132.234036000	12.384369000	13.429398000
1	133.925552000	12.773200000	13.876719000
7	129.775729000	7.919014000	12.769405000
6	128.972883000	7.641679000	11.585217000
6	127.867539000	8.684356000	11.388845000
8	127.461093000	8.937527000	10.239504000
6	128.368782000	6.231693000	11.664474000
1	129.914335000	7.167923000	13.441477000
1	129.612637000	7.708444000	10.702368000
1	127.856481000	5.999407000	10.727227000
1	127.663547000	6.144817000	12.497828000
1	129.165124000	5.496794000	11.811813000

7	127.326960000	9.289778000	12.463061000
6	126.298976000	10.302917000	12.264577000
6	126.779500000	11.596705000	11.581242000
8	125.968698000	12.311536000	10.985591000
1	127.695957000	9.111989000	13.396736000
1	125.878900000	10.561077000	13.241139000
1	125.499871000	9.905089000	11.634597000
7	128.088207000	11.914772000	11.720783000
6	128.672986000	13.060553000	11.049571000
6	129.454055000	12.791001000	9.750428000
8	129.742380000	13.744794000	9.030789000
1	128.717890000	11.254845000	12.169381000
1	127.867964000	13.749660000	10.790241000
7	129.798827000	11.498925000	9.484775000
6	130.580249000	11.151438000	8.302936000
6	129.823156000	10.308981000	7.253497000
8	130.237534000	10.289051000	6.095184000
6	131.873685000	10.394549000	8.665320000
6	132.865949000	11.176892000	9.474534000
6	132.941829000	12.522045000	9.748663000
7	133.940759000	10.527361000	10.063682000
6	134.646501000	11.461957000	10.674977000
7	134.080378000	12.684254000	10.515857000
1	129.768528000	10.830655000	10.250102000
1	130.821525000	12.089922000	7.800390000
1	132.339122000	10.073863000	7.725237000
1	131.624687000	9.476019000	9.210899000
1	132.297198000	13.347411000	9.485510000
1	135.555784000	11.312132000	11.239082000
1	134.454879000	13.553006000	10.875092000
7	128.772516000	9.568676000	7.694521000
6	128.122409000	8.588805000	6.830362000
6	126.647493000	8.882460000	6.511443000
8	126.167253000	8.487376000	5.438911000
6	128.270861000	7.140874000	7.355002000
8	127.372592000	6.804543000	8.398530000
1	128.506803000	9.618182000	8.671874000
1	128.648869000	8.643766000	5.876149000
1	129.323078000	7.000444000	7.651072000
1	128.059150000	6.455920000	6.531170000
1	127.407729000	7.508482000	9.073743000
7	125.921976000	9.554233000	7.428451000
6	124.498112000	9.815256000	7.266589000
6	124.083441000	10.977576000	8.162314000
8	124.312280000	10.619942000	9.517401000
1	126.281465000	9.689998000	8.366601000
1	123.912879000	8.922827000	7.528163000
1	124.655916000	11.874440000	7.886448000
1	123.016960000	11.193678000	7.992098000
1	124.716176000	11.371820000	9.995450000
7	133.305280000	16.144826000	4.827158000
6	133.921069000	16.574884000	6.104566000
6	135.332072000	15.982929000	6.320668000
8	135.951567000	16.226441000	7.362257000
6	132.939209000	16.120686000	7.211055000
6	132.155131000	14.980191000	6.543220000
6	132.006833000	15.487874000	5.105226000
1	134.061956000	17.662925000	6.141188000
1	132.258689000	16.942323000	7.464248000
1	133.467683000	15.831919000	8.122822000
1	132.746393000	14.056549000	6.553249000
1	131.196643000	14.772628000	7.026977000
1	131.819809000	14.683653000	4.385115000
1	131.165643000	16.196554000	5.041478000
7	135.789935000	15.227040000	5.303479000
6	137.115905000	14.627900000	5.238944000
6	137.114132000	13.146205000	4.801729000
6	136.771865000	12.160281000	5.938905000
6	138.493725000	12.792787000	4.220365000
6	135.411257000	12.344009000	6.620753000
1	135.134615000	15.132993000	4.531086000
1	137.580844000	14.732273000	6.222344000
1	136.363758000	13.030743000	4.002061000
1	137.567693000	12.225679000	6.691345000
1	136.823988000	11.144091000	5.518893000
1	138.741267000	13.423988000	3.358244000
1	138.524627000	11.747963000	3.889731000
1	139.272553000	12.930195000	4.980083000
1	135.222007000	11.530148000	7.331494000
1	134.589445000	12.346430000	5.893052000
1	135.362894000	13.283584000	7.178832000
16	134.688824000	4.739653000	2.564623000
1	134.456012000	6.064565000	2.435131000
6	141.074398000	12.760965000	9.249560000
6	139.944665000	13.697889000	8.835701000
8	139.389426000	13.590055000	7.742811000
6	140.525707000	11.356753000	9.574624000
6	141.599949000	10.294001000	9.882136000
6	140.951202000	9.048714000	10.507978000
6	142.413041000	9.914546000	8.634907000
1	141.766091000	12.709042000	8.402873000
1	139.852824000	11.437768000	10.440957000
1	139.908397000	11.020364000	8.731505000
1	142.294604000	10.720137000	10.623386000
1	141.701697000	8.276356000	10.715515000
1	140.455775000	9.293232000	11.455755000
1	140.193538000	8.610579000	9.846368000
1	143.187604000	9.179734000	8.883801000
1	142.912976000	10.779735000	8.185247000
1	141.764561000	9.468726000	7.869675000
7	139.570785000	14.632246000	9.758635000
6	138.410319000	15.495054000	9.565234000
6	137.068008000	14.838615000	9.957909000
6	136.903319000	14.596623000	11.441392000
6	137.433879000	13.451262000	12.061713000
6	136.229951000	15.528105000	12.249708000
6	137.304159000	13.248662000	13.437910000
6	136.096447000	15.328851000	13.626208000
6	136.634593000	14.188206000	14.226600000
1	140.007457000	14.610122000	10.668959000
1	138.354236000	15.759328000	8.508309000
1	136.277897000	15.492515000	9.576589000
1	136.989512000	13.898104000	9.401099000
1	137.951366000	12.711965000	11.454625000
1	135.812627000	16.422369000	11.791425000
1	137.725941000	12.356666000	13.894573000
1	135.571597000	16.065151000	14.229406000
1	136.530316000	14.031153000	15.296602000
7	136.341114000	5.001480000	5.149864000
7	133.945762000	6.543689000	4.933971000
7	134.836114000	2.639003000	4.579336000
6	137.277407000	2.749063000	4.861387000
6	136.196790000	7.440972000	5.369888000
6	131.502902000	6.439823000	4.670472000
6	132.568797000	1.731935000	4.312314000
6	137.386053000	4.110968000	5.104570000
6	134.827631000	7.568895000	5.181247000
6	131.392438000	5.062654000	4.554283000
6	133.949837000	1.609466000	4.387493000
8	134.185442000	4.374682000	6.631680000
6	138.631101000	4.805968000	5.315342000
6	134.116497000	8.821875000	5.240276000
6	130.143834000	4.375276000	4.320181000
6	134.663202000	0.362313000	4.285514000
6	138.327261000	6.126055000	5.464876000
6	132.796824000	8.545101000	5.043716000
6	130.442891000	3.050139000	4.237264000
6	135.987674000	0.650397000	4.427086000
6	136.895896000	6.241504000	5.342227000
6	132.698194000	7.117414000	4.863364000
6	131.871378000	2.925243000	4.407787000
6	136.087447000	2.074675000	4.623565000
7	132.426546000	4.162688000	4.619521000
26	134.369790000	4.580200000	4.853404000
1	138.192709000	2.165943000	4.856588000
1	136.766298000	8.351637000	5.529175000
1	130.586390000	7.017522000	4.602235000
1	131.994300000	0.826403000	4.145460000
6	132.989558000	6.929045000	10.377466000
7	134.126397000	7.608052000	10.038756000

8	131.858114000	7.393380000	10.353401000
16	135.936816000	6.565495000	11.941894000
6	133.294252000	5.507931000	10.853310000
7	134.236219000	4.845774000	9.988453000
8	136.152977000	5.083703000	8.743409000
16	134.051635000	5.848157000	12.595890000
6	132.092877000	4.553698000	10.955940000
8	137.766452000	7.187777000	9.614842000
6	132.717056000	3.222169000	10.650248000
6	133.851476000	3.430130000	9.678879000
6	134.920268000	2.379841000	9.770092000
6	134.645983000	1.182587000	10.373175000
6	133.427508000	0.951234000	11.067528000
6	132.510389000	2.017419000	11.257718000
6	135.303560000	5.530570000	9.523532000
6	135.383745000	6.949852000	10.106546000
6	136.497139000	7.776598000	9.448926000
1	133.492831000	3.458460000	8.629995000
1	135.850726000	2.544453000	9.241227000
1	131.594302000	4.601246000	11.928631000
1	131.365092000	4.871538000	10.195395000
1	135.390195000	0.391498000	10.329526000
1	133.238618000	-0.014012000	11.526495000
1	131.672677000	1.891238000	11.940635000
1	136.228072000	7.892783000	8.389115000
1	136.527651000	8.767669000	9.908198000
1	134.085745000	8.650024000	9.999163000
1	135.061947000	4.513755000	7.042603000
1	137.725330000	6.353585000	9.108827000
8	128.056540000	7.025574000	3.966423000
1	127.313207000	7.488848000	4.419585000
1	128.232532000	7.565402000	3.181762000
1	136.229173000	1.922063000	13.183631000
1	139.804175000	4.946721000	11.477174000
1	135.400740000	5.385867000	17.629323000
1	128.956159000	7.405678000	17.660864000
1	129.350361000	13.582109000	11.735729000
1	124.304746000	10.042279000	6.214593000
1	133.200257000	16.922425000	4.183493000
1	137.726571000	15.205468000	4.529145000
1	141.631632000	13.156469000	10.109273000
1	139.604057000	4.331314000	5.329629000
1	134.583066000	9.782089000	5.419315000
1	129.191703000	4.882085000	4.216028000
1	134.195348000	-0.601243000	4.128668000
1	138.999111000	6.959739000	5.625638000
1	131.951727000	9.222415000	5.058087000
1	129.774323000	2.217394000	4.058315000
1	136.831744000	-0.027509000	4.408130000
1	138.570692000	16.412939000	10.140766000
1	131.461911000	3.382942000	15.996174000

^{4}TS3$_B$:

6	138.617425000	4.088773000	13.693675000
6	139.847063000	3.812139000	12.829247000
8	140.691301000	2.977524000	13.158936000
6	138.934396000	4.070209000	15.199626000
6	139.867749000	5.224710000	15.592277000
6	137.642603000	4.094158000	16.027737000
1	138.148485000	5.040685000	13.411597000
1	139.462813000	3.129365000	15.396062000
1	140.104704000	5.192023000	16.662672000
1	139.402130000	6.198130000	15.384162000
1	140.811640000	5.172784000	15.039790000
1	137.863092000	4.049140000	17.101496000
1	137.070367000	5.013582000	15.843728000
1	136.993256000	3.245042000	15.781745000
7	139.925715000	4.537280000	11.678294000
6	140.989829000	4.333751000	10.706759000
6	140.484113000	4.054519000	9.284314000
6	139.582403000	2.845142000	9.099687000
6	139.595489000	1.741624000	9.961795000
6	138.716568000	2.801289000	7.995379000
6	138.774463000	0.635752000	9.726789000
6	137.897527000	1.703616000	7.742483000
6	137.923797000	0.614227000	8.618756000
8	137.086367000	-0.440136000	8.347644000
1	139.149329000	5.141125000	11.424576000
1	141.599853000	3.511653000	11.087295000
1	141.374215000	3.954896000	8.644182000
1	139.961145000	4.946639000	8.911623000
1	140.231736000	1.736322000	10.842253000
1	138.683690000	3.642420000	7.306731000
1	138.793733000	-0.207198000	10.416434000
1	137.228189000	1.692881000	6.887757000
1	137.197876000	-1.110203000	9.039894000
6	132.158924000	6.370545000	19.856283000
6	131.250717000	6.378774000	18.636317000
8	131.471179000	7.092091000	17.656247000
6	133.629287000	6.162427000	19.464342000
1	132.046869000	7.346646000	20.346181000
1	133.935380000	6.912267000	18.730462000
1	134.272517000	6.251603000	20.344703000
1	133.788230000	5.171685000	19.025620000
7	130.139914000	5.576708000	18.689820000
6	129.346215000	5.319814000	17.492038000
6	128.652986000	6.548809000	16.897147000
8	128.345517000	6.568021000	15.707342000
1	130.072101000	4.912292000	19.448842000
1	129.963901000	4.908087000	16.687452000
7	128.400515000	7.582274000	17.739368000
6	127.832037000	8.824318000	17.243352000
6	128.655608000	9.529927000	16.153960000
8	128.095834000	10.338692000	15.409532000
1	128.724098000	7.516984000	18.693968000
1	126.845875000	8.647809000	16.805406000
7	129.969441000	9.219330000	16.060072000
6	130.786623000	9.793955000	14.996012000
6	130.267053000	9.449619000	13.592051000
8	130.570886000	10.188463000	12.642281000
6	132.260196000	9.376720000	15.124134000
6	132.974120000	9.899035000	16.381216000
16	132.941188000	11.714672000	16.644854000
6	133.996165000	12.286888000	15.266298000
1	130.373578000	8.496120000	16.651074000
1	130.723383000	10.884728000	15.060331000
1	132.332489000	8.280709000	15.123858000
1	132.772522000	9.724571000	14.221270000
1	134.018228000	9.565581000	16.363489000
1	132.524673000	9.472293000	17.282484000
1	134.090909000	13.370956000	15.368967000
1	133.552181000	12.065657000	14.291969000
1	134.996234000	11.845986000	15.327360000
7	129.482398000	8.363779000	13.448653000
6	128.907223000	8.025379000	12.150819000
6	128.028245000	9.155646000	11.598683000
8	127.851762000	9.248910000	10.369377000
6	128.128184000	6.705822000	12.231443000
1	129.227147000	7.797302000	14.254175000
1	129.720880000	7.905161000	11.431291000
1	127.766218000	6.432895000	11.236757000
1	127.281125000	6.778941000	12.921237000
1	128.794821000	5.912418000	12.582728000
7	127.452909000	10.023694000	12.451480000
6	126.691345000	11.149382000	11.929666000
6	127.535201000	12.237248000	11.241845000
8	127.011791000	12.999737000	10.424406000
1	127.655354000	9.983279000	13.449494000
1	126.145744000	11.607962000	12.759718000
1	125.969380000	10.801862000	11.187315000
7	128.832396000	12.332358000	11.618348000
6	129.750073000	13.252855000	10.975149000
6	130.613208000	12.684806000	9.835269000
8	131.194015000	13.464018000	9.084299000
1	129.222744000	11.640055000	12.250660000
1	129.172479000	14.073830000	10.548070000
7	130.696064000	11.325777000	9.737064000
6	131.543804000	10.699954000	8.729105000
6	130.786460000	9.891112000	7.656020000
8	131.353446000	9.634082000	6.593172000

6	132.582477000	9.748540000	9.355711000
6	133.539135000	10.387211000	10.317605000
6	133.900379000	11.703375000	10.475698000
7	134.249787000	9.601871000	11.212520000
6	135.025314000	10.430510000	11.888948000
7	134.843866000	11.713458000	11.484112000
1	130.441008000	10.778121000	10.554140000
1	132.042861000	11.510425000	8.194175000
1	133.134560000	9.287014000	8.527557000
1	132.059567000	8.933303000	9.869814000
1	133.568496000	12.605973000	9.984558000
1	135.725742000	10.155693000	12.665483000
1	135.353460000	12.521133000	11.824465000
7	129.551936000	9.429652000	7.982558000
6	128.857647000	8.469306000	7.130156000
6	127.502932000	8.948878000	6.586055000
8	127.102888000	8.525635000	5.491894000
6	128.701821000	7.079823000	7.795952000
8	127.626227000	6.983153000	8.711684000
1	129.178046000	9.634795000	8.902569000
1	129.497736000	8.335394000	6.256646000
1	129.666863000	6.830739000	8.267006000
1	128.505831000	6.348840000	7.007848000
1	127.700664000	7.719336000	9.348539000
7	126.787524000	9.813093000	7.335291000
6	125.466003000	10.282987000	6.943135000
6	125.180637000	11.637463000	7.581981000
8	125.161836000	11.493927000	8.995250000
1	127.049945000	9.995407000	8.297266000
1	124.695762000	9.563220000	7.253255000
1	125.946836000	12.359390000	7.266894000
1	124.206152000	12.001446000	7.220728000
1	125.685465000	12.209944000	9.408605000
7	132.804520000	16.732639000	5.227611000
6	133.414450000	16.851578000	6.571186000
6	134.702106000	16.019507000	6.717011000
8	135.414721000	16.148950000	7.719076000
6	132.304664000	16.393683000	7.562602000
6	131.245351000	15.729239000	6.667250000
6	131.351041000	16.534313000	5.370655000
1	133.716093000	17.881729000	6.804636000
1	131.879579000	17.271826000	8.062515000
1	132.683541000	15.724764000	8.337658000
1	131.500196000	14.680315000	6.488162000
1	130.244609000	15.754735000	7.106887000
1	130.949895000	16.006396000	4.497885000
1	130.802225000	17.487382000	5.468845000
7	134.954509000	15.175822000	5.697120000
6	136.149066000	14.345404000	5.611501000
6	135.991257000	13.115366000	4.697801000
6	135.104264000	11.997070000	5.287821000
6	137.393292000	12.561491000	4.387616000
6	133.608375000	12.308620000	5.427527000
1	134.291347000	15.230413000	4.929099000
1	136.424216000	14.026019000	6.620591000
1	135.538002000	13.446504000	3.748087000
1	135.517269000	11.710431000	6.265985000
1	135.211431000	11.115273000	4.640587000
1	138.016755000	13.309950000	3.883260000
1	137.331304000	11.682575000	3.735794000
1	137.901419000	12.268573000	5.314272000
1	133.048790000	11.414095000	5.722450000
1	133.187390000	12.656043000	4.474367000
1	133.421000000	13.085966000	6.174427000
16	135.068418000	4.396226000	1.936208000
1	133.737025000	4.481240000	1.719199000
6	140.271775000	12.046851000	8.874385000
6	139.079498000	12.981621000	8.692143000
8	138.148540000	12.693083000	7.940509000
6	139.829282000	10.714728000	9.508484000
6	140.947556000	9.664592000	9.661119000
6	140.470270000	8.502933000	10.546935000
6	141.446117000	9.141544000	8.305186000
1	140.688181000	11.867583000	7.877244000
1	139.408282000	10.925323000	10.502997000
1	139.010036000	10.301792000	8.906394000
1	141.795903000	10.146881000	10.172053000
1	141.267349000	7.764064000	10.691994000
1	140.164311000	8.858159000	11.538804000
1	139.612728000	7.982651000	10.101001000
1	142.242066000	8.400011000	8.441488000
1	141.848582000	9.941018000	7.673350000
1	140.630666000	8.657660000	7.751928000
7	139.097254000	14.129645000	9.428390000
6	137.992299000	15.085516000	9.415465000
6	136.752057000	14.627398000	10.214541000
6	136.955879000	14.572067000	11.712278000
6	137.607252000	13.487036000	12.328020000
6	136.515771000	15.622875000	12.534256000
6	137.816765000	13.458532000	13.710597000
6	136.722910000	15.598554000	13.914291000
6	137.375866000	14.515644000	14.509120000
1	139.853985000	14.272399000	10.080748000
1	137.672071000	15.250808000	8.385452000
1	135.944699000	15.320103000	9.958136000
1	136.464817000	13.645986000	9.820065000
1	137.956807000	12.660167000	11.714167000
1	136.005607000	16.469156000	12.079976000
1	138.330061000	12.612466000	14.161425000
1	136.373369000	16.426020000	14.526472000
1	137.539864000	14.496856000	15.583178000
7	136.960354000	3.930516000	4.269172000
7	134.735101000	5.598389000	4.875416000
7	135.191614000	1.754219000	3.690380000
6	137.641413000	1.646678000	3.653159000
6	137.086809000	6.289330000	4.955510000
6	132.279703000	5.719510000	4.885918000
6	132.837351000	1.135111000	3.404490000
6	137.902277000	2.970687000	3.983026000
6	135.727576000	6.518146000	5.108501000
6	132.023599000	4.436176000	4.416778000
6	134.198230000	0.860755000	3.394517000
8	134.961876000	3.098457000	6.087427000
6	139.221604000	3.535756000	4.089985000
6	135.146685000	7.774512000	5.518042000
6	130.698609000	3.914089000	4.185385000
6	134.773192000	-0.416970000	3.043540000
6	139.066713000	4.844241000	4.444024000
6	133.794034000	7.610833000	5.513274000
6	130.857951000	2.626465000	3.767054000
6	136.124433000	-0.275583000	3.124930000
6	137.651591000	5.082151000	4.561765000
6	133.546095000	6.251002000	5.093537000
6	132.274586000	2.358899000	3.741010000
6	136.377372000	1.090287000	3.519792000
7	132.968077000	3.481435000	4.123402000
26	134.963870000	3.691181000	4.235777000
1	138.494896000	1.001753000	3.469599000
1	137.761310000	7.114444000	5.161599000
1	131.419939000	6.356367000	5.070648000
1	132.160652000	0.331608000	3.130821000
6	132.629371000	6.162587000	10.573569000
7	133.866457000	6.721818000	10.711830000
8	131.564912000	6.759942000	10.684876000
16	135.224582000	4.630077000	11.996376000
6	132.715267000	4.659747000	10.285303000
7	133.712065000	4.379795000	9.277493000
8	135.822951000	4.863042000	8.509264000
16	133.216042000	3.933530000	11.984060000
6	131.428722000	3.992394000	9.764213000
8	137.477647000	5.957452000	10.425926000
6	131.960429000	2.909267000	8.866239000
6	133.281256000	3.362350000	8.292286000
6	134.246508000	2.244548000	7.989909000
6	133.703677000	0.978551000	7.733935000
6	132.356297000	0.711944000	7.943241000
6	131.504881000	1.661554000	8.615495000
6	134.909315000	5.003501000	9.328209000
6	135.032808000	5.907234000	10.564079000
6	136.313228000	6.755092000	10.499329000

1	133.098156000	3.885840000	7.338091000
1	135.297377000	2.356767000	8.212845000
1	130.787045000	3.622357000	10.568396000
1	130.857827000	4.758799000	9.221179000
1	134.377280000	0.181904000	7.433358000
1	131.967075000	-0.281064000	7.738300000
1	130.538179000	1.334682000	8.990801000
1	136.219763000	7.417641000	9.627888000
1	136.390029000	7.373718000	11.395867000
1	133.962029000	7.709681000	11.018218000
1	135.440012000	3.797760000	6.576538000
1	137.361315000	5.403005000	9.625751000
8	128.936902000	6.719537000	4.388519000
1	128.208398000	7.307925000	4.697064000
1	129.332962000	7.196575000	3.644152000
1	137.885352000	3.301934000	13.464491000
1	141.636781000	5.221943000	10.672555000
1	131.837401000	5.612209000	20.580202000
1	127.704907000	9.509472000	18.086481000
1	130.429080000	13.673178000	11.725458000
1	125.438128000	10.349845000	5.852436000
1	133.027694000	17.530353000	4.639728000
1	136.984927000	14.958725000	5.240059000
1	141.061683000	12.511797000	9.479503000
1	140.139676000	2.989106000	3.915828000
1	135.714446000	8.664774000	5.759066000
1	129.792591000	4.493679000	4.317121000
1	134.201250000	-1.294211000	2.768239000
1	139.831839000	5.590939000	4.615690000
1	133.019768000	8.330127000	5.753196000
1	130.094220000	1.906832000	3.499194000
1	136.893971000	-1.011722000	2.929572000
1	138.371605000	16.034366000	9.808001000
1	128.580999000	4.578711000	17.742545000

^4Pr_B:

6	139.320680000	4.978604000	13.206390000
6	140.090607000	4.367282000	12.033131000
8	141.049153000	3.613523000	12.211951000
6	140.208119000	5.255947000	14.431352000
6	141.243527000	6.352055000	14.141784000
6	139.351443000	5.610321000	15.654596000
1	138.801826000	5.894799000	12.891413000
1	140.753277000	4.327280000	14.639226000
1	141.886150000	6.525703000	15.013437000
1	140.753591000	7.304286000	13.894885000
1	141.887291000	6.070363000	13.302511000
1	139.978175000	5.772560000	16.539932000
1	138.775843000	6.530418000	15.483758000
1	138.637617000	4.811227000	15.890137000
7	139.612781000	4.692547000	10.801414000
6	140.175198000	4.173212000	9.562315000
6	139.195157000	3.339254000	8.722358000
6	138.712997000	2.019673000	9.303282000
6	139.346197000	1.365331000	10.365645000
6	137.597610000	1.393884000	8.719252000
6	138.884608000	0.128922000	10.827311000
6	137.132592000	0.160753000	9.167338000
6	137.780471000	-0.479567000	10.229241000
8	137.286246000	-1.691694000	10.640990000
1	138.826454000	5.331119000	10.729845000
1	141.059043000	3.594653000	9.838896000
1	139.683936000	3.153248000	7.754077000
1	138.318667000	3.955946000	8.477771000
1	140.197101000	1.815786000	10.868082000
1	137.078823000	1.878796000	7.894254000
1	139.390297000	-0.357721000	11.660612000
1	136.272920000	-0.317259000	8.707147000
1	137.842035000	-2.018880000	11.365259000
6	132.348336000	5.946791000	19.542876000
6	131.372446000	6.049794000	18.378860000
8	131.504432000	6.856142000	17.458672000
6	133.070194000	7.262985000	19.838360000
1	131.825427000	5.575858000	20.433858000
1	132.366769000	8.041455000	20.152227000
1	133.800803000	7.122619000	20.640525000
1	133.595304000	7.622187000	18.949908000
7	130.311359000	5.177512000	18.409785000
6	129.515534000	4.927040000	17.211995000
6	128.698204000	6.121795000	16.713331000
8	128.334310000	6.171964000	15.540080000
1	130.318507000	4.458540000	19.121467000
1	130.147119000	4.626447000	16.369596000
7	128.408307000	7.089471000	17.618298000
6	127.730643000	8.309713000	17.214840000
6	128.450235000	9.126077000	16.128909000
8	127.800834000	9.925788000	15.450618000
1	128.786823000	7.002980000	18.550849000
1	126.739552000	8.084446000	16.811759000
7	129.776739000	8.914537000	15.961220000
6	130.498385000	9.594434000	14.890766000
6	129.934206000	9.282986000	13.497682000
8	130.147076000	10.086464000	12.575175000
6	132.002232000	9.280540000	14.925836000
6	132.746749000	9.816576000	16.158806000
16	132.552302000	11.605998000	16.516569000
6	133.333283000	12.352528000	15.041956000
1	130.260740000	8.205490000	16.507614000
1	130.362411000	10.673491000	15.013732000
1	132.152675000	8.193377000	14.882573000
1	132.438429000	9.690717000	14.009348000
1	133.813970000	9.588786000	16.056205000
1	132.401835000	9.311400000	17.065672000
1	133.303184000	13.435305000	15.189795000
1	132.790032000	12.109339000	14.124704000
1	134.379361000	12.045919000	14.946841000
7	129.208510000	8.161006000	13.336435000
6	128.600681000	7.840062000	12.050672000
6	127.643289000	8.941282000	11.576782000
8	127.421207000	9.076599000	10.359014000
6	127.894562000	6.479107000	12.114061000
1	129.041944000	7.534533000	14.120559000
1	129.391148000	7.790230000	11.297903000
1	127.510453000	6.217568000	11.124663000
1	127.071628000	6.485488000	12.836050000
1	128.614872000	5.713152000	12.417125000
7	127.049707000	9.736117000	12.486596000
6	126.200409000	10.831868000	12.041978000
6	126.944827000	11.991406000	11.356679000
8	126.337605000	12.746927000	10.592738000
1	127.290708000	9.662474000	13.474225000
1	125.668360000	11.227588000	12.912153000
1	125.465813000	10.466791000	11.320629000
7	128.251188000	12.151539000	11.675315000
6	129.083098000	13.144289000	11.022192000
6	129.975558000	12.650379000	9.869833000
8	130.478106000	13.476897000	9.113139000
1	128.707435000	11.468507000	12.272730000
1	128.433077000	13.914917000	10.605620000
7	130.173202000	11.303451000	9.770812000
6	131.060585000	10.744284000	8.758236000
6	130.360162000	9.884873000	7.683995000
8	130.968030000	9.622047000	6.647095000
6	132.174564000	9.884499000	9.386163000
6	133.135932000	10.626112000	10.267287000
6	133.392034000	11.971658000	10.379416000
7	133.982594000	9.923724000	11.113022000
6	134.734627000	10.829172000	11.713097000
7	134.409410000	12.082303000	11.307559000
1	129.971935000	10.733276000	10.588046000
1	131.493021000	11.588908000	8.217008000
1	132.719616000	9.410865000	8.560672000
1	131.721710000	9.071214000	9.966479000
1	132.942319000	12.831639000	9.905945000
1	135.516773000	10.636315000	12.433489000
1	134.878306000	12.931662000	11.597578000
7	129.120897000	9.410650000	7.975251000
6	128.459995000	8.455180000	7.091097000
6	127.079089000	8.895686000	6.580042000
8	126.704483000	8.528643000	5.456765000

6	128.380411000	7.030233000	7.689316000
8	127.351670000	6.847398000	8.644039000
1	128.713068000	9.626505000	8.877572000
1	129.095782000	8.388733000	6.206692000
1	129.375424000	6.791997000	8.101554000
1	128.177878000	6.331691000	6.873389000
1	127.394745000	7.580754000	9.287335000
7	126.312258000	9.662064000	7.383585000
6	124.968622000	10.083710000	7.009360000
6	124.609359000	11.384858000	7.717285000
8	124.592245000	11.163742000	9.120337000
1	126.554063000	9.789763000	8.360068000
1	124.236502000	9.308757000	7.275532000
1	125.338411000	12.161576000	7.447323000
1	123.619067000	11.716410000	7.367483000
1	125.055244000	11.897328000	9.573144000
7	133.414117000	16.474641000	5.251846000
6	133.948679000	16.527908000	6.632748000
6	135.299472000	15.795813000	6.781812000
8	135.883804000	15.796226000	7.871604000
6	132.853407000	15.881782000	7.520070000
6	132.027139000	15.051361000	6.526612000
6	132.035934000	15.939187000	5.279462000
1	134.144333000	17.557626000	6.959112000
1	132.225649000	16.664028000	7.962772000
1	133.283001000	15.300381000	8.338902000
1	132.530194000	14.101608000	6.311747000
1	131.025136000	14.823130000	6.895700000
1	131.820190000	15.388867000	4.356583000
1	131.281186000	16.737107000	5.378090000
7	135.739988000	15.187474000	5.663726000
6	137.007969000	14.481873000	5.538381000
6	136.887308000	13.071525000	4.918326000
6	136.441412000	11.984397000	5.919036000
6	138.242504000	12.676156000	4.307081000
6	135.086321000	12.195503000	6.604542000
1	135.123809000	15.302245000	4.862492000
1	137.457125000	14.419943000	6.532178000
1	136.144438000	13.121559000	4.105381000
1	137.223898000	11.888892000	6.683155000
1	136.415085000	11.029124000	5.374665000
1	138.553426000	13.382294000	3.527405000
1	138.193959000	11.678666000	3.855255000
1	139.018353000	12.660452000	5.082068000
1	134.817646000	11.315708000	7.202833000
1	134.283460000	12.350674000	5.873316000
1	135.104355000	13.056862000	7.279150000
16	135.058202000	4.821999000	1.746411000
1	133.760747000	4.672767000	1.395184000
6	140.936408000	12.175284000	9.202593000
6	139.819308000	13.173542000	8.919232000
8	139.162438000	13.121569000	7.880733000
6	140.347397000	10.788484000	9.530763000
6	141.385829000	9.666394000	9.729865000
6	140.713713000	8.430238000	10.348029000
6	142.100983000	9.299857000	8.420324000
1	141.548898000	12.120281000	8.297227000
1	139.743404000	10.874371000	10.446302000
1	139.654722000	10.507333000	8.727036000
1	142.144445000	10.027859000	10.441863000
1	141.429039000	7.610241000	10.478848000
1	140.291206000	8.660700000	11.334116000
1	139.893732000	8.061598000	9.718671000
1	142.840093000	8.507925000	8.588226000
1	142.628305000	10.154416000	7.981785000
1	141.382729000	8.934002000	7.675063000
7	139.573577000	14.093948000	9.898569000
6	138.435386000	15.004481000	9.844954000
6	137.120888000	14.398337000	10.387001000
6	137.128679000	14.135244000	11.876597000
6	137.682796000	12.957759000	12.411156000
6	136.603019000	15.079355000	12.775048000
6	137.723171000	12.739349000	13.790724000
6	136.637706000	14.863478000	14.154831000
6	137.200910000	13.693169000	14.668702000
1	140.091559000	14.024171000	10.762728000
1	138.265136000	15.279964000	8.803606000
1	136.319851000	15.092338000	10.114666000
1	136.934837000	13.473362000	9.829324000
1	138.081149000	12.205985000	11.733968000
1	136.166943000	15.995852000	12.383415000
1	138.165545000	11.825572000	14.180268000
1	136.226552000	15.611152000	14.828226000
1	137.233171000	13.525933000	15.741904000
7	136.740896000	4.147347000	4.431257000
7	134.510383000	5.828252000	4.906739000
7	134.996972000	2.056498000	3.624523000
6	137.439955000	1.881596000	3.781924000
6	136.857804000	6.469742000	5.226157000
6	132.063278000	6.004413000	4.768585000
6	132.659264000	1.467149000	3.172549000
6	137.693569000	3.180879000	4.196807000
6	135.495113000	6.717227000	5.271386000
6	131.825444000	4.741648000	4.242840000
6	134.014052000	1.176767000	3.240478000
8	134.936951000	2.990562000	6.861970000
6	139.007445000	3.704453000	4.458494000
6	134.909899000	7.960853000	5.706263000
6	130.512838000	4.244114000	3.920285000
6	134.589445000	-0.101826000	2.904880000
6	138.845291000	4.999862000	4.854220000
6	133.558434000	7.828682000	5.593097000
6	130.683113000	2.968004000	3.471311000
6	135.934288000	0.014265000	3.083187000
6	137.431753000	5.266208000	4.840052000
6	133.321140000	6.501198000	5.079720000
6	132.093537000	2.685156000	3.518698000
6	136.180344000	1.364357000	3.524069000
7	132.784634000	3.784814000	3.981577000
26	134.773694000	4.023452000	4.031521000
1	138.291859000	1.222641000	3.646964000
1	137.525278000	7.271182000	5.527376000
1	131.202134000	6.645425000	4.930998000
1	131.991338000	0.681097000	2.834226000
6	132.603860000	6.381176000	10.755934000
7	133.802132000	7.028267000	10.713086000
8	131.526762000	6.900059000	11.023138000
16	135.479954000	5.063237000	11.800875000
6	132.758476000	4.887566000	10.447725000
7	133.605634000	4.680664000	9.285645000
8	135.612839000	5.239410000	8.309297000
16	133.556792000	4.183741000	12.030185000
6	131.457664000	4.137855000	10.132660000
8	137.377422000	6.565748000	9.880916000
6	131.880565000	3.135148000	9.095115000
6	133.070638000	3.680337000	8.322301000
6	134.046800000	2.572759000	7.882016000
6	133.261203000	1.392057000	7.346557000
6	132.038670000	1.084675000	7.804130000
6	131.396787000	1.915978000	8.831207000
6	134.771255000	5.356486000	9.211770000
6	134.990356000	6.295240000	10.406382000
6	136.174497000	7.248543000	10.162861000
1	132.738941000	4.217105000	7.419194000
1	134.615310000	2.238279000	8.770303000
1	131.003473000	3.686601000	11.019031000
1	130.741170000	4.872714000	9.740058000
1	133.772312000	0.786230000	6.604739000
1	131.515056000	0.203460000	7.444348000
1	130.537420000	1.526029000	9.371547000
1	135.886599000	7.913351000	9.336551000
1	136.336570000	7.859265000	11.053716000
1	133.863739000	8.029576000	10.989245000
1	135.374359000	3.809655000	7.173311000
1	137.180992000	6.043735000	9.074847000
8	128.650882000	6.942992000	4.217925000
1	127.883160000	7.450767000	4.570832000
1	129.018761000	7.513206000	3.526483000
1	138.536312000	4.257567000	13.478673000
1	140.518987000	5.016344000	8.947803000

1	133.080517000	5.171655000	19.273726000
1	127.595744000	8.940633000	18.098118000
1	129.735991000	13.620219000	11.762879000
1	124.941698000	10.207326000	5.923777000
1	133.462022000	17.379083000	4.793581000
1	137.681344000	15.086775000	4.913324000
1	141.584473000	12.514484000	10.021879000
1	139.926563000	3.143105000	4.347430000
1	135.476275000	8.822417000	6.038364000
1	129.605668000	4.828246000	4.023231000
1	134.023678000	-0.964077000	2.575024000
1	139.604406000	5.719343000	5.134347000
1	132.780421000	8.547881000	5.820675000
1	129.928837000	2.266134000	3.137692000
1	136.702983000	-0.732756000	2.930661000
1	138.697619000	15.909799000	10.403129000
1	128.826356000	4.104403000	17.425705000

^{4}TS4$_B$:

6	134.656553000	1.025313000	14.050242000
6	135.874478000	0.283616000	13.492504000
8	136.254456000	-0.777909000	13.991193000
6	134.606954000	1.002079000	15.588856000
6	135.773011000	1.790496000	16.202111000
6	133.256309000	1.526039000	16.097096000
1	134.628666000	2.060025000	13.683941000
1	134.712463000	-0.047935000	15.887256000
1	135.744688000	1.744673000	17.297777000
1	135.732472000	2.849286000	15.910887000
1	136.736558000	1.384044000	15.878303000
1	133.203841000	1.473343000	17.192005000
1	133.106764000	2.575538000	15.805095000
1	132.419160000	0.944252000	15.692331000
7	136.465380000	0.862229000	12.414092000
6	137.681454000	0.344677000	11.798434000
6	137.529048000	0.005040000	10.309757000
6	136.606513000	-1.146560000	9.942638000
6	136.130449000	-2.081678000	10.869559000
6	136.240732000	-1.318346000	8.596737000
6	135.326320000	-3.152006000	10.466191000
6	135.446249000	-2.383019000	8.180740000
6	134.985440000	-3.311191000	9.121829000
8	134.205611000	-4.342463000	8.667286000
1	136.136561000	1.768085000	12.091316000
1	137.986866000	-0.525594000	12.382699000
1	138.538964000	-0.210668000	9.928673000
1	137.209233000	0.910912000	9.776740000
1	136.365922000	-1.983915000	11.925256000
1	136.587457000	-0.600847000	7.855285000
1	134.965884000	-3.863861000	11.207640000
1	135.175412000	-2.510887000	7.136919000
1	133.974821000	-4.911292000	9.418202000
6	132.801688000	5.868418000	20.528541000
6	132.109055000	5.827294000	19.176637000
8	132.340059000	6.657630000	18.296038000
6	134.321433000	5.683359000	20.379514000
1	132.596458000	6.853313000	20.964459000
1	134.729393000	6.433964000	19.697388000
1	134.812289000	5.791627000	21.351252000
1	134.566939000	4.692118000	19.983450000
7	131.192784000	4.825751000	18.982051000
6	130.684826000	4.525481000	17.646802000
6	129.882219000	5.651758000	16.989728000
8	129.798742000	5.716522000	15.764632000
1	131.132864000	4.097256000	19.680620000
1	131.500297000	4.293467000	16.954464000
7	129.276630000	6.540234000	17.817106000
6	128.567360000	7.692008000	17.285946000
6	129.414091000	8.643227000	16.424674000
8	128.844875000	9.400902000	15.635048000
1	129.430719000	6.454055000	18.811704000
1	127.738020000	7.371367000	16.649648000
7	130.758261000	8.592622000	16.571516000
6	131.624116000	9.406183000	15.723900000
6	131.463948000	9.095864000	14.226704000
8	131.809088000	9.941296000	13.392229000
6	133.099867000	9.278980000	16.132024000
6	133.439944000	9.831565000	17.524315000
16	132.992740000	11.584286000	17.831970000
6	134.123545000	12.436011000	16.674762000
1	131.185168000	7.905246000	17.189156000
1	131.320869000	10.453218000	15.823899000
1	133.404596000	8.224400000	16.101708000
1	133.686958000	9.795966000	15.366545000
1	134.513415000	9.705606000	17.705956000
1	132.920829000	9.262708000	18.301276000
1	133.998695000	13.507878000	16.850357000
1	133.879174000	12.221794000	15.630394000
1	135.165219000	12.165827000	16.875738000
7	130.925302000	7.905435000	13.877163000
6	130.636780000	7.597447000	12.480859000
6	129.745488000	8.657665000	11.823892000
8	129.818602000	8.838493000	10.593165000
6	129.976431000	6.217590000	12.364120000
1	130.625013000	7.242906000	14.588175000
1	131.563127000	7.601545000	11.899557000
1	129.776619000	5.996555000	11.312503000
1	129.035017000	6.177670000	12.921653000
1	130.642478000	5.447628000	12.764417000
7	128.888068000	9.350776000	12.594675000
6	128.044257000	10.383832000	12.017453000
6	128.780782000	11.613568000	11.467248000
8	128.173917000	12.399385000	10.733060000
1	128.903739000	9.245374000	13.608995000
1	127.338973000	10.717243000	12.783674000
1	127.465578000	9.983673000	11.179391000
7	130.075558000	11.786504000	11.820500000
6	130.880489000	12.837253000	11.228270000
6	131.690771000	12.453632000	9.977753000
8	132.316712000	13.328989000	9.383300000
1	130.536186000	11.125393000	12.444506000
1	130.227716000	13.664308000	10.942242000
7	131.681382000	11.139523000	9.620574000
6	132.482981000	10.625879000	8.517720000
6	131.672164000	9.976312000	7.380066000
8	132.229363000	9.776986000	6.297215000
6	133.545588000	9.607617000	8.993604000
6	134.564159000	10.189579000	9.930100000
6	134.897066000	11.503457000	10.156909000
7	135.356353000	9.374695000	10.722643000
6	136.143359000	10.184650000	11.412492000
7	135.899664000	11.480885000	11.102565000
1	131.234186000	10.471784000	10.240972000
1	132.975385000	11.491077000	8.068268000
1	134.037692000	9.203277000	8.101005000
1	133.049843000	8.757474000	9.480271000
1	134.497169000	12.426558000	9.767924000
1	136.885844000	9.879322000	12.137022000
1	136.354254000	12.299928000	11.489999000
7	130.400072000	9.608275000	7.664276000
6	129.537440000	8.862140000	6.745918000
6	128.356381000	9.764180000	6.322249000
8	128.453719000	10.503855000	5.335291000
6	129.152958000	7.497732000	7.345393000
8	128.402099000	7.580417000	8.552634000
1	130.022701000	9.850088000	8.572249000
1	130.120922000	8.685281000	5.841257000
1	130.073782000	6.917253000	7.496063000
1	128.527365000	6.957238000	6.627994000
1	128.986805000	7.900869000	9.268511000
7	127.267219000	9.712507000	7.117557000
6	126.132006000	10.611466000	6.972022000
6	126.169162000	11.741404000	7.999094000
8	126.103031000	11.191677000	9.305681000
1	127.304401000	9.089367000	7.920837000
1	125.209565000	10.033250000	7.099109000
1	127.091808000	12.321660000	7.859053000
1	125.317584000	12.417009000	7.811891000
1	126.688001000	11.729334000	9.877581000
7	131.918303000	14.448940000	3.513276000

6	132.126398000	15.176391000	4.788102000
6	133.620378000	15.376158000	5.123017000
8	133.950103000	15.978759000	6.149696000
6	131.385511000	14.346247000	5.868282000
6	131.227178000	12.964536000	5.214683000
6	130.999637000	13.311431000	3.742157000
1	131.714983000	16.194331000	4.751104000
1	130.397574000	14.786059000	6.051297000
1	131.920738000	14.332297000	6.820670000
1	132.145678000	12.376512000	5.321516000
1	130.400622000	12.379340000	5.626364000
1	131.226883000	12.473377000	3.076236000
1	129.945935000	13.594333000	3.583453000
7	134.473163000	14.866863000	4.208297000
6	135.912031000	15.084734000	4.197784000
6	136.768957000	13.803799000	4.098518000
6	136.859379000	13.004288000	5.415918000
6	138.184362000	14.196986000	3.639470000
6	135.565624000	12.341288000	5.905999000
1	134.005550000	14.406875000	3.429868000
1	136.172255000	15.638807000	5.103073000
1	136.324891000	13.153716000	3.326931000
1	137.244481000	13.678786000	6.192653000
1	137.620957000	12.223507000	5.275886000
1	138.162564000	14.716485000	2.673506000
1	138.821298000	13.311681000	3.527983000
1	138.652026000	14.864003000	4.373524000
1	135.770268000	11.705024000	6.777337000
1	135.117224000	11.708013000	5.130251000
1	134.818449000	13.081003000	6.206136000
16	135.638617000	4.218810000	2.700415000
1	136.866128000	4.632073000	3.089348000
6	139.835787000	17.189002000	8.152280000
6	138.415051000	16.794440000	7.744593000
8	138.182663000	16.233090000	6.675064000
6	140.585484000	17.862390000	6.992214000
6	142.096322000	18.056182000	7.221095000
6	142.762189000	18.577011000	5.938081000
6	142.402426000	18.988501000	8.403950000
1	139.824134000	17.827070000	9.044080000
1	140.427092000	17.245525000	6.100575000
1	140.123091000	18.837634000	6.779864000
1	142.528487000	17.068832000	7.448589000
1	143.845858000	18.684066000	6.066901000
1	142.587688000	17.898407000	5.094878000
1	142.361017000	19.561072000	5.662727000
1	143.483709000	19.120470000	8.530015000
1	142.006685000	18.600025000	9.349318000
1	141.964102000	19.982322000	8.241165000
7	137.437316000	17.102306000	8.644093000
6	136.037201000	16.735272000	8.452168000
6	135.693338000	15.296464000	8.899244000
6	135.831281000	15.067085000	10.386868000
6	137.079894000	14.784111000	10.968754000
6	134.714209000	15.152999000	11.234292000
6	137.211495000	14.606981000	12.350594000
6	134.841118000	14.976548000	12.614156000
6	136.089717000	14.705829000	13.179882000
1	137.713541000	17.488501000	9.535112000
1	135.791222000	16.821456000	7.393258000
1	134.668328000	15.098220000	8.570110000
1	136.345704000	14.612107000	8.345437000
1	137.954723000	14.695729000	10.329347000
1	133.734753000	15.329048000	10.797203000
1	138.189417000	14.397439000	12.778071000
1	133.961281000	15.047337000	13.249074000
1	136.189345000	14.571984000	14.253959000
7	136.429319000	2.979082000	5.410970000
7	135.474487000	5.642328000	5.417743000
7	133.983467000	2.118281000	4.150990000
6	135.916057000	0.690734000	4.680199000
6	137.644517000	4.917030000	6.299620000
6	133.597743000	7.087063000	4.763651000
6	131.791440000	2.838846000	3.322162000
6	136.745548000	1.651987000	5.248374000
6	136.681313000	5.881404000	6.026490000
6	132.759163000	6.115786000	4.234581000
6	132.762339000	1.875281000	3.576013000
8	134.066494000	3.649162000	6.624260000
6	138.068777000	1.390745000	5.761109000
6	136.819838000	7.289832000	6.322717000
6	131.464475000	6.401320000	3.653119000
6	132.633378000	0.475191000	3.249245000
6	138.551784000	2.576529000	6.229785000
6	135.682728000	7.895703000	5.882347000
6	130.947918000	5.203660000	3.264210000
6	133.799885000	-0.122626000	3.620203000
6	137.527494000	3.564059000	5.998566000
6	134.850588000	6.857108000	5.319303000
6	131.926429000	4.192958000	3.595993000
6	134.639670000	0.910641000	4.178527000
7	133.020731000	4.768783000	4.196175000
26	134.737040000	3.889531000	4.744444000
1	136.303056000	-0.322310000	4.619561000
1	138.571489000	5.248717000	6.758731000
1	133.237782000	8.109301000	4.760919000
1	130.871962000	2.511377000	2.846335000
6	134.136110000	5.792517000	10.811997000
7	135.296996000	6.500387000	10.858076000
8	133.047622000	6.280896000	10.519161000
16	136.819684000	6.383540000	13.249973000
6	134.264128000	4.306802000	11.123147000
7	135.391362000	3.713042000	11.338089000
8	137.654033000	3.869744000	11.193611000
16	135.031504000	6.233618000	14.078834000
6	132.994589000	3.496982000	11.096861000
8	138.941487000	6.341192000	11.283790000
6	132.870800000	2.806738000	9.739496000
6	132.467088000	3.518526000	8.620052000
6	132.472966000	2.905314000	7.319855000
6	132.678092000	1.485570000	7.252665000
6	133.080595000	0.788585000	8.378596000
6	133.208465000	1.442564000	9.615122000
6	136.580611000	4.453129000	11.258830000
6	136.522002000	5.969724000	11.303522000
6	137.738608000	6.614197000	10.607974000
1	132.216875000	4.571611000	8.701238000
1	131.905233000	3.380450000	6.527847000
1	133.043717000	2.747510000	11.891398000
1	132.142562000	4.156880000	11.268358000
1	132.565115000	0.982526000	6.299086000
1	133.298551000	-0.273220000	8.315733000
1	133.556890000	0.888584000	10.482182000
1	137.745496000	6.254659000	9.566959000
1	137.604880000	7.698539000	10.586910000
1	135.237645000	7.543651000	10.756421000
1	134.752701000	3.102890000	7.051166000
1	139.037026000	5.369963000	11.272453000
8	130.755045000	9.760729000	3.755347000
1	129.929768000	10.117463000	4.143811000
1	131.390994000	9.841637000	4.489234000
1	133.764380000	0.519035000	13.654166000
1	138.468035000	1.103142000	11.893437000
1	132.384116000	5.117297000	21.209690000
1	128.146037000	8.256965000	18.122416000
1	131.595288000	13.207847000	11.969533000
1	126.154963000	11.018858000	5.958660000
1	131.578539000	15.065985000	2.782173000
1	136.154679000	15.731695000	3.341385000
1	140.359890000	16.262447000	8.428639000
1	138.554818000	0.423131000	5.749846000
1	137.686885000	7.739949000	6.790372000
1	131.048928000	7.399819000	3.551468000
1	131.762890000	0.027025000	2.786850000
1	139.513770000	2.779514000	6.683474000
1	135.408389000	8.942523000	5.907730000
1	130.000753000	5.004871000	2.778155000
1	134.082717000	-1.163785000	3.527812000
1	135.425216000	17.457947000	9.002350000
1	130.043963000	3.641209000	17.717608000

^{4}IM4$_B$:

6	136.793320000	2.692487000	14.189718000
6	138.035854000	2.359592000	13.356755000
8	138.911570000	1.607407000	13.786787000
6	137.049857000	2.623248000	15.703938000
6	137.998707000	3.736596000	16.169386000
6	135.726421000	2.660735000	16.480665000
1	136.396620000	3.678675000	13.910612000
1	137.540876000	1.661663000	15.894183000
1	138.196589000	3.659356000	17.245649000
1	137.570528000	4.730233000	15.976939000
1	138.959217000	3.670530000	15.649589000
1	135.904160000	2.578695000	17.560741000
1	135.192212000	3.604294000	16.298209000
1	135.063117000	1.837365000	16.186127000
7	138.063880000	2.916210000	12.116537000
6	139.140874000	2.677355000	11.165859000
6	138.700131000	1.918179000	9.903049000
6	138.102171000	0.533571000	10.096360000
6	138.439799000	-0.303908000	11.167167000
6	137.206107000	0.033168000	9.137110000
6	137.905623000	-1.590075000	11.273118000
6	136.670685000	-1.249346000	9.227333000
6	137.023417000	-2.070743000	10.302723000
8	136.470763000	-3.323493000	10.355284000
1	137.343745000	3.581648000	11.847701000
1	139.921342000	2.136711000	11.704743000
1	139.585811000	1.849073000	9.251606000
1	137.980024000	2.540502000	9.354972000
1	139.099146000	0.046897000	11.954552000
1	136.927693000	0.659181000	8.292068000
1	138.177405000	-2.218194000	12.120475000
1	135.981628000	-1.627893000	8.478240000
1	136.817360000	-3.782709000	11.136080000
6	134.240216000	5.821780000	18.804372000
6	133.215433000	5.686007000	17.688619000
8	132.992927000	6.575834000	16.867082000
6	134.429384000	7.266367000	19.272402000
1	133.968045000	5.167745000	19.642633000
1	133.513275000	7.663109000	19.723287000
1	135.225541000	7.319958000	20.020833000
1	134.699845000	7.909647000	18.431667000
7	132.532148000	4.494189000	17.640180000
6	131.812561000	4.102501000	16.431625000
6	130.617143000	4.989663000	16.075925000
8	130.229232000	5.063778000	14.910838000
1	132.858247000	3.749052000	18.242218000
1	132.467202000	4.115547000	15.554334000
7	130.023201000	5.657544000	17.095872000
6	128.935167000	6.589750000	16.849801000
6	129.275037000	7.756412000	15.907257000
8	128.360102000	8.353741000	15.336563000
1	130.433656000	5.587364000	18.016517000
1	128.084283000	6.075460000	16.394854000
7	130.584033000	8.062332000	15.743253000
6	130.986163000	9.089087000	14.789646000
6	130.587683000	8.758744000	13.346178000
8	130.551241000	9.681006000	12.514986000
6	132.491255000	9.382743000	14.867294000
6	132.948967000	10.043050000	16.174867000
16	132.124961000	11.624250000	16.611661000
6	132.651776000	12.688632000	15.221652000
1	131.307810000	7.496019000	16.180598000
1	130.437812000	10.007812000	15.023604000
1	133.056952000	8.451356000	14.736602000
1	132.742527000	10.017629000	14.012494000
1	134.031854000	10.206211000	16.133391000
1	132.760348000	9.383874000	17.027641000
1	132.272641000	13.691118000	15.437785000
1	132.234744000	12.354009000	14.267815000
1	133.743284000	12.734001000	15.151759000
7	130.239835000	7.494413000	13.043035000
6	129.739794000	7.165693000	11.715405000
6	128.549281000	8.049078000	11.322117000
8	128.349978000	8.318554000	10.128399000
6	129.350492000	5.684434000	11.628551000
1	130.295245000	6.755317000	13.740316000
1	130.518361000	7.382525000	10.978455000
1	128.968039000	5.465646000	10.627235000
1	128.579487000	5.433291000	12.364000000
1	130.222815000	5.050813000	11.813174000
7	127.715329000	8.492173000	12.286467000
6	126.590652000	9.337659000	11.914932000
6	126.962051000	10.729851000	11.377452000
8	126.154121000	11.348255000	10.681168000
1	127.928440000	8.338860000	13.271019000
1	125.948228000	9.461691000	12.791538000
1	126.009950000	8.857180000	11.123346000
7	128.165133000	11.231195000	11.747718000
6	128.674436000	12.477570000	11.208673000
6	129.741158000	12.373654000	10.105045000
8	130.065159000	13.390367000	9.497606000
1	128.800074000	10.653596000	12.290947000
1	127.834440000	13.030749000	10.785060000
7	130.282032000	11.140813000	9.871911000
6	131.359606000	10.962128000	8.908669000
6	130.971793000	10.158013000	7.650294000
8	131.741474000	10.180782000	6.681495000
6	132.613729000	10.339026000	9.552113000
6	133.336186000	11.261353000	10.491152000
6	133.199008000	12.616030000	10.681137000
7	134.360239000	10.788401000	11.298195000
6	134.830434000	11.838826000	11.950984000
7	134.153258000	12.963651000	11.616587000
1	130.165946000	10.426272000	10.584129000
1	131.601526000	11.956262000	8.525369000
1	133.287697000	10.047935000	8.738013000
1	132.347538000	9.417642000	10.085364000
1	132.514404000	13.336354000	10.258101000
1	135.649851000	11.837415000	12.655412000
1	134.379496000	13.904180000	11.918279000
7	129.817926000	9.456725000	7.693127000
6	129.293931000	8.697704000	6.550760000
6	128.000729000	9.390333000	6.060990000
8	128.058435000	10.314799000	5.240221000
6	129.190384000	7.189778000	6.845464000
8	128.220573000	6.834296000	7.827737000
1	129.240940000	9.535696000	8.524480000
1	130.021334000	8.822386000	5.747953000
1	130.186203000	6.814636000	7.117455000
1	128.889746000	6.680879000	5.923981000
1	128.459356000	7.252785000	8.679837000
7	126.851838000	8.959652000	6.621545000
6	125.565532000	9.596419000	6.375640000
6	125.151516000	10.533616000	7.507820000
8	124.948569000	9.781163000	8.691558000
1	126.914667000	8.187644000	7.282159000
1	124.806750000	8.814281000	6.257759000
1	125.932924000	11.294270000	7.646470000
1	124.228086000	11.057614000	7.205111000
1	125.181350000	10.358474000	9.445126000
7	132.320220000	14.796621000	4.198201000
6	132.884458000	15.567967000	5.328639000
6	134.374576000	15.250590000	5.582262000
8	134.989550000	15.832965000	6.483881000
6	132.006737000	15.205949000	6.553675000
6	131.337193000	13.886566000	6.140698000
6	131.097288000	14.087581000	4.642051000
1	132.854524000	16.650897000	5.147638000
1	131.243656000	15.978606000	6.704190000
1	132.597382000	15.142855000	7.470837000
1	132.017530000	13.040277000	6.293167000
1	130.419599000	13.686257000	6.699874000
1	130.973505000	13.142255000	4.104188000
1	130.190959000	14.696130000	4.485432000
7	134.897555000	14.322962000	4.756276000
6	136.300934000	13.940653000	4.705501000
6	136.552155000	12.418181000	4.802292000
6	136.539328000	11.877692000	6.247756000

6	137.906399000	12.090367000	4.150402000
6	135.243870000	12.069405000	7.045953000
1	134.229021000	13.958159000	4.079956000
1	136.820749000	14.463787000	5.512007000
1	135.761362000	11.907293000	4.229837000
1	137.369058000	12.351933000	6.788114000
1	136.774087000	10.803934000	6.201242000
1	137.926697000	12.389449000	3.095457000
1	138.116023000	11.015317000	4.196253000
1	138.716542000	12.617328000	4.669367000
1	135.333772000	11.597530000	8.033738000
1	134.374784000	11.622694000	6.546429000
1	135.026129000	13.128510000	7.210248000
16	135.195280000	4.988534000	2.127242000
1	136.164210000	4.068480000	2.337573000
6	140.822831000	14.160113000	8.536594000
6	139.495203000	14.727768000	8.040227000
8	138.993541000	14.354544000	6.981409000
6	140.777508000	12.621956000	8.561246000
6	142.052267000	11.934009000	9.085334000
6	141.826125000	10.416297000	9.179711000
6	143.287633000	12.243442000	8.225718000
1	141.601650000	14.502423000	7.844809000
1	139.930091000	12.304959000	9.186131000
1	140.554121000	12.271596000	7.546170000
1	142.244750000	12.309621000	10.103042000
1	142.699524000	9.913228000	9.612067000
1	140.953550000	10.173801000	9.798133000
1	141.657841000	9.988385000	8.182472000
1	144.169922000	11.715734000	8.606955000
1	143.526533000	13.312744000	8.210932000
1	143.130540000	11.921643000	7.187894000
7	138.907829000	15.660585000	8.847529000
6	137.572668000	16.190632000	8.594128000
6	136.443130000	15.366818000	9.251181000
6	136.437643000	15.397600000	10.762352000
6	137.301050000	14.583460000	11.515845000
6	135.557146000	16.240153000	11.461771000
6	137.292088000	14.614600000	12.912860000
6	135.536399000	16.268644000	12.860112000
6	136.405958000	15.455098000	13.592555000
1	139.337581000	15.858465000	9.739733000
1	137.404377000	16.183792000	7.516641000
1	135.499250000	15.746263000	8.849068000
1	136.538833000	14.335396000	8.894083000
1	137.974282000	13.904364000	10.998877000
1	134.877369000	16.877492000	10.900471000
1	137.974455000	13.977780000	13.470914000
1	134.844057000	16.929549000	13.375646000
1	136.395182000	15.479017000	14.678930000
7	135.284201000	3.252670000	4.975832000
7	134.588761000	5.971599000	5.312660000
7	132.942879000	2.835715000	3.444266000
6	134.549245000	1.087954000	4.065975000
6	136.587479000	4.902342000	6.256727000
6	133.026660000	7.737623000	4.615868000
6	131.039953000	3.944883000	2.361332000
6	135.435816000	1.898103000	4.761410000
6	135.752810000	5.989736000	6.054870000
6	132.220636000	6.950437000	3.809154000
6	131.795051000	2.828932000	2.684860000
8	132.107356000	4.932298000	7.175395000
6	136.640048000	1.426337000	5.387573000
6	135.999377000	7.312916000	6.563465000
6	131.062860000	7.452267000	3.113157000
6	131.493119000	1.491242000	2.243722000
6	137.217431000	2.502876000	5.996753000
6	134.990057000	8.108440000	6.107979000
6	130.503824000	6.382556000	2.479746000
6	132.477894000	0.685411000	2.727240000
6	136.363281000	3.631113000	5.748885000
6	134.129562000	7.272898000	5.314766000
6	131.318437000	5.234980000	2.784898000
6	133.383496000	1.531175000	3.462336000
7	132.376380000	5.598626000	3.588841000
26	133.932408000	4.465044000	4.118822000
1	134.789391000	0.032411000	3.986368000
1	137.477488000	5.053770000	6.859457000
1	132.765439000	8.784105000	4.715255000
1	130.162915000	3.797876000	1.739208000
6	134.295841000	7.020162000	11.377626000
7	135.213560000	8.023589000	11.280417000
8	133.082483000	7.204800000	11.376354000
16	137.243360000	8.042201000	13.281930000
6	134.875986000	5.614542000	11.460057000
7	136.130787000	5.347871000	11.297790000
8	138.132590000	6.166971000	10.580843000
16	135.807879000	7.281294000	14.407638000
6	133.893987000	4.494903000	11.667605000
8	138.780317000	8.856655000	10.983583000
6	133.498055000	3.886574000	10.317229000
6	132.547691000	4.482888000	9.529761000
6	132.111745000	3.920596000	8.203085000
6	132.890783000	2.701501000	7.800517000
6	133.849836000	2.152082000	8.606266000
6	134.164577000	2.710870000	9.870164000
6	137.022034000	6.399842000	11.036473000
6	136.603059000	7.819500000	11.384063000
6	137.421806000	8.874867000	10.614898000
1	132.072679000	5.408598000	9.840490000
1	131.041987000	3.655567000	8.268685000
1	134.369558000	3.729097000	12.287100000
1	133.013722000	4.882225000	12.184070000
1	132.660411000	2.277176000	6.827417000
1	134.391163000	1.268545000	8.279469000
1	134.931187000	2.251589000	10.486932000
1	137.275626000	8.688472000	9.540186000
1	137.028434000	9.869434000	10.839055000
1	134.856313000	9.009255000	11.304902000
1	133.029063000	5.150141000	6.945944000
1	139.112752000	7.974060000	10.732268000
8	130.619376000	10.613944000	4.013304000
1	129.687136000	10.616076000	4.320992000
1	131.120390000	10.602028000	4.849719000
1	136.024380000	1.954548000	13.915777000
1	139.554822000	3.644628000	10.857398000
1	135.187353000	5.431933000	18.404953000
1	128.603885000	6.997222000	17.809287000
1	129.100986000	13.086872000	12.013741000
1	125.643695000	10.154542000	5.439517000
1	132.144170000	15.384544000	3.389809000
1	136.722374000	14.306181000	3.757599000
1	141.079161000	14.553679000	9.529844000
1	136.988911000	0.401764000	5.358906000
1	136.855626000	7.586457000	7.167833000
1	130.753269000	8.492848000	3.141537000
1	130.637189000	1.222423000	1.637932000
1	138.135725000	2.540894000	6.568591000
1	134.836773000	9.168945000	6.256556000
1	129.615626000	6.355308000	1.861102000
1	132.599808000	-0.382843000	2.600378000
1	137.542882000	17.229067000	8.942583000
1	131.452504000	3.077933000	16.567477000

^2Re_B:

6	136.938618000	2.737279000	13.303027000
6	138.331447000	2.830958000	12.672757000
8	139.204202000	1.992367000	12.880840000
6	136.900042000	1.882467000	14.578890000
6	137.713372000	2.516731000	15.716026000
6	135.449149000	1.625502000	15.010670000
1	136.545336000	3.744264000	13.502452000
1	137.367972000	0.921860000	14.332015000
1	137.682761000	1.893899000	16.618561000
1	137.315453000	3.508105000	15.980445000
1	138.760385000	2.636547000	15.424208000
1	135.409143000	0.963806000	15.885129000
1	134.949946000	2.568659000	15.279596000
1	134.862652000	1.164266000	14.207353000
7	138.506043000	3.903842000	11.844629000

6	139.713865000	4.119477000	11.065455000
6	139.497694000	3.985503000	9.540276000
6	139.132872000	2.590714000	9.081518000
6	140.113088000	1.699356000	8.628869000
6	137.803615000	2.139767000	9.103453000
6	139.788352000	0.409318000	8.204282000
6	137.463130000	0.853043000	8.694721000
6	138.457591000	-0.015862000	8.235595000
8	138.065713000	-1.260875000	7.806322000
1	137.751467000	4.573508000	11.743715000
1	140.441831000	3.384532000	11.416601000
1	140.428198000	4.302076000	9.050502000
1	138.723588000	4.696064000	9.222693000
1	141.153526000	2.016193000	8.600169000
1	137.016327000	2.811113000	9.435332000
1	140.568930000	-0.261731000	7.848567000
1	136.432032000	0.515296000	8.707364000
1	138.855573000	-1.769942000	7.565545000
6	134.862910000	5.612719000	17.605739000
6	133.680308000	5.651157000	16.647835000
8	133.384390000	6.642325000	15.981824000
6	135.315539000	7.001165000	18.058601000
1	134.616060000	4.977480000	18.466529000
1	134.531328000	7.509718000	18.629207000
1	136.201788000	6.920724000	18.695188000
1	135.562212000	7.626133000	17.196790000
7	132.938517000	4.495423000	16.570321000
6	132.043826000	4.259219000	15.439666000
6	130.824332000	5.179288000	15.365225000
8	130.224687000	5.317678000	14.298811000
1	133.315274000	3.676042000	17.030063000
1	132.568212000	4.378849000	14.486014000
7	130.455852000	5.814450000	16.505372000
6	129.396170000	6.809093000	16.504785000
6	129.636953000	8.025503000	15.592889000
8	128.675954000	8.717715000	15.249719000
1	131.035649000	5.692876000	17.323720000
1	128.456424000	6.364995000	16.165657000
7	130.908856000	8.255223000	15.189601000
6	131.201648000	9.306749000	14.222628000
6	130.485127000	9.102226000	12.880310000
8	130.358702000	10.076345000	12.118961000
6	132.712947000	9.472649000	14.000521000
6	133.489126000	9.990832000	15.221386000
16	132.861806000	11.533929000	15.993798000
6	133.097002000	12.737291000	14.637791000
1	131.662280000	7.633077000	15.475328000
1	130.799797000	10.249660000	14.608439000
1	133.152634000	8.511290000	13.701908000
1	132.841742000	10.149585000	13.150386000
1	134.538748000	10.134336000	14.940961000
1	133.476021000	9.251092000	16.027006000
1	132.758538000	13.704238000	15.020073000
1	132.500028000	12.480585000	13.758266000
1	134.151853000	12.823797000	14.359841000
7	129.970533000	7.889640000	12.610931000
6	129.183653000	7.642569000	11.410024000
6	128.019874000	8.630005000	11.275750000
8	127.589125000	8.922288000	10.144296000
6	128.663060000	6.197335000	11.400403000
1	130.133869000	7.109286000	13.242785000
1	129.817783000	7.803499000	10.534627000
1	128.148839000	5.996913000	10.457136000
1	127.980544000	6.013846000	12.236727000
1	129.502505000	5.501703000	11.487795000
7	127.456411000	9.147202000	12.383864000
6	126.372617000	10.109877000	12.243666000
6	126.771604000	11.448865000	11.596873000
8	125.918401000	12.130072000	11.021253000
1	127.843343000	8.943322000	13.304822000
1	125.962338000	10.311136000	13.237674000
1	125.580478000	9.693450000	11.616805000
7	128.058513000	11.843578000	11.747137000
6	128.567693000	13.039562000	11.102135000
6	129.362827000	12.849404000	9.798063000
8	129.586322000	13.833358000	9.096169000
1	128.728750000	11.211895000	12.178253000
1	127.719821000	13.682113000	10.860943000
7	129.796456000	11.588481000	9.509358000
6	130.648929000	11.335614000	8.353255000
6	130.023552000	10.426037000	7.274008000
8	130.549917000	10.374042000	6.162416000
6	132.004756000	10.726954000	8.761422000
6	132.846365000	11.590083000	9.656034000
6	132.815720000	12.946106000	9.883444000
7	133.883062000	11.026901000	10.385235000
6	134.465657000	12.022408000	11.028448000
7	133.852066000	13.202478000	10.761702000
1	129.810548000	10.905743000	10.263229000
1	130.802645000	12.300038000	7.864976000
1	132.548532000	10.505747000	7.834951000
1	131.838046000	9.764853000	9.259415000
1	132.149652000	13.719272000	9.530967000
1	135.319696000	11.948767000	11.686029000
1	134.145738000	14.105058000	11.112551000
7	128.941879000	9.690120000	7.639890000
6	128.347844000	8.708269000	6.739657000
6	126.875985000	8.975765000	6.383231000
8	126.438415000	8.607606000	5.282633000
6	128.506917000	7.252581000	7.243830000
8	127.559200000	6.872627000	8.224783000
1	128.608795000	9.752911000	8.595408000
1	128.904499000	8.794425000	5.804792000
1	129.543794000	7.133364000	7.597412000
1	128.358391000	6.578495000	6.397493000
1	127.564152000	7.546133000	8.932196000
7	126.104442000	9.595404000	7.298684000
6	124.677416000	9.816216000	7.103702000
6	124.193345000	10.892647000	8.069661000
8	124.405703000	10.442766000	9.398895000
1	126.421671000	9.689402000	8.257341000
1	124.119443000	8.886038000	7.278045000
1	124.735550000	11.828996000	7.875219000
1	123.123470000	11.079317000	7.885909000
1	124.754688000	11.175285000	9.945342000
7	133.351404000	15.792336000	4.646665000
6	133.948718000	16.320417000	5.895512000
6	135.351803000	15.742907000	6.183021000
8	135.979056000	16.114478000	7.180789000
6	132.941903000	15.959540000	7.017527000
6	132.116941000	14.814631000	6.408815000
6	132.012318000	15.235889000	4.940329000
1	134.094393000	17.407671000	5.853462000
1	132.292339000	16.818456000	7.223388000
1	133.452668000	15.698895000	7.947799000
1	132.663994000	13.867449000	6.484506000
1	131.142479000	14.684529000	6.888460000
1	131.787489000	14.399239000	4.269833000
1	131.214945000	15.987325000	4.819909000
7	135.792453000	14.846288000	5.278203000
6	137.117440000	14.241051000	5.289398000
6	137.122737000	12.739875000	4.927239000
6	136.739579000	11.814397000	6.101432000
6	138.520283000	12.355527000	4.411864000
6	135.355709000	12.032866000	6.723308000
1	135.142582000	14.674652000	4.514805000
1	137.548324000	14.390771000	6.282538000
1	136.400011000	12.585468000	4.108606000
1	137.507260000	11.919648000	6.878022000
1	136.806250000	10.776819000	5.739030000
1	138.798732000	12.940673000	3.527052000
1	138.560745000	11.294683000	4.137867000
1	139.272896000	12.531668000	5.189774000
1	135.147883000	11.265524000	7.478982000
1	134.557068000	11.983313000	5.971601000
1	135.285701000	13.005516000	7.218703000
16	134.323063000	3.966251000	2.707093000
1	133.536352000	5.018235000	2.386491000
6	140.951026000	12.702949000	9.574436000
6	139.827152000	13.577929000	9.029098000

8	139.247737000	13.311516000	7.976683000
6	140.397322000	11.549212000	10.436612000
6	141.464189000	10.564102000	10.958163000
6	140.855776000	9.647145000	12.031389000
6	142.082991000	9.729831000	9.825982000
1	141.485867000	12.307478000	8.706807000
1	139.869779000	11.981533000	11.299483000
1	139.647248000	10.992654000	9.859190000
1	142.267635000	11.153699000	11.427657000
1	141.602386000	8.942158000	12.416760000
1	140.478179000	10.228735000	12.882010000
1	140.020218000	9.068041000	11.620383000
1	142.846163000	9.046779000	10.217433000
1	142.561816000	10.353100000	9.062558000
1	141.313215000	9.123676000	9.331536000
7	139.482815000	14.651659000	9.800738000
6	138.343122000	15.505544000	9.483526000
6	136.982280000	14.953788000	9.964603000
6	136.787446000	14.984082000	11.463604000
6	137.312824000	13.979900000	12.295872000
6	136.082065000	16.036550000	12.071505000
6	137.150348000	14.031762000	13.682620000
6	135.912436000	16.089955000	13.457866000
6	136.448502000	15.088273000	14.269768000
1	139.945182000	14.773895000	10.690215000
1	138.290066000	15.615755000	8.399527000
1	136.213184000	15.547487000	9.461179000
1	136.888088000	13.930719000	9.583019000
1	137.850652000	13.147628000	11.848078000
1	135.670636000	16.827453000	11.447428000
1	137.571932000	13.246471000	14.305321000
1	135.363220000	16.915941000	13.902521000
1	136.320829000	15.128761000	15.348003000
7	136.183947000	4.516592000	5.286883000
7	133.896477000	6.191632000	5.005602000
7	134.510513000	2.217376000	5.174326000
6	136.940710000	2.193774000	5.477253000
6	136.213591000	6.963308000	5.196341000
6	131.462509000	6.222415000	4.702070000
6	132.197382000	1.449757000	4.908594000
6	137.159416000	3.565460000	5.418745000
6	134.846686000	7.178572000	5.097231000
6	131.266249000	4.851568000	4.631532000
6	133.555466000	1.235379000	5.118010000
8	134.004369000	4.237554000	6.852992000
6	138.459573000	4.184208000	5.487567000
6	134.223103000	8.478796000	5.065884000
6	129.984570000	4.228709000	4.401361000
6	134.160779000	-0.061221000	5.294603000
6	138.255605000	5.526996000	5.395662000
6	132.881492000	8.272399000	4.952667000
6	130.190113000	2.885783000	4.472562000
6	135.497266000	0.145916000	5.457391000
6	136.832447000	5.720555000	5.278801000
6	132.694309000	6.842129000	4.899921000
6	131.595040000	2.684827000	4.734106000
6	135.704911000	1.570269000	5.376764000
7	132.244464000	3.897454000	4.805189000
26	134.197036000	4.211999000	5.220114000
1	137.807792000	1.558964000	5.625432000
1	136.853740000	7.839515000	5.231415000
1	130.584003000	6.848690000	4.568544000
1	131.557423000	0.573698000	4.863761000
6	133.280287000	7.283511000	10.108902000
7	134.339884000	8.140889000	10.148582000
8	132.106282000	7.635394000	10.115093000
16	136.028163000	6.757966000	11.896879000
6	133.722026000	5.804630000	10.085199000
7	134.825242000	5.627578000	9.178350000
8	136.866911000	6.422880000	8.485565000
16	134.284850000	5.538889000	11.915673000
6	132.539403000	4.861831000	9.792272000
8	138.029568000	8.275605000	10.162397000
6	132.657261000	3.439487000	10.305315000
6	133.337439000	2.454609000	9.573671000
6	133.433507000	1.150097000	10.061850000
6	132.846034000	0.806445000	11.282128000
6	132.148016000	1.774890000	12.005683000
6	132.051171000	3.080635000	11.518932000
6	135.861532000	6.501571000	9.194700000
6	135.669864000	7.630813000	10.215602000
6	136.709781000	8.746190000	10.027314000
1	133.765544000	2.707866000	8.607255000
1	133.952663000	0.395166000	9.476997000
1	131.656561000	5.343214000	10.217685000
1	132.412793000	4.864716000	8.703155000
1	132.913755000	-0.212639000	11.653948000
1	131.661132000	1.509463000	12.941207000
1	131.488401000	3.822545000	12.082790000
1	136.527512000	9.192641000	9.038467000
1	136.553613000	9.519544000	10.783329000
1	134.165608000	9.155467000	10.298643000
1	134.741113000	4.964442000	8.392132000
1	138.120431000	7.594716000	9.464978000
8	128.319637000	7.127411000	3.851215000
1	127.586383000	7.608653000	4.304876000
1	128.438747000	7.604194000	3.016503000
1	136.270537000	2.300619000	12.547090000
1	140.106578000	5.122196000	11.281196000
1	135.682615000	5.101826000	17.079649000
1	129.247379000	7.159679000	17.530031000
1	129.213972000	13.585967000	11.798906000
1	124.508673000	10.115420000	6.065500000
1	133.314414000	16.500490000	3.920291000
1	137.757567000	14.780613000	4.575158000
1	141.661902000	13.298450000	10.163666000
1	139.390458000	3.644509000	5.601466000
1	134.757072000	9.418314000	5.127420000
1	129.076072000	4.785646000	4.207507000
1	133.618656000	-0.998246000	5.290239000
1	138.985300000	6.325909000	5.416126000
1	132.080580000	9.001408000	4.937192000
1	129.468863000	2.086512000	4.358542000
1	136.279443000	-0.579561000	5.640883000
1	138.529435000	16.493550000	9.917616000
1	131.689425000	3.225662000	15.497274000

^{2}TS1$_B$:

6	136.454372000	2.296420000	13.401293000
6	137.869751000	2.407206000	12.829588000
8	138.742330000	1.577851000	13.078627000
6	136.403223000	1.525049000	14.729904000
6	137.143754000	2.268772000	15.850416000
6	134.952139000	1.227245000	15.131145000
1	136.003815000	3.292642000	13.510655000
1	136.923753000	0.574138000	14.563666000
1	137.128157000	1.693118000	16.784365000
1	136.674156000	3.243337000	16.051028000
1	138.189342000	2.441664000	15.580094000
1	134.910471000	0.626464000	16.048534000
1	134.399673000	2.161050000	15.313781000
1	134.416236000	0.685626000	14.343546000
7	138.072009000	3.478076000	12.006264000
6	139.324234000	3.713745000	11.308128000
6	139.199304000	3.627420000	9.770238000
6	138.837585000	2.255437000	9.246325000
6	139.824067000	1.344032000	8.849219000
6	137.496636000	1.852313000	9.141327000
6	139.495752000	0.080885000	8.350893000
6	137.152229000	0.592112000	8.657240000
6	138.153843000	-0.295990000	8.252277000
8	137.758790000	-1.508646000	7.737810000
1	137.325125000	4.151619000	11.878326000
1	140.028742000	2.967148000	11.682104000
1	140.164881000	3.940073000	9.349726000
1	138.456395000	4.357908000	9.426800000
1	140.872946000	1.624214000	8.922571000
1	136.707437000	2.543390000	9.424461000
1	140.281676000	-0.606187000	8.040223000
1	136.113615000	0.289943000	8.567561000

1	138.548060000	-2.047874000	7.572356000	1	132.276835000	13.632728000	9.602711000
6	134.839852000	5.665584000	17.694958000	1	135.431949000	11.791337000	11.721355000
6	133.662903000	5.733246000	16.732161000	1	134.279559000	13.972099000	11.185897000
8	133.349551000	6.759374000	16.129095000	7	129.043706000	9.647174000	7.646511000
6	135.195423000	7.022566000	18.304456000	6	128.471629000	8.652163000	6.747930000
1	134.635274000	4.922910000	18.477014000	6	127.010024000	8.914553000	6.350916000
1	134.372777000	7.415002000	18.911794000	8	126.603059000	8.536086000	5.241077000
1	136.077367000	6.929983000	18.945505000	6	128.618964000	7.202701000	7.275292000
1	135.411274000	7.752227000	17.520143000	8	127.630710000	6.825689000	8.214863000
7	132.949217000	4.569224000	16.577107000	1	128.699806000	9.713079000	8.598456000
6	132.079051000	4.371257000	15.419617000	1	129.050290000	8.728015000	5.825857000
6	130.813856000	5.227668000	15.383720000	1	129.639500000	7.094345000	7.676822000
8	130.184401000	5.343709000	14.331185000	1	128.511060000	6.519311000	6.429397000
1	133.335485000	3.733804000	16.998345000	1	127.616697000	7.495992000	8.925880000
1	132.612035000	4.588231000	14.487399000	7	126.216392000	9.542524000	7.239691000
7	130.443400000	5.851414000	16.529873000	6	124.796435000	9.772040000	7.007214000
6	129.378242000	6.840459000	16.527969000	6	124.297192000	10.858336000	7.955004000
6	129.615101000	8.048561000	15.602444000	8	124.477138000	10.417024000	9.291293000
8	128.651692000	8.726631000	15.237074000	1	126.511305000	9.646569000	8.204722000
1	131.048673000	5.762700000	17.333605000	1	124.227799000	8.846932000	7.173350000
1	128.439791000	6.389808000	16.194896000	1	124.848726000	11.790270000	7.765643000
7	130.890044000	8.283595000	15.212991000	1	123.232774000	11.049487000	7.745812000
6	131.192446000	9.321734000	14.235432000	1	124.827046000	11.148502000	9.839116000
6	130.494669000	9.096790000	12.887148000	7	133.188723000	15.858743000	4.647764000
8	130.375452000	10.062514000	12.112508000	6	133.786651000	16.460341000	5.863167000
6	132.706550000	9.485971000	14.029757000	6	135.223260000	15.962955000	6.136367000
6	133.465255000	10.037999000	15.246488000	8	135.847824000	16.387401000	7.114495000
16	132.822525000	11.598325000	15.971531000	6	132.827369000	16.089941000	7.021434000
6	133.043273000	12.761178000	14.578396000	6	132.074378000	14.862983000	6.484168000
1	131.647184000	7.679830000	15.528650000	6	131.906057000	15.209226000	5.001745000
1	130.783536000	10.269844000	14.601051000	1	133.876725000	17.550879000	5.776859000
1	133.151467000	8.518307000	13.760942000	1	132.122916000	16.911476000	7.197868000
1	132.846514000	10.141094000	13.164568000	1	133.370746000	15.912331000	7.952772000
1	134.517322000	10.178919000	14.974152000	1	132.691580000	13.962352000	6.587200000
1	133.445821000	9.319633000	16.071024000	1	131.124301000	14.682848000	6.995001000
1	132.696296000	13.736518000	14.930648000	1	131.734255000	14.328039000	4.373774000
1	132.447810000	12.470678000	13.708381000	1	131.048411000	15.888364000	4.868612000
1	134.096482000	12.848696000	14.294963000	7	135.694470000	15.072442000	5.240656000
7	129.984785000	7.881500000	12.625831000	6	137.049952000	14.539044000	5.239354000
6	129.207482000	7.618791000	11.421625000	6	137.131069000	13.041184000	4.872983000
6	128.052758000	8.611252000	11.259934000	6	136.803183000	12.091503000	6.045249000
8	127.637457000	8.892741000	10.118972000	6	138.545091000	12.732335000	4.351344000
6	128.670513000	6.179439000	11.436934000	6	135.410030000	12.226813000	6.670290000
1	130.146280000	7.107034000	13.265720000	1	135.041497000	14.853179000	4.492255000
1	129.852183000	7.754196000	10.549616000	1	137.482585000	14.709824000	6.228199000
1	128.159815000	5.966518000	10.494604000	1	136.414070000	12.850507000	4.056957000
1	127.981823000	6.019536000	12.272934000	1	137.564671000	12.238958000	6.821089000
1	129.501519000	5.477038000	11.541544000	1	136.930328000	11.061383000	5.677147000
7	127.475439000	9.141685000	12.354276000	1	138.788192000	13.332566000	3.466208000
6	126.397601000	10.106771000	12.189328000	1	138.640924000	11.675401000	4.075813000
6	126.812422000	11.435346000	11.531315000	1	139.289875000	12.948552000	5.126615000
8	125.974016000	12.109774000	10.926016000	1	135.254238000	11.456619000	7.436207000
1	127.848313000	8.944431000	13.282860000	1	134.614128000	12.120041000	5.921617000
1	125.977083000	10.322368000	13.176065000	1	135.279330000	13.197632000	7.156539000
1	125.610544000	9.686432000	11.558766000	16	134.936893000	4.399670000	2.945116000
7	128.095988000	11.829541000	11.706948000	1	133.722087000	4.795749000	2.505566000
6	128.623842000	13.016905000	11.061475000	6	140.961294000	13.231229000	9.428443000
6	129.445944000	12.811219000	9.776575000	6	139.790300000	14.062997000	8.918235000
8	129.681612000	13.787337000	9.067486000	8	139.238808000	13.807385000	7.847746000
1	128.755489000	11.198872000	12.156176000	6	140.470476000	11.889595000	10.010196000
1	127.784276000	13.660413000	10.795165000	6	141.591367000	10.916121000	10.427638000
7	129.886615000	11.547645000	9.512156000	6	141.012204000	9.777991000	11.283297000
6	130.756250000	11.279372000	8.372718000	6	142.348758000	10.353282000	9.215096000
6	130.135599000	10.370763000	7.292203000	1	141.615386000	13.054617000	8.569148000
8	130.677173000	10.300929000	6.186694000	1	139.841629000	12.099094000	10.887809000
6	132.092772000	10.646075000	8.803857000	1	139.820956000	11.401590000	9.271936000
6	132.944221000	11.492043000	9.703613000	1	142.310578000	11.474666000	11.047718000
6	132.931161000	12.845602000	9.946512000	1	141.793858000	9.063339000	11.568310000
7	133.977174000	10.905225000	10.418453000	1	140.565386000	10.166568000	12.207129000
6	134.574846000	11.885607000	11.070062000	1	140.229681000	9.227124000	10.746670000
7	133.975701000	13.078010000	10.821632000	1	143.153244000	9.681399000	9.536677000
1	129.889170000	10.873468000	10.274514000	1	142.802807000	11.142199000	8.605048000
1	130.933946000	12.238808000	7.882471000	1	141.672605000	9.778214000	8.569319000
1	132.645868000	10.410950000	7.886616000	7	139.373439000	15.076612000	9.732149000
1	131.900500000	9.686686000	9.298659000	6	138.181841000	15.867257000	9.441815000

6	136.861388000	15.210129000	9.902329000
6	136.669649000	15.172992000	11.401709000
6	137.262517000	14.175612000	12.196457000
6	135.899620000	16.155077000	12.047956000
6	137.102553000	14.167595000	13.584360000
6	135.733110000	16.148294000	13.435685000
6	136.336612000	15.155333000	14.210121000
1	139.817488000	15.185592000	10.632523000
1	138.121139000	16.011073000	8.362218000
1	136.053059000	15.769681000	9.421788000
1	136.833768000	14.197600000	9.483825000
1	137.850598000	13.396085000	11.717901000
1	135.433014000	16.937317000	11.452765000
1	137.575318000	13.388935000	14.177866000
1	135.132799000	16.920121000	13.910499000
1	136.210532000	15.148614000	15.289261000
7	136.353538000	4.416519000	5.526045000
7	134.115981000	6.162033000	5.324130000
7	134.669770000	2.169893000	5.065815000
6	137.057144000	2.064680000	5.584586000
6	136.434316000	6.861185000	5.689593000
6	131.712855000	6.277926000	4.847095000
6	132.394472000	1.499583000	4.464532000
6	137.303033000	3.426128000	5.644587000
6	135.079134000	7.115673000	5.555550000
6	131.508946000	4.927340000	4.593929000
6	133.713475000	1.225013000	4.791722000
8	134.023957000	3.951194000	6.942451000
6	138.602659000	4.009002000	5.844388000
6	134.478677000	8.424722000	5.609336000
6	130.243942000	4.365882000	4.196139000
6	134.278632000	-0.097346000	4.891890000
6	138.430588000	5.359912000	5.846473000
6	133.143414000	8.260141000	5.393654000
6	130.439691000	3.020577000	4.087394000
6	135.588701000	0.060181000	5.231077000
6	137.024896000	5.598768000	5.663842000
6	132.934830000	6.846438000	5.199040000
6	131.818825000	2.766818000	4.413648000
6	135.818969000	1.480256000	5.322496000
7	132.463351000	3.941663000	4.702342000
26	134.385813000	4.170506000	5.239185000
1	137.898418000	1.400487000	5.747549000
1	137.089784000	7.711596000	5.844142000
1	130.852981000	6.931713000	4.725316000
1	131.749517000	0.654856000	4.242966000
6	133.234492000	7.114435000	10.038432000
7	134.314142000	7.948949000	10.097455000
8	132.068740000	7.505692000	9.990382000
16	135.940544000	6.599506000	11.962575000
6	133.631466000	5.638328000	9.992616000
7	134.743402000	5.320442000	9.246015000
8	136.838755000	6.038518000	8.647695000
16	134.121187000	5.616831000	12.131895000
6	132.437243000	4.692843000	9.864167000
8	137.997270000	7.946269000	10.190667000
6	132.575932000	3.308305000	10.464960000
6	133.356154000	2.316826000	9.851531000
6	133.445962000	1.039389000	10.407564000
6	132.749961000	0.725600000	11.578226000
6	131.962141000	1.702357000	12.189292000
6	131.877455000	2.982541000	11.636893000
6	135.773053000	6.189033000	9.284511000
6	135.618995000	7.407118000	10.203591000
6	136.705248000	8.467686000	9.988471000
1	133.874624000	2.547499000	8.926125000
1	134.051305000	0.280622000	9.918174000
1	131.573126000	5.201469000	10.295423000
1	132.249035000	4.608963000	8.785514000
1	132.808876000	-0.275214000	11.998742000
1	131.398426000	1.463881000	13.088533000
1	131.259020000	3.733751000	12.124173000
1	136.571686000	8.863486000	8.968768000
1	136.560802000	9.292527000	10.691307000
1	134.164117000	8.956811000	10.276746000
1	134.466105000	4.495529000	7.666484000
1	138.036071000	7.186065000	9.569837000
8	128.569946000	7.178622000	3.824563000
1	127.804376000	7.603472000	4.284910000
1	128.722527000	7.743646000	3.052442000
1	135.843544000	1.776323000	12.649949000
1	139.704550000	4.709278000	11.573964000
1	135.694795000	5.276038000	17.123342000
1	129.232209000	7.198058000	17.551250000
1	129.256936000	13.567411000	11.767074000
1	124.655832000	10.066294000	5.963318000
1	133.070028000	16.546669000	3.910954000
1	137.654720000	15.112596000	4.520687000
1	141.543579000	13.776913000	10.183130000
1	139.512375000	3.440123000	5.981133000
1	135.020682000	9.344415000	5.787582000
1	129.353790000	4.959299000	4.023085000
1	133.728663000	-1.015373000	4.730290000
1	139.170731000	6.136059000	5.987973000
1	132.357273000	9.006496000	5.382514000
1	129.722883000	2.255292000	3.818640000
1	136.335844000	-0.696848000	5.431507000
1	138.304262000	16.849368000	9.910757000
1	131.782459000	3.318958000	15.391097000

^{2}TS1'$_{B}$:

6	136.102743000	2.003465000	13.475881000
6	137.259753000	2.063788000	12.474427000
8	138.396416000	1.695148000	12.769844000
6	136.568841000	2.007400000	14.940508000
6	137.234056000	3.337809000	15.322190000
6	135.398937000	1.682930000	15.880518000
1	135.400792000	2.828398000	13.292471000
1	137.323150000	1.217275000	15.036773000
1	137.559668000	3.326394000	16.370547000
1	136.540932000	4.180072000	15.186276000
1	138.114990000	3.522511000	14.701021000
1	135.734025000	1.637560000	16.924635000
1	134.616976000	2.453532000	15.810557000
1	134.936433000	0.718961000	15.635727000
7	136.924637000	2.529242000	11.237240000
6	137.854335000	2.529892000	10.112572000
6	137.402127000	1.656222000	8.929827000
6	137.062969000	0.211257000	9.251106000
6	137.735392000	-0.526147000	10.235177000
6	136.058401000	-0.444920000	8.520922000
6	137.419196000	-1.864042000	10.479604000
6	135.734358000	-1.780831000	8.751976000
6	136.419373000	-2.498104000	9.737390000
8	136.065631000	-3.808113000	9.935369000
1	135.950841000	2.734894000	11.051002000
1	138.811266000	2.197274000	10.519184000
1	138.209465000	1.696522000	8.183230000
1	136.544644000	2.129682000	8.435037000
1	138.500539000	-0.056282000	10.846114000
1	135.525109000	0.097947000	7.743723000
1	137.952133000	-2.412557000	11.255353000
1	134.959013000	-2.279428000	8.178171000
1	136.633721000	-4.180439000	10.627603000
6	134.702121000	5.653914000	17.958907000
6	133.514292000	5.652998000	17.008597000
8	133.246580000	6.593538000	16.261785000
6	135.165074000	7.059910000	18.343093000
1	134.460849000	5.059737000	18.849981000
1	134.388693000	7.599226000	18.896246000
1	136.056282000	7.004566000	18.975384000
1	135.408778000	7.641365000	17.450554000
7	132.732047000	4.521053000	17.029942000
6	131.836612000	4.218611000	15.916583000
6	130.665281000	5.186031000	15.734059000
8	130.107252000	5.274924000	14.640253000
1	133.085470000	3.730516000	17.554193000
1	132.374100000	4.216948000	14.962526000
7	130.288050000	5.910142000	16.816882000
6	129.264541000	6.937167000	16.716665000

6	129.577680000	8.085078000	15.741433000
8	128.653932000	8.791096000	15.329843000
1	130.838979000	5.824414000	17.659589000
1	128.322091000	6.502761000	16.372525000
7	130.867308000	8.241454000	15.361048000
6	131.228042000	9.231432000	14.353891000
6	130.563751000	8.972490000	12.994294000
8	130.511880000	9.901187000	12.170485000
6	132.750622000	9.359276000	14.195744000
6	133.477443000	9.916089000	15.429879000
16	132.847089000	11.501944000	16.106932000
6	133.213440000	12.651166000	14.733279000
1	131.588441000	7.608280000	15.701239000
1	130.830022000	10.201364000	14.671520000
1	133.185086000	8.377586000	13.962039000
1	132.933544000	9.994215000	13.323597000
1	134.542330000	10.028037000	15.196252000
1	133.410113000	9.211646000	16.263909000
1	132.878271000	13.639755000	15.058752000
1	132.673673000	12.381550000	13.821167000
1	134.287532000	12.698761000	14.529199000
7	130.002331000	7.769280000	12.773241000
6	129.217424000	7.505261000	11.572962000
6	128.117429000	8.555390000	11.365814000
8	127.731192000	8.821908000	10.213830000
6	128.596346000	6.101134000	11.635326000
1	130.091227000	7.025196000	13.461511000
1	129.868885000	7.578805000	10.697053000
1	128.097051000	5.878947000	10.688821000
1	127.875418000	6.023730000	12.455771000
1	129.375729000	5.351313000	11.795137000
7	127.559298000	9.146953000	12.439555000
6	126.523709000	10.150229000	12.236778000
6	126.993427000	11.440190000	11.541174000
8	126.178847000	12.138807000	10.932165000
1	127.911513000	8.962025000	13.378598000
1	126.104682000	10.412228000	13.212761000
1	125.725001000	9.742983000	11.612083000
7	128.298960000	11.774212000	11.680872000
6	128.871602000	12.911973000	10.985948000
6	129.695839000	12.618117000	9.719881000
8	129.989212000	13.552398000	8.978084000
1	128.930968000	11.132984000	12.152912000
1	128.055532000	13.570748000	10.685912000
7	130.076289000	11.324421000	9.509579000
6	130.988511000	10.966978000	8.428392000
6	130.410496000	9.956762000	7.410216000
8	131.083580000	9.644221000	6.425709000
6	132.304627000	10.381582000	8.969732000
6	133.121434000	11.284207000	9.844963000
6	133.127558000	12.651506000	9.985933000
7	134.100493000	10.733397000	10.659820000
6	134.686833000	11.749610000	11.268168000
7	134.128122000	12.928822000	10.898141000
1	130.022966000	10.682172000	10.296514000
1	131.180324000	11.885134000	7.866685000
1	132.896984000	10.064979000	8.103789000
1	132.074885000	9.471530000	9.534425000
1	132.511410000	13.422618000	9.548261000
1	135.505752000	11.689252000	11.970451000
1	134.436017000	13.843229000	11.205029000
7	129.183049000	9.451012000	7.684183000
6	128.546514000	8.467668000	6.811650000
6	127.102229000	8.826620000	6.422554000
8	126.691744000	8.552632000	5.286530000
6	128.619597000	7.019643000	7.353261000
8	127.685597000	6.725994000	8.373682000
1	128.743479000	9.717879000	8.555945000
1	129.120622000	8.478372000	5.884390000
1	129.659051000	6.841624000	7.675825000
1	128.402611000	6.340291000	6.525670000
1	127.743464000	7.426187000	9.052232000
7	126.324031000	9.430175000	7.347427000
6	124.925828000	9.756993000	7.095809000
6	124.487566000	10.888751000	8.018139000
8	124.606757000	10.455880000	9.365256000
1	126.591960000	9.439613000	8.325973000
1	124.290871000	8.876663000	7.266022000
1	125.110186000	11.774962000	7.830357000
1	123.444477000	11.154098000	7.783545000
1	124.975094000	11.182787000	9.906150000
7	132.719710000	16.433059000	4.779528000
6	133.254110000	16.746767000	6.125173000
6	134.726900000	16.320939000	6.302204000
8	135.315412000	16.568339000	7.360699000
6	132.328072000	15.997012000	7.118224000
6	131.655678000	14.924112000	6.247871000
6	131.467466000	15.659456000	4.918607000
1	133.241560000	17.824380000	6.333959000
1	131.570651000	16.683390000	7.513972000
1	132.887627000	15.597958000	7.967773000
1	132.329776000	14.070754000	6.106416000
1	130.723437000	14.553046000	6.679484000
1	131.337300000	14.983698000	4.065801000
1	130.578774000	16.310143000	4.970267000
7	135.263581000	15.679716000	5.245817000
6	136.649537000	15.241215000	5.153029000
6	136.818936000	13.791425000	4.646383000
6	136.601162000	12.718233000	5.734192000
6	138.228209000	13.632011000	4.050496000
6	135.230432000	12.704709000	6.421149000
1	134.628645000	15.579164000	4.457570000
1	137.103679000	15.351948000	6.140632000
1	136.083737000	13.625379000	3.841695000
1	137.385751000	12.842529000	6.491637000
1	136.770334000	11.737652000	5.264835000
1	138.393258000	14.326052000	3.217395000
1	138.383855000	12.614200000	3.674223000
1	138.988355000	13.830298000	4.815776000
1	135.144044000	11.838622000	7.089502000
1	134.413210000	12.641152000	5.691749000
1	135.073556000	13.602317000	7.026795000
16	135.481565000	4.589865000	2.941460000
1	134.177377000	4.728197000	2.614997000
6	140.863015000	13.921651000	9.042108000
6	139.599495000	14.687284000	8.664603000
8	139.011375000	14.478837000	7.604316000
6	140.506019000	12.534695000	9.615357000
6	141.713186000	11.632173000	9.941339000
6	141.257731000	10.428193000	10.781332000
6	142.448325000	11.166605000	8.675188000
1	141.452230000	13.814405000	8.126889000
1	139.918605000	12.678580000	10.533687000
1	139.848287000	12.018891000	8.903175000
1	142.420336000	12.220272000	10.547568000
1	142.098139000	9.759287000	11.002046000
1	140.831089000	10.752617000	11.738674000
1	140.488637000	9.843880000	10.260885000
1	143.311643000	10.542093000	8.933185000
1	142.818154000	12.006420000	8.076385000
1	141.783814000	10.569111000	8.037697000
7	139.144807000	15.580304000	9.593459000
6	137.864274000	16.264716000	9.456823000
6	136.655287000	15.447736000	9.966373000
6	136.635679000	15.235829000	11.463622000
6	137.332477000	14.170828000	12.062026000
6	135.935514000	16.116820000	12.305505000
6	137.341562000	14.000836000	13.448921000
6	135.938626000	15.948765000	13.692677000
6	136.644634000	14.891126000	14.270603000
1	139.626755000	15.636809000	10.479341000
1	137.698750000	16.472359000	8.399089000
1	135.758337000	15.978271000	9.632168000
1	136.663692000	14.486803000	9.439786000
1	137.867357000	13.466726000	11.429344000
1	135.388001000	16.947450000	11.864824000
1	137.895433000	13.174522000	13.887991000
1	135.390196000	16.645446000	14.321459000
1	136.652014000	14.761572000	15.349433000
7	137.232590000	4.263912000	5.286579000

7	135.138542000	6.193364000	5.556901000
7	135.297315000	2.203015000	4.890310000
6	137.722266000	1.875017000	5.004733000
6	137.542025000	6.677885000	5.614237000
6	132.707517000	6.534591000	5.405607000
6	132.908447000	1.768685000	4.559401000
6	138.092629000	3.199451000	5.187183000
6	136.206815000	7.050467000	5.659521000
6	132.349261000	5.229919000	5.091138000
6	134.234425000	1.372459000	4.653851000
8	135.119106000	3.849036000	6.952612000
6	139.457187000	3.659150000	5.290543000
6	135.736162000	8.407676000	5.811088000
6	130.998323000	4.803359000	4.808178000
6	134.697834000	0.014005000	4.483562000
6	139.405538000	5.010233000	5.447490000
6	134.372797000	8.361151000	5.784247000
6	131.060427000	3.462401000	4.577035000
6	136.051937000	0.043063000	4.615076000
6	138.009476000	5.376934000	5.449428000
6	134.013530000	6.975317000	5.601628000
6	132.442980000	3.070759000	4.709493000
6	136.415902000	1.420253000	4.860891000
7	133.215030000	4.163348000	5.007247000
26	135.220169000	4.185419000	5.272458000
1	138.516894000	1.137823000	4.945142000
1	138.284420000	7.464076000	5.710408000
1	131.920952000	7.281551000	5.460172000
1	132.172316000	1.001048000	4.341509000
6	133.514719000	7.040402000	9.850611000
7	134.561705000	7.897312000	10.034567000
8	132.344838000	7.306957000	10.114597000
16	136.575806000	6.858926000	11.742371000
6	133.857707000	5.757803000	9.131656000
7	135.072455000	5.504333000	8.720027000
8	137.282385000	6.053347000	8.634785000
16	134.987786000	5.998769000	12.564108000
6	132.754822000	4.792620000	8.825625000
8	138.206936000	8.372267000	9.860771000
6	132.680028000	3.702204000	9.894920000
6	133.293384000	2.461584000	9.677542000
6	133.197624000	1.452705000	10.640938000
6	132.489050000	1.678158000	11.824796000
6	131.887280000	2.919416000	12.049436000
6	131.993118000	3.930415000	11.092878000
6	136.166758000	6.326113000	9.051430000
6	135.916078000	7.509930000	9.961834000
6	136.856797000	8.683304000	9.612846000
1	133.829461000	2.287359000	8.747890000
1	133.668562000	0.491261000	10.454695000
1	131.814573000	5.345064000	8.786904000
1	132.959978000	4.336841000	7.852877000
1	132.401214000	0.885852000	12.563678000
1	131.320480000	3.108520000	12.957379000
1	131.540759000	4.901498000	11.270799000
1	136.671781000	8.949509000	8.561031000
1	136.594754000	9.544254000	10.231593000
1	134.352577000	8.843271000	10.414534000
1	135.197742000	4.691636000	7.856546000
1	138.407091000	7.581162000	9.324308000
8	128.588247000	6.972307000	3.937372000
1	127.835509000	7.487625000	4.306253000
1	129.027599000	7.591179000	3.335593000
1	135.549530000	1.075970000	13.268115000
1	137.991704000	3.556256000	9.749139000
1	135.514306000	5.118861000	17.445574000
1	129.093165000	7.356833000	17.711972000
1	129.520563000	13.473765000	11.667968000
1	124.823365000	10.043145000	6.045986000
1	132.589059000	17.270823000	4.221285000
1	137.184857000	15.918929000	4.471573000
1	141.468649000	14.482254000	9.766935000
1	140.327787000	3.017640000	5.240230000
1	136.378647000	9.276334000	5.890468000
1	130.139650000	5.463843000	4.748646000
1	134.056325000	-0.834690000	4.283133000
1	140.225013000	5.709161000	5.556833000
1	133.652188000	9.167527000	5.843663000
1	130.252334000	2.786875000	4.326518000
1	136.755216000	-0.777063000	4.548115000
1	137.930810000	17.219773000	9.989094000
1	131.428535000	3.215131000	16.072574000

^{2}IM1$_{B}$:

6	135.966434000	2.195766000	13.373081000
6	137.184016000	1.903214000	12.488977000
8	138.193101000	1.347983000	12.929867000
6	136.247629000	2.046941000	14.876146000
6	137.197338000	3.137759000	15.391146000
6	134.933564000	2.038086000	15.671151000
1	135.583607000	3.202621000	13.152071000
1	136.744956000	1.079326000	15.014504000
1	137.403042000	3.006899000	16.461478000
1	136.764333000	4.138301000	15.248325000
1	138.151300000	3.098247000	14.858711000
1	135.124200000	1.893293000	16.742722000
1	134.397555000	2.990874000	15.549862000
1	134.264302000	1.235069000	15.338388000
7	137.042022000	2.272041000	11.188387000
6	138.072321000	2.068941000	10.178139000
6	137.653924000	1.130304000	9.036174000
6	137.396423000	-0.329710000	9.373730000
6	137.841563000	-0.943916000	10.549908000
6	136.709879000	-1.127758000	8.442551000
6	137.611070000	-2.302461000	10.785541000
6	136.477260000	-2.482351000	8.662332000
6	136.931867000	-3.077401000	9.844225000
8	136.678133000	-4.413497000	10.023310000
1	136.191701000	2.743629000	10.896155000
1	138.956472000	1.698934000	10.700810000
1	138.445141000	1.191396000	8.273184000
1	136.758981000	1.546748000	8.554437000
1	138.354753000	-0.368726000	11.314372000
1	136.353109000	-0.676264000	7.518660000
1	137.961525000	-2.755251000	11.712349000
1	135.949183000	-3.090945000	7.934238000
1	137.060316000	-4.688217000	10.871257000
6	134.533475000	5.793674000	18.010281000
6	133.312262000	5.812311000	17.104210000
8	133.069546000	6.727298000	16.316898000
6	135.064177000	7.191456000	18.332773000
1	134.304203000	5.235724000	18.927461000
1	134.330887000	7.776045000	18.898797000
1	135.976628000	7.119772000	18.932195000
1	135.294713000	7.735033000	17.413386000
7	132.470883000	4.729370000	17.209847000
6	131.514272000	4.430264000	16.148116000
6	130.409140000	5.469967000	15.954832000
8	129.847584000	5.573733000	14.865135000
1	132.799726000	3.945234000	17.758934000
1	132.011032000	4.339473000	15.176462000
7	130.084995000	6.238356000	17.024179000
6	129.107972000	7.308616000	16.902997000
6	129.448530000	8.384106000	15.856991000
8	128.543909000	9.076989000	15.386511000
1	130.628191000	6.136901000	17.870128000
1	128.132947000	6.907213000	16.614165000
7	130.748758000	8.507061000	15.497539000
6	131.138815000	9.442120000	14.450295000
6	130.524884000	9.112340000	13.083720000
8	130.515370000	9.996993000	12.210172000
6	132.664754000	9.568091000	14.336027000
6	133.342683000	10.223403000	15.547849000
16	132.711936000	11.879197000	16.029861000
6	133.116775000	12.858888000	14.540177000
1	131.448259000	7.860113000	15.855770000
1	130.727423000	10.425128000	14.703940000
1	133.110288000	8.575442000	14.191389000
1	132.876864000	10.136963000	13.425809000
1	134.420195000	10.293453000	15.361248000

1	133.215410000	9.605334000	16.442089000
1	132.808658000	13.886136000	14.752918000
1	132.572540000	12.505484000	13.659870000
1	134.192626000	12.853421000	14.340283000
7	129.963881000	7.904209000	12.902263000
6	129.252473000	7.583556000	11.671776000
6	128.118645000	8.579980000	11.394185000
8	127.745144000	8.771970000	10.222346000
6	128.718958000	6.144747000	11.714611000
1	130.005370000	7.196041000	13.632176000
1	129.944855000	7.680112000	10.831309000
1	128.260418000	5.892831000	10.754738000
1	127.983915000	6.013975000	12.515315000
1	129.547741000	5.453831000	11.893499000
7	127.536279000	9.223112000	12.424110000
6	126.508011000	10.218807000	12.156625000
6	127.005523000	11.496770000	11.456290000
8	126.209205000	12.198133000	10.825895000
1	127.873990000	9.090153000	13.376756000
1	126.045595000	10.499192000	13.107463000
1	125.738713000	9.796105000	11.505896000
7	128.312963000	11.813788000	11.609429000
6	128.920214000	12.920873000	10.894651000
6	129.753713000	12.571687000	9.648161000
8	130.065787000	13.473328000	8.874620000
1	128.928124000	11.174628000	12.105633000
1	128.123750000	13.588583000	10.563453000
7	130.119397000	11.266470000	9.488188000
6	130.996240000	10.853778000	8.398313000
6	130.332906000	9.933203000	7.349805000
8	130.909043000	9.740725000	6.277273000
6	132.265727000	10.153933000	8.924744000
6	133.158152000	11.018677000	9.766872000
6	133.233827000	12.387476000	9.873549000
7	134.126104000	10.441908000	10.575394000
6	134.771969000	11.442795000	11.148725000
7	134.264267000	12.638396000	10.759096000
1	130.065545000	10.657057000	10.300352000
1	131.266581000	11.761526000	7.853263000
1	132.821289000	9.783566000	8.055146000
1	131.984115000	9.268683000	9.508226000
1	132.648273000	13.177910000	9.428327000
1	135.599588000	11.358942000	11.838408000
1	134.618367000	13.544167000	11.040918000
7	129.157602000	9.351938000	7.696771000
6	128.496398000	8.387172000	6.822375000
6	127.045299000	8.744086000	6.459188000
8	126.612263000	8.447063000	5.336460000
6	128.578717000	6.934214000	7.347646000
8	127.674127000	6.635611000	8.394596000
1	128.785128000	9.528623000	8.622452000
1	129.050911000	8.411095000	5.883470000
1	129.625681000	6.744575000	7.634967000
1	128.330508000	6.262190000	6.522128000
1	127.748680000	7.330900000	9.075691000
7	126.282745000	9.361955000	7.386183000
6	124.878186000	9.673767000	7.151123000
6	124.448254000	10.850431000	8.018510000
8	124.568232000	10.488779000	9.386462000
1	126.581972000	9.411312000	8.354105000
1	124.250310000	8.801489000	7.379683000
1	125.071715000	11.724794000	7.784243000
1	123.404837000	11.106117000	7.774838000
1	124.963407000	11.234339000	9.881835000
7	132.602411000	16.921438000	4.969645000
6	133.223153000	17.131306000	6.298736000
6	134.679528000	16.622880000	6.368958000
8	135.326225000	16.747607000	7.415122000
6	132.314964000	16.373854000	7.300121000
6	131.576620000	15.357623000	6.416023000
6	131.345159000	16.157630000	5.131299000
1	133.278803000	18.195379000	6.562369000
1	131.596044000	17.069212000	7.749609000
1	132.894929000	15.926783000	8.110996000
1	132.222082000	14.496602000	6.206131000
1	130.657647000	14.981980000	6.871083000
1	131.166122000	15.523541000	4.255586000
1	130.470985000	16.818689000	5.250258000
7	135.137329000	16.057467000	5.235178000
6	136.489375000	15.552946000	5.038065000
6	136.547356000	14.134993000	4.426743000
6	136.335217000	13.003825000	5.455465000
6	137.906368000	13.942459000	3.732347000
6	135.015558000	13.026469000	6.235658000
1	134.459224000	16.047886000	4.476971000
1	136.997719000	15.568077000	6.005205000
1	135.756494000	14.063998000	3.661884000
1	137.174254000	13.031491000	6.162279000
1	136.410171000	12.049549000	4.913845000
1	138.061247000	14.682688000	2.937862000
1	137.979671000	12.946171000	3.280947000
1	138.722036000	14.044666000	4.458369000
1	134.921007000	12.130202000	6.861453000
1	134.148631000	13.050976000	5.563518000
1	134.955307000	13.895002000	6.898299000
16	135.640953000	4.953757000	3.017551000
1	135.635591000	6.302623000	2.932284000
6	140.826749000	13.663323000	8.645546000
6	139.585072000	14.506078000	8.372826000
8	138.942651000	14.390207000	7.330155000
6	140.434243000	12.263193000	9.161234000
6	141.612290000	11.284502000	9.344097000
6	141.166964000	10.064322000	10.165873000
6	142.213006000	10.845904000	7.999797000
1	141.365446000	13.581254000	7.697208000
1	139.919831000	12.379561000	10.126127000
1	139.702339000	11.825874000	8.468834000
1	142.398133000	11.805656000	9.913528000
1	141.992026000	9.353674000	10.294804000
1	140.826771000	10.362499000	11.165299000
1	140.338408000	9.530622000	9.683703000
1	143.061449000	10.168992000	8.154208000
1	142.573277000	11.694699000	7.407795000
1	141.466344000	10.312496000	7.397110000
7	139.212971000	15.359274000	9.373164000
6	137.960544000	16.105639000	9.334978000
6	136.736532000	15.307585000	9.839316000
6	136.756474000	15.017087000	11.323413000
6	137.435751000	13.901110000	11.844319000
6	136.115689000	15.877781000	12.231026000
6	137.486125000	13.662917000	13.220458000
6	136.160755000	15.642064000	13.607136000
6	136.849375000	14.534670000	14.108171000
1	139.735650000	15.342363000	10.237318000
1	137.764424000	16.393443000	8.301508000
1	135.851675000	15.889918000	9.565000000
1	136.692422000	14.376988000	9.262032000
1	137.924216000	13.211873000	11.159831000
1	135.581407000	16.745567000	11.850100000
1	138.025398000	12.798036000	13.599261000
1	135.658616000	16.324484000	14.288002000
1	136.889530000	14.352753000	15.178695000
7	137.202454000	4.978616000	5.660258000
7	134.984929000	6.763999000	5.409428000
7	135.485002000	2.799908000	4.974734000
6	137.914563000	2.650878000	5.337145000
6	137.301921000	7.415676000	5.931008000
6	132.549965000	6.916803000	5.083761000
6	133.149927000	2.139920000	4.592620000
6	138.153750000	3.988003000	5.618630000
6	135.959062000	7.688403000	5.707274000
6	132.305324000	5.565134000	4.900683000
6	134.506472000	1.872527000	4.720593000
8	134.932207000	4.544776000	7.050801000
6	139.455768000	4.547540000	5.880022000
6	135.375179000	9.004735000	5.786054000
6	131.005090000	5.015400000	4.590656000
6	135.091987000	0.560977000	4.608775000
6	139.283291000	5.887848000	6.053254000
6	134.038843000	8.864728000	5.553698000

6	131.176734000	3.670953000	4.469380000
6	136.433480000	0.707930000	4.804830000
6	137.875024000	6.151600000	5.895660000
6	133.803186000	7.460582000	5.329133000
6	132.576342000	3.396740000	4.692341000
6	136.670783000	2.108824000	5.044396000
7	133.244159000	4.564589000	4.966969000
26	135.209074000	4.773809000	5.283834000
1	138.765008000	1.976750000	5.342879000
1	137.955895000	8.257675000	6.135283000
1	131.708939000	7.601147000	5.046417000
1	132.492310000	1.304118000	4.376658000
6	133.467124000	6.688310000	10.547198000
7	134.529871000	7.541469000	10.505374000
8	132.300192000	7.059608000	10.618963000
16	136.723655000	7.101711000	12.270361000
6	133.813745000	5.205100000	10.481235000
7	135.002155000	4.764166000	10.251690000
8	137.024569000	5.326069000	9.378714000
16	135.274947000	6.610693000	13.522470000
6	132.640235000	4.277803000	10.655150000
8	138.133347000	7.830850000	9.817828000
6	132.896033000	2.803884000	10.442342000
6	133.351344000	2.326027000	9.203281000
6	133.544142000	0.958618000	9.007392000
6	133.286159000	0.049662000	10.037579000
6	132.822876000	0.516822000	11.267389000
6	132.630052000	1.887228000	11.465524000
6	136.020414000	5.688561000	9.975879000
6	135.874621000	7.123872000	10.451165000
6	136.760885000	8.099729000	9.651392000
1	133.574948000	3.022456000	8.398364000
1	133.901021000	0.603452000	8.044767000
1	132.215219000	4.466449000	11.651132000
1	131.869943000	4.649522000	9.963654000
1	133.453828000	-1.012047000	9.881515000
1	132.610087000	-0.181757000	12.072649000
1	132.262491000	2.246665000	12.425096000
1	136.447727000	8.040212000	8.599465000
1	136.576198000	9.117060000	10.005155000
1	134.335830000	8.563211000	10.590746000
1	135.801608000	4.439038000	7.480590000
1	138.273158000	6.936538000	9.452234000
8	128.550499000	7.103516000	3.826396000
1	127.780256000	7.522768000	4.274690000
1	128.907149000	7.806583000	3.263633000
1	135.172975000	1.499686000	13.067540000
1	138.323716000	3.041745000	9.740364000
1	135.304470000	5.213270000	17.483023000
1	128.996318000	7.788434000	17.879629000
1	129.570438000	13.486460000	11.572282000
1	124.754062000	9.903267000	6.089879000
1	132.451975000	17.799055000	4.482411000
1	137.027803000	16.247452000	4.376493000
1	141.492737000	14.154702000	9.367635000
1	140.374577000	3.975789000	5.910695000
1	135.931494000	9.909379000	5.996250000
1	130.106820000	5.608754000	4.450792000
1	134.535427000	-0.344298000	4.402036000
1	140.031711000	6.644441000	6.252549000
1	133.254784000	9.610931000	5.552407000
1	130.436868000	2.917417000	4.230493000
1	137.204135000	-0.052342000	4.796242000
1	138.090266000	17.018370000	9.926330000
1	131.044261000	3.468387000	16.375584000

^{2}TS2$_{B}$:

6	136.505101000	2.411992000	13.815570000
6	137.822829000	2.386468000	13.038714000
8	138.754607000	1.659296000	13.387397000
6	136.723045000	2.371530000	15.338510000
6	137.413937000	3.647583000	15.841180000
6	135.397781000	2.132642000	16.075479000
1	135.892895000	3.279410000	13.536245000
1	137.391804000	1.525457000	15.537280000
1	137.581199000	3.604735000	16.924857000
1	136.804794000	4.537449000	15.626223000
1	138.388614000	3.784305000	15.361843000
1	135.559149000	2.058073000	17.158561000
1	134.692844000	2.957329000	15.894606000
1	134.914764000	1.205018000	15.745329000
7	137.868995000	3.185728000	11.938863000
6	139.032512000	3.291766000	11.066912000
6	138.768169000	2.863596000	9.615627000
6	138.417750000	1.404894000	9.367784000
6	138.663779000	0.379355000	10.288198000
6	137.852003000	1.048543000	8.131489000
6	138.359003000	-0.951228000	9.984034000
6	137.547131000	-0.272305000	7.813114000
6	137.805937000	-1.284095000	8.745519000
8	137.493941000	-2.571651000	8.391740000
1	137.089385000	3.804044000	11.751007000
1	139.822149000	2.695486000	11.528510000
1	139.673857000	3.118241000	9.043486000
1	137.974375000	3.497811000	9.198307000
1	139.077424000	0.600688000	11.267459000
1	137.650653000	1.823167000	7.394399000
1	138.557379000	-1.730576000	10.718897000
1	137.111871000	-0.530780000	6.852405000
1	137.753486000	-3.163075000	9.115322000
6	134.573340000	6.127503000	18.201176000
6	133.387989000	6.109418000	17.246770000
8	133.126148000	7.035793000	16.480979000
6	135.021826000	7.541723000	18.573150000
1	134.334635000	5.540388000	19.097553000
1	134.239946000	8.076327000	19.122958000
1	135.914550000	7.502074000	19.204439000
1	135.256215000	8.119177000	17.675499000
7	132.604277000	4.979879000	17.288141000
6	131.693354000	4.665035000	16.191542000
6	130.505368000	5.615734000	16.023303000
8	129.908817000	5.660052000	14.948053000
1	132.945869000	4.201411000	17.837083000
1	132.215858000	4.674239000	15.228769000
7	130.164250000	6.380749000	17.089436000
6	129.145461000	7.412378000	16.974918000
6	129.432081000	8.499018000	15.922745000
8	128.497986000	9.173513000	15.484739000
1	130.742528000	6.329752000	17.916241000
1	128.185462000	6.971200000	16.694149000
7	130.715106000	8.637117000	15.512255000
6	131.055788000	9.554798000	14.431893000
6	130.352208000	9.219257000	13.110458000
8	130.280004000	10.101459000	12.238174000
6	132.574636000	9.640072000	14.216350000
6	133.345130000	10.327694000	15.353619000
16	132.751076000	11.996569000	15.838562000
6	132.960808000	12.912123000	14.269507000
1	131.444504000	8.030938000	15.881272000
1	130.683845000	10.550503000	14.696503000
1	132.987594000	8.631586000	14.081406000
1	132.739352000	10.169722000	13.273207000
1	134.403261000	10.393814000	15.076483000
1	133.291967000	9.733616000	16.271028000
1	132.676105000	13.947580000	14.475075000
1	132.313581000	12.519228000	13.480141000
1	134.004033000	12.896477000	13.941012000
7	129.793926000	8.004304000	12.971434000
6	129.026934000	7.652840000	11.783316000
6	127.899934000	8.655544000	11.515655000
8	127.509576000	8.842461000	10.348179000
6	128.464242000	6.229457000	11.908637000
1	129.902723000	7.300911000	13.698461000
1	129.686938000	7.700999000	10.914456000
1	127.989750000	5.939454000	10.967673000
1	127.737620000	6.155094000	12.724338000
1	129.279430000	5.529610000	12.114184000
7	127.327575000	9.299749000	12.550534000
6	126.281210000	10.279126000	12.290710000
6	126.747977000	11.562533000	11.579630000

8	125.935785000	12.239635000	10.943274000	1	135.244011000	11.576887000	7.447275000
1	127.690404000	9.180884000	13.495562000	1	134.575483000	12.341194000	5.996360000
1	125.827001000	10.557892000	13.246124000	1	135.357662000	13.326779000	7.241056000
1	125.510770000	9.840137000	11.652568000	16	135.020652000	4.947043000	2.454053000
7	128.045547000	11.915736000	11.740150000	1	134.576912000	6.222499000	2.403877000
6	128.618284000	13.055486000	11.048545000	6	141.054456000	12.926697000	9.236047000
6	129.424529000	12.765796000	9.769678000	6	139.915783000	13.849496000	8.815564000
8	129.705648000	13.703402000	9.026547000	8	139.369842000	13.736480000	7.718692000
1	128.680230000	11.282281000	12.218750000	6	140.515386000	11.527103000	9.596658000
1	127.804229000	13.721281000	10.758476000	6	141.597332000	10.475098000	9.913927000
7	129.797042000	11.473102000	9.548271000	6	140.960390000	9.241064000	10.573278000
6	130.609191000	11.106254000	8.394190000	6	142.396992000	10.072336000	8.665327000
6	129.894066000	10.210281000	7.360747000	1	141.736764000	12.860515000	8.382993000
8	130.328873000	10.165907000	6.210532000	1	139.852065000	11.623491000	10.468712000
6	131.910717000	10.390894000	8.807746000	1	139.889971000	11.169730000	8.768182000
6	132.869007000	11.219924000	9.611434000	1	142.299120000	10.919628000	10.637512000
6	132.928193000	12.577598000	9.819785000	1	141.715541000	8.474972000	10.786701000
7	133.930884000	10.608508000	10.261232000	1	140.477023000	9.503619000	11.522531000
6	134.614212000	11.577747000	10.843053000	1	140.195180000	8.787392000	9.931207000
7	134.044538000	12.786513000	10.608061000	1	143.177003000	9.345451000	8.920400000
1	129.768155000	10.828210000	10.333453000	1	142.888442000	10.929514000	8.191553000
1	130.839337000	12.032582000	7.864700000	1	141.741006000	9.608167000	7.917631000
1	132.402605000	10.051010000	7.887969000	7	139.522509000	14.776212000	9.738926000
1	131.668223000	9.485021000	9.376737000	6	138.345176000	15.615354000	9.546219000
1	132.284216000	13.383391000	9.500306000	6	137.020532000	14.945430000	9.975170000
1	135.510391000	11.463586000	11.435794000	6	136.886890000	14.730549000	11.465942000
1	134.404942000	13.675135000	10.930879000	6	137.437477000	13.600258000	12.096146000
7	128.859122000	9.451758000	7.808201000	6	136.225780000	15.673706000	12.270712000
6	128.260114000	8.421011000	6.966578000	6	137.339182000	13.423615000	13.478620000
6	126.778542000	8.644121000	6.622834000	6	136.123513000	15.500354000	13.653337000
8	126.328303000	8.195635000	5.558272000	6	136.681582000	14.374627000	14.263640000
6	128.464666000	6.996153000	7.533797000	1	139.953290000	14.756105000	10.652230000
8	127.569729000	6.649089000	8.576339000	1	138.268941000	15.853661000	8.484553000
1	128.576124000	9.522556000	8.779374000	1	136.213071000	15.578546000	9.595033000
1	128.794930000	8.470643000	6.016596000	1	136.947199000	13.992529000	9.439101000
1	129.518372000	6.912559000	7.846588000	1	137.945247000	12.851888000	11.491959000
1	128.294337000	6.280309000	6.726489000	1	135.793382000	16.556741000	11.804657000
1	127.558771000	7.375674000	9.228290000	1	137.776618000	12.543175000	13.942960000
7	126.016196000	9.316636000	7.509000000	1	135.607656000	16.245322000	14.253672000
6	124.587706000	9.527807000	7.318272000	1	136.601882000	14.237842000	15.338531000
6	124.128279000	10.725413000	8.142943000	7	136.661459000	5.103878000	5.057921000
8	124.342150000	10.447374000	9.519082000	7	134.184630000	6.516857000	4.954890000
1	126.359628000	9.500437000	8.445081000	7	135.295740000	2.706410000	4.312250000
1	124.024328000	8.635018000	7.623023000	6	137.720570000	2.923745000	4.660719000
1	124.682367000	11.620801000	7.827817000	6	136.387524000	7.516689000	5.406641000
1	123.059671000	10.903805000	7.944745000	6	131.746232000	6.286246000	4.717632000
1	124.720036000	11.232752000	9.963261000	6	133.086100000	1.704218000	3.917845000
7	133.215252000	16.094518000	4.797696000	6	137.751695000	4.272816000	4.982517000
6	133.816557000	16.534076000	6.078743000	6	135.010290000	7.576709000	5.244303000
6	135.234650000	15.963306000	6.304415000	6	131.715017000	4.920937000	4.476444000
8	135.841971000	16.214525000	7.351412000	6	134.470171000	1.651203000	4.013532000
6	132.834401000	16.066251000	7.179229000	8	134.529372000	4.242590000	6.476776000
6	132.068483000	14.916361000	6.506423000	6	138.956874000	5.020314000	5.241925000
6	131.923002000	15.422359000	5.067583000	6	134.228677000	8.779322000	5.390951000
1	133.941207000	17.624133000	6.115739000	6	130.506612000	4.185050000	4.185642000
1	132.141960000	16.878738000	7.429514000	6	135.251999000	0.454475000	3.835546000
1	133.361704000	15.783267000	8.093489000	6	138.583431000	6.313955000	5.447170000
1	132.670729000	13.999860000	6.519907000	6	132.922702000	8.435487000	5.207917000
1	131.110065000	14.697418000	6.985051000	6	130.884199000	2.896276000	3.963308000
1	131.750148000	14.616034000	4.346269000	6	136.555200000	0.797276000	4.042129000
1	131.073786000	16.120997000	4.998631000	6	137.148550000	6.360037000	5.316182000
7	135.711979000	15.216789000	5.289408000	6	132.903477000	7.017989000	4.943167000
6	137.047729000	14.638801000	5.231751000	6	132.319635000	2.842240000	4.113637000
6	137.070409000	13.144389000	4.839808000	6	136.573640000	2.205384000	4.349155000
6	136.760881000	12.187408000	6.010525000	7	132.801648000	4.083773000	4.442114000
6	138.449925000	12.799835000	4.252992000	26	134.722988000	4.593757000	4.727710000
6	135.409244000	12.372050000	6.709581000	1	138.665979000	2.391186000	4.638607000
1	135.062185000	15.110463000	4.513955000	1	136.907936000	8.446838000	5.613366000
1	137.518704000	14.781913000	6.207273000	1	130.795742000	6.811130000	4.713813000
1	136.313091000	12.989950000	4.053475000	1	132.566446000	0.785966000	3.663453000
1	137.567373000	12.286324000	6.747847000	6	133.027852000	6.944480000	10.421013000
1	136.821427000	11.160408000	5.619142000	7	134.148639000	7.680899000	10.164934000
1	138.674023000	13.407141000	3.367583000	8	131.885732000	7.383830000	10.466012000
1	138.498265000	11.745566000	3.956183000	16	135.998621000	6.461785000	11.899653000
1	139.234617000	12.978046000	4.998015000	6	133.369922000	5.469930000	10.674047000

7	134.293610000	4.974287000	9.736648000
8	136.239185000	5.377458000	8.563241000
16	134.148988000	5.624781000	12.476214000
6	132.169487000	4.505247000	10.737995000
8	137.789800000	7.380016000	9.666678000
6	132.746379000	3.155977000	10.395338000
6	133.485444000	3.114361000	9.160841000
6	134.373176000	2.029157000	8.914025000
6	134.477349000	0.996245000	9.831173000
6	133.693166000	1.012334000	10.997948000
6	132.849186000	2.102243000	11.284312000
6	135.356918000	5.693403000	9.378453000
6	135.420761000	7.047392000	10.139588000
6	136.513571000	7.963792000	9.568275000
1	133.163330000	3.680002000	8.290473000
1	134.917959000	2.013845000	7.977249000
1	131.679715000	4.527699000	11.715227000
1	131.436598000	4.845084000	9.995901000
1	135.147736000	0.165093000	9.635811000
1	133.764834000	0.191565000	11.706453000
1	132.313854000	2.136983000	12.229797000
1	136.233680000	8.183197000	8.527661000
1	136.530390000	8.902435000	10.128553000
1	134.086707000	8.719730000	10.204052000
1	135.302624000	4.575762000	6.981146000
1	137.736598000	6.578612000	9.107049000
8	128.297010000	6.790327000	4.128910000
1	127.528964000	7.221687000	4.572279000
1	128.465371000	7.344744000	3.352643000
1	135.942764000	1.520074000	13.504730000
1	139.371668000	4.335790000	11.059549000
1	135.392417000	5.594190000	17.697044000
1	129.020899000	7.887777000	17.951987000
1	129.274567000	13.607378000	11.731343000
1	124.402172000	9.690083000	6.253071000
1	133.105642000	16.869658000	4.151825000
1	137.640842000	15.205490000	4.498869000
1	141.618609000	13.342422000	10.081643000
1	139.952951000	4.596048000	5.246823000
1	134.642217000	9.754484000	5.614094000
1	129.523523000	4.639041000	4.145535000
1	134.841052000	-0.516046000	3.588028000
1	139.208415000	7.172767000	5.656920000
1	132.041446000	9.062562000	5.270932000
1	130.264423000	2.046370000	3.706194000
1	137.434709000	0.167515000	3.995915000
1	138.498180000	16.548867000	10.098479000
1	131.302622000	3.655668000	16.352151000

^{2}IM2$_B$:

6	136.781169000	2.663024000	13.786423000
6	138.183597000	2.779545000	13.185304000
8	139.115934000	2.074955000	13.573854000
6	136.796263000	2.233364000	15.262446000
6	137.452971000	3.297914000	16.153014000
6	135.377549000	1.901639000	15.745954000
1	136.226445000	3.603581000	13.665721000
1	137.408367000	1.325519000	15.322781000
1	137.454240000	2.987387000	17.205385000
1	136.916145000	4.255449000	16.082382000
1	138.491252000	3.470088000	15.854216000
1	135.389525000	1.542151000	16.782876000
1	134.730832000	2.790282000	15.703322000
1	134.911426000	1.125932000	15.126824000
7	138.303624000	3.690541000	12.180043000
6	139.532849000	3.895616000	11.428111000
6	139.433268000	3.491540000	9.947401000
6	139.107901000	2.037311000	9.648414000
6	139.499506000	0.979046000	10.477910000
6	138.415308000	1.718240000	8.468844000
6	139.216171000	-0.345953000	10.139023000
6	138.125728000	0.401426000	8.117832000
6	138.533410000	-0.641384000	8.956145000
8	138.229716000	-1.922247000	8.574203000
1	137.502722000	4.263540000	11.941103000
1	140.310428000	3.331772000	11.947168000
1	140.392256000	3.762112000	9.478592000
1	138.677434000	4.121872000	9.461432000
1	139.998444000	1.174282000	11.421724000
1	138.093122000	2.518508000	7.805930000
1	139.524775000	-1.150573000	10.805192000
1	137.579169000	0.171015000	7.208463000
1	138.583963000	-2.535629000	9.236750000
6	134.527886000	5.906092000	18.079711000
6	133.409645000	5.866455000	17.047718000
8	133.159555000	6.800770000	16.287572000
6	134.955596000	7.327493000	18.446847000
1	134.221974000	5.342994000	18.971320000
1	134.137856000	7.880060000	18.921259000
1	135.797886000	7.300378000	19.144636000
1	135.260663000	7.880438000	17.554964000
7	132.668074000	4.707839000	17.016293000
6	131.828481000	4.394090000	15.863451000
6	130.633081000	5.328417000	15.657998000
8	130.108433000	5.420852000	14.548088000
1	133.010755000	3.925445000	17.559261000
1	132.403957000	4.428629000	14.932545000
7	130.204526000	6.018928000	16.743708000
6	129.152948000	7.017696000	16.649878000
6	129.444643000	8.206093000	15.716665000
8	128.505660000	8.910233000	15.338749000
1	130.733802000	5.924547000	17.599167000
1	128.232344000	6.565166000	16.270793000
7	130.726829000	8.388204000	15.323414000
6	131.057345000	9.376468000	14.303206000
6	130.352240000	9.115736000	12.964600000
8	130.283012000	10.045461000	12.142655000
6	132.574877000	9.497327000	14.097402000
6	133.326403000	10.159501000	15.261516000
16	132.711903000	11.810714000	15.781846000
6	132.901154000	12.759789000	14.229943000
1	131.462473000	7.766688000	15.652450000
1	130.671488000	10.348984000	14.628413000
1	133.008336000	8.503178000	13.926700000
1	132.733213000	10.064418000	13.175251000
1	134.387030000	10.241447000	14.998797000
1	133.266863000	9.541162000	16.162539000
1	132.611691000	13.788776000	14.460017000
1	132.248501000	12.378930000	13.439091000
1	133.941129000	12.758046000	13.890726000
7	129.776957000	7.917007000	12.765462000
6	128.974832000	7.642699000	11.580085000
6	127.869759000	8.686017000	11.385515000
8	127.462828000	8.940692000	10.236647000
6	128.370838000	6.232472000	11.655493000
1	129.913870000	7.164583000	13.436437000
1	129.615117000	7.711511000	10.697789000
1	127.858653000	6.002620000	10.717580000
1	127.665604000	6.143234000	12.488589000
1	129.167349000	5.497360000	11.800851000
7	127.329758000	9.290307000	12.460595000
6	126.302578000	10.304486000	12.263475000
6	126.784447000	11.598387000	11.581404000
8	125.974340000	12.314876000	10.986737000
1	127.698725000	9.111263000	13.394065000
1	125.882891000	10.561943000	13.240393000
1	125.503041000	9.908103000	11.633131000
7	128.093488000	11.914790000	11.721236000
6	128.679730000	13.060421000	11.051079000
6	129.460810000	12.790787000	9.751943000
8	129.750921000	13.744724000	9.033234000
1	128.722302000	11.254115000	12.169965000
1	127.875594000	13.750688000	10.792111000
7	129.803602000	11.498393000	9.485260000
6	130.585947000	11.150915000	8.303943000
6	129.829353000	10.309483000	7.253304000
8	130.246696000	10.287204000	6.096078000
6	131.878413000	10.393193000	8.667500000
6	132.869309000	11.174234000	9.479585000
6	132.946234000	12.519267000	9.754008000

7	133.941767000	10.523267000	10.071236000
6	134.647266000	11.456852000	10.684402000
7	134.082986000	12.679869000	10.524175000
1	129.772656000	10.829846000	10.250342000
1	130.828400000	12.089575000	7.802235000
1	132.345405000	10.073610000	7.727801000
1	131.628157000	9.473970000	9.211339000
1	132.303480000	13.345556000	9.489102000
1	135.554919000	11.305701000	11.250787000
1	134.457848000	13.548245000	10.883886000
7	128.775406000	9.572755000	7.692297000
6	128.123900000	8.594873000	6.827060000
6	126.649751000	8.891899000	6.507856000
8	126.169492000	8.500175000	5.434087000
6	128.269207000	7.145935000	7.350171000
8	127.369446000	6.809909000	8.392447000
1	128.508315000	9.623051000	8.669220000
1	128.650915000	8.649656000	5.873167000
1	129.320917000	7.003219000	7.646942000
1	128.056903000	6.462389000	6.525341000
1	127.406387000	7.512362000	9.069124000
7	125.924869000	9.562946000	7.425890000
6	124.501641000	9.827180000	7.263629000
6	124.088844000	10.988834000	8.161108000
8	124.316118000	10.628488000	9.515718000
1	126.283963000	9.696025000	8.364581000
1	123.914419000	8.935500000	7.523315000
1	124.663324000	11.884990000	7.887087000
1	123.022915000	11.207441000	7.990576000
1	124.721326000	11.378749000	9.995226000
7	133.300606000	16.146939000	4.822077000
6	133.915859000	16.572323000	6.101305000
6	135.325283000	15.976625000	6.317327000
8	135.943523000	16.214544000	7.360958000
6	132.931776000	16.117517000	7.205538000
6	132.146117000	14.980237000	6.534092000
6	132.000737000	15.491422000	5.097029000
1	134.059190000	17.659949000	6.140822000
1	132.252662000	16.939950000	7.459907000
1	133.458508000	15.825369000	8.117217000
1	132.735319000	14.055269000	6.542945000
1	131.186572000	14.773739000	7.016147000
1	131.813341000	14.689153000	4.374832000
1	131.160844000	16.201674000	5.033712000
7	135.783277000	15.223998000	5.297786000
6	137.107702000	14.621492000	5.232895000
6	137.101964000	13.137190000	4.804238000
6	136.761739000	12.158372000	5.948145000
6	138.478970000	12.778058000	4.220310000
6	135.404839000	12.349789000	6.635173000
1	135.128420000	15.133518000	4.524570000
1	137.575690000	14.730700000	6.214307000
1	136.348618000	13.018599000	4.007851000
1	137.561073000	12.225095000	6.696773000
1	136.808838000	11.139808000	5.533264000
1	138.724538000	13.403550000	3.353469000
1	138.507147000	11.731177000	3.895965000
1	139.260632000	12.918902000	4.976488000
1	135.216811000	11.540780000	7.351724000
1	134.579611000	12.349884000	5.911394000
1	135.361963000	13.292858000	7.187755000
16	134.680502000	4.730327000	2.568010000
1	134.470927000	6.059035000	2.438074000
6	141.072763000	12.763612000	9.241724000
6	139.943105000	13.700824000	8.828220000
8	139.389476000	13.594996000	7.734342000
6	140.523465000	11.360313000	9.569920000
6	141.597333000	10.297487000	9.878541000
6	140.948328000	9.053459000	10.506615000
6	142.409739000	9.915809000	8.631549000
1	141.763015000	12.709905000	8.394020000
1	139.851377000	11.443407000	10.436662000
1	139.905224000	11.022608000	8.727979000
1	142.292508000	10.724449000	10.618827000
1	141.698628000	8.281217000	10.715253000
1	140.453230000	9.299728000	11.454113000
1	140.190373000	8.614365000	9.845966000
1	143.184074000	9.181014000	8.881205000
1	142.909867000	10.780138000	8.180461000
1	141.760781000	9.469167000	7.867206000
7	139.567336000	14.632609000	9.753073000
6	138.406056000	15.494597000	9.561248000
6	137.065060000	14.838198000	9.958430000
6	136.904495000	14.597845000	11.442622000
6	137.434782000	13.451964000	12.062191000
6	136.235157000	15.531109000	12.252226000
6	137.308802000	13.250599000	13.438933000
6	136.105394000	15.333117000	13.629243000
6	136.643304000	14.191962000	14.228892000
1	140.003339000	14.608683000	10.663688000
1	138.347289000	15.756847000	8.503994000
1	136.273521000	15.491231000	9.578567000
1	136.985275000	13.896933000	9.403078000
1	137.949152000	12.711327000	11.454080000
1	135.817963000	16.425716000	11.794502000
1	137.730419000	12.358231000	13.895022000
1	135.583653000	16.070804000	14.233444000
1	136.542027000	14.035939000	15.299337000
7	136.341620000	4.990680000	5.142737000
7	133.951339000	6.543502000	4.934117000
7	134.821596000	2.633835000	4.588537000
6	137.265610000	2.733681000	4.852904000
6	136.208457000	7.430856000	5.360450000
6	131.507911000	6.451594000	4.674161000
6	132.548769000	1.736229000	4.339028000
6	137.382389000	4.095882000	5.091233000
6	134.839152000	7.564746000	5.177250000
6	131.390476000	5.074624000	4.564161000
6	133.929628000	1.607422000	4.407375000
8	134.194173000	4.381083000	6.638183000
6	138.631906000	4.786041000	5.291701000
6	134.134643000	8.821440000	5.235253000
6	130.138033000	4.392326000	4.335793000
6	134.636608000	0.356544000	4.306257000
6	138.334541000	6.107516000	5.441806000
6	132.813203000	8.551301000	5.041258000
6	130.429931000	3.065147000	4.261344000
6	135.963295000	0.639358000	4.436886000
6	136.902739000	6.228604000	5.329511000
6	132.706881000	7.123761000	4.863883000
6	131.857987000	2.933682000	4.430254000
6	136.070840000	2.064045000	4.626270000
7	132.420232000	4.169534000	4.633576000
26	134.365308000	4.578289000	4.857932000
1	138.178107000	2.146237000	4.843384000
1	136.782525000	8.339248000	5.516255000
1	130.594430000	7.033821000	4.604422000
1	131.968925000	0.832774000	4.179655000
6	132.988970000	6.923022000	10.372908000
7	134.126515000	7.603431000	10.038952000
8	131.857226000	7.386333000	10.345298000
16	135.930182000	6.561442000	11.949061000
6	133.293324000	5.501845000	10.848430000
7	134.239605000	4.841245000	9.987136000
8	136.162618000	5.081942000	8.751861000
16	134.042985000	5.841559000	12.594569000
6	132.092710000	4.546127000	10.945740000
8	137.768894000	7.188106000	9.631538000
6	132.720089000	3.215417000	10.642952000
6	133.858164000	3.424855000	9.676294000
6	134.927982000	2.376124000	9.772180000
6	134.653005000	1.178624000	10.374551000
6	133.432131000	0.945678000	11.064024000
6	132.512738000	2.010609000	11.250091000
6	135.308449000	5.527331000	9.527572000
6	135.384174000	6.946526000	10.111618000
6	136.499438000	7.774848000	9.459182000
1	133.503839000	3.452968000	8.625814000
1	135.860539000	2.541935000	9.247378000
1	131.589746000	4.593127000	11.916168000

1	131.367977000	4.862938000	10.181843000
1	135.398631000	0.388685000	10.334257000
1	133.242849000	-0.019595000	11.522770000
1	131.672418000	1.883465000	11.929622000
1	136.235347000	7.890126000	8.398007000
1	136.526260000	8.766216000	9.918005000
1	134.085533000	8.645395000	10.002032000
1	135.075665000	4.510096000	7.041406000
1	137.730675000	6.353453000	9.126098000
8	128.056613000	7.036513000	3.962340000
1	127.313663000	7.501248000	4.414699000
1	128.235236000	7.576347000	3.178302000
1	136.241043000	1.914882000	13.188820000
1	139.807278000	4.957247000	11.477212000
1	135.377759000	5.355164000	17.651598000
1	128.944174000	7.398786000	17.653349000
1	129.357643000	13.580610000	11.737740000
1	124.309401000	10.056412000	6.211912000
1	133.197499000	16.926551000	4.180546000
1	137.717190000	15.193729000	4.517837000
1	141.631564000	13.160014000	10.100020000
1	139.602960000	4.307360000	5.299625000
1	134.607151000	9.779317000	5.411269000
1	129.188517000	4.903515000	4.229020000
1	134.163395000	-0.605602000	4.156978000
1	139.011011000	6.938565000	5.596764000
1	131.971408000	9.232710000	5.055932000
1	129.756677000	2.234863000	4.088610000
1	136.804188000	-0.042391000	4.414811000
1	138.567373000	16.413588000	10.134805000
1	131.449580000	3.374809000	15.986368000

^{2}TS3$_{B}$:

6	138.617314000	3.935489000	13.640907000
6	139.855631000	3.707973000	12.774008000
8	140.696277000	2.853578000	13.059209000
6	138.894429000	3.747531000	15.142379000
6	139.851767000	4.822020000	15.678067000
6	137.581855000	3.727709000	15.937742000
1	138.187862000	4.928010000	13.449851000
1	139.388392000	2.774251000	15.250187000
1	140.059918000	4.666973000	16.743748000
1	139.421134000	5.826871000	15.566132000
1	140.807407000	4.797741000	15.144417000
1	137.771207000	3.561640000	17.005528000
1	137.044794000	4.680923000	15.839364000
1	136.912271000	2.932522000	15.587532000
7	139.941167000	4.497716000	11.666608000
6	141.002393000	4.342645000	10.683117000
6	140.495552000	4.099533000	9.253553000
6	139.612294000	2.882336000	9.033532000
6	139.653889000	1.749173000	9.855382000
6	138.732025000	2.863952000	7.939825000
6	138.845886000	0.640154000	9.591965000
6	137.924214000	1.764436000	7.659123000
6	137.979371000	0.645706000	8.496234000
8	137.151402000	-0.411308000	8.203045000
1	139.169441000	5.122126000	11.451744000
1	141.626981000	3.518719000	11.034743000
1	141.385080000	4.034964000	8.608003000
1	139.956924000	4.994367000	8.910833000
1	140.299793000	1.723222000	10.728365000
1	138.676440000	3.728680000	7.282286000
1	138.886809000	-0.226084000	10.251232000
1	137.236773000	1.779208000	6.818455000
1	137.288113000	-1.103801000	8.868200000
6	132.117237000	6.475113000	19.914598000
6	131.218961000	6.472361000	18.687314000
8	131.447310000	7.177031000	17.702774000
6	133.589894000	6.256810000	19.536822000
1	132.005164000	7.457391000	20.391905000
1	133.904556000	6.996704000	18.796474000
1	134.226491000	6.354238000	20.421119000
1	133.748537000	5.260222000	19.111384000
7	130.108189000	5.670189000	18.738702000
6	129.323946000	5.403055000	17.536991000
6	128.635216000	6.626823000	16.926279000
8	128.337322000	6.635862000	15.733927000
1	130.033845000	5.012921000	19.503300000
1	129.947997000	4.984674000	16.740800000
7	128.375587000	7.667228000	17.757668000
6	127.809941000	8.904454000	17.246659000
6	128.641402000	9.601430000	16.157735000
8	128.086718000	10.402851000	15.401683000
1	128.691123000	7.610097000	18.715467000
1	126.827419000	8.723262000	16.802490000
7	129.956343000	9.291792000	16.077018000
6	130.780996000	9.858225000	15.014218000
6	130.271334000	9.502825000	13.609337000
8	130.579315000	10.234994000	12.655928000
6	132.253250000	9.440398000	15.154900000
6	132.960775000	9.971468000	16.411906000
16	132.928067000	11.789042000	16.661385000
6	133.986998000	12.350117000	15.281289000
1	130.356618000	8.574219000	16.677403000
1	130.718201000	10.949467000	15.069804000
1	132.324099000	8.344298000	15.163407000
1	132.771360000	9.780839000	14.252492000
1	134.004642000	9.636856000	16.402241000
1	132.506151000	9.551906000	17.313965000
1	134.082076000	13.434858000	15.375933000
1	133.545349000	12.121889000	14.307469000
1	134.986748000	11.909379000	15.348253000
7	129.492576000	8.412062000	13.468698000
6	128.929224000	8.061468000	12.168934000
6	128.045640000	9.181307000	11.603004000
8	127.874878000	9.263300000	10.372201000
6	128.160988000	6.735767000	12.251086000
1	129.231229000	7.852438000	14.277032000
1	129.748865000	7.943918000	11.455614000
1	127.806119000	6.456147000	11.255679000
1	127.310002000	6.804378000	12.936510000
1	128.832683000	5.949403000	12.608703000
7	127.460842000	10.053755000	12.444850000
6	126.697603000	11.171030000	11.907466000
6	127.541342000	12.254998000	11.213351000
8	127.020125000	13.007220000	10.385146000
1	127.658129000	10.024310000	13.444221000
1	126.145827000	11.635957000	12.729873000
1	125.981049000	10.813214000	11.164755000
7	128.835868000	12.359036000	11.596816000
6	129.753182000	13.278322000	10.951487000
6	130.620230000	12.707215000	9.816163000
8	131.202752000	13.484681000	9.064707000
1	129.225543000	11.674711000	12.238144000
1	129.175191000	14.096203000	10.518988000
7	130.703834000	11.348045000	9.721803000
6	131.551648000	10.720173000	8.715120000
6	130.795160000	9.905543000	7.645609000
8	131.357628000	9.656271000	6.578952000
6	132.592599000	9.770969000	9.341938000
6	133.540355000	10.407839000	10.313672000
6	133.903357000	11.723194000	10.474313000
7	134.237964000	9.621830000	11.218332000
6	135.007594000	10.449336000	11.902849000
7	134.834716000	11.732192000	11.493880000
1	130.446096000	10.802956000	10.539533000
1	132.048573000	11.530032000	8.177541000
1	133.150626000	9.317635000	8.513090000
1	132.071004000	8.950011000	9.847909000
1	133.579555000	12.625938000	9.978082000
1	135.697892000	10.173777000	12.688146000
1	135.342368000	12.539071000	11.838960000
7	129.568990000	9.427971000	7.981784000
6	128.883259000	8.452473000	7.139671000
6	127.528096000	8.916938000	6.583572000
8	127.127878000	8.467098000	5.500314000
6	128.729230000	7.072949000	7.826817000
8	127.644234000	6.984743000	8.732771000
1	129.199525000	9.627551000	8.904875000

1	129.527517000	8.309255000	6.270585000
1	129.690110000	6.838593000	8.313261000
1	128.545919000	6.328035000	7.049084000
1	127.712618000	7.727140000	9.362971000
7	126.813215000	9.798238000	7.313009000
6	125.489630000	10.256543000	6.914922000
6	125.197007000	11.614499000	7.543237000
8	125.182664000	11.481851000	8.957701000
1	127.077928000	10.002759000	8.269739000
1	124.723025000	9.534969000	7.229852000
1	125.958005000	12.338605000	7.220603000
1	124.219461000	11.969583000	7.181511000
1	125.701875000	12.204358000	9.365168000
7	132.799851000	16.774227000	5.243973000
6	133.402975000	16.889422000	6.590934000
6	134.688349000	16.054743000	6.741336000
8	135.398229000	16.182279000	7.745631000
6	132.287201000	16.431011000	7.575471000
6	131.231273000	15.770975000	6.672867000
6	131.345233000	16.579165000	5.378953000
1	133.705044000	17.918562000	6.828279000
1	131.861409000	17.308718000	8.075588000
1	132.660544000	15.758853000	8.350383000
1	131.485234000	14.722071000	6.492560000
1	130.228145000	15.797251000	7.107105000
1	130.947339000	16.054445000	4.502782000
1	130.798187000	17.533306000	5.476835000
7	134.942328000	15.210320000	5.722542000
6	136.135586000	14.377603000	5.642082000
6	135.985053000	13.160025000	4.710811000
6	135.094026000	12.033956000	5.279541000
6	137.389327000	12.610237000	4.403659000
6	133.598614000	12.345829000	5.423119000
1	134.282556000	15.266444000	4.951755000
1	136.397757000	14.044494000	6.650276000
1	135.538858000	13.503844000	3.762281000
1	135.504899000	11.729138000	6.253110000
1	135.199999000	11.163342000	4.617019000
1	138.015505000	13.364477000	3.911475000
1	137.332083000	11.738485000	3.741810000
1	137.891855000	12.307559000	5.330255000
1	133.038084000	11.447295000	5.703709000
1	133.178630000	12.708901000	4.475392000
1	133.411905000	13.111466000	6.182299000
16	134.882462000	4.304178000	1.943531000
1	134.096588000	3.302680000	1.486667000
6	140.256984000	12.068589000	8.905152000
6	139.065793000	13.003969000	8.718598000
8	138.136136000	12.714806000	7.965682000
6	139.813191000	10.739934000	9.545769000
6	140.930665000	9.689711000	9.704026000
6	140.452735000	8.533292000	10.596399000
6	141.428821000	9.158950000	8.350944000
1	140.672977000	11.884149000	7.908802000
1	139.392181000	10.955827000	10.539141000
1	138.993774000	10.324612000	8.945543000
1	141.779369000	10.174166000	10.212315000
1	141.249047000	7.794185000	10.744717000
1	140.147867000	8.894162000	11.586573000
1	139.594217000	8.011497000	10.154137000
1	142.224283000	8.417649000	8.491186000
1	141.831845000	9.954831000	7.714957000
1	140.613117000	8.672639000	7.800243000
7	139.083150000	14.153412000	9.452800000
6	137.978633000	15.109689000	9.436865000
6	136.737621000	14.653984000	10.236162000
6	136.941112000	14.601407000	11.734010000
6	137.589033000	13.515770000	12.352238000
6	136.504777000	15.655760000	12.553463000
6	137.798885000	13.490006000	13.734842000
6	136.712373000	15.634251000	13.933453000
6	137.361895000	14.550626000	14.530819000
1	139.838348000	14.296286000	10.106912000
1	137.659384000	15.272749000	8.406211000
1	135.930898000	15.346794000	9.978018000
1	136.450081000	13.671948000	9.843441000
1	137.936013000	12.686385000	11.740297000
1	135.997309000	16.502582000	12.097182000
1	138.309559000	12.643379000	14.187606000
1	136.365936000	16.464478000	14.543666000
1	137.526265000	14.534065000	15.604860000
7	136.955070000	3.979002000	4.171139000
7	134.714036000	5.591147000	4.764252000
7	135.201675000	1.738702000	3.654629000
6	137.651960000	1.717273000	3.522748000
6	137.040951000	6.347301000	4.784817000
6	132.262538000	5.614397000	4.900074000
6	132.868365000	1.007001000	3.567167000
6	137.906105000	3.044238000	3.852774000
6	135.677663000	6.550087000	4.955420000
6	132.010779000	4.304434000	4.502242000
6	134.237805000	0.787374000	3.452009000
8	135.086558000	3.147417000	6.024826000
6	139.223718000	3.632692000	3.906180000
6	135.071192000	7.799836000	5.353682000
6	130.682251000	3.746586000	4.359805000
6	134.842741000	-0.472623000	3.086626000
6	139.055362000	4.938607000	4.254912000
6	133.726639000	7.589145000	5.396931000
6	130.851065000	2.447109000	3.993049000
6	136.188262000	-0.268324000	3.073748000
6	137.635136000	5.141965000	4.418369000
6	133.518849000	6.207986000	5.017262000
6	132.276205000	2.217718000	3.908540000
6	136.401232000	1.116055000	3.429733000
7	132.963206000	3.363214000	4.211947000
26	134.954768000	3.660057000	4.209585000
1	138.513814000	1.092008000	3.307454000
1	137.697019000	7.196224000	4.954494000
1	131.392851000	6.234567000	5.100467000
1	132.208975000	0.168701000	3.360792000
6	132.621875000	6.186222000	10.558084000
7	133.856173000	6.738821000	10.734815000
8	131.556516000	6.787651000	10.635453000
16	135.158527000	4.643028000	12.068854000
6	132.708098000	4.682654000	10.274576000
7	133.735455000	4.390341000	9.302517000
8	135.874437000	4.859851000	8.604727000
16	133.147543000	3.955049000	11.991291000
6	131.433585000	4.027078000	9.710650000
8	137.470367000	5.959789000	10.541565000
6	131.985964000	2.932408000	8.841360000
6	133.331461000	3.362376000	8.316093000
6	134.286210000	2.232181000	8.055426000
6	133.753757000	0.974207000	7.806242000
6	132.390388000	0.718108000	7.974749000
6	131.524408000	1.683369000	8.591175000
6	134.935113000	5.008815000	9.389970000
6	135.021674000	5.916869000	10.626840000
6	136.307506000	6.759883000	10.600657000
1	133.196014000	3.874275000	7.345214000
1	135.347875000	2.381812000	8.171966000
1	130.756548000	3.670690000	10.491411000
1	130.893388000	4.794818000	9.138838000
1	134.429923000	0.170762000	7.531082000
1	132.002440000	-0.275848000	7.772064000
1	130.538427000	1.375166000	8.929809000
1	136.233798000	7.433987000	9.735946000
1	136.366494000	7.367917000	11.505923000
1	133.948094000	7.729327000	11.034865000
1	135.605026000	3.836856000	6.478619000
1	137.362731000	5.410726000	9.735793000
8	128.939106000	6.585476000	4.480393000
1	128.223425000	7.205340000	4.755220000
1	129.307542000	6.979703000	3.675767000
1	137.863968000	3.205281000	13.313314000
1	141.634150000	5.242209000	10.674395000
1	131.787181000	5.726599000	20.644872000
1	127.675621000	9.596684000	18.082863000
1	130.429337000	13.703354000	11.701653000

1	125.462063000	10.315087000	5.823632000
1	133.028301000	17.572137000	4.658383000
1	136.977813000	14.993187000	5.289279000
1	141.047502000	12.535505000	9.507969000
1	140.145068000	3.103367000	3.698602000
1	135.615730000	8.712510000	5.562091000
1	129.770732000	4.314161000	4.503606000
1	134.295450000	-1.381487000	2.869891000
1	139.809212000	5.704250000	4.389061000
1	132.937406000	8.286726000	5.651585000
1	130.093801000	1.700895000	3.786385000
1	136.976339000	-0.975076000	2.846197000
1	138.357958000	16.059340000	9.827440000
1	128.556910000	4.663904000	17.787706000

$^{2}Pr_{B}$:

6	139.242797000	4.902213000	13.183042000
6	140.023250000	4.303258000	12.010496000
8	140.965062000	3.528797000	12.191099000
6	140.121889000	5.167189000	14.417220000
6	141.161901000	6.263108000	14.144382000
6	139.256842000	5.513393000	15.636905000
1	138.726275000	5.821684000	12.874144000
1	140.662998000	4.235285000	14.621183000
1	141.798122000	6.428428000	15.022313000
1	140.676153000	7.218338000	13.900880000
1	141.811587000	5.986556000	13.307920000
1	139.877170000	5.666270000	16.528420000
1	138.685158000	6.436562000	15.469473000
1	138.538978000	4.714376000	15.860124000
7	139.575487000	4.663395000	10.777587000
6	140.160946000	4.171730000	9.537430000
6	139.186550000	3.389403000	8.643908000
6	138.670434000	2.055366000	9.158512000
6	139.236063000	1.370685000	10.239425000
6	137.590746000	1.449803000	8.491768000
6	138.741925000	0.125554000	10.640538000
6	137.092963000	0.209161000	8.879435000
6	137.672452000	-0.460667000	9.962881000
8	137.148307000	-1.679276000	10.313572000
1	138.798923000	5.313779000	10.706143000
1	141.024331000	3.564945000	9.818119000
1	139.691439000	3.234930000	7.678846000
1	138.325843000	4.030826000	8.406633000
1	140.060635000	1.801230000	10.799728000
1	137.131589000	1.957840000	7.645613000
1	139.195381000	-0.385719000	11.488961000
1	136.261748000	-0.253093000	8.355282000
1	137.657538000	-2.028031000	11.061617000
6	132.369689000	6.110728000	19.629590000
6	131.395542000	6.181894000	18.461640000
8	131.519123000	6.976523000	17.530034000
6	133.065040000	7.444125000	19.911496000
1	131.851697000	5.740203000	20.523574000
1	132.345834000	8.212064000	20.215476000
1	133.796761000	7.327203000	20.716401000
1	133.584496000	7.803570000	19.019841000
7	130.346763000	5.295675000	18.502706000
6	129.554923000	5.019049000	17.307939000
6	128.723588000	6.196894000	16.792204000
8	128.361275000	6.226948000	15.617861000
1	130.361483000	4.587421000	19.224911000
1	130.190619000	4.714421000	16.470098000
7	128.419844000	7.173054000	17.683480000
6	127.728863000	8.379486000	17.261195000
6	128.441017000	9.187732000	16.164257000
8	127.783773000	9.968965000	15.472278000
1	128.797933000	7.104445000	18.617670000
1	126.741044000	8.137029000	16.860109000
7	129.770565000	8.990229000	16.003359000
6	130.487150000	9.662926000	14.925173000
6	129.938453000	9.318458000	13.533448000
8	130.152619000	10.106137000	12.597612000
6	131.995862000	9.376621000	14.977050000
6	132.722787000	9.957520000	16.200026000
16	132.499736000	11.752989000	16.506026000
6	133.282044000	12.470631000	15.017576000
1	130.261747000	8.294692000	16.560684000
1	130.331397000	10.741501000	15.027655000
1	132.165296000	8.291517000	14.963879000
1	132.430954000	9.770308000	14.053026000
1	133.793956000	9.742238000	16.112521000
1	132.377068000	9.473074000	17.117924000
1	133.235189000	13.556379000	15.136807000
1	132.749644000	12.196021000	14.102740000
1	134.333229000	12.176997000	14.939156000
7	129.224167000	8.187134000	13.386767000
6	128.622294000	7.843726000	12.103352000
6	127.655446000	8.929977000	11.613919000
8	127.433137000	9.046951000	10.394385000
6	127.929799000	6.476645000	12.180820000
1	129.054741000	7.574226000	14.181022000
1	129.414480000	7.791769000	11.352626000
1	127.548533000	6.201913000	11.193853000
1	127.106444000	6.482040000	12.902390000
1	128.657647000	5.720698000	12.490745000
7	127.054662000	9.732744000	12.512071000
6	126.198259000	10.816388000	12.051722000
6	126.935706000	11.973026000	11.353911000
8	126.323930000	12.716989000	10.582317000
1	127.292843000	9.673439000	13.501272000
1	125.661336000	11.219094000	12.915675000
1	125.467827000	10.437105000	11.333433000
7	128.241039000	12.144513000	11.670832000
6	129.067004000	13.135632000	11.007949000
6	129.959204000	12.636850000	9.857389000
8	130.457200000	13.460057000	9.094177000
1	128.701757000	11.470251000	12.274718000
1	128.412515000	13.899628000	10.586116000
7	130.160481000	11.290042000	9.766228000
6	131.040649000	10.726592000	8.749688000
6	130.332663000	9.865649000	7.681475000
8	130.925779000	9.617120000	6.632948000
6	132.154622000	9.862308000	9.371784000
6	133.113637000	10.595734000	10.262139000
6	133.380067000	11.939182000	10.375350000
7	133.945244000	9.886893000	11.117323000
6	134.698910000	10.786586000	11.723993000
7	134.388958000	12.042242000	11.313565000
1	129.963168000	10.724501000	10.587399000
1	131.472724000	11.568828000	8.204862000
1	132.700796000	9.396455000	8.542404000
1	131.700910000	9.043194000	9.943082000
1	132.942059000	12.802445000	9.896925000
1	135.471973000	10.588073000	12.452620000
1	134.862002000	12.887968000	11.607221000
7	129.107496000	9.368569000	7.994694000
6	128.450997000	8.396731000	7.125334000
6	127.064165000	8.818919000	6.614587000
8	126.684425000	8.430196000	5.500550000
6	128.385447000	6.979498000	7.743444000
8	127.358407000	6.799383000	8.700882000
1	128.711060000	9.576298000	8.904062000
1	129.083624000	8.324019000	6.239167000
1	129.382589000	6.757820000	8.159816000
1	128.190140000	6.268023000	6.937263000
1	127.399166000	7.537832000	9.338257000
7	126.298238000	9.594560000	7.410258000
6	124.949036000	10.000758000	7.039875000
6	124.585753000	11.309934000	7.730682000
8	124.579304000	11.109638000	9.137062000
1	126.548237000	9.741597000	8.381788000
1	124.224207000	9.225005000	7.323255000
1	125.307950000	12.087185000	7.444368000
1	123.590968000	11.629908000	7.382864000
1	125.042249000	11.851551000	9.576139000
7	133.427592000	16.495203000	5.290042000
6	133.958668000	16.539032000	6.672572000
6	135.312434000	15.811563000	6.818707000
8	135.894108000	15.805384000	7.909902000

6	132.864071000	15.881049000	7.551878000
6	132.043406000	15.055389000	6.549735000
6	132.051804000	15.953128000	5.309686000
1	134.149308000	17.566805000	7.007957000
1	132.232096000	16.656971000	7.999710000
1	133.294375000	15.294769000	8.366919000
1	132.550532000	14.109256000	6.328515000
1	131.041442000	14.820326000	6.914426000
1	131.841090000	15.409028000	4.381973000
1	131.293223000	16.746879000	5.412314000
7	135.758049000	15.215445000	5.696105000
6	137.029754000	14.517787000	5.565529000
6	136.917396000	13.115490000	4.926139000
6	136.472782000	12.012842000	5.910248000
6	138.276351000	12.734358000	4.314115000
6	135.115399000	12.209604000	6.595569000
1	135.142938000	15.335420000	4.894754000
1	137.476508000	14.444416000	6.559633000
1	136.176997000	13.173606000	4.111409000
1	137.253815000	11.908588000	6.674608000
1	136.451045000	11.065367000	5.352204000
1	138.586566000	13.452135000	3.544814000
1	138.233814000	11.742638000	3.849094000
1	139.049690000	12.711715000	5.091365000
1	134.847366000	11.319516000	7.178664000
1	134.314069000	12.374926000	5.864902000
1	135.129540000	13.060148000	7.283945000
16	134.948202000	4.657934000	2.161794000
1	134.119005000	3.746020000	1.604478000
6	140.944095000	12.151802000	9.209084000
6	139.831624000	13.156626000	8.931143000
8	139.182682000	13.121695000	7.886954000
6	140.348590000	10.770133000	9.547722000
6	141.381380000	9.641714000	9.740972000
6	140.707574000	8.413604000	10.373186000
6	142.078820000	9.263540000	8.425208000
1	141.548804000	12.089675000	8.299123000
1	139.754205000	10.863601000	10.468682000
1	139.646453000	10.490168000	8.751742000
1	142.150536000	10.001668000	10.442353000
1	141.418401000	7.588782000	10.498365000
1	140.299614000	8.651608000	11.363628000
1	139.876618000	8.048863000	9.756145000
1	142.814533000	8.467522000	8.588670000
1	142.606658000	10.112291000	7.976122000
1	141.349513000	8.898619000	7.690309000
7	139.579985000	14.062621000	9.922427000
6	138.442521000	14.974238000	9.875265000
6	137.126325000	14.363104000	10.407508000
6	137.135108000	14.078765000	11.893057000
6	137.668396000	12.883086000	12.407621000
6	136.634992000	15.021258000	12.807718000
6	137.713275000	12.644876000	13.783860000
6	136.674947000	14.786157000	14.184074000
6	137.217180000	13.597345000	14.678187000
1	140.090627000	13.978117000	10.789694000
1	138.275680000	15.260871000	8.836449000
1	136.327125000	15.062907000	10.144642000
1	136.938148000	13.446395000	9.837076000
1	138.046747000	12.132797000	11.717467000
1	136.215267000	15.951712000	12.431410000
1	138.139506000	11.717027000	14.157856000
1	136.284083000	15.532814000	14.870539000
1	137.253700000	13.415330000	15.748866000
7	136.787266000	4.179989000	4.556719000
7	134.537900000	5.820173000	4.986070000
7	135.055794000	2.022082000	3.772716000
6	137.510040000	1.930270000	3.884062000
6	136.870770000	6.530733000	5.246906000
6	132.082562000	5.918513000	4.877614000
6	132.729518000	1.365665000	3.360862000
6	137.749119000	3.235185000	4.293421000
6	135.502050000	6.752451000	5.291814000
6	131.852783000	4.628659000	4.413730000
6	134.096215000	1.115093000	3.391485000
8	134.940254000	3.257191000	6.559632000
6	139.064806000	3.792965000	4.495059000
6	134.888061000	8.002962000	5.667283000
6	130.538679000	4.109402000	4.108435000
6	134.709362000	-0.137698000	3.024300000
6	138.888921000	5.090921000	4.869825000
6	133.540137000	7.828577000	5.575831000
6	130.720104000	2.829418000	3.682194000
6	136.052708000	0.023138000	3.181473000
6	137.465937000	5.323410000	4.903417000
6	133.333621000	6.467439000	5.134881000
6	132.138081000	2.565546000	3.728643000
6	136.261130000	1.375793000	3.638586000
7	132.815726000	3.673802000	4.183613000
26	134.800954000	3.948178000	4.282211000
1	138.375064000	1.293360000	3.724941000
1	137.523768000	7.361106000	5.498506000
1	131.211842000	6.551640000	5.021248000
1	132.078073000	0.565622000	3.022040000
6	132.572890000	6.337356000	10.666533000
7	133.769344000	6.989857000	10.676854000
8	131.488261000	6.839595000	10.934369000
16	135.437986000	4.987960000	11.703013000
6	132.741569000	4.861222000	10.289197000
7	133.607577000	4.722014000	9.129589000
8	135.641985000	5.313563000	8.233038000
16	133.519467000	4.078232000	11.842108000
6	131.453309000	4.115470000	9.915827000
8	137.363374000	6.586586000	9.891938000
6	131.905646000	3.169865000	8.837829000
6	133.080578000	3.787269000	8.097921000
6	134.073821000	2.737865000	7.568419000
6	133.328494000	1.552502000	6.996669000
6	132.125175000	1.182817000	7.458866000
6	131.458706000	1.950275000	8.518692000
6	134.776568000	5.394219000	9.117362000
6	134.968034000	6.277047000	10.358538000
6	136.150307000	7.247803000	10.180765000
1	132.728569000	4.385373000	7.243208000
1	134.694434000	2.382432000	8.409867000
1	130.989178000	3.617032000	10.771145000
1	130.735497000	4.861017000	9.546405000
1	133.852804000	0.988717000	6.232707000
1	131.634970000	0.293512000	7.073519000
1	130.609784000	1.512705000	9.038482000
1	135.873311000	7.947646000	9.380232000
1	136.290517000	7.818048000	11.101662000
1	133.819698000	7.983821000	10.977918000
1	135.401123000	4.032239000	6.948449000
1	137.186689000	6.099105000	9.060139000
8	128.628205000	6.806183000	4.301512000
1	127.863395000	7.331382000	4.633800000
1	129.008665000	7.351180000	3.596808000
1	138.457356000	4.177926000	13.442966000
1	140.539457000	5.026645000	8.960813000
1	133.117646000	5.347059000	19.371285000
1	127.585456000	9.021732000	18.134933000
1	129.718797000	13.620829000	11.743533000
1	124.913111000	10.107603000	5.952737000
1	133.472190000	17.403889000	4.839904000
1	137.702304000	15.134113000	4.950771000
1	141.600461000	12.489741000	10.022277000
1	139.991572000	3.250969000	4.353635000
1	135.433810000	8.894948000	5.950043000
1	129.626187000	4.687488000	4.197117000
1	134.168648000	-1.015085000	2.691997000
1	139.640902000	5.835024000	5.101122000
1	132.745659000	8.537893000	5.776224000
1	129.973375000	2.115321000	3.357298000
1	136.843339000	-0.695274000	3.004513000
1	138.703447000	15.873571000	10.443721000
1	128.875670000	4.191127000	17.532723000

^{2}TS4$_{B}$:

6	134.196790000	0.714763000	13.804106000

6	135.504905000	0.054945000	13.355374000
8	135.880251000	-1.015140000	13.838798000
6	133.878030000	0.464304000	15.288106000
6	134.905871000	1.138984000	16.207768000
6	132.450758000	0.920194000	15.622181000
1	134.220087000	1.793061000	13.594779000
1	133.947755000	-0.618872000	15.443941000
1	134.683291000	0.932249000	17.262035000
1	134.903137000	2.229579000	16.072618000
1	135.915732000	0.770663000	16.002577000
1	132.205700000	0.704578000	16.669809000
1	132.333336000	2.002308000	15.467356000
1	131.707230000	0.413590000	14.994816000
7	136.181783000	0.718717000	12.381596000
6	137.460527000	0.263445000	11.852148000
6	137.454642000	0.003877000	10.339152000
6	136.534659000	-1.089467000	9.821175000
6	136.032045000	-2.119309000	10.626413000
6	136.201002000	-1.107569000	8.456849000
6	135.234035000	-3.132046000	10.087222000
6	135.413305000	-2.114497000	7.904874000
6	134.926386000	-3.138489000	8.724965000
8	134.152344000	-4.107456000	8.141326000
1	135.828550000	1.612117000	12.049501000
1	137.729622000	-0.633057000	12.414092000
1	138.492277000	-0.232135000	10.056277000
1	137.223377000	0.945359000	9.823196000
1	136.236155000	-2.136628000	11.692900000
1	136.567919000	-0.314594000	7.808219000
1	134.850070000	-3.918733000	10.735478000
1	135.165085000	-2.119304000	6.847629000
1	133.900491000	-4.753107000	8.819813000
6	132.856284000	6.007563000	20.625792000
6	132.184944000	5.936220000	19.262677000
8	132.416255000	6.755500000	18.372244000
6	134.379873000	6.150759000	20.498819000
1	132.448516000	6.889101000	21.137885000
1	134.628264000	7.012802000	19.874605000
1	134.830722000	6.291055000	21.485651000
1	134.826457000	5.260580000	20.043409000
7	131.267365000	4.933603000	19.079316000
6	130.758217000	4.621110000	17.747282000
6	129.953715000	5.741873000	17.083050000
8	129.867187000	5.797839000	15.857814000
1	131.214886000	4.207752000	19.781214000
1	131.572906000	4.385353000	17.055036000
7	129.351646000	6.636917000	17.905943000
6	128.641087000	7.785763000	17.370306000
6	129.484517000	8.730143000	16.498292000
8	128.911258000	9.485017000	15.708954000
1	129.505707000	6.554853000	18.900891000
1	127.807806000	7.462371000	16.740534000
7	130.829695000	8.677085000	16.635462000
6	131.690436000	9.482575000	15.775069000
6	131.526239000	9.155329000	14.281585000
8	131.875870000	9.988879000	13.437207000
6	133.167569000	9.367940000	16.181280000
6	133.504237000	9.933132000	17.569227000
16	133.059623000	11.689820000	17.858404000
6	134.206615000	12.528883000	16.708097000
1	131.259779000	7.990605000	17.251872000
1	131.382862000	10.529442000	15.863275000
1	133.479856000	8.315297000	16.156784000
1	133.748727000	9.885263000	15.411398000
1	134.576858000	9.807155000	17.756508000
1	132.980505000	9.373595000	18.349832000
1	134.080542000	13.602560000	16.871041000
1	133.976159000	12.304506000	15.662701000
1	135.245339000	12.260085000	16.925777000
7	130.973992000	7.966686000	13.946087000
6	130.669614000	7.652199000	12.554485000
6	129.785692000	8.720037000	11.900244000
8	129.846239000	8.893487000	10.667643000
6	129.994896000	6.278345000	12.450823000
1	130.673477000	7.312352000	14.664620000
1	131.590942000	7.642907000	11.965474000
1	129.770999000	6.058240000	11.403517000
1	129.064422000	6.246560000	13.026947000
1	130.662793000	5.503103000	12.837895000
7	128.947079000	9.431200000	12.673801000
6	128.108561000	10.465812000	12.091624000
6	128.854842000	11.674460000	11.508929000
8	128.257730000	12.440537000	10.746619000
1	128.967070000	9.331001000	13.688506000
1	127.421547000	10.823121000	12.863830000
1	127.512288000	10.058926000	11.269641000
7	130.146441000	11.855412000	11.873283000
6	130.958563000	12.893648000	11.268819000
6	131.798156000	12.482741000	10.047245000
8	132.454467000	13.342668000	9.462606000
1	130.601036000	11.201981000	12.509407000
1	130.305490000	13.706971000	10.945939000
7	131.774413000	11.166701000	9.700040000
6	132.595626000	10.630106000	8.622268000
6	131.801012000	9.977035000	7.475463000
8	132.353548000	9.823879000	6.382585000
6	133.627263000	9.595975000	9.132438000
6	134.642663000	10.166306000	10.079329000
6	135.002447000	11.474903000	10.293405000
7	135.402199000	9.343877000	10.896343000
6	136.197659000	10.145346000	11.586496000
7	135.989609000	11.442900000	11.254982000
1	131.305088000	10.514962000	10.320497000
1	133.110910000	11.482186000	8.174477000
1	134.128496000	9.167950000	8.255586000
1	133.103591000	8.763731000	9.619608000
1	134.629143000	12.400821000	9.885674000
1	136.921797000	9.834073000	12.326925000
1	136.459998000	12.257294000	11.634014000
7	130.553215000	9.536571000	7.767110000
6	129.729928000	8.746791000	6.849062000
6	128.485469000	9.572784000	6.455544000
8	128.516887000	10.327619000	5.475351000
6	129.451617000	7.349444000	7.432002000
8	128.709015000	7.355829000	8.646892000
1	130.166785000	9.750050000	8.678677000
1	130.310825000	8.622470000	5.933740000
1	130.415521000	6.838091000	7.564814000
1	128.860198000	6.773943000	6.712808000
1	129.258242000	7.749456000	9.354769000
7	127.414121000	9.447485000	7.267001000
6	126.221192000	10.271496000	7.126359000
6	126.250238000	11.497409000	8.036808000
8	126.239707000	11.081581000	9.392719000
1	127.499084000	8.815076000	8.059135000
1	125.347170000	9.657489000	7.370005000
1	127.146594000	12.090330000	7.808040000
1	125.370081000	12.122419000	7.809795000
1	126.809239000	11.697364000	9.898005000
7	131.672907000	14.449928000	3.609132000
6	131.951868000	15.172864000	4.873191000
6	133.463012000	15.300473000	5.164504000
8	133.849897000	15.877738000	6.185860000
6	131.211769000	14.382764000	5.981412000
6	131.022892000	12.992555000	5.356122000
6	130.761525000	13.314856000	3.882991000
1	131.588398000	16.208683000	4.839090000
1	130.234220000	14.844273000	6.168584000
1	131.765098000	14.379427000	6.923799000
1	131.940820000	12.401757000	5.450166000
1	130.204359000	12.417794000	5.797695000
1	130.975682000	12.463676000	3.229435000
1	129.704650000	13.594674000	3.742394000
7	134.264179000	14.758730000	4.223378000
6	135.711589000	14.899901000	4.178245000
6	136.492498000	13.569984000	4.073384000
6	136.649777000	12.825787000	5.415849000
6	137.884904000	13.861434000	3.487140000
6	135.359486000	12.365454000	6.104882000
1	133.753826000	14.327227000	3.455570000

1	136.022680000	15.451440000	5.068912000
1	135.952821000	12.913002000	3.372282000
1	137.216224000	13.477320000	6.095525000
1	137.283214000	11.945183000	5.234609000
1	137.813929000	14.314736000	2.490778000
1	138.475089000	12.942143000	3.395165000
1	138.433844000	14.554441000	4.136546000
1	135.600103000	11.764149000	6.992156000
1	134.741066000	11.745299000	5.444403000
1	134.751376000	13.211353000	6.437783000
16	136.108179000	4.469794000	2.856518000
1	137.310428000	4.852153000	3.343593000
6	139.761900000	17.165482000	7.824773000
6	138.329539000	16.706427000	7.545195000
8	138.038889000	16.089738000	6.521260000
6	140.389381000	17.832477000	6.591131000
6	141.903467000	18.094662000	6.696738000
6	142.446139000	18.607759000	5.353955000
6	142.259856000	19.070071000	7.829712000
1	139.796617000	17.829872000	8.696587000
1	140.189261000	17.183309000	5.731484000
1	139.871125000	18.781728000	6.389714000
1	142.393971000	17.132222000	6.912418000
1	143.531641000	18.757968000	5.394438000
1	142.232803000	17.902471000	4.542280000
1	141.987151000	19.569199000	5.088733000
1	143.340496000	19.253043000	7.864363000
1	141.959996000	18.689982000	8.812967000
1	141.764405000	20.038834000	7.680218000
7	137.411610000	17.023320000	8.502045000
6	136.007949000	16.628416000	8.418024000
6	135.723845000	15.196392000	8.922380000
6	135.969370000	15.003565000	10.401378000
6	137.250530000	14.708069000	10.898276000
6	134.918015000	15.133673000	11.324349000
6	137.477433000	14.558700000	12.271204000
6	135.139668000	14.986150000	12.695188000
6	136.420477000	14.700299000	13.176167000
1	137.736430000	17.471595000	9.346199000
1	135.686424000	16.683605000	7.377188000
1	134.680778000	14.971309000	8.676752000
1	136.346742000	14.509679000	8.338716000
1	138.074707000	14.586335000	10.199728000
1	133.914178000	15.321054000	10.951784000
1	138.479330000	14.338321000	12.632750000
1	134.309611000	15.090233000	13.389760000
1	136.594874000	14.589205000	14.243333000
7	136.719315000	3.195667000	5.605737000
7	135.705280000	5.839387000	5.572027000
7	134.402691000	2.300960000	4.144693000
6	136.313132000	0.904674000	4.817658000
6	137.822024000	5.148672000	6.600376000
6	133.838625000	7.248139000	4.818809000
6	132.269067000	2.988226000	3.150747000
6	137.073492000	1.875960000	5.460891000
6	136.862031000	6.096173000	6.264437000
6	133.064897000	6.265374000	4.216470000
6	133.240957000	2.042942000	3.462755000
8	134.259883000	3.787144000	6.642515000
6	138.354785000	1.634772000	6.079613000
6	136.950611000	7.503310000	6.582362000
6	131.799725000	6.526644000	3.562903000
6	133.171631000	0.646487000	3.104006000
6	138.776024000	2.826037000	6.591674000
6	135.832715000	8.091012000	6.073841000
6	131.353200000	5.326398000	3.101818000
6	134.313394000	0.065853000	3.567742000
6	137.755057000	3.796091000	6.283640000
6	135.061408000	7.041674000	5.446325000
6	132.342041000	4.337734000	3.467460000
6	135.079001000	1.106420000	4.212399000
7	133.373351000	4.929001000	4.157710000
26	135.064239000	4.080520000	4.822556000
1	136.724434000	-0.099901000	4.779837000
1	138.705592000	5.495335000	7.128913000
1	133.448531000	8.259210000	4.814154000
1	131.398688000	2.648482000	2.597459000
6	134.074420000	5.793578000	10.860984000
7	135.253402000	6.459484000	10.997830000
8	133.022721000	6.327272000	10.516890000
16	136.576802000	6.195298000	13.495551000
6	134.134322000	4.295386000	11.131347000
7	135.227192000	3.655391000	11.388128000
8	137.499501000	3.744846000	11.378712000
16	134.725597000	6.049990000	14.171953000
6	132.841341000	3.533626000	10.998483000
8	138.845754000	6.165014000	11.699210000
6	132.782428000	2.893398000	9.612458000
6	132.505364000	3.662154000	8.492568000
6	132.588776000	3.095017000	7.173713000
6	132.733030000	1.669533000	7.067918000
6	133.004803000	0.914555000	8.195386000
6	133.060873000	1.518564000	9.462641000
6	136.441156000	4.357538000	11.407297000
6	136.424184000	5.872618000	11.512645000
6	137.709200000	6.505567000	10.943649000
1	132.299775000	4.722994000	8.593626000
1	132.111004000	3.625264000	6.357494000
1	132.814629000	2.757594000	11.768229000
1	132.003071000	4.217801000	11.140287000
1	132.678281000	1.207181000	6.088940000
1	133.178201000	-0.153613000	8.109142000
1	133.308334000	0.917936000	10.333298000
1	137.789053000	6.189484000	9.891819000
1	137.609660000	7.593638000	10.959949000
1	135.237284000	7.505937000	10.916162000
1	134.883959000	3.183258000	7.086742000
1	138.917298000	5.193135000	11.644845000
8	130.848277000	9.778508000	3.854320000
1	129.986787000	10.034809000	4.243062000
1	131.468270000	9.917170000	4.593457000
1	133.393218000	0.287887000	13.185707000
1	138.218821000	1.027832000	12.060884000
1	132.596356000	5.135748000	21.238050000
1	128.225049000	8.356993000	18.205158000
1	131.654677000	13.291443000	12.013677000
1	126.150756000	10.584762000	6.081741000
1	131.295511000	15.070960000	2.900586000
1	135.966447000	15.522306000	3.307509000
1	140.343061000	16.268318000	8.083314000
1	138.857133000	0.675806000	6.103617000
1	137.773058000	7.965448000	7.114280000
1	131.348647000	7.511118000	3.478845000
1	132.354378000	0.188586000	2.561040000
1	139.694014000	3.043393000	7.123225000
1	135.538432000	9.132457000	6.099033000
1	130.445897000	5.112342000	2.550577000
1	134.625649000	-0.967639000	3.484227000
1	135.423236000	17.354772000	8.992594000
1	130.118738000	3.736568000	17.826288000

^{2}IM4$_B$:

6	136.763277000	2.908467000	14.133988000
6	137.985671000	2.495312000	13.306460000
8	138.849763000	1.747123000	13.766263000
6	137.010453000	2.848420000	15.650058000
6	138.027876000	3.905601000	16.101809000
6	135.688160000	2.989613000	16.417773000
1	136.426368000	3.912725000	13.840852000
1	137.435572000	1.860200000	15.861531000
1	138.219108000	3.830092000	17.179448000
1	137.661430000	4.920739000	15.896764000
1	138.982057000	3.770956000	15.584088000
1	135.855565000	2.913932000	17.500148000
1	135.221230000	3.964387000	16.216195000
1	134.971856000	2.207835000	16.133422000
7	138.009775000	2.980772000	12.036341000
6	139.081736000	2.687404000	11.094815000
6	138.629592000	1.893460000	9.858320000
6	138.029489000	0.515633000	10.091737000

6	138.314754000	-0.265504000	11.218572000
6	137.186183000	-0.037471000	9.113013000
6	137.779213000	-1.547992000	11.361054000
6	136.650592000	-1.316525000	9.240011000
6	136.948993000	-2.080836000	10.372646000
8	136.397424000	-3.332292000	10.460022000
1	137.297064000	3.642206000	11.738801000
1	139.852996000	2.153125000	11.653074000
1	139.511276000	1.802742000	9.204171000
1	137.909296000	2.502944000	9.295961000
1	138.935420000	0.125345000	12.018696000
1	136.950615000	0.542906000	8.223337000
1	138.009898000	-2.132181000	12.251036000
1	136.003114000	-1.735971000	8.475822000
1	136.703426000	-3.748748000	11.280721000
6	134.159775000	5.895138000	18.988147000
6	133.147925000	5.747538000	17.861526000
8	132.944773000	6.620608000	17.017657000
6	134.447263000	7.353199000	19.349727000
1	133.820042000	5.329373000	19.865459000
1	133.553774000	7.850817000	19.741623000
1	135.227835000	7.406298000	20.114465000
1	134.784258000	7.907052000	18.470152000
7	132.452135000	4.561971000	17.832277000
6	131.746378000	4.150070000	16.622287000
6	130.562628000	5.039050000	16.234129000
8	130.189287000	5.094770000	15.063313000
1	132.763393000	3.827687000	18.455259000
1	132.413088000	4.140090000	15.754138000
7	129.961309000	5.730171000	17.234157000
6	128.881392000	6.662524000	16.957081000
6	129.238972000	7.808251000	15.996061000
8	128.334615000	8.396864000	15.399939000
1	130.359234000	5.673493000	18.161179000
1	128.033071000	6.143942000	16.502201000
7	130.551028000	8.108620000	15.845388000
6	130.966594000	9.120217000	14.881005000
6	130.583948000	8.768320000	13.437674000
8	130.546886000	9.678236000	12.593546000
6	132.471352000	9.412684000	14.971455000
6	132.917506000	10.086296000	16.276420000
16	132.090902000	11.672016000	16.691032000
6	132.644903000	12.727317000	15.304643000
1	131.267302000	7.547891000	16.301973000
1	130.417451000	10.043287000	15.094663000
1	133.037930000	8.479361000	14.858167000
1	132.731868000	10.037825000	14.111997000
1	134.000732000	10.248986000	16.242899000
1	132.721579000	9.435184000	17.133721000
1	132.264825000	13.732007000	15.508496000
1	132.243747000	12.388546000	14.345509000
1	133.737641000	12.769329000	15.254021000
7	130.256294000	7.494723000	13.147890000
6	129.771769000	7.141354000	11.819637000
6	128.575317000	8.005424000	11.402100000
8	128.389162000	8.261911000	10.203268000
6	129.400617000	5.654534000	11.752355000
1	130.296445000	6.770019000	13.861200000
1	130.556178000	7.354664000	11.088899000
1	129.031236000	5.415123000	10.750935000
1	128.625761000	5.404626000	12.484143000
1	130.278662000	5.034406000	11.955464000
7	127.721836000	8.443956000	12.351513000
6	126.587471000	9.267516000	11.961375000
6	126.937317000	10.672389000	11.443969000
8	126.121299000	11.286169000	10.752745000
1	127.927148000	8.306959000	13.340055000
1	125.922535000	9.370009000	12.823843000
1	126.035880000	8.780404000	11.153107000
7	128.131380000	11.188613000	11.822452000
6	128.622227000	12.447480000	11.295985000
6	129.676112000	12.366654000	10.178527000
8	129.985551000	13.392813000	9.579714000
1	128.774320000	10.615821000	12.361303000
1	127.772709000	12.999363000	10.889999000

7	130.221901000	11.140126000	9.923605000
6	131.288796000	10.981090000	8.945564000
6	130.897792000	10.175071000	7.689846000
8	131.652041000	10.219535000	6.709217000
6	132.559615000	10.371175000	9.567481000
6	133.281534000	11.290987000	10.508935000
6	133.143378000	12.644169000	10.707818000
7	134.309585000	10.814136000	11.308306000
6	134.781270000	11.860637000	11.965828000
7	134.100926000	12.986827000	11.642149000
1	130.117269000	10.416225000	10.627724000
1	131.511363000	11.980347000	8.564150000
1	133.227828000	10.101535000	8.741370000
1	132.315197000	9.438520000	10.090783000
1	132.454668000	13.365603000	10.293484000
1	135.603866000	11.856087000	12.666461000
1	134.331261000	13.925791000	11.945852000
7	129.763129000	9.445421000	7.746953000
6	129.240767000	8.676521000	6.610301000
6	127.916361000	9.330814000	6.151949000
8	127.928877000	10.271234000	5.348103000
6	129.190027000	7.164462000	6.897856000
8	128.255763000	6.775520000	7.901292000
1	129.197830000	9.505167000	8.587873000
1	129.947040000	8.828988000	5.793356000
1	130.202621000	6.816592000	7.142817000
1	128.882451000	6.651234000	5.981205000
1	128.502493000	7.196289000	8.749970000
7	126.791217000	8.847985000	6.718629000
6	125.479841000	9.439429000	6.491904000
6	125.063296000	10.394100000	7.608448000
8	124.903013000	9.669248000	8.815943000
1	126.892068000	8.073404000	7.371520000
1	124.744401000	8.630739000	6.413633000
1	125.826276000	11.178690000	7.710741000
1	124.120252000	10.884980000	7.310906000
1	125.138205000	10.271814000	9.548931000
7	132.284440000	14.794897000	4.239204000
6	132.898413000	15.578563000	5.334379000
6	134.390361000	15.241736000	5.546882000
8	135.042678000	15.835102000	6.414422000
6	132.054204000	15.256265000	6.594213000
6	131.332756000	13.951302000	6.223829000
6	131.050591000	14.139135000	4.730988000
1	132.880499000	16.658455000	5.133857000
1	131.319072000	16.052319000	6.761109000
1	132.675557000	15.188546000	7.490705000
1	131.992847000	13.087262000	6.365634000
1	130.428877000	13.786812000	6.816334000
1	130.873821000	13.192388000	4.210777000
1	130.162979000	14.779924000	4.595136000
7	134.872524000	14.287682000	4.726239000
6	136.267993000	13.881545000	4.643702000
6	136.492616000	12.353910000	4.712555000
6	136.478247000	11.785616000	6.147323000
6	137.837500000	12.015512000	4.046774000
6	135.186191000	11.976143000	6.951020000
1	134.177794000	13.916819000	4.080444000
1	136.811560000	14.382554000	5.448643000
1	135.690395000	11.866403000	4.135507000
1	137.315675000	12.238005000	6.694760000
1	136.696721000	10.709846000	6.078235000
1	137.857148000	12.333702000	2.997391000
1	138.028978000	10.936471000	4.072190000
1	138.659368000	12.519170000	4.570443000
1	135.268676000	11.476836000	7.926400000
1	134.312619000	11.555373000	6.437885000
1	134.982471000	13.033042000	7.145585000
16	135.398577000	5.129913000	2.439082000
1	135.909432000	3.914013000	2.135901000
6	140.820683000	14.041223000	8.445167000
6	139.491357000	14.613983000	7.959226000
8	138.973608000	14.232726000	6.911107000
6	140.761014000	12.504261000	8.495466000
6	142.039336000	11.812070000	9.005144000

6	141.798898000	10.298854000	9.130575000
6	143.260024000	12.093661000	8.115544000
1	141.591822000	14.364521000	7.735886000
1	139.923456000	12.205713000	9.142375000
1	140.513874000	12.140212000	7.490744000
1	142.256461000	12.202840000	10.012118000
1	142.674998000	9.793321000	9.554479000
1	140.936053000	10.078956000	9.770670000
1	141.605558000	9.855511000	8.144718000
1	144.144201000	11.562398000	8.487384000
1	143.510062000	13.159866000	8.078386000
1	143.078177000	11.756993000	7.086533000
7	138.922070000	15.560860000	8.762920000
6	137.591191000	16.104806000	8.516645000
6	136.456290000	15.299189000	9.187066000
6	136.459389000	15.342103000	10.697838000
6	137.318807000	14.526046000	11.453413000
6	135.591653000	16.199799000	11.394744000
6	137.318070000	14.569167000	12.850143000
6	135.578731000	16.240394000	12.792878000
6	136.444077000	15.423999000	13.527561000
1	139.365231000	15.766103000	9.646822000
1	137.412825000	16.094344000	7.440870000
1	135.515353000	15.688749000	8.787776000
1	136.536794000	14.263826000	8.837181000
1	137.982777000	13.836246000	10.938652000
1	134.915751000	16.839709000	10.831659000
1	137.997402000	13.930487000	13.409791000
1	134.896951000	16.913692000	13.306558000
1	136.440011000	15.457705000	14.613753000
7	135.404686000	3.171565000	4.894137000
7	134.739485000	5.872561000	5.450928000
7	133.198576000	2.994275000	3.166408000
6	134.677982000	1.125760000	3.752265000
6	136.709631000	4.686045000	6.320731000
6	133.180348000	7.710490000	4.959809000
6	131.425360000	4.276658000	2.051160000
6	135.537292000	1.836682000	4.577387000
6	135.891738000	5.799271000	6.210721000
6	132.424257000	7.042027000	4.010495000
6	132.138004000	3.112373000	2.295980000
8	132.113755000	4.787849000	7.031184000
6	136.709377000	1.281892000	5.203342000
6	136.124458000	7.046003000	6.889342000
6	131.325059000	7.641458000	3.287621000
6	131.847357000	1.842345000	1.680621000
6	137.298256000	2.295501000	5.898703000
6	135.114725000	7.889320000	6.529667000
6	130.846105000	6.674623000	2.459482000
6	132.744894000	0.947783000	2.177995000
6	136.475056000	3.462780000	5.711252000
6	134.272529000	7.163043000	5.617707000
6	131.644966000	5.492236000	2.678245000
6	133.591863000	1.673604000	3.088935000
7	132.612614000	5.731650000	3.626363000
26	134.099272000	4.495329000	4.132590000
1	134.889925000	0.073310000	3.590078000
1	137.588046000	4.772359000	6.953043000
1	132.904998000	8.731594000	5.200219000
1	130.614079000	4.224087000	1.331579000
6	134.254532000	7.045421000	11.236844000
7	135.170198000	8.055850000	11.197937000
8	133.041291000	7.224028000	11.198920000
16	137.177630000	7.960807000	13.227279000
6	134.840818000	5.642534000	11.313078000
7	136.095952000	5.386668000	11.139100000
8	138.075499000	6.239737000	10.393950000
16	135.697585000	7.202610000	14.294355000
6	133.869370000	4.516679000	11.538012000
8	138.744276000	8.889830000	10.990777000
6	133.489598000	3.861321000	10.206417000
6	132.527978000	4.414625000	9.401837000
6	132.110046000	3.808011000	8.089242000
6	132.903382000	2.583430000	7.732149000
6	133.872123000	2.077243000	8.554100000
6	134.177912000	2.684627000	9.797598000
6	136.976634000	6.449177000	10.888010000
6	136.557860000	7.848832000	11.307942000
6	137.388368000	8.944915000	10.614320000
1	132.034214000	5.339706000	9.684957000
1	131.041660000	3.537741000	8.149608000
1	134.347874000	3.776144000	12.185524000
1	132.980887000	4.908369000	12.036366000
1	132.674392000	2.118126000	6.777611000
1	134.426638000	1.190741000	8.259165000
1	134.951066000	2.258708000	10.429907000
1	137.249426000	8.832265000	9.528894000
1	137.002133000	9.925754000	10.901850000
1	134.810815000	9.039335000	11.256419000
1	133.037029000	5.039153000	6.844004000
1	139.073230000	8.026824000	10.675211000
8	130.457580000	10.714054000	4.083286000
1	129.533232000	10.665804000	4.409193000
1	130.979584000	10.685342000	4.906071000
1	135.951141000	2.214925000	13.868294000
1	139.513554000	3.635627000	10.752267000
1	135.082456000	5.404494000	18.646500000
1	128.541179000	7.091003000	17.904236000
1	129.053361000	13.048161000	12.104908000
1	125.515426000	9.975667000	5.540337000
1	132.108095000	15.365689000	3.418549000
1	136.680122000	14.254242000	3.694223000
1	141.096221000	14.448185000	9.427817000
1	137.032817000	0.253155000	5.106180000
1	136.967398000	7.233037000	7.543462000
1	130.995591000	8.670767000	3.409627000
1	131.052009000	1.673817000	0.965685000
1	138.203930000	2.269669000	6.491160000
1	134.947942000	8.917061000	6.824699000
1	130.023255000	6.737284000	1.758348000
1	132.843086000	-0.106862000	1.953803000
1	137.576507000	17.145424000	8.859461000
1	131.376313000	3.131313000	16.774292000

Model A structures:

^4Re_A:

16	132.994388000	3.633483000	3.584590000
1	132.246057000	4.660871000	3.123213000
7	135.453063000	4.630605000	5.479592000
7	133.263473000	6.426459000	5.162151000
7	133.655041000	2.595834000	6.335147000
6	136.083788000	2.350617000	6.113857000
6	135.613732000	6.947441000	4.704448000
6	130.835457000	6.671848000	5.385433000
6	131.304975000	2.072190000	6.797823000
6	136.374816000	3.639892000	5.682506000
6	134.267322000	7.275543000	4.771115000
6	130.541509000	5.381525000	5.794754000
6	132.654041000	1.746833000	6.734146000
8	133.668912000	5.068656000	7.423541000
6	137.700821000	4.111051000	5.363931000
6	133.727908000	8.570208000	4.437638000
6	129.215779000	4.895911000	6.087673000
6	133.196572000	0.453978000	7.070871000
6	137.566758000	5.404128000	4.963253000
6	132.383535000	8.496772000	4.638252000
6	129.349481000	3.603114000	6.487884000
6	134.541065000	0.528846000	6.869875000
6	136.160701000	5.715284000	5.039703000
6	132.106808000	7.155455000	5.090032000
6	130.756586000	3.292532000	6.438357000
6	134.818205000	1.869235000	6.416428000
7	131.470847000	4.384492000	5.997782000
26	133.479601000	4.555505000	5.878948000
1	136.917137000	1.663865000	6.225928000
1	136.299842000	7.724275000	4.383880000
1	130.004111000	7.362114000	5.279477000
1	130.622551000	1.300872000	7.141009000
6	134.386954000	8.297514000	11.120250000
7	135.361700000	9.161984000	10.677354000
8	133.483178000	8.629421000	11.866045000
16	137.595673000	7.517339000	10.989259000
6	134.613403000	6.862180000	10.593947000
7	135.024677000	6.903335000	9.212516000
8	136.472654000	7.809090000	7.675416000
16	136.044076000	6.246307000	11.723875000
6	133.362493000	5.981303000	10.823673000
8	138.507152000	9.363884000	8.751117000
6	133.593849000	4.557088000	11.292617000
6	133.543641000	4.256894000	12.661718000
6	133.745839000	2.951593000	13.114620000
6	133.990266000	1.925613000	12.200818000
6	134.029533000	2.212323000	10.833910000
6	133.831874000	3.517501000	10.382035000
6	136.000661000	7.755605000	8.812501000
6	136.490779000	8.664286000	9.947117000
6	137.354562000	9.816459000	9.408946000
1	133.342430000	5.052149000	13.375740000
1	133.705093000	2.737649000	14.179452000
1	132.755257000	6.516311000	11.558165000
1	132.806805000	5.980555000	9.879236000
1	134.143196000	0.907921000	12.550978000
1	134.208894000	1.417903000	10.113668000
1	133.852686000	3.734646000	9.317298000
1	136.705090000	10.420222000	8.754275000
1	137.678710000	10.444274000	10.246193000
1	135.451184000	10.029443000	11.196934000
1	134.685574000	6.194273000	8.550139000
1	138.172489000	8.781998000	8.039416000
1	138.602063000	3.517184000	5.443448000
1	134.316021000	9.413118000	4.098694000
1	128.311918000	5.484540000	5.997620000
1	132.609733000	-0.391010000	7.407129000
1	138.334765000	6.098109000	4.648074000
1	131.635388000	9.265991000	4.496185000
1	128.577949000	2.908422000	6.793785000
1	135.288753000	-0.241681000	7.007101000

^2Re_A:

16	132.947611000	3.644591000	3.569658000
1	132.231534000	4.704593000	3.131134000
7	135.482579000	4.576956000	5.439577000
7	133.331600000	6.422315000	5.178231000
7	133.657105000	2.571694000	6.302135000
6	136.073992000	2.274546000	6.033690000
6	135.685181000	6.901836000	4.697361000
6	130.910592000	6.715839000	5.436078000
6	131.305620000	2.100192000	6.814750000
6	136.384812000	3.561850000	5.610170000
6	134.346706000	7.256560000	4.785178000
6	130.594634000	5.430025000	5.843618000
6	132.645526000	1.745330000	6.721089000
8	133.716822000	5.017202000	7.427996000
6	137.714910000	4.006849000	5.273335000
6	133.828977000	8.564407000	4.470936000
6	129.264922000	4.973587000	6.164726000
6	133.165407000	0.439307000	7.043438000
6	137.604542000	5.309475000	4.896494000
6	132.485473000	8.514089000	4.684414000
6	129.378321000	3.676416000	6.557145000
6	134.506570000	0.483724000	6.814148000
6	136.208360000	5.652652000	5.004271000
6	132.187652000	7.174234000	5.126518000
6	130.776682000	3.334216000	6.473470000
6	134.804634000	1.818993000	6.358648000
7	131.504628000	4.410968000	6.018965000
26	133.521497000	4.542326000	5.873841000
1	136.894323000	1.569048000	6.123016000
1	136.383416000	7.668508000	4.378493000
1	130.093495000	7.425413000	5.348351000
1	130.614020000	1.342692000	7.170134000
6	134.292837000	8.299002000	11.110177000
7	135.245871000	9.186361000	10.664702000
8	133.375853000	8.612677000	11.847657000
16	137.521174000	7.605315000	11.001940000
6	134.559847000	6.866871000	10.594105000
7	134.975434000	6.911250000	9.214297000
8	136.410174000	7.840883000	7.679290000
16	136.000708000	6.294990000	11.733175000
6	133.331598000	5.954877000	10.824386000
8	138.394819000	9.458894000	8.752566000
6	133.596837000	4.539090000	11.300844000
6	133.549944000	4.244712000	12.671334000
6	133.781951000	2.946926000	13.131622000
6	134.053072000	1.922335000	12.223844000
6	134.089212000	2.203061000	10.855617000
6	133.862011000	3.500804000	10.396277000
6	135.932815000	7.784466000	8.813944000
6	136.392278000	8.713862000	9.944580000
6	137.227612000	9.884976000	9.402300000
1	133.328086000	5.038482000	13.380856000
1	133.743552000	2.737637000	14.197471000
1	132.708299000	6.477372000	11.554483000
1	132.778698000	5.935715000	9.878419000
1	134.229030000	0.910335000	12.579628000
1	134.288976000	1.409418000	10.139919000
1	133.881425000	3.712838000	9.330491000
1	136.565186000	10.466561000	8.740493000
1	137.530998000	10.527188000	10.236354000
1	135.308674000	10.059895000	11.178134000
1	134.660666000	6.188384000	8.554455000
1	138.078795000	8.862567000	8.044205000
1	138.602997000	3.390529000	5.325456000
1	134.429585000	9.399503000	4.134626000
1	128.372832000	5.582911000	6.098015000
1	132.566540000	-0.392749000	7.390556000
1	138.382948000	5.989680000	4.576652000
1	131.750817000	9.298860000	4.557837000
1	128.598432000	2.998303000	6.878692000
1	135.239110000	-0.304047000	6.934096000

^{4}TS1$_{A}$:

16	133.075223000	4.424256000	3.727181000
1	131.746746000	4.553833000	3.939243000
7	135.620245000	4.590609000	5.182864000
7	133.589014000	6.439280000	5.945469000
7	133.942716000	2.416206000	5.943783000
6	136.246781000	2.220082000	5.141598000
6	135.765863000	7.029893000	4.990395000
6	131.303438000	6.643984000	6.808051000
6	131.690330000	1.819773000	6.697024000
6	136.501086000	3.569046000	4.935882000
6	134.515643000	7.346853000	5.503664000
6	131.003351000	5.288275000	6.894830000
6	132.981894000	1.506584000	6.296211000
8	134.369345000	4.442432000	7.598325000
6	137.741911000	4.095105000	4.423026000
6	134.008746000	8.688768000	5.650622000
6	129.742776000	4.759748000	7.349888000
6	133.499935000	0.164251000	6.185456000
6	137.599476000	5.446841000	4.365596000
6	132.761367000	8.582233000	6.185028000
6	129.848166000	3.402418000	7.304491000
6	134.787721000	0.277460000	5.760260000
6	136.275933000	5.745346000	4.851384000
6	132.502774000	7.173660000	6.352460000
6	131.175482000	3.104411000	6.829933000
6	135.049285000	1.688127000	5.606357000
7	131.858676000	4.264967000	6.570721000
26	133.784478000	4.428238000	5.984096000
1	137.043749000	1.522095000	4.905011000
1	136.405549000	7.852886000	4.690004000
1	130.531832000	7.345683000	7.108212000
1	131.030966000	0.993409000	6.943679000
6	133.822761000	8.419652000	9.865306000
7	134.922220000	9.162657000	9.502092000
8	132.712271000	8.898106000	10.028946000
16	136.887173000	8.031043000	11.133299000
6	134.184999000	6.932823000	10.030087000
7	135.031999000	6.446273000	9.014346000
8	136.934435000	6.907267000	7.807539000
16	135.181920000	7.040109000	11.789511000
6	132.945287000	6.065210000	10.308329000
8	138.526750000	8.918852000	8.770470000
6	133.132022000	4.866531000	11.219584000
6	132.606409000	4.898931000	12.519333000
6	132.758849000	3.812012000	13.382892000
6	133.435857000	2.670443000	12.953195000
6	133.953697000	2.623416000	11.656187000
6	133.802695000	3.709416000	10.793590000
6	136.080354000	7.206842000	8.659034000
6	136.207043000	8.551002000	9.415084000
6	137.242955000	9.480121000	8.766557000
1	132.073000000	5.784636000	12.857456000
1	132.346659000	3.858800000	14.387591000
1	132.183387000	6.730088000	10.722644000
1	132.585168000	5.740463000	9.326030000
1	133.556072000	1.821802000	13.621710000
1	134.477780000	1.735315000	11.311908000
1	134.198528000	3.659129000	9.783650000
1	136.878442000	9.693168000	7.748165000
1	137.290580000	10.425135000	9.319920000
1	134.842963000	10.169203000	9.602369000
1	134.691125000	5.323707000	8.164717000
1	138.412920000	8.067344000	8.295641000
1	138.599385000	3.493345000	4.151285000
1	134.550156000	9.583664000	5.372050000
1	128.900065000	5.361989000	7.663670000
1	132.937801000	-0.733447000	6.408187000
1	138.317543000	6.189262000	4.043175000
1	132.065539000	9.369172000	6.444287000
1	129.110083000	2.658839000	7.575898000
1	135.504451000	-0.507902000	5.558022000

^{2}TS1$_{A}$:

16	133.038186000	4.400901000	3.716685000
1	131.705772000	4.409147000	3.943655000
7	135.601734000	4.605224000	5.159988000
7	133.591200000	6.460966000	5.948271000
7	133.918723000	2.436485000	5.930472000
6	136.213748000	2.229987000	5.107615000
6	135.759571000	7.042480000	4.967895000
6	131.309379000	6.677458000	6.820746000
6	131.677264000	1.851948000	6.722787000
6	136.473332000	3.578380000	4.902654000
6	134.516844000	7.364231000	5.497765000
6	131.003090000	5.323611000	6.910896000
6	132.959976000	1.531681000	6.300696000
8	134.372121000	4.440837000	7.594774000
6	137.712063000	4.097016000	4.377844000
6	134.017685000	8.708008000	5.648348000
6	129.746230000	4.801126000	7.382447000
6	133.470434000	0.186665000	6.183567000
6	137.577734000	5.449738000	4.322536000
6	132.773536000	8.606868000	6.192071000
6	129.848142000	3.443322000	7.344630000
6	134.751965000	0.293474000	5.739340000
6	136.261537000	5.755990000	4.822659000
6	132.510064000	7.199984000	6.360936000
6	131.168933000	3.139024000	6.856276000
6	135.018332000	1.702993000	5.580918000
7	131.851621000	4.296714000	6.581089000
26	133.776245000	4.455741000	5.983194000
1	137.005436000	1.528879000	4.862712000
1	136.398711000	7.863029000	4.659736000
1	130.543606000	7.383693000	7.125045000
1	131.018618000	1.029465000	6.983890000
6	133.817049000	8.397159000	9.887885000
7	134.907841000	9.150325000	9.519388000
8	132.703602000	8.865791000	10.060003000
16	136.894229000	8.027811000	11.130829000
6	134.192644000	6.912912000	10.045737000
7	135.035664000	6.436130000	9.022697000
8	136.922373000	6.917185000	7.799026000
16	135.200969000	7.026028000	11.799620000
6	132.962272000	6.034697000	10.331247000
8	138.507665000	8.935512000	8.756703000
6	133.167863000	4.830052000	11.230530000
6	132.654239000	4.847378000	12.535344000
6	132.823888000	3.754843000	13.388535000
6	133.506400000	2.622595000	12.943192000
6	134.012361000	2.590549000	11.641053000
6	133.844238000	3.682196000	10.788795000
6	136.074106000	7.206495000	8.660054000
6	136.196485000	8.549042000	9.419518000
6	137.220203000	9.488261000	8.766491000
1	132.116575000	5.725757000	12.885668000
1	132.420766000	3.789995000	14.397381000
1	132.200514000	6.690703000	10.759723000
1	132.592961000	5.715640000	9.350411000
1	133.640087000	1.769613000	13.603580000
1	134.540685000	1.709768000	11.284645000
1	134.231147000	3.643579000	9.774936000
1	136.845337000	9.703236000	7.752260000
1	137.266234000	10.431140000	9.323628000
1	134.821206000	10.155934000	9.622795000
1	134.692337000	5.318775000	8.166158000
1	138.394913000	8.085203000	8.279268000
1	138.562701000	3.489911000	4.096718000
1	134.560774000	9.600328000	5.364881000
1	128.908801000	5.407394000	7.702495000
1	132.907481000	-0.708119000	6.415755000
1	138.296950000	6.187788000	3.992726000
1	132.082591000	9.396673000	6.455624000
1	129.111635000	2.703134000	7.629248000
1	135.462215000	-0.495307000	5.527815000

$^{4}IM1_{A}$:

16	133.284475000	4.146945000	3.411115000
1	131.947192000	4.315482000	3.509999000
7	135.702097000	4.209629000	5.036498000
7	133.843285000	6.324753000	5.507555000
7	133.752006000	2.302634000	5.869061000
6	136.066122000	1.788534000	5.236623000
6	136.145817000	6.604317000	4.704575000
6	131.512889000	6.833721000	6.089406000
6	131.396059000	2.003577000	6.483124000
6	136.475667000	3.081145000	4.941878000
6	134.902847000	7.087196000	5.083898000
6	131.070406000	5.530357000	6.267540000
6	132.674177000	1.534129000	6.222310000
8	134.277834000	4.416870000	7.383633000
6	137.803925000	3.432931000	4.506060000
6	134.529404000	8.479278000	5.064724000
6	129.731537000	5.174156000	6.664466000
6	133.054061000	0.141925000	6.263980000
6	137.823977000	4.785230000	4.349708000
6	133.229191000	8.545466000	5.463456000
6	129.697503000	3.816443000	6.768298000
6	134.368847000	0.082538000	5.920079000
6	136.509136000	5.266049000	4.693015000
6	132.802440000	7.192544000	5.723808000
6	131.016187000	3.337467000	6.438699000
6	134.795267000	1.437637000	5.664013000
7	131.830776000	4.394984000	6.116355000
26	133.795082000	4.304152000	5.667018000
1	136.792984000	0.992258000	5.113280000
1	136.894931000	7.329159000	4.403883000
1	130.790296000	7.631330000	6.230315000
1	130.637964000	1.275373000	6.752715000
6	133.599008000	8.048022000	10.945281000
7	134.590667000	8.998810000	10.897549000
8	132.608909000	8.158650000	11.649534000
16	137.250085000	8.074872000	11.308008000
6	133.784475000	6.891042000	9.974160000
7	134.708130000	6.871478000	9.069556000
8	136.384692000	8.014684000	8.033905000
16	136.320703000	6.851070000	12.546461000
6	132.800984000	5.760200000	10.084033000
8	137.695526000	10.116398000	9.269569000
6	133.265748000	4.747523000	11.132170000
6	132.899909000	4.884840000	12.477053000
6	133.335560000	3.953850000	13.421751000
6	134.143748000	2.883603000	13.031090000
6	134.512725000	2.746817000	11.690963000
6	134.079817000	3.675220000	10.742448000
6	135.655361000	7.904684000	9.007507000
6	135.805666000	8.861508000	10.185830000
6	136.379640000	10.223520000	9.743067000
1	132.278823000	5.722978000	12.780757000
1	133.041598000	4.064554000	14.462312000
1	131.828656000	6.170953000	10.368218000
1	132.733426000	5.274680000	9.107923000
1	134.482349000	2.159332000	13.767380000
1	135.138037000	1.914102000	11.379686000
1	134.358935000	3.569817000	9.696677000
1	135.693267000	10.645854000	8.991897000
1	136.397897000	10.899864000	10.605154000
1	134.512079000	9.732018000	11.596277000
1	134.567004000	5.321319000	7.633346000
1	137.651836000	9.496007000	8.516123000
1	138.606666000	2.723317000	4.351560000
1	135.189018000	9.286342000	4.772876000
1	128.934936000	5.884996000	6.844067000
1	132.384974000	-0.669475000	6.520626000
1	138.646802000	5.417580000	4.041767000
1	132.595561000	9.418121000	5.560584000
1	128.867859000	3.181878000	7.052823000
1	135.006373000	-0.788070000	5.833422000

$^{2}IM1_{A}$:

16	133.461029000	3.997741000	3.410924000
1	132.123216000	4.189247000	3.432503000
7	135.796096000	4.226561000	5.139686000
7	133.810742000	6.254593000	5.457202000
7	133.910858000	2.246946000	5.945068000
6	136.280455000	1.839567000	5.454608000
6	136.123648000	6.627724000	4.726070000
6	131.439436000	6.658682000	5.946691000
6	131.543412000	1.837897000	6.441779000
6	136.634170000	3.141265000	5.128341000
6	134.844077000	7.057144000	5.043219000
6	131.055801000	5.339038000	6.139525000
6	132.856146000	1.430934000	6.260702000
8	134.254937000	4.437222000	7.412855000
6	137.959622000	3.549692000	4.735623000
6	134.401632000	8.426913000	4.967471000
6	129.719690000	4.924303000	6.486195000
6	133.305546000	0.063098000	6.365977000
6	137.911796000	4.893005000	4.518919000
6	133.087669000	8.438371000	5.324680000
6	129.746384000	3.568444000	6.612572000
6	134.639841000	0.065809000	6.101148000
6	136.558532000	5.312273000	4.784399000
6	132.721303000	7.073675000	5.613919000
6	131.100261000	3.149637000	6.350123000
6	135.008576000	1.434116000	5.826833000
7	131.876086000	4.239822000	6.043374000
26	133.859702000	4.239250000	5.681873000
1	137.054069000	1.080674000	5.398232000
1	136.844771000	7.380501000	4.425585000
1	130.673223000	7.421607000	6.041716000
1	130.810653000	1.077188000	6.690859000
6	133.540008000	8.142272000	10.901486000
7	134.548748000	9.075275000	10.861064000
8	132.544965000	8.272522000	11.595265000
16	137.189877000	8.102361000	11.281504000
6	133.715210000	6.979068000	9.935906000
7	134.642465000	6.944644000	9.035550000
8	136.329598000	8.071696000	7.998825000
16	136.232768000	6.898320000	12.518142000
6	132.715624000	5.862295000	10.044480000
8	137.680155000	10.139895000	9.248329000
6	133.142058000	4.861535000	11.120054000
6	132.740513000	5.021911000	12.452103000
6	133.140722000	4.100896000	13.421841000
6	133.948891000	3.017472000	13.069258000
6	134.353402000	2.857576000	11.742034000
6	133.956006000	3.775898000	10.768444000
6	135.601603000	7.967033000	8.973986000
6	135.764590000	8.918026000	10.155123000
6	136.365286000	10.270459000	9.718869000
1	132.120010000	5.870457000	12.726400000
1	132.819215000	4.229670000	14.452124000
1	131.742932000	6.290906000	10.300472000
1	132.662128000	5.361470000	9.075334000
1	134.259894000	2.300877000	13.824990000
1	134.978861000	2.014514000	11.460272000
1	134.262614000	3.652266000	9.732379000
1	135.689134000	10.708632000	8.967530000
1	136.394250000	10.943169000	10.583492000
1	134.477395000	9.811144000	11.557741000
1	134.526760000	5.354507000	7.632448000
1	137.626102000	9.524970000	8.491133000
1	138.806480000	2.881210000	4.647486000
1	135.028745000	9.257635000	4.670281000
1	128.882558000	5.598103000	6.617858000
1	132.665999000	-0.775881000	6.608917000
1	138.711580000	5.557720000	4.218569000
1	132.408346000	9.280041000	5.375202000
1	128.936668000	2.898628000	6.872588000
1	135.326296000	-0.770964000	6.078354000

^{4}TS2$_{A}$:

16	133.289571000	4.387634000	3.694688000
1	132.007548000	4.645758000	4.036465000
7	135.939606000	4.055388000	4.866586000
7	134.536910000	6.418912000	5.636242000
7	133.887739000	2.451163000	6.036392000
6	135.943718000	1.602038000	5.002929000
6	136.694044000	6.358103000	4.464130000
6	132.438838000	7.269561000	6.590248000
6	131.659890000	2.507028000	7.063029000
6	136.497244000	2.822496000	4.643685000
6	135.613779000	7.017174000	5.031837000
6	131.850289000	6.046902000	6.881499000
6	132.780150000	1.851077000	6.575062000
8	134.993083000	4.409682000	7.424204000
6	137.775307000	2.973439000	3.992635000
6	135.444638000	8.448737000	5.029640000
6	130.570602000	5.895885000	7.527941000
6	132.934245000	0.415500000	6.545667000
6	137.985708000	4.309344000	3.839003000
6	134.244916000	8.704777000	5.621714000
6	130.346246000	4.556161000	7.648325000
6	134.141298000	0.161466000	5.972099000
6	136.837905000	4.980388000	4.397282000
6	133.679985000	7.429864000	5.991541000
6	131.490396000	3.885062000	7.083062000
6	134.726408000	1.440740000	5.646699000
7	132.388208000	4.811601000	6.610009000
26	134.212168000	4.431001000	5.823969000
1	136.503232000	0.706073000	4.754243000
1	137.483479000	6.966069000	4.034437000
1	131.880756000	8.164643000	6.844501000
1	130.856318000	1.897981000	7.464629000
6	132.386197000	8.237158000	10.285412000
7	133.401077000	9.032522000	9.799149000
8	131.199205000	8.495544000	10.197318000
16	135.372548000	8.697217000	11.765738000
6	132.970183000	6.989701000	10.962226000
7	133.967545000	6.390545000	10.153788000
8	135.829578000	6.755699000	8.834752000
16	133.679301000	7.753312000	12.598665000
6	131.953139000	5.897517000	11.349595000
8	137.062773000	9.203272000	9.359391000
6	132.796670000	4.656471000	11.478919000
6	133.067298000	4.011747000	12.671310000
6	134.128242000	3.091657000	12.754439000
6	134.956652000	2.849935000	11.643233000
6	134.702440000	3.469917000	10.432235000
6	133.598052000	4.367151000	10.314412000
6	134.913790000	7.135906000	9.582590000
6	134.769378000	8.641322000	9.954742000
6	135.716615000	9.517548000	9.119276000
1	132.491911000	4.253728000	13.561014000
1	134.332163000	2.594511000	13.698550000
1	131.409242000	6.152620000	12.262571000
1	131.223532000	5.822432000	10.534118000
1	135.782456000	2.149642000	11.732018000
1	135.283433000	3.262277000	9.540101000
1	133.172650000	4.478576000	9.322925000
1	135.436884000	9.384206000	8.064218000
1	135.571199000	10.568105000	9.394558000
1	133.169976000	9.994267000	9.575574000
1	135.340136000	5.281499000	7.727518000
1	137.154218000	8.270779000	9.077899000
1	138.417545000	2.151072000	3.704506000
1	136.154902000	9.150168000	4.610995000
1	129.950579000	6.720016000	7.856697000
1	132.199694000	-0.288809000	6.914981000
1	138.836777000	4.813208000	3.399072000
1	133.760961000	9.660166000	5.781140000
1	129.492698000	4.052196000	8.083967000
1	134.605973000	-0.795276000	5.770501000

^{2}TS2$_{A}$:

16	133.466859000	4.223963000	3.701715000
1	132.165629000	4.453396000	3.986896000
7	136.085741000	4.050052000	4.974311000
7	134.548202000	6.365791000	5.621454000
7	134.069556000	2.385104000	6.121104000
6	136.200362000	1.605139000	5.189274000
6	136.743699000	6.373123000	4.521657000
6	132.383583000	7.143189000	6.485168000
6	131.804910000	2.364021000	7.065160000
6	136.708856000	2.839412000	4.811012000
6	135.615101000	6.996540000	5.032806000
6	131.841826000	5.902816000	6.791489000
6	132.971607000	1.748524000	6.637208000
8	135.035075000	4.434762000	7.484324000
6	138.000734000	3.031910000	4.199541000
6	135.380041000	8.417834000	4.982272000
6	130.548459000	5.710129000	7.398520000
6	133.193437000	0.321699000	6.657251000
6	138.152705000	4.371010000	4.009041000
6	134.151970000	8.634009000	5.530715000
6	130.379989000	4.365057000	7.547147000
6	134.431937000	0.109109000	6.136300000
6	136.955158000	5.002607000	4.505357000
6	133.634830000	7.344961000	5.920787000
6	131.572271000	3.732685000	7.039659000
6	134.968603000	1.404925000	5.793293000
7	132.443656000	4.686740000	6.572026000
26	134.309442000	4.370880000	5.859396000
1	136.809916000	0.729947000	4.988982000
1	137.517874000	7.004884000	4.098582000
1	131.777840000	8.017836000	6.697954000
1	131.016073000	1.729237000	7.455934000
6	132.196982000	8.240619000	10.107565000
7	133.199184000	9.061534000	9.638049000
8	131.005456000	8.445628000	9.959421000
16	135.097005000	8.869613000	11.694110000
6	132.800763000	7.041360000	10.850834000
7	133.854919000	6.458382000	10.105447000
8	135.752204000	6.855997000	8.847816000
16	133.407781000	7.885190000	12.487890000
6	131.813065000	5.920766000	11.233016000
8	136.866694000	9.365502000	9.342395000
6	132.699707000	4.720937000	11.440597000
6	132.943325000	4.127893000	12.665270000
6	134.035576000	3.256032000	12.825376000
6	134.920254000	3.011693000	11.759069000
6	134.693802000	3.579380000	10.517245000
6	133.560646000	4.425211000	10.321558000
6	134.793073000	7.222655000	9.546411000
6	134.574120000	8.732341000	9.863114000
6	135.520622000	9.619702000	9.038765000
1	132.320743000	4.375860000	13.520896000
1	134.218167000	2.799765000	13.794171000
1	131.220401000	6.182522000	12.113201000
1	131.123295000	5.787516000	10.390735000
1	135.768763000	2.349572000	11.906958000
1	135.320663000	3.365452000	9.658308000
1	133.175412000	4.486149000	9.309399000
1	135.289416000	9.443793000	7.978251000
1	135.324229000	10.671579000	9.274534000
1	132.939717000	10.005034000	9.371917000
1	135.347170000	5.327563000	7.763025000
1	137.004015000	8.428760000	9.095622000
1	138.691306000	2.233198000	3.960928000
1	136.070013000	9.139580000	4.564185000
1	129.881292000	6.513413000	7.683630000
1	132.478962000	-0.405832000	7.020903000
1	138.994054000	4.901653000	3.582101000
1	133.619818000	9.569803000	5.646784000
1	129.535230000	3.833856000	7.967271000
1	134.948057000	-0.829797000	5.981406000

^{4}IM2$_{A}$:

16	133.276285000	4.335022000	3.699432000
1	132.000752000	4.631973000	4.033960000
7	135.927789000	4.038068000	4.871513000
7	134.501319000	6.393821000	5.624394000
7	133.901605000	2.423334000	6.066637000
6	135.969032000	1.587836000	5.044304000
6	136.650527000	6.346331000	4.436616000
6	132.395637000	7.226055000	6.577816000
6	131.677816000	2.460265000	7.101629000
6	136.503223000	2.810727000	4.664027000
6	135.567498000	6.999412000	5.006617000
6	131.821303000	5.998604000	6.875617000
6	132.806534000	1.814694000	6.620859000
8	134.978626000	4.413126000	7.428317000
6	137.775610000	2.970814000	4.005104000
6	135.383926000	8.428741000	4.998290000
6	130.545735000	5.838744000	7.528768000
6	132.984668000	0.381614000	6.617804000
6	137.965274000	4.307439000	3.830448000
6	134.185552000	8.676262000	5.598326000
6	130.338925000	4.498089000	7.664170000
6	134.193709000	0.137053000	6.045142000
6	136.810731000	4.969790000	4.384482000
6	133.634918000	7.398214000	5.977024000
6	131.489191000	3.835526000	7.101735000
6	134.756532000	1.419498000	5.693956000
7	132.371700000	4.766740000	6.611339000
26	134.194423000	4.398972000	5.824967000
1	136.541593000	0.696434000	4.809569000
1	137.429922000	6.958553000	3.994758000
1	131.826917000	8.115006000	6.829752000
1	130.886295000	1.845910000	7.518427000
6	132.326068000	8.184579000	10.159206000
7	133.350359000	8.971575000	9.682220000
8	131.142309000	8.405808000	9.976908000
16	135.188885000	8.819023000	11.804962000
6	132.881143000	7.017690000	10.980034000
7	133.981784000	6.371306000	10.296987000
8	135.880069000	6.701503000	9.037610000
16	133.451784000	7.876322000	12.588316000
6	131.891902000	5.883968000	11.297685000
8	137.027571000	9.239960000	9.480303000
6	132.804035000	4.694188000	11.387175000
6	132.826294000	3.699571000	12.321827000
6	133.966278000	2.866064000	12.455155000
6	135.149028000	3.163148000	11.725131000
6	135.196656000	4.165088000	10.795971000
6	133.933119000	4.875270000	10.403292000
6	134.926314000	7.128794000	9.699486000
6	134.713928000	8.631717000	9.956881000
6	135.689485000	9.486530000	9.134071000
1	131.996380000	3.588882000	13.016455000
1	133.967098000	2.063678000	13.185821000
1	131.312584000	6.071093000	12.205426000
1	131.186236000	5.822664000	10.457037000
1	136.047432000	2.585405000	11.927821000
1	136.095540000	4.384323000	10.232752000
1	133.657662000	4.514478000	9.394900000
1	135.486384000	9.285248000	8.071831000
1	135.491163000	10.544619000	9.336020000
1	133.112204000	9.905564000	9.366064000
1	135.371541000	5.280313000	7.661479000
1	137.181180000	8.301410000	9.258424000
1	138.428997000	2.153938000	3.726684000
1	136.083259000	9.135204000	4.569715000
1	129.920057000	6.659373000	7.854882000
1	132.263674000	-0.327392000	7.004251000
1	138.807116000	4.817110000	3.379576000
1	133.693232000	9.627953000	5.754383000
1	129.494944000	3.987724000	8.110706000
1	134.674575000	-0.815189000	5.861260000

^{2}IM2$_{A}$:

16	133.414223000	4.207544000	3.699761000
1	132.127858000	4.529011000	3.962807000
7	136.020673000	4.014950000	4.991046000
7	134.500889000	6.350017000	5.612472000
7	133.986918000	2.378004000	6.143235000
6	136.122566000	1.573553000	5.242263000
6	136.691688000	6.328497000	4.503090000
6	132.345546000	7.149754000	6.477973000
6	131.717053000	2.379719000	7.073001000
6	136.639186000	2.799562000	4.847451000
6	135.571112000	6.967325000	5.013563000
6	131.790131000	5.915226000	6.781582000
6	132.882407000	1.753080000	6.659371000
8	134.952311000	4.438797000	7.494075000
6	137.931772000	2.976379000	4.233905000
6	135.351418000	8.390607000	4.961650000
6	130.494251000	5.738313000	7.388966000
6	133.098029000	0.325858000	6.701973000
6	138.088622000	4.311555000	4.019546000
6	134.129352000	8.621992000	5.519150000
6	130.311227000	4.396137000	7.540539000
6	134.340280000	0.100720000	6.196348000
6	136.894120000	4.956463000	4.505056000
6	133.599168000	7.339931000	5.912969000
6	131.497131000	3.749692000	7.036002000
6	134.885604000	1.388963000	5.839010000
7	132.377427000	4.691410000	6.562373000
26	134.237121000	4.353725000	5.858390000
1	136.729101000	0.692420000	5.059888000
1	137.469372000	6.949003000	4.069844000
1	131.748354000	8.029424000	6.693793000
1	130.922798000	1.754905000	7.468482000
6	132.214360000	8.166309000	10.005012000
7	133.224479000	8.980373000	9.543190000
8	131.030208000	8.338912000	9.777916000
16	134.985576000	8.952754000	11.735805000
6	132.782545000	7.041574000	10.874240000
7	133.934466000	6.424756000	10.253138000
8	135.860988000	6.794620000	9.050578000
16	133.256461000	7.963812000	12.479953000
6	131.827553000	5.875866000	11.180302000
8	136.893652000	9.387435000	9.468526000
6	132.781837000	4.726080000	11.337061000
6	132.796526000	3.747922000	12.289617000
6	133.962766000	2.968138000	12.497391000
6	135.169558000	3.307872000	11.827826000
6	135.221912000	4.296223000	10.884343000
6	133.949438000	4.933473000	10.406740000
6	134.869127000	7.202822000	9.666815000
6	134.588859000	8.701530000	9.875128000
6	135.561223000	9.573353000	9.066899000
1	131.937441000	3.613488000	12.943353000
1	133.959746000	2.178424000	13.241758000
1	131.204470000	6.059942000	12.059218000
1	131.159586000	5.765668000	10.314284000
1	136.081095000	2.777231000	12.091499000
1	136.140035000	4.551734000	10.369105000
1	133.749208000	4.528890000	9.394830000
1	135.405738000	9.338088000	8.003560000
1	135.315688000	10.627655000	9.233605000
1	132.962056000	9.897579000	9.198335000
1	135.366947000	5.305205000	7.688283000
1	137.090728000	8.449990000	9.278361000
1	138.619383000	2.171068000	4.009435000
1	136.045864000	9.104031000	4.536646000
1	129.839111000	6.550408000	7.675961000
1	132.377699000	-0.392925000	7.071227000
1	138.932235000	4.831255000	3.583685000
1	133.608867000	9.564303000	5.635682000
1	129.461993000	3.874936000	7.963995000
1	134.854706000	-0.842160000	6.061627000

^{4}TS3$_{A}$:

16	133.313721000	3.917977000	3.673359000
1	132.005369000	4.123066000	3.945015000
7	135.978545000	3.894310000	5.102816000
7	134.405730000	6.218621000	5.609619000
7	133.965286000	2.253049000	6.302842000
6	136.121421000	1.463163000	5.444215000
6	136.612803000	6.201630000	4.538461000
6	132.227263000	7.007206000	6.427214000
6	131.685032000	2.249964000	7.205617000
6	136.617598000	2.683601000	5.005818000
6	135.477235000	6.839195000	5.017474000
6	131.680722000	5.772613000	6.759941000
6	132.868684000	1.628283000	6.833410000
8	134.918574000	4.433512000	7.665512000
6	137.910390000	2.860710000	4.392850000
6	135.242159000	8.260199000	4.940762000
6	130.375524000	5.589778000	7.343943000
6	133.110870000	0.207225000	6.933457000
6	138.046338000	4.190601000	4.131784000
6	134.011417000	8.486272000	5.478688000
6	130.220867000	4.251027000	7.554376000
6	134.361636000	-0.013158000	6.445326000
6	136.838309000	4.832078000	4.587471000
6	133.488975000	7.203199000	5.883159000
6	131.432128000	3.612609000	7.104971000
6	134.884024000	1.272275000	6.042736000
7	132.300330000	4.555209000	6.609678000
26	134.166995000	4.225430000	5.899110000
1	136.746432000	0.588428000	5.294921000
1	137.386207000	6.820109000	4.094451000
1	131.610445000	7.885463000	6.590825000
1	130.896907000	1.624973000	7.613645000
6	132.556263000	8.626689000	10.897336000
7	133.528650000	9.423162000	10.332656000
8	131.399493000	8.960236000	11.069285000
16	135.739142000	8.738235000	11.929950000
6	133.140552000	7.273010000	11.311408000
7	133.993215000	6.732050000	10.272309000
8	135.669360000	7.181317000	8.763551000
16	134.138109000	7.698040000	12.878530000
6	132.106906000	6.169263000	11.600880000
8	137.092401000	9.536428000	9.373971000
6	132.799088000	4.924259000	11.120318000
6	132.736172000	3.646058000	11.555924000
6	133.690295000	2.682252000	11.068810000
6	134.797296000	3.074847000	10.325717000
6	134.945599000	4.391761000	9.872136000
6	133.750846000	5.298593000	10.011028000
6	134.894446000	7.532127000	9.658038000
6	134.880210000	8.957770000	10.230885000
6	135.739582000	9.906175000	9.379446000
1	132.028883000	3.350872000	12.326887000
1	133.614966000	1.655195000	11.413571000
1	131.813050000	6.131391000	12.653291000
1	131.203419000	6.405503000	11.020828000
1	135.598703000	2.365502000	10.142137000
1	135.923195000	4.800893000	9.664114000
1	133.211177000	5.229406000	9.051612000
1	135.302657000	9.915776000	8.368878000
1	135.678966000	10.915934000	9.798722000
1	133.330553000	10.416535000	10.270618000
1	135.305058000	5.333171000	7.671894000
1	137.117620000	8.654441000	8.955560000
1	138.612969000	2.060319000	4.198669000
1	135.934124000	8.975470000	4.514568000
1	129.679803000	6.391006000	7.559562000
1	132.400753000	-0.512302000	7.321172000
1	138.883774000	4.707318000	3.680515000
1	133.480642000	9.425012000	5.577228000
1	129.373059000	3.730270000	7.981363000
1	134.892902000	-0.951431000	6.347667000

^{2}TS3$_{A}$:

16	134.137855000	3.421345000	3.692331000
1	133.753515000	4.477596000	2.941031000
7	135.759716000	4.790197000	5.919493000
7	133.437343000	6.130920000	4.938987000
7	134.176633000	2.490517000	6.515978000
6	136.586587000	2.719103000	6.942099000
6	135.726758000	7.007237000	4.861234000
6	130.994858000	6.000491000	4.778800000
6	131.907125000	1.557249000	6.470938000
6	136.753197000	4.007771000	6.448483000
6	134.359590000	7.095534000	4.628066000
6	130.832874000	4.712712000	5.270831000
6	133.272797000	1.474636000	6.705821000
8	133.507644000	5.092889000	7.419146000
6	138.020125000	4.694226000	6.367439000
6	133.697892000	8.252539000	4.068714000
6	129.562389000	4.030409000	5.352024000
6	133.937888000	0.317790000	7.256851000
6	137.781313000	5.889047000	5.756851000
6	132.364935000	7.978497000	4.066766000
6	129.818807000	2.774228000	5.807990000
6	135.248714000	0.654049000	7.413920000
6	136.366190000	5.939283000	5.476678000
6	132.212460000	6.649954000	4.615133000
6	131.245259000	2.690104000	6.016604000
6	135.390904000	2.012062000	6.944886000
7	131.838159000	3.881812000	5.695588000
26	133.795645000	4.306188000	5.769370000
1	137.467964000	2.211947000	7.322308000
1	136.340655000	7.851585000	4.563006000
1	130.099521000	6.533464000	4.473645000
1	131.308853000	0.674413000	6.674217000
6	134.348702000	8.795230000	11.820943000
7	135.454888000	9.162575000	11.084692000
8	133.615301000	9.568338000	12.405869000
16	137.224454000	7.080674000	11.757027000
6	134.188399000	7.271670000	11.836574000
7	134.385215000	6.710308000	10.517645000
8	135.647702000	6.759020000	8.601636000
16	135.560345000	6.714726000	13.038311000
6	132.809047000	6.755718000	12.283795000
8	138.234514000	7.836509000	9.037750000
6	132.641757000	5.503436000	11.467375000
6	132.024910000	4.333602000	11.759710000
6	132.260376000	3.183863000	10.931949000
6	133.266478000	3.183711000	9.962770000
6	133.957707000	4.346248000	9.636053000
6	133.422066000	5.642828000	10.183643000
6	135.410874000	7.142570000	9.749560000
6	136.288950000	8.165690000	10.481464000
6	137.327731000	8.784870000	9.531818000
1	131.427095000	4.226804000	12.661296000
1	131.747282000	2.256482000	11.168416000
1	132.749439000	6.585393000	13.361932000
1	132.072327000	7.534288000	12.039608000
1	133.562190000	2.247227000	9.500758000
1	134.905368000	4.310280000	9.120532000
1	132.751141000	6.036191000	9.395208000
1	136.769106000	9.293257000	8.731065000
1	137.908625000	9.533638000	10.080542000
1	135.808290000	10.103752000	11.223366000
1	134.260199000	5.706325000	7.560204000
1	137.715213000	7.216709000	8.489871000
1	138.962730000	4.292374000	6.717097000
1	134.203299000	9.150786000	3.736873000
1	128.610696000	4.468176000	5.078284000
1	133.449586000	-0.619812000	7.491364000
1	138.488112000	6.669512000	5.503943000
1	131.547429000	8.603673000	3.730326000
1	129.121384000	1.966091000	5.989146000
1	136.058748000	0.048645000	7.800862000

$^{4}Pr_A$:

16	133.315231000	3.848814000	3.397312000
1	132.055628000	3.427966000	3.653366000
7	135.920115000	4.080558000	5.316373000
7	134.001661000	6.116840000	5.754002000
7	134.040594000	2.120063000	6.149875000
6	136.359551000	1.668590000	5.484650000
6	136.290870000	6.480831000	4.948394000
6	131.683618000	6.572238000	6.423440000
6	131.726626000	1.746762000	6.873978000
6	136.729400000	2.973996000	5.198337000
6	135.034145000	6.921254000	5.333367000
6	131.291753000	5.258771000	6.644068000
6	133.005614000	1.313710000	6.558780000
8	135.137040000	4.357783000	8.450436000
6	138.040569000	3.360623000	4.746773000
6	134.626836000	8.303688000	5.338957000
6	129.978576000	4.869151000	7.084239000
6	133.433232000	-0.062500000	6.611042000
6	138.021075000	4.713739000	4.591380000
6	133.332347000	8.330587000	5.761682000
6	129.990026000	3.511540000	7.207932000
6	134.737810000	-0.082762000	6.223235000
6	136.698493000	5.154788000	4.950861000
6	132.948008000	6.963294000	6.008403000
6	131.310905000	3.070307000	6.847397000
6	135.106365000	1.280305000	5.931668000
7	132.095170000	4.148147000	6.498912000
26	133.954968000	4.092550000	5.734584000
1	137.107483000	0.893956000	5.348667000
1	137.012285000	7.227832000	4.632904000
1	130.941910000	7.349601000	6.578578000
1	131.000217000	0.997939000	7.173581000
6	133.246447000	9.073123000	10.943310000
7	134.302643000	9.655243000	10.277647000
8	132.206491000	9.637327000	11.223456000
16	136.466290000	8.539377000	11.684574000
6	133.576385000	7.624354000	11.311867000
7	134.183759000	6.923846000	10.190476000
8	135.856388000	7.019868000	8.607267000
16	134.785287000	7.803107000	12.768951000
6	132.377417000	6.762419000	11.726465000
8	137.725493000	9.067839000	9.029570000
6	132.720329000	5.407680000	11.170900000
6	132.391606000	4.188366000	11.612806000
6	132.914397000	2.999293000	10.926202000
6	133.930330000	3.101988000	10.056480000
6	134.611563000	4.423202000	9.764345000
6	133.605629000	5.575918000	9.947329000
6	135.200450000	7.521194000	9.529143000
6	135.515668000	8.923681000	10.067801000
6	136.468225000	9.680795000	9.126834000
1	131.748728000	4.061789000	12.480618000
1	132.480655000	2.031031000	11.160531000
1	132.209392000	6.764932000	12.806730000
1	131.481814000	7.196537000	11.260457000
1	134.355499000	2.236433000	9.557943000
1	135.431030000	4.558253000	10.495648000
1	132.992565000	5.627290000	9.034144000
1	135.960115000	9.760010000	8.153295000
1	136.628307000	10.689372000	9.522188000
1	134.309368000	10.668522000	10.222029000
1	135.551363000	5.226882000	8.267215000
1	137.554772000	8.183451000	8.652819000
1	138.860426000	2.674078000	4.578500000
1	135.258308000	9.131877000	5.042574000
1	129.163359000	5.556397000	7.272111000
1	132.803446000	-0.892293000	6.905967000
1	138.821539000	5.367655000	4.269528000
1	132.679120000	9.185067000	5.886264000
1	129.186321000	2.856605000	7.519749000
1	135.402793000	-0.932298000	6.133592000

$^{2}Pr_A$:

16	134.188560000	3.424064000	3.742894000
1	134.925986000	4.431287000	3.222383000
7	135.629703000	5.084336000	5.925494000
7	133.204408000	6.103135000	4.924545000
7	134.277068000	2.676530000	6.727935000
6	136.656760000	3.176920000	7.087807000
6	135.366706000	7.255996000	4.819369000
6	130.819025000	5.613834000	4.591432000
6	132.164855000	1.433826000	6.623033000
6	136.698534000	4.428201000	6.485770000
6	133.999816000	7.174514000	4.593248000
6	130.796814000	4.326723000	5.110319000
6	133.504487000	1.564964000	6.965915000
8	133.378436000	5.450516000	7.762278000
6	137.894585000	5.225027000	6.357930000
6	133.213445000	8.206359000	3.961568000
6	129.626940000	3.480188000	5.127749000
6	134.286103000	0.539998000	7.613647000
6	137.538280000	6.368800000	5.704284000
6	131.934453000	7.745303000	3.908307000
6	130.006117000	2.295832000	5.680264000
6	135.544947000	1.037599000	7.754275000
6	136.123575000	6.272563000	5.442667000
6	131.939929000	6.428186000	4.500427000
6	131.405673000	2.421934000	6.009995000
6	135.535711000	2.363670000	7.183429000
7	131.867415000	3.669505000	5.663134000
26	133.760472000	4.351642000	5.736356000
1	137.586481000	2.791507000	7.494884000
1	135.878916000	8.156135000	4.493862000
1	129.886579000	6.007320000	4.198104000
1	131.675766000	0.492675000	6.854690000
6	135.209631000	8.143602000	12.385580000
7	136.244678000	8.518541000	11.556776000
8	134.804505000	8.796240000	13.327870000
16	137.503046000	6.030262000	11.171348000
6	134.660039000	6.771842000	11.980355000
7	134.484723000	6.680971000	10.539801000
8	135.466981000	7.085825000	8.500346000
16	135.993615000	5.569672000	12.604253000
6	133.292780000	6.416670000	12.574782000
8	138.388344000	7.418363000	8.767624000
6	132.625886000	5.644830000	11.469864000
6	131.705714000	4.676657000	11.536223000
6	131.262913000	4.014846000	10.303953000
6	131.960746000	4.142983000	9.165717000
6	133.245063000	4.941978000	9.093484000
6	133.189992000	6.086178000	10.126559000
6	135.482965000	7.113875000	9.734265000
6	136.691908000	7.638369000	10.519510000
6	137.715801000	8.323766000	9.599082000
1	131.289908000	4.363216000	12.490731000
1	130.376101000	3.388042000	10.338129000
1	133.371360000	5.857922000	13.511091000
1	132.778075000	7.359683000	12.805999000
1	131.669951000	3.631579000	8.257550000
1	134.095695000	4.279641000	9.326552000
1	132.564886000	6.882421000	9.689153000
1	137.182767000	9.102930000	9.035008000
1	138.475439000	8.804121000	10.225336000
1	136.853853000	9.259238000	11.888626000
1	134.159629000	6.050097000	7.761184000
1	137.746318000	7.061494000	8.124099000
1	138.872593000	4.933675000	6.719571000
1	133.605751000	9.152751000	3.611274000
1	128.652556000	3.769051000	4.754338000
1	133.905792000	-0.428887000	7.911718000
1	138.165869000	7.203581000	5.418028000
1	131.057265000	8.233815000	3.503113000
1	129.408067000	1.410827000	5.857389000
1	136.414088000	0.561416000	8.190226000

^{4}TS4$_{A}$:

16	133.709434000	3.747317000	3.776748000
1	132.564502000	3.029949000	3.842414000
7	136.090000000	3.217071000	5.603157000
7	134.774105000	5.712728000	5.820015000
7	133.635510000	1.864654000	6.256958000
6	135.792996000	0.788857000	5.782341000
6	137.132831000	5.403856000	5.229213000
6	132.609022000	6.787059000	6.259641000
6	131.254626000	2.161113000	6.765594000
6	136.552213000	1.929123000	5.554594000
6	136.010704000	6.190974000	5.460862000
6	131.852401000	5.644911000	6.491940000
6	132.379873000	1.378614000	6.543161000
8	134.799804000	3.892097000	7.896685000
6	137.960494000	1.915221000	5.224729000
6	135.977598000	7.628612000	5.351799000
6	130.447638000	5.660228000	6.822987000
6	132.399278000	-0.064124000	6.578355000
6	138.338858000	3.211755000	5.069932000
6	134.704454000	8.015336000	5.645713000
6	130.060265000	4.360946000	6.960961000
6	133.676709000	-0.445052000	6.302831000
6	137.162734000	4.018211000	5.306880000
6	133.958085000	6.811832000	5.928111000
6	131.230441000	3.551566000	6.719885000
6	134.439589000	0.764066000	6.097313000
7	132.311944000	4.350276000	6.444228000
26	134.189761000	3.776965000	5.989869000
1	136.298397000	-0.168521000	5.696948000
1	138.054794000	5.911574000	4.962958000
1	132.105126000	7.743603000	6.352299000
1	130.322404000	1.649822000	6.986120000
6	133.835752000	8.456307000	11.741377000
7	134.424707000	9.596363000	11.252189000
8	133.946830000	8.095028000	12.902798000
16	136.047990000	9.253590000	8.949230000
6	132.950475000	7.728575000	10.742494000
7	132.665756000	8.186415000	9.571343000
8	132.821267000	9.947657000	8.136434000
16	136.414641000	7.518202000	9.814200000
6	132.351764000	6.424625000	11.205969000
8	134.833578000	11.843453000	8.366728000
6	133.261585000	5.238812000	10.893412000
6	134.374501000	4.952704000	11.711570000
6	135.189274000	3.840269000	11.437583000
6	134.920683000	3.020261000	10.357426000
6	133.838932000	3.326436000	9.467112000
6	132.980717000	4.421963000	9.807954000
6	133.248455000	9.385547000	9.134560000
6	134.445229000	9.964646000	9.884544000
6	134.553376000	11.492477000	9.695382000
1	134.586440000	5.589261000	12.565432000
1	136.026477000	3.619071000	12.093941000
1	132.176314000	6.486504000	12.283777000
1	131.398550000	6.299557000	10.686706000
1	135.541134000	2.155668000	10.145228000
1	133.430975000	2.531374000	8.854439000
1	132.110021000	4.612036000	9.190278000
1	133.617097000	11.941572000	10.062749000
1	135.376242000	11.870875000	10.312264000
1	135.076693000	10.046242000	11.888450000
1	135.023278000	4.825014000	8.076204000
1	134.085438000	11.499644000	7.841680000
1	138.561363000	1.019897000	5.126620000
1	136.825044000	8.246766000	5.083912000
1	129.850999000	6.557656000	6.925423000
1	131.538772000	-0.687782000	6.785520000
1	139.315413000	3.605611000	4.818139000
1	134.289269000	9.014376000	5.670547000
1	129.078540000	3.970921000	7.199025000
1	134.081673000	-1.447066000	6.236614000

^{2}TS4$_{A}$:

16	133.855817000	3.825514000	3.722735000
1	132.719207000	3.092222000	3.743297000
7	136.203750000	3.336253000	5.591377000
7	134.795735000	5.776932000	5.849275000
7	133.784945000	1.881291000	6.152533000
6	135.988575000	0.894964000	5.695576000
6	137.176510000	5.567632000	5.304088000
6	132.584337000	6.762838000	6.264434000
6	131.384242000	2.080234000	6.613879000
6	136.711689000	2.066798000	5.516320000
6	136.022811000	6.307950000	5.533749000
6	131.862972000	5.588842000	6.443162000
6	132.541591000	1.344136000	6.398098000
8	134.839535000	3.896760000	7.872256000
6	138.126061000	2.111008000	5.216563000
6	135.942368000	7.746557000	5.468097000
6	130.451251000	5.546047000	6.740664000
6	132.611411000	-0.097412000	6.395453000
6	138.461917000	3.423787000	5.106962000
6	134.650318000	8.080404000	5.742615000
6	130.107419000	4.230747000	6.836035000
6	133.906957000	-0.425877000	6.137407000
6	137.253570000	4.181969000	5.341436000
6	133.939784000	6.843850000	5.969865000
6	131.310962000	3.469739000	6.601877000
6	134.630629000	0.814306000	5.980975000
7	132.369212000	4.312836000	6.371715000
26	134.276062000	3.817265000	5.947915000
1	136.528935000	-0.041533000	5.594277000
1	138.085441000	6.114799000	5.073534000
1	132.045484000	7.698419000	6.372723000
1	130.466355000	1.530762000	6.800026000
6	133.631016000	8.358391000	11.822523000
7	134.193196000	9.533792000	11.387956000
8	133.711097000	7.968850000	12.977233000
16	135.921353000	9.320140000	9.144395000
6	132.814905000	7.625448000	10.769821000
7	132.562554000	8.102900000	9.599211000
8	132.706504000	9.906398000	8.216981000
16	136.344715000	7.596061000	10.004543000
6	132.244237000	6.289953000	11.175298000
8	134.629312000	11.873411000	8.581580000
6	133.198553000	5.141292000	10.857304000
6	134.303032000	4.873880000	11.693101000
6	135.156623000	3.791655000	11.416571000
6	134.934417000	2.983168000	10.317329000
6	133.862691000	3.273444000	9.409716000
6	132.965176000	4.336795000	9.751535000
6	133.114369000	9.335250000	9.218006000
6	134.253450000	9.941521000	10.033166000
6	134.307495000	11.476780000	9.887736000
1	134.477554000	5.499799000	12.563164000
1	135.986672000	3.583663000	12.086193000
1	132.033254000	6.313636000	12.248227000
1	131.312002000	6.151551000	10.622409000
1	135.584419000	2.141029000	10.103348000
1	133.491250000	2.477832000	8.774775000
1	132.102374000	4.512863000	9.118746000
1	133.339179000	11.878953000	10.225197000
1	135.087562000	11.870416000	10.549093000
1	134.799809000	9.990918000	12.062782000
1	135.032999000	4.830953000	8.077173000
1	133.918059000	11.515457000	8.016300000
1	138.759871000	1.240182000	5.105974000
1	136.773913000	8.401045000	5.239524000
1	129.820485000	6.418832000	6.851662000
1	131.769348000	-0.756099000	6.566923000
1	139.429047000	3.858266000	4.887685000
1	134.199931000	9.063345000	5.788771000
1	129.135096000	3.800307000	7.040755000
1	134.348464000	-1.410949000	6.052874000

^{4}**IM4$_{A}$:**

16	133.696360000	3.669413000	3.733301000
1	132.498347000	3.047580000	3.822996000
7	136.072282000	3.186693000	5.568360000
7	134.772885000	5.680419000	5.789629000
7	133.618870000	1.848882000	6.243702000
6	135.764258000	0.757361000	5.746946000
6	137.133951000	5.366293000	5.202739000
6	132.609414000	6.772412000	6.209667000
6	131.243936000	2.157633000	6.776771000
6	136.524923000	1.893829000	5.507432000
6	136.015337000	6.155781000	5.437471000
6	131.846038000	5.634275000	6.438557000
6	132.366686000	1.370931000	6.555318000
8	134.791686000	3.901584000	8.028505000
6	137.929066000	1.870942000	5.164883000
6	135.990382000	7.593274000	5.337040000
6	130.442764000	5.656672000	6.775605000
6	132.381896000	-0.070757000	6.609343000
6	138.317506000	3.165535000	5.015304000
6	134.716944000	7.985215000	5.622833000
6	130.054852000	4.360554000	6.937221000
6	133.652985000	-0.460218000	6.315945000
6	137.151011000	3.979837000	5.266387000
6	133.961244000	6.785949000	5.893555000
6	131.221586000	3.545457000	6.702016000
6	134.416548000	0.742616000	6.082672000
7	132.304045000	4.338526000	6.404829000
26	134.170583000	3.755134000	5.918264000
1	136.263784000	-0.202713000	5.658558000
1	138.058853000	5.870069000	4.939631000
1	132.110654000	7.732276000	6.296599000
1	130.313891000	1.651521000	7.017050000
6	133.777912000	8.465904000	11.790335000
7	134.383159000	9.597043000	11.297360000
8	133.873949000	8.112097000	12.954786000
16	135.998406000	9.214309000	8.989152000
6	132.899120000	7.738069000	10.786452000
7	132.617615000	8.197630000	9.614917000
8	132.780814000	9.962712000	8.184621000
16	136.360511000	7.496690000	9.890536000
6	132.321592000	6.420502000	11.232201000
8	134.828187000	11.821835000	8.400100000
6	133.275909000	5.272613000	10.901327000
6	134.293996000	4.905158000	11.823729000
6	135.191985000	3.854546000	11.506872000
6	135.112750000	3.183946000	10.317063000
6	134.095998000	3.536298000	9.274901000
6	133.156833000	4.622069000	9.700800000
6	133.205058000	9.394405000	9.180392000
6	134.410062000	9.959239000	9.928946000
6	134.544304000	11.483483000	9.731444000
1	134.384851000	5.441068000	12.762939000
1	135.958398000	3.584306000	12.229125000
1	132.137915000	6.458588000	12.309219000
1	131.376862000	6.277603000	10.702538000
1	135.808983000	2.387818000	10.069587000
1	133.538463000	2.646014000	8.956441000
1	132.379915000	4.898455000	8.996916000
1	133.616417000	11.950742000	10.097390000
1	135.374543000	11.851344000	10.344812000
1	135.035436000	10.044830000	11.934735000
1	135.198530000	4.780367000	8.135647000
1	134.071918000	11.489821000	7.878972000
1	138.521915000	0.971321000	5.057408000
1	136.843126000	8.208132000	5.078459000
1	129.848945000	6.556770000	6.871003000
1	131.522875000	-0.688295000	6.839795000
1	139.295591000	3.552712000	4.759063000
1	134.307206000	8.986605000	5.648837000
1	129.074850000	3.975924000	7.190573000
1	134.053363000	-1.464480000	6.255978000

^{4}**Re$_{A,B3LYP-D3}$:**

16	133.438110000	3.615843000	3.852941000
1	132.965584000	4.709745000	3.215902000
7	135.303850000	4.425285000	6.381413000
7	133.550554000	6.416362000	5.346621000
7	133.103923000	2.644051000	6.668814000
6	135.443887000	2.111872000	7.171234000
6	135.975031000	6.653288000	5.615854000
6	131.224655000	6.924770000	4.768110000
6	130.673004000	2.420589000	6.436821000
6	135.998387000	3.343147000	6.855651000
6	134.726576000	7.115989000	5.233456000
6	130.661024000	5.713270000	5.131945000
6	131.930647000	1.938373000	6.774364000
8	133.040938000	5.152152000	7.660289000
6	137.406493000	3.646078000	6.939196000
6	134.485119000	8.411370000	4.647759000
6	129.258137000	5.395950000	5.025823000
6	132.189726000	0.607939000	7.262352000
6	137.555428000	4.924315000	6.496753000
6	133.147785000	8.492650000	4.416168000
6	129.103394000	4.130893000	5.501386000
6	133.536798000	0.511771000	7.442695000
6	136.237032000	5.395190000	6.147784000
6	132.575556000	7.244816000	4.859026000
6	130.411578000	3.667952000	5.894586000
6	134.096646000	1.788547000	7.078867000
7	131.352891000	4.641220000	5.650844000
26	133.302175000	4.582680000	6.146894000
1	136.117342000	1.339951000	7.529332000
1	136.816358000	7.326171000	5.492895000
1	130.559645000	7.684171000	4.368672000
1	129.828860000	1.753598000	6.581769000
6	135.030431000	7.642442000	11.322442000
7	135.583836000	8.787103000	10.791046000
8	134.911870000	7.446665000	12.519277000
16	137.132083000	7.617178000	8.775830000
6	134.620573000	6.639024000	10.231658000
7	134.002895000	7.319826000	9.125876000
8	134.174016000	9.030945000	7.610378000
16	136.326441000	5.885745000	9.725795000
6	133.698300000	5.525057000	10.774368000
8	136.585881000	10.361733000	7.596457000
6	134.386768000	4.240508000	11.182374000
6	135.082651000	4.136824000	12.395540000
6	135.705610000	2.938977000	12.747239000
6	135.637407000	1.833127000	11.894963000
6	134.946057000	1.931512000	10.685656000
6	134.325569000	3.129925000	10.329926000
6	134.575494000	8.441177000	8.612933000
6	135.826131000	8.877810000	9.383674000
6	136.284237000	10.283568000	8.966767000
1	135.136684000	5.003889000	13.047162000
1	136.243553000	2.867956000	13.688999000
1	133.165920000	5.955148000	11.628297000
1	132.970069000	5.298748000	9.994126000
1	136.119193000	0.899510000	12.174428000
1	134.880178000	1.076444000	10.018356000
1	133.794777000	3.217658000	9.386572000
1	135.480690000	10.978303000	9.261406000
1	137.191083000	10.547403000	9.521475000
1	136.133162000	9.352867000	11.429904000
1	133.464808000	6.728632000	8.475428000
1	135.756734000	10.094534000	7.149528000
1	138.163493000	2.958149000	7.292240000
1	135.252654000	9.150996000	4.460866000
1	128.504892000	6.069286000	4.637619000
1	131.430146000	-0.144698000	7.429095000
1	138.459077000	5.512918000	6.410717000
1	132.581760000	9.313312000	3.995341000
1	128.196090000	3.546634000	5.584259000
1	134.113494000	-0.335667000	7.790268000

^{4}IM1$_{A,B3LYP\text{-}D3}$:

16	133.048162000	4.728963000	3.627041000
1	131.760476000	4.721971000	4.036806000
7	135.727174000	4.233831000	4.664027000
7	134.315647000	6.394145000	5.882555000
7	133.707989000	2.416223000	5.539872000
6	135.698230000	1.814257000	4.240748000
6	136.498928000	6.562920000	4.769564000
6	132.167530000	7.047115000	6.879459000
6	131.569653000	2.238664000	6.726947000
6	136.259289000	3.080185000	4.150290000
6	135.398403000	7.100474000	5.417864000
6	131.622328000	5.776338000	6.995936000
6	132.658528000	1.704750000	6.052824000
8	134.844406000	4.045589000	7.229674000
6	137.535093000	3.359612000	3.537989000
6	135.193449000	8.510577000	5.629810000
6	130.359085000	5.492772000	7.630551000
6	132.817638000	0.302246000	5.744421000
6	137.772613000	4.689164000	3.709090000
6	133.961909000	8.649867000	6.197862000
6	130.203735000	4.139074000	7.611542000
6	133.966234000	0.186002000	5.024657000
6	136.637173000	5.229342000	4.416017000
6	133.416343000	7.323687000	6.344932000
6	131.364136000	3.590724000	6.952845000
6	134.518670000	1.514923000	4.902136000
7	132.211326000	4.607017000	6.579268000
26	134.013554000	4.396783000	5.699569000
1	136.238133000	0.993512000	3.779915000
1	137.295352000	7.242697000	4.485863000
1	131.575686000	7.880017000	7.243485000
1	130.808478000	1.549861000	7.078469000
6	133.027957000	7.444853000	10.035169000
7	133.790042000	8.589567000	9.983880000
8	131.844607000	7.446423000	10.335871000
16	136.185271000	8.411385000	11.507467000
6	133.761070000	6.189621000	9.598783000
7	134.936913000	6.209873000	9.060640000
8	136.664209000	7.474203000	8.281602000
16	135.058599000	7.216054000	12.603786000
6	133.064654000	4.882768000	9.836010000
8	137.091249000	10.044259000	9.241864000
6	133.361905000	4.445098000	11.264276000
6	132.486645000	4.772338000	12.305388000
6	132.804382000	4.435186000	13.622484000
6	134.001318000	3.773290000	13.908269000
6	134.878412000	3.446083000	12.870899000
6	134.561856000	3.783968000	11.554250000
6	135.650722000	7.408278000	8.960731000
6	135.192743000	8.602487000	9.790610000
6	135.691178000	9.937967000	9.207630000
1	131.572597000	5.314939000	12.081108000
1	132.119639000	4.693988000	14.425764000
1	131.990887000	5.013977000	9.697275000
1	133.441746000	4.157767000	9.110484000
1	134.250653000	3.516156000	14.934143000
1	135.811526000	2.932928000	13.086874000
1	135.247527000	3.545596000	10.745108000
1	135.286539000	10.015566000	8.187907000
1	135.286766000	10.761902000	9.806876000
1	133.346253000	9.413637000	10.377494000
1	135.118851000	4.862954000	7.709371000
1	137.422403000	9.287589000	8.720182000
1	138.159184000	2.617447000	3.057094000
1	135.905469000	9.279787000	5.360175000
1	129.710411000	6.244450000	8.060559000
1	132.122713000	-0.473040000	6.040638000
1	138.630781000	5.269159000	3.394941000
1	133.445126000	9.557393000	6.483158000
1	129.386741000	3.547899000	8.004927000
1	134.416950000	-0.705928000	4.608786000

^{4}Re$_{A,PBE0}$:

16	133.235098000	3.657885000	3.665351000
1	132.441977000	4.503382000	2.976164000
7	135.242170000	5.299897000	5.604669000
7	132.876257000	6.596041000	4.758455000
7	133.709871000	3.133793000	6.563943000
6	136.135644000	3.345650000	6.759241000
6	135.145913000	7.420960000	4.403055000
6	130.439730000	6.461715000	4.719926000
6	131.452694000	2.217025000	6.754414000
6	136.269069000	4.567155000	6.119815000
6	133.769771000	7.523239000	4.306025000
6	130.312235000	5.198353000	5.262377000
6	132.825782000	2.158398000	6.927357000
8	133.171284000	5.675008000	7.215924000
6	137.531076000	5.206266000	5.863906000
6	133.085604000	8.642599000	3.720096000
6	129.059563000	4.523166000	5.456926000
6	133.511912000	1.054422000	7.539135000
6	137.252696000	6.337273000	5.168502000
6	131.757376000	8.385705000	3.834256000
6	129.346949000	3.322166000	6.018615000
6	134.830753000	1.378830000	7.554256000
6	135.823950000	6.385848000	5.024554000
6	131.644306000	7.105140000	4.476436000
6	130.773650000	3.267678000	6.172159000
6	134.939925000	2.674501000	6.944411000
7	131.353281000	4.413886000	5.689612000
26	133.287467000	4.927759000	5.779596000
1	137.039808000	2.870400000	7.125751000
1	135.739107000	8.232606000	3.994227000
1	129.528578000	6.980212000	4.437604000
1	130.867021000	1.372312000	7.103445000
6	135.268002000	7.269869000	11.418753000
7	136.361903000	8.023787000	11.088597000
8	134.663367000	7.383287000	12.465861000
16	137.898468000	6.130135000	9.760554000
6	134.926728000	6.266685000	10.311574000
7	134.969948000	6.922250000	9.040221000
8	136.199168000	8.251159000	7.636511000
16	136.310333000	4.979830000	10.513128000
6	133.574306000	5.577554000	10.527696000
8	138.804603000	8.631916000	8.420214000
6	133.584118000	4.362710000	11.425630000
6	133.532143000	4.472225000	12.819607000
6	133.520500000	3.331036000	13.615064000
6	133.562708000	2.065170000	13.033329000
6	133.613098000	1.946408000	11.647790000
6	133.618111000	3.087969000	10.850685000
6	136.028071000	7.701272000	8.720094000
6	137.039990000	7.803664000	9.858881000
6	138.077217000	8.890217000	9.580023000
1	133.516165000	5.459729000	13.269973000
1	133.479276000	3.430993000	14.696619000
1	132.899354000	6.341651000	10.928955000
1	133.200700000	5.287275000	9.539989000
1	133.554073000	1.176144000	13.658741000
1	133.643476000	0.964928000	11.181320000
1	133.648545000	2.991117000	9.768302000
1	137.531812000	9.847159000	9.533748000
1	138.784337000	8.934538000	10.415827000
1	136.820765000	8.522129000	11.841809000
1	134.357210000	6.582512000	8.284572000
1	138.125727000	8.575139000	7.720034000
1	138.491320000	4.826659000	6.187069000
1	133.574680000	9.506360000	3.288453000
1	128.092995000	4.932439000	5.192175000
1	133.030255000	0.156171000	7.903898000
1	137.935475000	7.089576000	4.795659000
1	130.919346000	8.990466000	3.512063000
1	128.666927000	2.534083000	6.315611000
1	135.666101000	0.805693000	7.935269000

^{4}Re$_{A,M06L}$:

16	133.551126000	3.715272000	3.863636000
1	133.007648000	4.785419000	3.248528000
7	135.322381000	4.402756000	6.407381000
7	133.586609000	6.438548000	5.403621000
7	133.107498000	2.641071000	6.647560000
6	135.429136000	2.092788000	7.184047000
6	136.014400000	6.610932000	5.624602000
6	131.268824000	6.991041000	4.870920000
6	130.687810000	2.440166000	6.358379000
6	136.003542000	3.316342000	6.884872000
6	134.773907000	7.103127000	5.252708000
6	130.687956000	5.779897000	5.208639000
6	131.932816000	1.937427000	6.703478000
8	133.064590000	5.126212000	7.711315000
6	137.408559000	3.603059000	6.972774000
6	134.555539000	8.393203000	4.661757000
6	129.288406000	5.479283000	5.089193000
6	132.173850000	0.600532000	7.164483000
6	137.572796000	4.874333000	6.521068000
6	133.215888000	8.511632000	4.469643000
6	129.127557000	4.194457000	5.500417000
6	133.512458000	0.498456000	7.384208000
6	136.265763000	5.353573000	6.164220000
6	132.625029000	7.288763000	4.935455000
6	130.428438000	3.711491000	5.871308000
6	134.081860000	1.775878000	7.063105000
7	131.370563000	4.686687000	5.679762000
26	133.321266000	4.583497000	6.160637000
1	136.093095000	1.306325000	7.539229000
1	136.872251000	7.262951000	5.470174000
1	130.608078000	7.769515000	4.493556000
1	129.837437000	1.768952000	6.464531000
6	135.009750000	7.624035000	11.323328000
7	135.559813000	8.758080000	10.766401000
8	134.933797000	7.429641000	12.522003000
16	136.970046000	7.554311000	8.680061000
6	134.543370000	6.634909000	10.254810000
7	133.890173000	7.311716000	9.182450000
8	134.018539000	9.022642000	7.661294000
16	136.232218000	5.880438000	9.691451000
6	133.661270000	5.517800000	10.822203000
8	136.498269000	10.221301000	7.524319000
6	134.377758000	4.252967000	11.206488000
6	135.068104000	4.142681000	12.418417000
6	135.737472000	2.965795000	12.738880000
6	135.722777000	1.887427000	11.856730000
6	135.035132000	1.990450000	10.649649000
6	134.365285000	3.166821000	10.325417000
6	134.451976000	8.425968000	8.642671000
6	135.731125000	8.843675000	9.358336000
6	136.212687000	10.210342000	8.892373000
1	135.080572000	4.992675000	13.099505000
1	136.271572000	2.888611000	13.684635000
1	133.146247000	5.945484000	11.691618000
1	132.911037000	5.281703000	10.060594000
1	136.244166000	0.966088000	12.112373000
1	135.006920000	1.147379000	9.959319000
1	133.828117000	3.263714000	9.379555000
1	135.431789000	10.941124000	9.167853000
1	137.134449000	10.479654000	9.422819000
1	136.150362000	9.314709000	11.375662000
1	133.333498000	6.712823000	8.551334000
1	135.647187000	9.993972000	7.098267000
1	138.158435000	2.906131000	7.330299000
1	135.341148000	9.108001000	4.438918000
1	128.538806000	6.175026000	4.728413000
1	131.407099000	-0.155505000	7.291693000
1	138.486647000	5.451914000	6.428546000
1	132.661175000	9.345256000	4.053197000
1	128.216493000	3.608326000	5.549933000
1	134.079477000	-0.360499000	7.727482000

^{4}IM1$_{A,M06L}$:

16	133.059222000	4.774673000	3.649344000
1	131.779412000	4.897619000	4.057954000
7	135.715575000	4.184905000	4.728771000
7	134.330690000	6.375171000	5.935013000
7	133.650646000	2.408255000	5.564959000
6	135.660757000	1.770390000	4.326792000
6	136.511715000	6.501368000	4.831774000
6	132.190554000	7.056171000	6.913422000
6	131.472276000	2.280260000	6.669772000
6	136.246088000	3.024314000	4.237306000
6	135.418251000	7.064904000	5.469060000
6	131.611045000	5.799160000	7.012216000
6	132.566504000	1.719732000	6.029624000
8	134.777929000	4.027039000	7.298335000
6	137.530389000	3.281684000	3.650528000
6	135.237418000	8.472093000	5.677118000
6	130.334505000	5.540304000	7.615759000
6	132.700857000	0.322743000	5.719157000
6	137.778144000	4.609100000	3.811434000
6	134.011195000	8.630035000	6.248336000
6	130.140286000	4.194668000	7.561724000
6	133.872954000	0.181274000	5.046057000
6	136.641337000	5.165501000	4.488755000
6	133.446970000	7.318175000	6.389812000
6	131.292470000	3.631206000	6.916393000
6	134.458621000	1.489928000	4.952518000
7	132.174301000	4.626280000	6.580329000
26	133.987050000	4.377136000	5.729565000
1	136.199464000	0.936503000	3.881998000
1	137.323199000	7.168384000	4.549259000
1	131.608414000	7.902120000	7.274083000
1	130.680190000	1.606070000	6.988480000
6	132.976844000	7.554423000	9.989360000
7	133.749024000	8.691532000	9.870858000
8	131.792642000	7.583634000	10.284178000
16	136.061463000	8.475676000	11.509542000
6	133.705200000	6.288405000	9.619272000
7	134.893248000	6.267244000	9.094915000
8	136.674239000	7.479122000	8.350486000
16	134.854332000	7.333658000	12.537542000
6	132.993506000	5.003421000	9.877018000
8	137.114732000	10.027809000	9.335200000
6	133.408358000	4.470942000	11.232697000
6	132.576023000	4.617967000	12.343332000
6	132.990787000	4.170579000	13.595521000
6	134.243601000	3.580847000	13.748392000
6	135.080222000	3.437379000	12.643647000
6	134.667018000	3.883029000	11.392201000
6	135.623568000	7.446870000	8.978252000
6	135.149286000	8.667124000	9.736654000
6	135.728127000	9.965189000	9.183386000
1	131.603620000	5.095436000	12.223358000
1	132.332846000	4.286197000	14.455346000
1	131.913736000	5.178482000	9.850490000
1	133.274941000	4.296994000	9.084140000
1	134.568585000	3.235236000	14.728099000
1	136.060535000	2.977966000	12.756825000
1	135.319377000	3.777325000	10.525217000
1	135.412687000	10.043995000	8.127964000
1	135.303056000	10.817431000	9.729995000
1	133.312269000	9.539048000	10.221879000
1	135.080060000	4.853495000	7.745077000
1	137.458419000	9.263286000	8.833876000
1	138.157430000	2.526267000	3.189414000
1	135.964366000	9.231273000	5.407160000
1	129.696429000	6.303360000	8.048615000
1	131.973604000	-0.437965000	5.982082000
1	138.650562000	5.177808000	3.508943000
1	133.505575000	9.547795000	6.533163000
1	129.296327000	3.617388000	7.924003000
1	134.316770000	-0.721668000	4.640758000

Model G structures:

$^{4}Re_{G}$:

6	-14.681272000	38.588600000	97.431108000
6	-14.255748000	39.956668000	96.898059000
8	-14.951373000	40.672098000	96.187759000
6	-16.155227000	38.470538000	97.890505000
6	-17.162226000	38.427303000	96.720901000
6	-16.507510000	39.562258000	98.913767000
6	-17.110898000	37.140396000	95.888440000
1	-14.489033000	37.863320000	96.628791000
1	-16.217685000	37.500015000	98.406749000
1	-18.171690000	38.524781000	97.141048000
1	-17.005481000	39.301745000	96.082877000
1	-15.808285000	39.555720000	99.761565000
1	-16.483422000	40.556842000	98.456501000
1	-17.517635000	39.409117000	99.309491000
1	-16.126094000	36.973987000	95.433610000
1	-17.359764000	36.262779000	96.497101000
1	-17.836341000	37.187193000	95.068508000
7	-12.959292000	40.311349000	97.228200000
6	-12.354748000	41.583874000	96.853032000
6	-11.173314000	41.419255000	95.866780000
8	-10.368046000	42.332280000	95.682622000
6	-11.918082000	42.404116000	98.084253000
6	-13.086461000	43.020784000	98.866418000
6	-12.648761000	43.584251000	100.229278000
6	-13.777185000	44.253739000	101.024866000
7	-13.975425000	45.697369000	100.689581000
1	-12.474116000	39.751917000	97.917883000
1	-13.134146000	42.126737000	96.303852000
1	-11.232769000	43.184720000	97.741624000
1	-11.329485000	41.752534000	98.748618000
1	-13.552984000	43.809492000	98.259795000
1	-13.868469000	42.268218000	99.032197000
1	-11.812674000	44.287728000	100.107511000
1	-12.257152000	42.760364000	100.839541000
1	-13.579153000	44.218259000	102.099545000
1	-14.738238000	43.768135000	100.838176000
1	-14.839605000	46.071503000	101.158216000
1	-13.237978000	46.325291000	101.055389000
1	-14.055485000	45.916819000	99.675034000
7	-11.110624000	40.242577000	95.188819000
6	-9.959499000	39.886953000	94.363272000
6	-9.798861000	40.620742000	93.018383000
8	-8.731800000	40.550723000	92.428501000
1	-11.780070000	39.528882000	95.441480000
1	-9.033096000	40.081111000	94.911803000
7	-10.882669000	41.310545000	92.570307000
6	-10.846288000	42.161538000	91.383381000
6	-10.300337000	43.575886000	91.675325000
8	-10.121163000	44.368677000	90.747813000
6	-12.286186000	42.269221000	90.799133000
6	-12.375480000	42.659350000	89.353915000
6	-12.466193000	43.909001000	88.786454000
7	-12.383517000	41.684960000	88.372361000
6	-12.469030000	42.333180000	87.232127000
7	-12.525113000	43.684876000	87.427524000
1	-11.727558000	41.294025000	93.125782000
1	-10.183116000	41.690846000	90.654327000
1	-12.743018000	41.277705000	90.894728000
1	-12.863391000	42.965171000	91.421442000
1	-12.441695000	44.903781000	89.208136000
1	-12.503187000	41.886372000	86.247424000
1	-12.573700000	44.395321000	86.711987000
7	-10.123329000	43.890753000	92.979764000
6	-9.877077000	45.244566000	93.441452000
6	-10.803997000	45.612912000	94.610098000
6	-12.274353000	45.476011000	94.308076000
6	-13.303645000	45.203472000	95.183348000
7	-12.807037000	45.698131000	93.049121000
6	-14.118005000	45.566519000	93.177713000
7	-14.468853000	45.259194000	94.447244000
1	-10.212330000	43.170126000	93.686919000
1	-10.037895000	45.914447000	92.595966000
1	-10.562021000	44.989158000	95.478653000
1	-10.573847000	46.652098000	94.881692000
1	-13.308851000	44.994371000	96.242151000
1	-14.844802000	45.683605000	92.386200000
6	-16.199161000	50.012952000	92.336481000
6	-14.934797000	49.461046000	92.969766000
8	-14.981534000	48.838266000	94.044081000
6	-17.219192000	48.902016000	91.997802000
6	-18.615470000	49.478069000	91.734291000
16	-19.888531000	48.228420000	91.316877000
6	-20.092251000	47.374841000	92.923806000
1	-16.640866000	50.690943000	93.079030000
1	-17.261150000	48.193657000	92.829464000
1	-16.881646000	48.341217000	91.118505000
1	-18.959352000	50.058777000	92.600091000
1	-18.598321000	50.159315000	90.875479000
1	-20.871017000	46.623203000	92.770489000
1	-19.173761000	46.872252000	93.238814000
1	-20.431777000	48.060734000	93.706246000
7	-13.757072000	49.707574000	92.333437000
6	-12.506390000	49.075909000	92.750313000
6	-11.766227000	49.766371000	93.895598000
8	-10.930687000	49.139204000	94.552655000
1	-13.805220000	50.080825000	91.394477000
1	-11.842342000	48.971718000	91.885985000
1	-12.703706000	48.052494000	93.075095000
7	-12.089308000	51.056222000	94.170113000
6	-11.455233000	51.805239000	95.239897000
6	-12.325788000	52.106021000	96.467591000
8	-12.050373000	53.074551000	97.181022000
1	-12.799242000	51.501284000	93.604128000
1	-10.586889000	51.222311000	95.562742000
7	-13.380725000	51.279468000	96.707392000
6	-14.264091000	51.570634000	97.839187000
6	-13.665741000	50.943314000	99.113707000
8	-13.906913000	49.790766000	99.503617000
6	-15.741548000	51.253500000	97.532566000
6	-16.209270000	49.781915000	97.528998000
6	-16.877156000	49.319652000	98.849444000
6	-18.391300000	49.229796000	98.678734000
7	-19.115032000	48.816553000	99.924020000
1	-13.390551000	50.333170000	96.314670000
1	-14.203188000	52.654441000	97.965776000
1	-16.353447000	51.826626000	98.240448000
1	-15.922803000	51.705766000	96.550492000
1	-15.379796000	49.115982000	97.289710000
1	-16.927267000	49.657114000	96.707905000
1	-16.486088000	48.342447000	99.146403000
1	-16.625564000	50.006107000	99.665915000
1	-18.640820000	48.491639000	97.912386000
1	-18.827435000	50.187727000	98.381590000
1	-18.677063000	48.016201000	100.418822000
1	-19.145660000	49.585634000	100.620671000
1	-20.124966000	48.626038000	99.645321000
7	-12.800762000	51.763784000	99.756262000
6	-12.123403000	51.371330000	100.969186000
6	-13.083741000	51.314518000	102.171110000
8	-14.293837000	51.464512000	102.047235000
1	-12.503711000	52.596710000	99.252711000
1	-11.678213000	50.373533000	100.862887000
7	-12.487553000	51.081481000	103.382164000
6	-13.260280000	50.446880000	104.455422000
6	-14.476451000	51.239127000	104.938004000
8	-15.484541000	50.646266000	105.323354000
1	-11.512029000	50.807825000	103.348000000
1	-13.624926000	49.467604000	104.133744000
7	-14.367323000	52.589925000	104.950757000
6	-15.460909000	53.438162000	105.403941000
6	-16.749165000	53.401477000	104.561877000
8	-17.772165000	53.900455000	105.005830000
1	-13.528904000	53.008393000	104.571789000
1	-15.758599000	53.156808000	106.417745000
7	-16.663336000	52.788334000	103.344321000
6	-17.821939000	52.622263000	102.492905000
6	-18.421415000	51.203110000	102.563869000

8	-19.369923000	50.895890000	101.803059000
6	-17.505914000	52.987845000	101.020273000
6	-17.060496000	54.422173000	100.836563000
6	-15.702545000	54.738386000	100.697158000
6	-17.996765000	55.464669000	100.807839000
6	-15.289507000	56.062256000	100.531055000
6	-17.588193000	56.788808000	100.647022000
6	-16.231751000	57.091679000	100.506569000
1	-15.775004000	52.417381000	103.018527000
1	-18.592848000	53.296478000	102.883010000
1	-16.719997000	52.309830000	100.664286000
1	-18.401936000	52.782453000	100.426120000
1	-14.966900000	53.937011000	100.721658000
1	-19.056091000	55.238480000	100.911622000
1	-14.232639000	56.290177000	100.417778000
1	-18.328526000	57.583807000	100.627172000
1	-15.913127000	58.122104000	100.376564000
7	-17.888525000	50.351073000	103.444283000
6	-18.413475000	49.017202000	103.749334000
6	-17.304995000	47.959895000	103.822845000
6	-16.866579000	47.326073000	102.495262000
8	-15.637910000	47.063055000	102.358773000
8	-17.766777000	47.010436000	101.654870000
1	-17.142432000	50.691847000	104.049794000
1	-19.155597000	48.755063000	102.995186000
1	-16.433369000	48.357610000	104.349731000
1	-17.685183000	47.124357000	104.426873000
6	-24.315271000	35.218007000	98.176433000
6	-23.049187000	34.436472000	98.462821000
8	-22.718754000	34.088528000	99.602238000
1	-24.087298000	36.166139000	97.677129000
7	-22.263633000	34.120534000	97.391888000
6	-21.087198000	33.271948000	97.601779000
6	-20.009559000	34.017703000	98.405834000
8	-19.477382000	35.047713000	97.964167000
6	-20.613194000	32.952569000	96.164073000
6	-21.142151000	34.128962000	95.325717000
6	-22.494345000	34.454766000	95.974077000
1	-21.379424000	32.366713000	98.145799000
1	-21.073688000	32.011779000	95.841586000
1	-19.528157000	32.834907000	96.099600000
1	-21.239160000	33.884286000	94.263870000
1	-20.469528000	34.985228000	95.426841000
1	-22.775698000	35.505010000	95.856440000
1	-23.294721000	33.823746000	95.559470000
7	-19.650572000	33.447887000	99.578970000
6	-18.659263000	34.029928000	100.477662000
6	-19.252681000	34.860938000	101.631610000
6	-20.108942000	36.070710000	101.223216000
6	-19.327404000	37.152507000	100.456949000
7	-20.122510000	38.342888000	100.140572000
6	-21.054574000	38.402653000	99.148312000
7	-21.151746000	37.462731000	98.221052000
7	-21.894973000	39.453754000	99.137040000
1	-20.297729000	32.771024000	99.963881000
1	-17.994455000	34.631846000	99.854505000
1	-19.865123000	34.203047000	102.261683000
1	-18.412984000	35.189454000	102.261412000
1	-20.526188000	36.519115000	102.135020000
1	-20.965115000	35.729636000	100.630169000
1	-18.472491000	37.496600000	101.050573000
1	-18.924924000	36.752533000	99.523383000
1	-20.315697000	38.963430000	100.915829000
1	-20.565789000	36.629678000	98.220389000
1	-21.814227000	37.611163000	97.417634000
1	-21.522306000	40.341739000	99.461626000
1	-22.564912000	39.531949000	98.338967000
16	-26.725637000	46.040506000	95.035621000
8	-22.626818000	45.505078000	95.476896000
26	-24.271310000	45.613613000	95.322210000
6	-24.936849000	42.596928000	96.798739000
7	-24.563519000	43.727025000	94.662627000
6	-24.317974000	44.110706000	92.255327000
7	-24.192491000	46.264283000	93.415513000
8	-23.644404000	39.761902000	97.001424000

6	-24.104889000	48.683976000	93.799407000
7	-24.392945000	47.533316000	95.946733000
8	-22.890429000	37.811571000	96.160135000
6	-24.534333000	47.156146000	98.366038000
7	-24.622822000	44.999841000	97.204962000
6	-24.873421000	42.618443000	95.412258000
6	-25.107247000	41.460911000	94.560049000
6	-24.887383000	41.906585000	93.287615000
6	-24.559577000	43.303629000	93.363872000
6	-25.598966000	40.098218000	94.973061000
6	-24.566352000	38.954580000	94.923469000
6	-23.627929000	38.844645000	96.134240000
6	-24.136940000	45.484808000	92.284488000
6	-23.928568000	46.308373000	91.119186000
6	-23.871254000	47.596541000	91.554932000
6	-24.046827000	47.558494000	92.985893000
6	-24.303228000	48.671228000	95.169738000
6	-24.393929000	49.844675000	95.997965000
6	-24.507327000	49.414834000	97.284404000
6	-24.492417000	47.975734000	97.248984000
6	-24.590899000	45.766447000	98.342013000
6	-24.713901000	44.933539000	99.511118000
6	-24.840027000	43.652001000	99.065892000
6	-24.787624000	43.705342000	97.626811000
1	-25.059223000	41.628251000	97.269081000
1	-24.302835000	43.626850000	91.283625000
1	-24.004375000	49.651467000	93.317848000
1	-24.538185000	47.640676000	99.336493000
1	-26.031640000	40.139504000	95.976464000
1	-26.421959000	39.838195000	94.294749000
1	-25.091741000	37.995848000	94.837225000
1	-23.939123000	39.033528000	94.026826000
6	-19.861976000	42.837048000	98.453695000
7	-18.961738000	43.750836000	98.924939000
8	-20.442660000	42.034390000	99.170396000
16	-16.981987000	43.422624000	97.030416000
6	-20.013508000	42.938748000	96.918155000
7	-20.036299000	44.342384000	96.535507000
8	-18.973168000	46.371701000	96.656692000
16	-18.448146000	42.092988000	96.234165000
6	-19.092024000	45.178848000	96.993521000
8	-16.897906000	45.045145000	100.026143000
6	-18.163883000	44.520403000	98.015980000
6	-21.232156000	42.172783000	96.370913000
6	-21.094166000	41.682875000	94.939757000
6	-21.181191000	42.560014000	93.848394000
6	-21.011265000	42.091348000	92.545917000
6	-20.761833000	40.736693000	92.310857000
6	-20.695773000	39.852104000	93.387505000
6	-20.860650000	40.322377000	94.692758000
6	-17.337272000	45.564647000	98.788686000
1	-18.579362000	43.643016000	99.861050000
1	-20.793433000	44.697750000	95.940023000
1	-17.194572000	45.703644000	100.714608000
1	-21.407349000	43.609872000	94.012209000
1	-21.078333000	42.785820000	91.712472000
1	-20.629147000	40.373708000	91.295133000
1	-20.521463000	38.793200000	93.218019000
1	-20.830588000	39.620346000	95.520597000
1	-21.414956000	41.326218000	97.032983000
1	-22.094565000	42.840425000	96.474588000
1	-17.992701000	46.425207000	98.944300000
1	-16.496127000	45.901763000	98.172092000
1	-27.044265000	46.185594000	96.341640000
1	-24.970238000	42.744431000	99.640686000
1	-24.721725000	45.299674000	100.529661000
1	-24.589970000	50.001675000	98.189546000
1	-24.366010000	50.860430000	95.625253000
1	-24.972559000	41.348259000	92.363483000
1	-23.853127000	45.934910000	90.106184000
1	-23.728942000	48.499382000	90.976174000
1	-18.928353000	49.073197000	104.715914000
1	-15.105363000	54.471352000	105.437814000
1	-12.587065000	50.300428000	105.306498000
1	-11.312050000	52.079239000	101.164246000

1	-11.092610000	52.770896000	94.876340000
1	-15.970877000	50.614364000	91.448325000
1	-24.983045000	34.650269000	97.516980000
1	-24.823291000	35.408430000	99.122789000
1	-10.007719000	38.813711000	94.159002000
1	-14.021672000	38.302229000	98.261567000
1	-18.062651000	33.211800000	100.897927000
1	-8.833947000	45.362240000	93.763553000
1	-15.428602000	45.325730000	94.794689000
8	-13.189343000	48.204496000	101.799765000
1	-13.431471000	48.768767000	101.036564000
1	-14.057102000	47.991951000	102.212067000
8	-21.603270000	48.839391000	98.911949000
1	-21.570038000	49.818797000	98.908235000
1	-21.565340000	48.548306000	97.961588000
8	-16.861001000	46.639140000	94.748847000
1	-16.383945000	47.488301000	94.723532000
1	-17.573318000	46.719860000	95.410823000
8	-13.203961000	48.400891000	96.115638000
1	-12.294121000	48.317107000	95.767135000
1	-13.784471000	48.333635000	95.324116000
8	-21.281222000	47.945421000	96.319545000
1	-21.935870000	47.271478000	96.037978000
1	-20.440386000	47.445690000	96.325827000
8	-14.156044000	47.125463000	98.334014000
1	-13.743172000	47.312358000	97.460345000
1	-14.141410000	48.006699000	98.756475000
8	-20.755127000	51.546595000	99.463320000
1	-21.317201000	52.335113000	99.438452000
1	-20.404903000	51.500192000	100.378650000
8	-11.604383000	46.825080000	90.636802000
1	-12.041887000	46.399883000	91.406389000
1	-10.891947000	46.183285000	90.450202000

^{4}TS1$_{G}$:

6	-14.747002000	38.543952000	97.114402000
6	-14.337258000	39.912574000	96.574184000
8	-15.023194000	40.587517000	95.817506000
6	-16.189659000	38.461244000	97.677091000
6	-17.273581000	38.427729000	96.578138000
6	-16.451706000	39.574127000	98.704269000
6	-17.316599000	37.123512000	95.773199000
1	-14.636159000	37.829457000	96.288165000
1	-16.236114000	37.498885000	98.210002000
1	-18.249053000	38.567662000	97.061734000
1	-17.133896000	39.283016000	95.910670000
1	-15.692772000	39.573210000	99.499233000
1	-16.454116000	40.560092000	98.227305000
1	-17.432740000	39.440114000	99.173165000
1	-16.372327000	36.922360000	95.251584000
1	-17.536877000	36.263039000	96.417664000
1	-18.099444000	37.173655000	95.008294000
7	-13.073781000	40.325038000	96.962283000
6	-12.517386000	41.626921000	96.614786000
6	-11.336483000	41.532241000	95.619566000
8	-10.562846000	42.476454000	95.456258000
6	-12.112712000	42.432788000	97.864851000
6	-13.307343000	42.990636000	98.650832000
6	-12.903640000	43.541272000	100.028708000
6	-14.057226000	44.196976000	100.802337000
7	-14.195005000	45.660858000	100.536588000
1	-12.595662000	39.790304000	97.675728000
1	-13.317290000	42.153404000	96.078895000
1	-11.454033000	43.244235000	97.541870000
1	-11.503744000	41.788124000	98.517894000
1	-13.800880000	43.771687000	98.056615000
1	-14.063198000	42.207771000	98.795309000
1	-12.072468000	44.254654000	99.932397000
1	-12.515856000	42.715012000	100.638020000
1	-13.918091000	44.099757000	101.882456000
1	-15.019881000	43.750966000	100.540099000
1	-15.038881000	46.049555000	101.024327000
1	-13.426987000	46.233903000	100.928007000
1	-14.269700000	45.932924000	99.534553000
7	-11.245095000	40.380518000	94.903489000
6	-10.105754000	40.095335000	94.037208000
6	-10.008877000	40.884189000	92.717584000
8	-8.960786000	40.867419000	92.090423000
1	-11.886710000	39.637384000	95.142981000
1	-9.171525000	40.301439000	94.568022000
7	-11.123613000	41.563504000	92.333967000
6	-11.143858000	42.463582000	91.183862000
6	-10.572659000	43.861332000	91.504594000
8	-10.409993000	44.680400000	90.596311000
6	-12.611258000	42.602202000	90.680577000
6	-12.768897000	43.054095000	89.259329000
6	-12.874994000	44.327565000	88.750696000
7	-12.832845000	42.122891000	88.238450000
6	-12.966549000	42.820106000	87.132363000
7	-13.000802000	44.162561000	87.387689000
1	-11.950927000	41.498305000	92.911974000
1	-10.522315000	42.023973000	90.400627000
1	-13.070177000	41.609948000	90.757059000
1	-13.151363000	43.273728000	91.360333000
1	-12.819933000	45.303414000	89.212145000
1	-13.051547000	42.416306000	86.132236000
1	-13.074353000	44.903684000	86.706296000
7	-10.354105000	44.133746000	92.811749000
6	-10.052448000	45.466666000	93.301498000
6	-10.908979000	45.823367000	94.525630000
6	-12.395801000	45.727668000	94.299336000
6	-13.381431000	45.452546000	95.222436000
7	-12.991081000	45.997077000	93.078042000
6	-14.296230000	45.890062000	93.277150000
7	-14.583388000	45.557123000	94.555197000
1	-10.439001000	43.394978000	93.500891000
1	-10.237424000	46.165341000	92.484797000
1	-10.637886000	45.172443000	95.365178000
1	-10.639174000	46.849407000	94.811761000
1	-13.332624000	45.215302000	96.274101000
1	-15.062773000	46.042495000	92.530696000
6	-16.278019000	50.233786000	92.377695000
6	-15.006541000	49.745047000	93.050524000
8	-15.058003000	49.163956000	94.144539000
6	-17.253888000	49.067788000	92.100907000
6	-18.676020000	49.564420000	91.819417000
16	-19.899406000	48.229607000	91.520963000
6	-19.871149000	47.377442000	93.140531000
1	-16.747478000	50.936975000	93.078281000
1	-17.260192000	48.407166000	92.972117000
1	-16.895995000	48.473733000	91.251739000
1	-19.030056000	50.187784000	92.650992000
1	-18.704645000	50.182339000	90.914440000
1	-20.684289000	46.647625000	93.143996000
1	-18.929984000	46.852700000	93.312861000
1	-20.036778000	48.088103000	93.955896000
7	-13.825782000	49.993288000	92.415766000
6	-12.579211000	49.355120000	92.836962000
6	-11.828127000	50.030985000	93.982221000
8	-10.976459000	49.396123000	94.610307000
1	-13.876579000	50.318836000	91.459271000
1	-11.917681000	49.250289000	91.971209000
1	-12.782631000	48.330681000	93.155953000
7	-12.155694000	51.312563000	94.289840000
6	-11.500919000	52.042297000	95.360457000
6	-12.327804000	52.291006000	96.629695000
8	-12.015733000	53.220711000	97.379128000
1	-12.877674000	51.764089000	93.744458000
1	-10.610796000	51.468291000	95.636402000
7	-13.385150000	51.466612000	96.864032000
6	-14.225481000	51.711596000	98.038185000
6	-13.603371000	51.005492000	99.258703000
8	-13.860637000	49.841443000	99.599110000
6	-15.717223000	51.433096000	97.767115000
6	-16.194892000	49.966918000	97.680096000
6	-16.859029000	49.438158000	98.974587000
6	-18.376691000	49.528683000	98.860021000
7	-19.103168000	49.072410000	100.091805000
1	-13.415691000	50.537665000	96.431770000
1	-14.139816000	52.785656000	98.221181000

1	-16.293371000	51.960344000	98.538435000
1	-15.931740000	51.955492000	96.827640000
1	-15.369544000	49.310166000	97.407720000
1	-16.916729000	49.887502000	96.856683000
1	-16.567612000	48.397404000	99.143905000
1	-16.500109000	50.000302000	99.844182000
1	-18.731052000	48.910158000	98.033652000
1	-18.715194000	50.551286000	98.669256000
1	-18.697296000	48.220362000	100.530296000
1	-19.085884000	49.793404000	100.838976000
1	-20.115883000	48.942432000	99.833714000
7	-12.701242000	51.770737000	99.919568000
6	-12.009381000	51.289473000	101.090943000
6	-12.946728000	51.181520000	102.308135000
8	-14.152127000	51.388093000	102.225703000
1	-12.397318000	52.624549000	99.457163000
1	-11.592554000	50.289475000	100.912763000
7	-12.335074000	50.836680000	103.484196000
6	-13.110742000	50.160578000	104.529671000
6	-14.274296000	50.971271000	105.102357000
8	-15.295683000	50.399216000	105.484266000
1	-11.372573000	50.527958000	103.407391000
1	-13.528778000	49.225792000	104.146818000
7	-14.104792000	52.312952000	105.194228000
6	-15.147495000	53.178013000	105.727765000
6	-16.459998000	53.241990000	104.925883000
8	-17.446820000	53.758177000	105.428679000
1	-13.261271000	52.717882000	104.811882000
1	-15.427521000	52.852359000	106.733347000
7	-16.437997000	52.693543000	103.675137000
6	-17.628174000	52.626453000	102.854775000
6	-18.290655000	51.234309000	102.871126000
8	-19.270902000	51.008244000	102.119783000
6	-17.343266000	53.059203000	101.394236000
6	-16.841432000	54.481607000	101.272475000
6	-15.476669000	54.747547000	101.098919000
6	-17.731281000	55.562497000	101.336168000
6	-15.012183000	56.060126000	100.988371000
6	-17.270833000	56.875188000	101.231559000
6	-15.908332000	57.128032000	101.055485000
1	-15.576847000	52.305491000	103.299646000
1	-18.354743000	53.311489000	103.306630000
1	-16.601112000	52.368907000	100.973643000
1	-18.267245000	52.927350000	100.822687000
1	-14.776483000	53.916010000	101.052236000
1	-18.795252000	55.375656000	101.467294000
1	-13.951112000	56.249280000	100.846670000
1	-17.975687000	57.700477000	101.283022000
1	-15.549468000	58.149765000	100.968784000
7	-17.782186000	50.315300000	103.695177000
6	-18.366878000	48.993936000	103.943100000
6	-17.319258000	47.875576000	103.902552000
6	-16.955766000	47.328284000	102.514700000
8	-15.747513000	47.022446000	102.312812000
8	-17.898971000	47.126732000	101.686323000
1	-17.000521000	50.584671000	104.292254000
1	-19.151728000	48.820898000	103.206548000
1	-16.411159000	48.183227000	104.428310000
1	-17.729966000	47.018460000	104.453948000
6	-24.499001000	35.198803000	98.751251000
6	-23.242069000	34.412786000	99.066071000
8	-22.881983000	34.163155000	100.222111000
1	-24.267678000	36.086064000	98.151755000
7	-22.503079000	33.980557000	98.002813000
6	-21.336509000	33.125269000	98.247729000
6	-20.209980000	33.915590000	98.935659000
8	-19.625451000	34.839936000	98.350612000
6	-20.927171000	32.656845000	96.831516000
6	-21.481574000	33.747677000	95.898655000
6	-22.797812000	34.161048000	96.569126000
1	-21.627096000	32.283941000	98.886916000
1	-21.406771000	31.693284000	96.624810000
1	-19.846681000	32.523109000	96.735251000
1	-21.631474000	33.394393000	94.874149000
1	-20.794994000	34.598575000	95.876135000
1	-23.076433000	35.195198000	96.349081000
1	-23.621865000	33.498669000	96.263784000
7	-19.876379000	33.496947000	100.178353000
6	-18.836516000	34.130248000	100.982697000
6	-19.353685000	35.154393000	102.011446000
6	-20.132174000	36.351157000	101.441947000
6	-19.300003000	37.253985000	100.514807000
7	-20.028018000	38.435773000	100.040947000
6	-21.037665000	38.393356000	99.127653000
7	-21.247429000	37.324978000	98.369708000
7	-21.838087000	39.465531000	99.019958000
1	-20.571997000	32.938571000	100.658210000
1	-18.139331000	34.590484000	100.279278000
1	-19.999476000	34.635737000	102.731700000
1	-18.483188000	35.508888000	102.582628000
1	-20.499253000	36.955290000	102.282456000
1	-21.020993000	35.988350000	100.913754000
1	-18.411294000	37.625475000	101.037390000
1	-18.946435000	36.694329000	99.645887000
1	-20.107142000	39.201547000	100.697248000
1	-20.661540000	36.493955000	98.423858000
1	-21.945613000	37.396389000	97.593253000
1	-21.431251000	40.378997000	99.218013000
1	-22.573308000	39.458844000	98.281409000
16	-26.272764000	45.392174000	94.655816000
8	-22.202357000	44.984211000	94.724727000
26	-23.921525000	45.162648000	94.727515000
6	-24.738940000	42.401118000	96.564013000
7	-24.363811000	43.250284000	94.304290000
6	-24.079806000	43.310625000	91.869999000
7	-23.854119000	45.579245000	92.746303000
8	-23.794408000	39.554277000	97.043998000
6	-23.706511000	48.016797000	92.846603000
7	-23.834059000	47.131509000	95.135040000
8	-23.102648000	37.521220000	96.358579000
6	-23.704041000	47.009452000	97.584958000
7	-24.075540000	44.763656000	96.682147000
6	-24.766794000	42.269549000	95.184329000
6	-25.122212000	41.049985000	94.473257000
6	-24.846802000	41.310774000	93.160117000
6	-24.391358000	42.669403000	93.067548000
6	-25.746664000	39.804167000	95.041571000
6	-24.832293000	38.566496000	95.108743000
6	-23.827507000	38.556266000	96.271093000
6	-23.840866000	44.671475000	91.722662000
6	-23.653184000	45.341143000	90.457970000
6	-23.579819000	46.672457000	90.730168000
6	-23.708823000	46.807258000	92.161358000
6	-23.782188000	48.163998000	94.228928000
6	-23.720088000	49.424405000	94.919730000
6	-23.701481000	49.139521000	96.257091000
6	-23.757791000	47.706881000	96.374326000
6	-23.860217000	45.640108000	97.723469000
6	-23.970337000	44.948082000	98.982423000
6	-24.299020000	43.658318000	98.691884000
6	-24.371111000	43.560668000	97.255791000
1	-24.941554000	41.507916000	97.144043000
1	-24.084469000	42.704541000	90.969622000
1	-23.610486000	48.921813000	92.255597000
1	-23.548525000	47.600079000	98.482867000
1	-26.146563000	40.002335000	96.039494000
1	-26.607517000	39.560607000	94.405281000
1	-25.451793000	37.666398000	95.205545000
1	-24.268076000	38.443352000	94.176495000
6	-19.914876000	42.833637000	98.293108000
7	-19.112811000	43.828359000	98.766325000
8	-20.428281000	41.995608000	99.032010000
16	-17.078592000	43.699233000	96.908536000
6	-20.056771000	42.913378000	96.756645000
7	-20.256522000	44.271607000	96.312613000
8	-19.375229000	46.397776000	96.460124000
16	-18.359117000	42.188741000	96.166751000
6	-19.416104000	45.175535000	96.791122000
8	-17.126926000	45.324263000	99.800288000
6	-18.413142000	44.674685000	97.848681000

6	-21.119519000	41.938714000	96.219557000
6	-20.917416000	41.361078000	94.826672000
6	-20.714174000	42.166991000	93.694586000
6	-20.562062000	41.593619000	92.431890000
6	-20.618649000	40.206309000	92.272119000
6	-20.828728000	39.396049000	93.388264000
6	-20.973783000	39.970100000	94.653749000
6	-17.721039000	45.811501000	98.609862000
1	-18.715634000	43.747804000	99.697933000
1	-21.467314000	44.692439000	95.447552000
1	-17.391341000	45.941412000	100.533044000
1	-20.672346000	43.245081000	93.801052000
1	-20.389540000	42.234387000	91.570552000
1	-20.494566000	39.762710000	91.287466000
1	-20.878379000	38.315519000	93.282342000
1	-21.145538000	39.325881000	95.511014000
1	-21.204774000	41.115357000	96.928922000
1	-22.068587000	42.481261000	96.265104000
1	-18.498594000	46.550335000	98.833049000
1	-16.980720000	46.287987000	97.957057000
1	-26.375090000	46.651971000	95.133396000
1	-24.485724000	42.837155000	99.370971000
1	-23.840914000	45.411400000	99.951648000
1	-23.632594000	49.815449000	97.098448000
1	-23.688733000	50.391815000	94.435274000
1	-24.975953000	40.651290000	92.311098000
1	-23.604492000	44.844637000	89.497796000
1	-23.446966000	47.497430000	90.043195000
1	-18.837507000	49.018214000	104.933215000
1	-14.747600000	54.192272000	105.807869000
1	-12.424577000	49.920555000	105.348555000
1	-11.176515000	51.963847000	101.312448000
1	-11.169136000	53.025082000	95.013454000
1	-16.060149000	50.790277000	91.458335000
1	-25.207615000	34.588297000	98.178138000
1	-24.962878000	35.496182000	99.692672000
1	-10.122502000	39.029375000	93.793674000
1	-14.038123000	38.226652000	97.890936000
1	-18.291843000	33.339561000	101.512112000
1	-8.991585000	45.549538000	93.573132000
1	-15.523124000	45.617175000	94.956615000
8	-13.242967000	48.061797000	101.795095000
1	-13.447251000	48.691520000	101.074078000
1	-14.122394000	47.871292000	102.192939000
8	-21.612492000	49.324588000	99.009450000
1	-21.508847000	50.282401000	99.208668000
1	-21.285725000	49.198212000	98.082152000
8	-16.978932000	46.864790000	95.061821000
1	-16.650827000	47.777396000	95.101788000
1	-17.822999000	46.816595000	95.563881000
8	-13.268866000	48.625983000	96.157799000
1	-12.365826000	48.518996000	95.800155000
1	-13.860946000	48.607993000	95.370926000
8	-20.580681000	48.815526000	96.479973000
1	-21.326212000	48.908858000	95.866249000
1	-20.308191000	47.865654000	96.425681000
8	-14.233436000	47.242933000	98.299013000
1	-13.840465000	47.461573000	97.424589000
1	-14.153552000	48.092335000	98.773925000
8	-20.715763000	51.874982000	99.906280000
1	-21.289258000	52.650401000	99.998390000
1	-20.314021000	51.737025000	100.792132000
8	-11.889238000	47.143916000	90.628954000
1	-12.295780000	46.715219000	91.414282000
1	-11.194597000	46.495537000	90.400503000

^{4}IM1$_{G}$:

6	-13.186707000	38.607422000	96.669173000
6	-13.008472000	40.081157000	96.322311000
8	-13.900096000	40.773031000	95.846825000
6	-14.250825000	38.304418000	97.756354000
6	-15.690421000	38.694756000	97.366025000
6	-13.859518000	38.923109000	99.106959000
6	-16.202471000	38.067551000	96.065408000
1	-13.469852000	38.104120000	95.737655000
1	-14.229908000	37.210800000	97.874438000
1	-16.345446000	38.391949000	98.195217000
1	-15.763177000	39.785372000	97.289642000
1	-12.853276000	38.616377000	99.419361000
1	-13.881593000	40.019922000	99.062255000
1	-14.557875000	38.614537000	99.893089000
1	-15.675484000	38.471396000	95.194943000
1	-16.084968000	36.976126000	96.067391000
1	-17.267009000	38.284992000	95.925481000
7	-11.734382000	40.581652000	96.528459000
6	-11.378519000	41.972043000	96.268297000
6	-10.196622000	42.097427000	95.278105000
8	-9.558678000	43.145250000	95.174992000
6	-11.104984000	42.768721000	97.560661000
6	-12.384058000	43.131997000	98.329034000
6	-12.101489000	43.642691000	99.751280000
6	-13.355089000	44.089062000	100.517342000
7	-13.710494000	45.522741000	100.297857000
1	-11.090383000	40.028699000	97.079887000
1	-12.252245000	42.399942000	95.760805000
1	-10.551492000	43.672480000	97.289177000
1	-10.435985000	42.177656000	98.204197000
1	-12.946199000	43.882135000	97.755268000
1	-13.040472000	42.255077000	98.397861000
1	-11.370265000	44.463331000	99.731215000
1	-11.627752000	42.836588000	100.325608000
1	-13.222132000	43.974646000	101.596488000
1	-14.232793000	43.507864000	100.221598000
1	-14.611686000	45.764812000	100.782279000
1	-13.047622000	46.192664000	100.730705000
1	-13.808581000	45.827349000	99.307682000
7	-9.944300000	41.009684000	94.501189000
6	-8.767306000	40.930335000	93.640730000
6	-8.785559000	41.755790000	92.340197000
8	-7.747514000	41.905619000	91.715019000
1	-10.494681000	40.179610000	94.673634000
1	-7.885119000	41.271486000	94.190055000
7	-9.988270000	42.276008000	91.973234000
6	-10.150143000	43.191673000	90.846790000
6	-9.829186000	44.655778000	91.216721000
8	-9.816836000	45.524141000	90.340895000
6	-11.615816000	43.088903000	90.329798000
6	-11.847690000	43.554788000	88.923347000
6	-12.155636000	44.809993000	88.452694000
7	-11.770990000	42.655260000	87.875704000
6	-12.019980000	43.354354000	86.790921000
7	-12.262565000	44.666814000	87.085855000
1	-10.792891000	42.081954000	92.553856000
1	-9.455295000	42.885503000	90.061727000
1	-11.890695000	42.028815000	90.366495000
1	-12.268622000	43.628495000	91.028381000
1	-12.253973000	45.767525000	88.943484000
1	-12.047336000	42.971209000	85.779581000
1	-12.459985000	45.405401000	86.426570000
7	-9.652887000	44.912053000	92.533904000
6	-9.588338000	46.254928000	93.080276000
6	-10.487379000	46.399939000	94.315859000
6	-11.946631000	46.102342000	94.074776000
6	-12.903787000	45.755906000	95.003421000
7	-12.553758000	46.229073000	92.835694000
6	-13.837408000	45.970781000	93.027473000
7	-14.100183000	45.670932000	94.321511000
1	-9.598768000	44.142574000	93.190757000
1	-9.895722000	46.945317000	92.294068000
1	-10.118532000	45.743106000	95.112865000
1	-10.375081000	47.433217000	94.670181000
1	-12.842068000	45.585259000	96.067250000
1	-14.602873000	45.982870000	92.263979000
6	-16.418150000	50.053238000	92.305357000
6	-15.099246000	49.728887000	92.981813000
8	-15.074399000	49.115935000	94.062890000
6	-17.240227000	48.777062000	92.014477000
6	-18.687206000	49.104764000	91.628677000
16	-19.751915000	47.640976000	91.338802000
6	-19.892323000	46.979198000	93.038665000

1	-16.979721000	50.690774000	93.001086000
1	-17.227956000	48.142653000	92.905416000
1	-16.766441000	48.202906000	91.209675000
1	-19.154071000	49.732178000	92.399351000
1	-18.719132000	49.669499000	90.689732000
1	-20.678718000	46.218320000	93.030118000
1	-18.958946000	46.523475000	93.379710000
1	-20.186014000	47.772969000	93.733529000
7	-13.957580000	50.149279000	92.373781000
6	-12.642803000	49.669172000	92.796862000
6	-11.991600000	50.399845000	93.969912000
8	-11.074995000	49.850891000	94.588069000
1	-14.039710000	50.500173000	91.428348000
1	-11.966355000	49.671982000	91.935923000
1	-12.717390000	48.619643000	93.087757000
7	-12.468458000	51.623493000	94.313210000
6	-11.881211000	52.404243000	95.389137000
6	-12.683372000	52.511369000	96.694118000
8	-12.435818000	53.434438000	97.473844000
1	-13.228311000	52.012296000	93.770944000
1	-10.913599000	51.949326000	95.621543000
7	-13.646637000	51.577542000	96.925923000
6	-14.462108000	51.691887000	98.136940000
6	-13.742988000	50.989582000	99.305950000
8	-13.874682000	49.789102000	99.588341000
6	-15.930736000	51.291859000	97.898988000
6	-16.263533000	49.794956000	97.714153000
6	-16.869833000	49.123741000	98.969635000
6	-18.391907000	49.112531000	98.874340000
7	-19.073658000	48.495062000	100.060879000
1	-13.605296000	50.669720000	96.453225000
1	-14.465558000	52.759808000	98.368423000
1	-16.520151000	51.699551000	98.730152000
1	-16.236359000	51.860340000	97.013139000
1	-15.378220000	49.239502000	97.407358000
1	-16.974464000	49.694166000	96.883948000
1	-16.499774000	48.097969000	99.056809000
1	-16.540285000	49.643990000	99.876467000
1	-18.711312000	48.547375000	97.996412000
1	-18.807249000	50.119629000	98.777147000
1	-18.608698000	47.635475000	100.412389000
1	-19.096792000	49.148491000	100.869440000
1	-20.083356000	48.338992000	99.783537000
7	-12.908633000	51.807095000	99.992147000
6	-12.146868000	51.343638000	101.127165000
6	-13.044566000	51.057848000	102.346913000
8	-14.266121000	51.143142000	102.293078000
1	-12.707323000	52.712814000	99.576097000
1	-11.617451000	50.412562000	100.885079000
7	-12.376157000	50.700081000	103.487290000
6	-13.048057000	49.868596000	104.493206000
6	-14.298069000	50.488444000	105.119349000
8	-15.250459000	49.773954000	105.433874000
1	-11.386009000	50.511539000	103.379205000
1	-13.347025000	48.913316000	104.053914000
7	-14.277795000	51.825643000	105.339264000
6	-15.402838000	52.514130000	105.956542000
6	-16.722066000	52.509945000	105.164517000
8	-17.756020000	52.857439000	105.714793000
1	-13.481388000	52.355679000	105.013028000
1	-15.633739000	52.063537000	106.925762000
7	-16.650267000	52.098253000	103.864036000
6	-17.834514000	51.984487000	103.040690000
6	-18.359465000	50.538983000	102.939316000
8	-19.334483000	50.289220000	102.189049000
6	-17.606341000	52.568303000	101.623207000
6	-17.241376000	54.037117000	101.628566000
6	-15.908745000	54.446159000	101.487799000
6	-18.230409000	55.018060000	101.782808000
6	-15.571994000	55.801560000	101.500367000
6	-17.897759000	56.372683000	101.800360000
6	-16.565793000	56.768762000	101.658198000
1	-15.755475000	51.844511000	103.454534000
1	-18.621366000	52.552176000	103.550558000
1	-16.807352000	51.989908000	101.142200000
1	-18.519938000	52.402962000	101.043852000
1	-15.132983000	53.692771000	101.368071000
1	-19.271338000	54.719337000	101.888997000
1	-14.534193000	56.103428000	101.383558000
1	-18.678146000	57.119018000	101.920457000
1	-16.306077000	57.823588000	101.666913000
7	-17.744386000	49.598797000	103.661629000
6	-18.220687000	48.220414000	103.817107000
6	-17.088988000	47.193227000	103.701735000
6	-16.688717000	46.749156000	102.287327000
8	-15.456185000	46.571100000	102.073738000
8	-17.608657000	46.489776000	101.448387000
1	-16.978138000	49.884749000	104.271205000
1	-18.997234000	48.035673000	103.074757000
1	-16.203301000	47.541401000	104.240328000
1	-17.425404000	46.276248000	104.205112000
6	-25.574215000	36.162513000	99.728895000
6	-24.472069000	35.170370000	100.038411000
8	-23.953375000	35.070940000	101.156782000
1	-25.280426000	36.834048000	98.914441000
7	-24.059769000	34.366344000	99.015591000
6	-23.073248000	33.319430000	99.292362000
6	-21.699917000	33.929294000	99.617395000
8	-21.087577000	34.615012000	98.786404000
6	-23.040860000	32.498812000	97.980769000
6	-23.502816000	33.495555000	96.903755000
6	-24.555406000	34.346841000	97.626533000
1	-23.407797000	32.714704000	100.142960000
1	-23.750637000	31.668124000	98.066917000
1	-22.052705000	32.077169000	97.778042000
1	-23.907218000	33.002700000	96.014676000
1	-22.663310000	34.126851000	96.599705000
1	-24.630834000	35.359328000	97.220501000
1	-25.546760000	33.871317000	97.583084000
7	-21.188564000	33.613380000	100.830872000
6	-19.906684000	34.121914000	101.306457000
6	-20.009252000	35.356494000	102.222336000
6	-20.683422000	36.596998000	101.614483000
6	-19.922310000	37.204898000	100.422879000
7	-20.539333000	38.424257000	99.896738000
6	-21.646710000	38.445134000	99.103659000
7	-22.100149000	37.357617000	98.500276000
7	-22.283590000	39.616908000	98.936980000
1	-21.847671000	33.272268000	101.519473000
1	-19.311889000	34.335266000	100.415645000
1	-20.560409000	35.077055000	103.129764000
1	-18.989118000	35.604454000	102.550970000
1	-20.768500000	37.360584000	102.399767000
1	-21.708953000	36.349442000	101.316973000
1	-18.900468000	37.471947000	100.718308000
1	-19.837282000	36.483217000	99.607170000
1	-20.408420000	39.263800000	100.445280000
1	-21.689155000	36.436454000	98.639791000
1	-22.905619000	37.454711000	97.828756000
1	-21.745615000	40.466534000	99.074479000
1	-23.067890000	39.669842000	98.244901000
16	-26.828239000	45.747209000	94.225374000
8	-22.730598000	44.701087000	94.224650000
26	-24.490307000	45.192039000	94.218931000
6	-25.344941000	42.748460000	96.436272000
7	-25.142552000	43.296407000	94.062045000
6	-25.138195000	43.061083000	91.626713000
7	-24.401729000	45.313712000	92.212028000
8	-24.304849000	39.835905000	97.055840000
6	-23.682408000	47.646549000	92.012771000
7	-23.996252000	47.154022000	94.400805000
8	-24.205837000	37.595433000	96.817086000
6	-24.058329000	47.396237000	96.841544000
7	-24.648889000	45.095758000	96.234607000
6	-25.481149000	42.448602000	95.088778000
6	-25.995077000	41.182315000	94.573062000
6	-25.913051000	41.287187000	93.216601000
6	-25.379023000	42.589333000	92.906325000
6	-26.600598000	40.052757000	95.363041000
6	-25.766160000	38.759932000	95.462631000

6	-24.668248000	38.743421000	96.537422000
6	-24.662537000	44.336034000	91.305896000
6	-24.409911000	44.803337000	89.959876000
6	-23.993855000	46.092904000	90.071755000
6	-23.999311000	46.406076000	91.484255000
6	-23.700228000	47.999177000	93.367042000
6	-23.406244000	49.315842000	93.865066000
6	-23.531474000	49.255581000	95.228490000
6	-23.890807000	47.896277000	95.545864000
6	-24.400424000	46.091616000	97.157532000
6	-24.557355000	45.582137000	98.498773000
6	-24.906115000	44.272140000	98.379271000
6	-24.968595000	43.982685000	96.964629000
1	-25.534183000	41.943916000	97.136724000
1	-25.338865000	42.384250000	90.801535000
1	-23.396306000	48.422650000	91.309727000
1	-23.876764000	48.076029000	97.668827000
1	-26.854268000	40.390280000	96.372168000
1	-27.552601000	39.799098000	94.878882000
1	-26.430506000	37.913741000	95.671148000
1	-25.290344000	38.535315000	94.498985000
6	-19.852971000	42.719096000	98.108459000
7	-18.739328000	43.439757000	98.434764000
8	-20.313252000	41.849270000	98.840158000
16	-16.864512000	43.553684000	96.309664000
6	-20.541913000	43.184623000	96.827223000
7	-20.266357000	44.338148000	96.293596000
8	-19.051655000	46.261219000	96.412843000
16	-17.644728000	41.801000000	95.892831000
6	-19.185746000	45.077310000	96.740083000
8	-16.710330000	44.780117000	99.534977000
6	-18.140409000	44.405222000	97.601643000
6	-21.616014000	42.329721000	96.223786000
6	-21.042112000	41.391136000	95.157831000
6	-20.809197000	41.878729000	93.863918000
6	-20.289023000	41.031777000	92.882523000
6	-20.001781000	39.698123000	93.182056000
6	-20.246994000	39.208040000	94.467245000
6	-20.762717000	40.052103000	95.453403000
6	-17.286540000	45.404667000	98.408090000
1	-18.219754000	43.195124000	99.274851000
1	-22.276263000	45.131270000	94.969103000
1	-16.998478000	45.325629000	100.318627000
1	-21.074559000	42.906562000	93.630356000
1	-20.117719000	41.414057000	91.879629000
1	-19.601369000	39.040266000	92.415318000
1	-20.052073000	38.164768000	94.701388000
1	-20.970166000	39.663613000	96.445396000
1	-22.118815000	41.746499000	96.996458000
1	-22.329542000	43.001374000	95.737354000
1	-17.956145000	46.213650000	98.715979000
1	-16.521980000	45.845492000	97.757374000
1	-26.795466000	46.748183000	95.133883000
1	-25.121335000	43.555480000	99.161414000
1	-24.419901000	46.168515000	99.398163000
1	-23.427462000	50.055906000	95.951235000
1	-23.159667000	50.171194000	93.248997000
1	-26.216678000	40.558382000	92.474712000
1	-24.544048000	44.211506000	89.063624000
1	-23.713160000	46.783186000	89.286844000
1	-18.685239000	48.138542000	104.807065000
1	-15.115852000	53.553810000	106.134650000
1	-12.326562000	49.670516000	105.292845000
1	-11.395241000	52.097961000	101.379103000
1	-11.695262000	53.430626000	95.060355000
1	-16.263722000	50.634231000	91.388440000
1	-26.488562000	35.643899000	99.415310000
1	-25.781209000	36.738695000	100.631805000
1	-8.609702000	39.881698000	93.373826000
1	-12.229720000	38.170574000	96.983490000
1	-19.402595000	33.315142000	101.851687000
1	-8.557794000	46.510215000	93.360508000
1	-15.035635000	45.654714000	94.729933000
8	-13.091116000	47.941724000	101.669933000
1	-13.348359000	48.606587000	100.998592000
1	-13.950474000	47.638521000	102.041883000
8	-21.567791000	48.729339000	99.013104000
1	-21.535993000	49.674907000	99.282810000
1	-21.318578000	48.713814000	98.055886000
8	-16.517761000	46.826452000	95.142503000
1	-16.246532000	47.729763000	94.895722000
1	-17.403273000	46.872503000	95.544283000
8	-13.260703000	48.792131000	96.103990000
1	-12.344763000	48.808192000	95.763213000
1	-13.829179000	48.720484000	95.303826000
8	-20.657613000	48.591003000	96.371478000
1	-21.418793000	48.711418000	95.774079000
1	-20.344012000	47.677221000	96.221310000
8	-14.049033000	47.220030000	98.187120000
1	-13.716745000	47.547305000	97.320981000
1	-14.049014000	48.037234000	98.721932000
8	-20.855066000	51.265795000	100.076724000
1	-21.509610000	51.959317000	100.249074000
1	-20.440523000	51.070436000	100.945440000
8	-11.655435000	47.713137000	90.479709000
1	-11.987478000	47.157911000	91.218479000
1	-10.863541000	47.207043000	90.211572000

Deprotonated model:

$^4Re_{Bm}$:

6	137.752946000	3.613250000	13.405308000
6	139.187456000	3.622455000	12.873253000
8	140.092631000	2.980918000	13.408625000
6	137.670251000	3.319067000	14.911516000
6	138.253815000	4.473089000	15.739824000
6	136.220759000	3.019275000	15.320987000
1	137.248185000	4.559706000	13.170383000
1	138.283951000	2.429669000	15.100295000
1	138.216596000	4.247298000	16.814292000
1	137.695032000	5.403099000	15.566386000
1	139.300098000	4.650466000	15.472262000
1	136.152126000	2.812740000	16.399242000
1	135.564913000	3.868375000	15.085211000
1	135.828111000	2.143888000	14.789038000
7	139.367979000	4.370090000	11.747876000
6	140.597939000	4.349978000	10.977578000
6	140.355268000	3.980458000	9.497111000
6	139.812662000	2.583132000	9.291598000
6	140.671866000	1.501326000	9.068234000
6	138.433041000	2.322869000	9.345338000
6	140.182514000	0.205139000	8.900562000
6	137.927456000	1.033687000	9.182434000
6	138.806089000	-0.030490000	8.958694000
8	138.267059000	-1.284410000	8.795249000
1	138.579626000	4.867449000	11.341656000
1	141.260172000	3.628214000	11.462457000
1	141.309365000	4.092441000	8.964823000
1	139.663569000	4.715827000	9.068846000
1	141.745886000	1.670651000	9.020584000
1	137.736722000	3.140630000	9.516204000
1	140.870705000	-0.620834000	8.722544000
1	136.859102000	0.847424000	9.229188000
1	138.994486000	-1.914508000	8.677116000
6	135.184466000	6.232696000	18.126461000
6	133.927253000	6.230756000	17.266783000
8	133.430968000	7.268818000	16.826094000
6	135.401032000	7.550646000	18.871013000
1	135.158856000	5.385424000	18.824173000
1	134.597044000	7.738923000	19.591306000
1	136.349601000	7.527560000	19.416826000
1	135.423775000	8.387515000	18.168835000
7	133.371646000	4.998473000	17.032686000
6	132.505685000	4.732848000	15.879734000
6	131.142144000	5.414537000	15.854725000
8	130.469962000	5.380553000	14.824319000
1	133.918340000	4.205770000	17.345545000
1	133.006551000	5.032536000	14.948564000
7	130.714839000	6.065464000	16.971906000
6	129.581080000	6.970481000	16.871067000
6	129.744367000	8.103957000	15.840222000
8	128.738701000	8.639655000	15.363310000
1	131.380244000	6.200258000	17.719011000
1	128.684989000	6.421347000	16.573999000
7	131.008228000	8.451931000	15.506591000
6	131.257827000	9.467584000	14.490470000
6	130.689216000	9.093336000	13.116311000
8	130.464451000	10.001152000	12.294681000
6	132.753355000	9.797139000	14.365974000
6	133.371376000	10.474349000	15.596911000
16	132.525256000	11.987611000	16.211720000
6	132.634638000	13.079312000	14.749010000
1	131.804772000	7.960945000	15.911645000
1	130.714793000	10.375626000	14.773823000
1	133.315125000	8.878250000	14.151488000
1	132.868508000	10.435318000	13.484326000
1	134.417181000	10.718571000	15.379130000
1	133.376353000	9.793893000	16.453534000
1	132.180562000	14.031997000	15.036073000
1	132.085335000	12.671114000	13.896015000
1	133.675852000	13.260650000	14.463708000
7	130.398800000	7.805285000	12.870632000
6	129.775632000	7.404060000	11.616087000
6	128.490561000	8.194736000	11.365706000
8	128.112558000	8.430577000	10.205284000
6	129.473765000	5.899230000	11.619035000
1	130.647330000	7.082285000	13.540879000
1	130.450213000	7.630216000	10.784484000
1	129.011348000	5.616697000	10.668585000
1	128.802434000	5.627953000	12.439883000
1	130.402172000	5.333664000	11.735443000
7	127.754831000	8.600404000	12.422679000
6	126.543048000	9.365097000	12.186090000
6	126.753158000	10.748820000	11.548391000
8	125.828477000	11.283270000	10.927654000
1	128.106034000	8.490991000	13.374008000
1	126.029254000	9.498214000	13.143002000
1	125.879997000	8.819675000	11.509564000
7	127.950410000	11.342533000	11.759477000
6	128.303640000	12.599140000	11.129199000
6	129.191824000	12.528349000	9.874408000
8	129.362497000	13.553012000	9.216810000
1	128.691545000	10.825891000	12.229651000
1	127.382174000	13.103292000	10.833171000
7	129.751497000	11.322270000	9.570810000
6	130.674578000	11.193385000	8.451205000
6	130.141418000	10.360742000	7.268690000
8	130.708778000	10.472257000	6.174363000
6	132.040641000	10.625502000	8.879543000
6	132.799805000	11.489451000	9.843377000
6	132.675347000	12.829001000	10.131852000
7	133.856493000	10.958844000	10.563334000
6	134.360375000	11.955958000	11.264054000
7	133.675949000	13.109493000	11.043438000
1	129.786883000	10.615525000	10.300158000
1	130.801577000	12.198198000	8.045030000
1	132.633074000	10.494364000	7.965780000
1	131.926786000	9.622407000	9.307071000
1	131.968971000	13.575845000	9.802124000
1	135.204647000	11.899587000	11.935880000
1	133.912553000	14.017080000	11.421489000
7	129.112868000	9.520070000	7.514022000
6	128.543389000	8.626198000	6.499801000
6	127.174935000	9.175795000	6.037708000
8	127.070800000	9.805808000	4.977190000
6	128.537333000	7.158733000	6.969095000
8	127.640257000	6.877536000	8.035008000
1	128.700489000	9.522014000	8.442297000
1	129.196741000	8.692595000	5.630379000
1	129.567763000	6.881764000	7.231530000
1	128.226091000	6.529275000	6.128911000
1	127.981892000	7.295173000	8.852393000
7	126.151886000	8.976613000	6.896740000
6	124.832155000	9.561642000	6.729784000
6	124.472331000	10.477535000	7.898242000
8	124.406677000	9.716510000	9.091977000
1	126.308538000	8.335123000	7.669886000
1	124.082335000	8.762764000	6.655860000
1	125.223488000	11.275806000	7.976355000
1	123.499816000	10.953843000	7.680605000
1	124.773520000	10.266123000	9.813640000
7	133.385737000	14.318461000	4.498573000
6	134.024134000	15.382410000	5.309666000
6	135.447166000	14.998106000	5.782618000
8	136.064446000	15.729126000	6.566659000
6	133.070332000	15.631194000	6.499087000
6	132.325280000	14.294849000	6.625133000
6	132.135014000	13.875058000	5.163698000
1	134.161085000	16.303399000	4.727300000
1	132.364482000	16.433252000	6.250108000
1	133.617317000	15.930902000	7.396157000
1	132.951685000	13.561034000	7.145788000
1	131.376323000	14.368222000	7.164779000
1	131.987931000	12.795504000	5.055620000
1	131.251691000	14.377507000	4.739575000
7	135.924718000	13.860306000	5.242579000
6	137.287656000	13.371237000	5.413092000
6	137.410097000	11.837356000	5.302008000

6	136.988630000	11.082872000	6.580398000
6	138.864840000	11.478902000	4.952208000
6	135.523677000	11.230907000	7.007003000
1	135.268083000	13.382941000	4.629708000
1	137.661638000	13.710385000	6.382189000
1	136.766559000	11.501712000	4.471526000
1	137.647250000	11.407981000	7.396523000
1	137.198290000	10.015586000	6.419279000
1	139.175306000	11.940258000	4.006533000
1	138.987602000	10.394213000	4.853327000
1	139.541918000	11.826791000	5.741389000
1	135.315137000	10.614613000	7.889709000
1	134.840394000	10.912393000	6.209275000
1	135.274670000	12.264609000	7.265042000
16	134.358368000	4.729417000	2.343234000
1	133.673324000	5.828991000	1.955165000
6	140.803855000	12.808080000	10.050633000
6	139.759498000	13.576898000	9.245421000
8	139.382317000	13.200536000	8.137177000
6	140.172494000	11.938384000	11.158500000
6	141.172296000	11.038650000	11.915403000
6	140.512079000	10.460050000	13.176929000
6	141.707794000	9.902786000	11.029435000
1	141.339834000	12.183388000	9.332530000
1	139.676792000	12.597816000	11.885677000
1	139.387855000	11.304050000	10.724787000
1	142.022232000	11.667416000	12.227563000
1	141.222784000	9.848183000	13.746023000
1	140.152368000	11.256698000	13.841321000
1	139.661588000	9.824826000	12.905393000
1	142.418378000	9.279249000	11.585224000
1	142.224911000	10.275128000	10.137730000
1	140.881314000	9.260594000	10.701857000
7	139.262895000	14.703001000	9.844449000
6	138.163282000	15.473499000	9.275442000
6	136.758704000	14.989057000	9.700778000
6	136.433912000	15.236417000	11.157041000
6	136.883622000	14.366952000	12.166497000
6	135.686389000	16.360702000	11.545550000
6	136.607572000	14.617171000	13.513307000
6	135.403062000	16.612604000	12.890945000
6	135.865508000	15.742831000	13.881403000
1	139.540621000	14.899286000	10.795944000
1	138.228259000	15.401648000	8.189043000
1	136.041863000	15.499718000	9.049674000
1	136.687546000	13.920100000	9.468774000
1	137.446265000	13.479316000	11.889068000
1	135.324958000	17.044665000	10.780598000
1	136.968899000	13.930261000	14.274640000
1	134.819065000	17.488025000	13.164096000
1	135.646590000	15.936204000	14.928032000
7	134.471292000	3.281320000	5.004703000
7	133.211484000	5.825449000	4.882320000
7	132.463130000	2.199654000	3.298739000
6	134.376788000	0.967937000	4.204706000
6	135.187220000	5.280920000	6.227903000
6	131.346807000	7.096469000	3.912483000
6	130.424697000	2.728894000	2.046273000
6	134.960273000	1.997586000	4.924597000
6	134.213049000	6.153491000	5.767194000
6	130.764253000	6.048244000	3.202040000
6	131.416274000	1.857815000	2.485245000
8	131.854929000	3.588197000	5.503126000
6	136.154321000	1.866276000	5.725129000
6	134.105812000	7.540528000	6.153558000
6	129.578819000	6.197220000	2.392785000
6	131.497856000	0.465434000	2.120597000
6	136.372527000	3.076653000	6.303876000
6	133.034473000	8.055172000	5.492717000
6	129.310944000	4.967967000	1.871739000
6	132.614651000	-0.029868000	2.724326000
6	135.318178000	3.950151000	5.849022000
6	132.475629000	6.979449000	4.708265000
6	130.336083000	4.077352000	2.361396000
6	133.205530000	1.063290000	3.455512000
7	131.212798000	4.757610000	3.169404000
26	132.760567000	3.982002000	4.206667000
1	134.868453000	-0.000059000	4.236196000
1	135.915157000	5.665757000	6.934730000
1	130.862343000	8.068282000	3.818538000
1	129.657888000	2.319831000	1.394565000
6	133.662396000	7.103593000	10.049457000
7	134.625380000	8.038847000	10.304986000
8	132.505725000	7.385005000	9.723038000
16	135.775619000	7.168454000	12.749219000
6	134.178233000	5.677955000	10.004615000
7	135.447225000	5.375774000	9.957602000
8	137.591411000	6.154648000	10.079852000
16	133.900870000	6.307209000	12.861112000
6	133.121529000	4.645442000	9.700008000
8	138.135650000	8.521857000	11.331193000
6	133.510749000	3.191354000	9.843201000
6	133.290994000	2.303182000	8.783338000
6	133.584879000	0.943747000	8.923114000
6	134.103022000	0.456615000	10.123982000
6	134.324334000	1.339315000	11.186572000
6	134.030175000	2.695535000	11.048481000
6	136.372298000	6.359497000	10.247352000
6	135.885765000	7.662514000	10.832533000
6	136.901881000	8.806167000	10.708733000
1	132.880149000	2.670468000	7.844690000
1	133.409806000	0.268911000	8.088662000
1	132.251732000	4.881213000	10.323155000
1	132.783125000	4.840544000	8.672021000
1	134.334324000	-0.600464000	10.232890000
1	134.728610000	0.967974000	12.125876000
1	134.203771000	3.382980000	11.872890000
1	137.030298000	9.020126000	9.634467000
1	136.490427000	9.701082000	11.183583000
1	134.309144000	9.010612000	10.446005000
1	138.397789000	7.653554000	10.958927000
8	129.583362000	9.897551000	3.570540000
1	128.652334000	9.889062000	3.878176000
1	130.054796000	10.237393000	4.355048000
1	137.214900000	2.833765000	12.845896000
1	141.086279000	5.334918000	11.019256000
1	136.026463000	6.042277000	17.445666000
1	129.396732000	7.408745000	17.856742000
1	128.819173000	13.242013000	11.851702000
1	124.837610000	10.118928000	5.789942000
1	133.208554000	14.628999000	3.549111000
1	137.928205000	13.835631000	4.647291000
1	141.527833000	13.504254000	10.497401000
1	136.728849000	0.956143000	5.839215000
1	134.765495000	8.034391000	6.855589000
1	129.050989000	7.133308000	2.264178000
1	130.789727000	-0.048196000	1.482519000
1	137.160447000	3.363270000	6.986440000
1	132.607919000	9.048061000	5.546228000
1	128.502644000	4.673497000	1.214066000
1	133.014797000	-1.035267000	2.686682000
1	138.301168000	16.524057000	9.555355000
1	132.336617000	3.653261000	15.830136000

^{4}TS7$_{Bm}$:

6	136.691849000	2.839938000	13.097571000
6	138.214093000	2.980155000	13.144235000
8	138.920072000	2.299705000	13.890156000
6	136.093456000	2.185385000	14.351842000
6	136.255854000	3.090109000	15.582872000
6	134.618189000	1.831964000	14.115455000
1	136.223835000	3.816119000	12.914412000
1	136.658011000	1.263654000	14.539902000
1	135.827552000	2.612835000	16.477024000
1	135.748273000	4.050716000	15.422391000
1	137.314037000	3.284829000	15.781635000
1	134.176372000	1.359012000	15.003321000
1	134.035109000	2.730963000	13.877105000
1	134.502792000	1.133745000	13.277304000
7	138.720344000	3.896579000	12.270307000

6	140.141427000	4.028631000	12.009858000
6	140.497011000	3.786664000	10.525153000
6	140.119260000	2.410396000	10.023490000
6	140.999297000	1.328706000	10.139384000
6	138.856256000	2.171854000	9.457423000
6	140.640620000	0.049874000	9.710419000
6	138.482992000	0.900339000	9.025870000
6	139.377454000	-0.166247000	9.152937000
8	138.969249000	-1.400343000	8.707056000
1	138.083924000	4.406296000	11.664468000
1	140.642212000	3.302217000	12.654722000
1	141.577419000	3.949371000	10.410497000
1	139.995106000	4.549612000	9.917959000
1	141.985465000	1.482776000	10.573078000
1	138.153489000	2.995268000	9.352982000
1	141.343348000	-0.777189000	9.807198000
1	137.510186000	0.729547000	8.576035000
1	139.681122000	-2.036398000	8.876761000
6	135.382519000	6.237269000	18.170200000
6	134.103126000	6.304263000	17.345557000
8	133.659071000	7.368187000	16.908793000
6	135.695901000	7.545541000	18.897281000
1	135.326516000	5.397893000	18.875359000
1	134.923159000	7.785878000	19.635861000
1	136.655159000	7.470329000	19.419245000
1	135.749427000	8.374806000	18.187750000
7	133.466275000	5.108075000	17.143002000
6	132.546677000	4.884826000	16.022532000
6	131.218390000	5.631476000	16.037787000
8	130.494642000	5.591946000	15.042943000
1	133.978851000	4.287018000	17.439280000
1	133.030631000	5.157872000	15.072961000
7	130.883974000	6.354843000	17.143407000
6	129.813873000	7.334458000	17.043054000
6	130.019783000	8.408422000	15.958075000
8	129.038481000	8.996295000	15.491074000
1	131.598983000	6.492358000	17.842652000
1	128.870915000	6.839672000	16.801289000
7	131.291447000	8.647332000	15.565538000
6	131.577150000	9.600278000	14.499458000
6	130.921999000	9.222072000	13.166765000
8	130.725978000	10.116138000	12.322268000
6	133.088200000	9.796280000	14.300425000
6	133.808421000	10.486738000	15.466363000
16	133.106075000	12.094428000	16.022827000
6	133.179068000	13.073926000	14.481177000
1	132.066917000	8.127143000	15.975006000
1	131.122639000	10.560489000	14.766196000
1	133.562195000	8.822923000	14.115654000
1	133.218295000	10.373997000	13.380196000
1	134.859820000	10.634439000	15.195519000
1	133.796207000	9.856762000	16.360915000
1	132.808412000	14.072767000	14.727938000
1	132.546305000	12.647196000	13.697933000
1	134.205949000	13.165464000	14.113482000
7	130.535504000	7.950336000	12.978632000
6	129.840867000	7.553748000	11.761686000
6	128.576302000	8.390247000	11.554586000
8	128.138658000	8.592930000	10.408625000
6	129.496113000	6.059084000	11.795218000
1	130.763968000	7.234633000	13.663565000
1	130.485534000	7.745484000	10.898488000
1	128.962130000	5.786993000	10.879835000
1	128.875503000	5.810473000	12.661842000
1	130.417141000	5.472293000	11.847992000
7	127.930464000	8.883087000	12.632008000
6	126.756371000	9.714268000	12.429668000
6	127.019333000	11.061346000	11.735275000
8	126.092905000	11.644910000	11.162979000
1	128.330854000	8.795903000	13.566359000
1	126.301414000	9.909411000	13.405427000
1	126.027793000	9.191269000	11.804772000
7	128.268674000	11.568793000	11.841889000
6	128.671310000	12.771124000	11.139446000
6	129.474853000	12.589527000	9.838975000
8	129.646652000	13.568976000	9.116329000
1	128.999539000	11.013922000	12.283404000
1	127.771239000	13.328269000	10.874574000
7	129.962592000	11.343969000	9.570484000
6	130.803632000	11.105499000	8.402942000
6	130.145334000	10.254354000	7.297218000
8	130.653011000	10.267590000	6.168875000
6	132.149131000	10.455386000	8.777529000
6	133.020487000	11.293023000	9.666386000
6	132.994049000	12.644350000	9.922616000
7	134.080231000	10.714823000	10.344446000
6	134.681476000	11.696540000	10.988027000
7	134.059206000	12.885472000	10.769553000
1	130.022525000	10.681282000	10.339048000
1	130.977224000	12.079515000	7.941587000
1	132.674055000	10.246895000	7.837686000
1	131.986068000	9.478654000	9.248272000
1	132.319004000	13.425617000	9.607800000
1	135.556104000	11.599685000	11.616429000
1	134.360585000	13.784305000	11.122957000
7	129.079957000	9.501960000	7.653990000
6	128.385820000	8.599358000	6.730403000
6	127.006402000	9.204943000	6.382754000
8	126.867559000	9.919531000	5.382087000
6	128.381131000	7.143477000	7.234088000
8	127.573140000	6.894373000	8.378064000
1	128.729226000	9.582932000	8.603656000
1	128.955803000	8.616681000	5.802053000
1	129.424647000	6.849137000	7.415180000
1	127.989895000	6.505215000	6.435438000
1	127.949897000	7.382721000	9.140398000
7	126.014953000	8.953777000	7.266397000
6	124.708968000	9.589490000	7.197108000
6	124.511335000	10.630201000	8.297938000
8	124.493547000	9.989308000	9.561264000
1	126.215078000	8.297420000	8.017204000
1	123.930340000	8.821616000	7.285440000
1	125.320271000	11.371714000	8.238323000
1	123.559993000	11.159444000	8.109002000
1	124.911450000	10.597615000	10.204118000
7	133.087897000	14.378194000	4.318233000
6	133.693140000	15.529464000	5.031054000
6	135.171477000	15.274898000	5.417645000
8	135.786305000	16.077052000	6.130704000
6	132.809254000	15.765147000	6.273816000
6	132.202520000	14.377731000	6.529166000
6	131.943242000	13.857593000	5.109957000
1	133.716104000	16.424092000	4.394654000
1	132.015044000	16.483641000	6.035538000
1	133.388704000	16.167093000	7.108370000
1	132.934104000	13.734832000	7.034054000
1	131.294980000	14.398810000	7.139745000
1	131.893825000	12.764617000	5.064547000
1	130.984824000	14.248733000	4.735446000
7	135.696207000	14.156760000	4.882540000
6	137.087595000	13.745823000	5.016264000
6	137.276489000	12.225676000	4.837193000
6	136.955213000	11.406504000	6.105433000
6	138.721679000	11.940093000	4.396477000
6	135.560708000	11.605207000	6.710219000
1	135.031515000	13.599887000	4.350588000
1	137.459824000	14.058232000	5.996050000
1	136.603226000	11.894927000	4.028677000
1	137.711432000	11.647249000	6.862915000
1	137.085158000	10.344125000	5.854877000
1	138.958501000	12.435635000	3.446815000
1	138.885514000	10.864262000	4.264424000
1	139.428201000	12.298019000	5.155100000
1	135.403356000	10.916863000	7.548557000
1	134.767185000	11.424165000	5.973703000
1	135.426378000	12.622583000	7.090919000
16	134.312875000	4.182003000	2.765609000
1	133.123715000	4.393144000	2.158152000
6	140.513429000	13.107074000	9.770615000
6	139.525582000	13.888842000	8.910066000

8	139.136864000	13.468927000	7.822133000
6	139.799065000	12.135863000	10.735171000
6	140.755586000	11.240227000	11.551622000
6	140.022998000	10.598361000	12.740761000
6	141.403311000	10.148885000	10.684089000
1	141.153355000	12.551898000	9.079597000
1	139.185117000	12.724866000	11.431971000
1	139.106419000	11.503952000	10.163451000
1	141.553286000	11.887477000	11.951238000
1	140.722616000	10.031323000	13.367068000
1	139.548068000	11.357041000	13.376334000
1	139.253575000	9.897340000	12.396455000
1	142.131041000	9.571579000	11.267132000
1	141.930545000	10.561886000	9.816248000
1	140.637743000	9.451107000	10.323842000
7	139.087368000	15.072867000	9.438409000
6	138.025000000	15.860052000	8.822871000
6	136.601344000	15.467073000	9.278429000
6	136.312690000	15.793662000	10.726533000
6	136.673796000	14.913483000	11.762174000
6	135.704467000	17.008564000	11.081781000
6	136.444857000	15.239247000	13.102977000
6	135.471621000	17.337108000	12.418379000
6	135.842703000	16.454460000	13.435690000
1	139.368668000	15.307887000	10.379938000
1	138.086142000	15.721167000	7.742699000
1	135.902840000	15.986024000	8.614034000
1	136.476400000	14.394057000	9.092495000
1	137.138084000	13.962486000	11.511046000
1	135.408029000	17.700498000	10.296554000
1	136.738621000	14.544073000	13.885684000
1	134.994073000	18.281932000	12.665519000
1	135.659764000	16.707823000	14.476399000
7	135.294288000	3.162882000	5.346047000
7	134.221521000	5.792166000	5.486907000
7	132.779320000	2.185408000	4.389209000
6	134.814370000	0.847725000	4.677127000
6	136.458987000	5.124712000	6.241113000
6	132.309183000	7.202361000	4.865720000
6	130.500021000	2.844369000	3.778622000
6	135.646550000	1.850981000	5.154768000
6	135.451779000	6.054445000	6.035353000
6	131.474798000	6.193151000	4.402023000
6	131.509868000	1.904754000	3.964657000
8	132.969380000	3.764589000	6.625354000
6	137.010902000	1.642272000	5.576061000
6	135.575419000	7.466347000	6.316123000
6	130.141516000	6.417417000	3.890034000
6	131.390238000	0.498906000	3.658357000
6	137.467064000	2.838226000	6.040573000
6	134.416962000	8.051752000	5.908834000
6	129.621852000	5.185488000	3.627851000
6	132.616219000	-0.055583000	3.879232000
6	136.386000000	3.781639000	5.890559000
6	133.576493000	6.998594000	5.391151000
6	130.640287000	4.213611000	3.950344000
6	133.478599000	1.007540000	4.334191000
7	131.768440000	4.853210000	4.410453000
26	133.492268000	4.003994000	4.996956000
1	135.238982000	-0.147270000	4.582477000
1	137.379919000	5.473737000	6.696747000
1	131.925647000	8.217445000	4.848546000
1	129.541128000	2.483481000	3.418449000
6	133.545477000	6.843421000	9.956289000
7	134.575386000	7.706040000	10.180048000
8	132.396597000	7.204149000	9.681807000
16	135.790976000	6.873713000	12.608989000
6	133.960445000	5.381167000	9.895794000
7	135.193607000	4.980827000	9.896753000
8	137.396387000	5.580305000	9.986368000
16	133.861160000	6.194396000	12.895908000
6	132.807831000	4.425178000	9.669986000
8	138.155282000	8.009511000	11.006528000
6	133.080704000	3.082122000	9.040033000
6	132.271787000	2.700864000	7.893942000
6	132.338586000	1.335345000	7.441472000
6	133.294727000	0.477996000	7.942483000
6	134.157460000	0.906683000	8.969428000
6	134.029940000	2.192783000	9.519354000
6	136.203155000	5.904417000	10.138036000
6	135.832230000	7.265910000	10.661169000
6	136.911480000	8.332623000	10.425852000
1	131.308027000	3.194782000	7.797340000
1	131.644946000	1.008436000	6.674851000
1	132.350552000	4.278589000	10.661087000
1	132.063153000	4.971558000	9.085363000
1	133.368192000	-0.537420000	7.562462000
1	134.908719000	0.228379000	9.365486000
1	134.675274000	2.494361000	10.336179000
1	136.997189000	8.478586000	9.335606000
1	136.576870000	9.276354000	10.864564000
1	134.341383000	8.703778000	10.287331000
1	138.339284000	7.100649000	10.690691000
8	129.203178000	9.649713000	3.705904000
1	128.323664000	9.816420000	4.104048000
1	129.807687000	9.971332000	4.400980000
1	136.462836000	2.219453000	12.218373000
1	140.487372000	5.031744000	12.299035000
1	136.192384000	5.992732000	17.468298000
1	129.697525000	7.824973000	18.014421000
1	129.272901000	13.402438000	11.803523000
1	124.626093000	10.060329000	6.214454000
1	132.797148000	14.634369000	3.380562000
1	137.690358000	14.277710000	4.264246000
1	141.153724000	13.790421000	10.345652000
1	137.531345000	0.693760000	5.534036000
1	136.450981000	7.931380000	6.751418000
1	129.702152000	7.401021000	3.744056000
1	130.486430000	0.020247000	3.301889000
1	138.436040000	3.072381000	6.461036000
1	134.134851000	9.095204000	5.944115000
1	128.644471000	4.938747000	3.231288000
1	132.924799000	-1.085412000	3.747758000
1	138.212478000	16.917470000	9.040974000
1	132.326333000	3.814725000	15.979167000
